# An artificial intelligence enabled chemical synthesis robot for exploration and optimization of nanomaterials

**DOI:** 10.1126/sciadv.abo2626

**Published:** 2022-10-07

**Authors:** Yibin Jiang, Daniel Salley, Abhishek Sharma, Graham Keenan, Margaret Mullin, Leroy Cronin

**Affiliations:** ^1^ School of Chemistry, University of Glasgow, University Avenue, Glasgow G12 8QQ, UK.; ^2^ Glasgow Imaging Facility, Institute of Infection Immunity and Inflammation, College of Medical Veterinary and Life Sciences, University of Glasgow, University Avenue, Glasgow G12 8QQ, UK.

## Abstract

We present an autonomous chemical synthesis robot for the exploration, discovery, and optimization of nanostructures driven by real-time spectroscopic feedback, theory, and machine learning algorithms that control the reaction conditions and allow the selective templating of reactions. This approach allows the transfer of materials as seeds between cycles of exploration, opening the search space like gene transfer in biology. The open-ended exploration of the seed-mediated multistep synthesis of gold nanoparticles (AuNPs) via in-line ultraviolet-visible characterization led to the discovery of five categories of nanoparticles by only performing ca. 1000 experiments in three hierarchically linked chemical spaces. The platform optimized nanostructures with desired optical properties by combining experiments and extinction spectrum simulations to achieve a yield of up to 95%. The synthetic procedure is outputted in a universal format using the chemical description language (χDL) with analytical data to produce a unique digital signature to enable the reproducibility of the synthesis.

## Teaser

An autonomous system is implemented to achieve the open-ended exploration and target-specific optimization of AuNPs.

## INTRODUCTION

Nanomaterials have unique size- and shape-controlled physical and chemical properties with applications in areas of medicine (*1*), electronics (*2*), catalysis (*3*), and quantum technologies (*4*). Controlling the morphology of nanomaterials is crucial for tuning their unique characteristics such as optical (*5*), electrical (*6*), and magnetic (*7*) properties. Although certain types of nanoparticles such as Au nanorods can be reliably fabricated (*8*), the synthesis of nanomaterials often suffers from irreproducibility (*9*), low yield (*10*), and polydispersity (*11*). Various bottom-up fabrication methods including electrochemical (*12*), photochemical (*13*), bio-templated (*14*), and seed-mediated (*15*) synthesis have been developed to create nanomaterials with desired properties. Despite the availability of various synthetic routes, finding optimal conditions for a target nanostructure with high shape yield and monodispersity is a huge challenge. This is due to the high dimensionality and sensitivity to the synthetic conditions such as reagent concentrations (*16*), order of reagent addition (*17*), temperature (*18*), and mixing rate (*19*). Despite this sensitivity, a standard, robust, and unique digital signature that originates from both the synthetic procedure and validation of the synthesis is still lacking. These problems become more pronounced when multistep synthesis (*20*) is required to achieve the targeted nanostructure.

The development of autonomous precision robotic architectures capable of parallel experiments in a closed-loop approach guided by machine learning (ML) algorithms can provide a viable path to address high dimensionality and sensitivity to synthetic conditions. Recently, various autonomous platforms have been developed for chemical synthesis (*21*, *22*), product separation (*23*, *24*), and in-line characterization (*25*). It has also been demonstrated to accelerate material discovery by combining autonomous platforms with customized ML algorithms (*24*–*31*). However, recent automation of nanomaterials synthesis (*32*) focuses on user-defined target-specific optimization (*31*, *33*) without exploration and can still require manual steps (*34*). A system that unbiasedly performs open-ended exploration and searches for a diversified set of high-performance products is still lacking. Quality diversity (QD) algorithms (*35*) such as novelty search with local competition (*36*) or multidimensional archive of phenotypic elites (MAP-Elites) (*37*) have been applied in several areas including real-time decision-making (*38*), adaptive robotic control (*39*), de novo drug molecule discovery (*40*), and novel protocell behavior search (*41*). In contrast to the classic optimization algorithms that target a single highest performance solution, QD algorithms can find solutions with both diversified behavior and high performance, thus suitable to explore the chemical space and facilitate the diversity of products.

A crucial requirement for a closed-loop autonomous system is the selection of appropriate characterization techniques (*32*). Various characterization techniques such as atomic force microscopy (*42*), scanning electron microscopy (*43*), transmission electron microscopy (TEM) (*44*), dynamic light scattering (*45*), and small-angle x-ray scattering (*46*) are widely applied to investigate the morphology of nanomaterials. Although electron microscopy can provide detailed information on nanostructures, it is still impractical to implement it in the closed loop because of its cost and complexity. Considering the strong dependence of electromagnetic properties of metallic nanoparticles on the morphology and composition, in-line optical spectroscopy such as ultraviolet-visible (UV-Vis) and infrared (IR) are optimal and practical characterization techniques and thus can be used as structural indicators. For open-ended exploration, increasing the diversity of spectral patterns could lead to the discovery of nanomaterials with distinct morphologies. The spectroscopic features such as peak prominence and broadness can be further used to search for synthetic conditions with higher yield and better monodispersity.

**Fig. 1.**
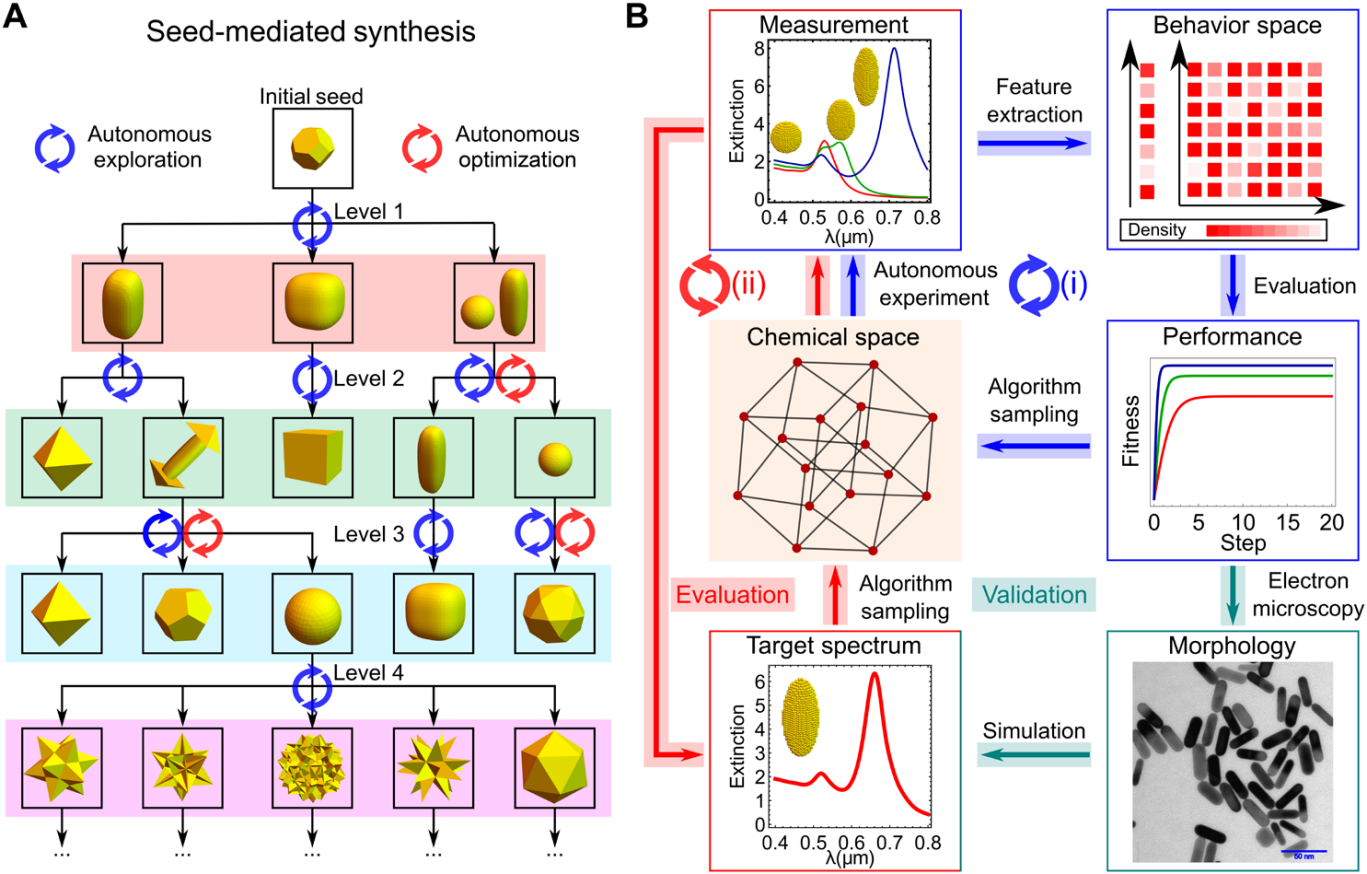
The closed-loop approach toward exploration and optimization in the seed-mediated synthesis of nanoparticles. (**A**) A pictorial representation of AuNPs from hierarchically linked chemical synthetic spaces in the seed-mediated synthesis. (**B**) Closed-loop approach for exploration [(i), blue cycle)] and optimization [(ii), red cycle], respectively. In the exploration, the UV-Vis features of samples are extracted to evaluate their behavior and performance, respectively. New experiments are designed to increase the spectral diversity and performance of samples. After exploration, TEM is used to reveal the morphologies of the high-performance samples, which offers a target spectrum for optimization through an extinction spectrum simulation engine. In the optimization, the UV-Vis spectra of samples are compared to a target spectrum and new experiments are designed to search multiple nanostructures with high spectral similarity to it.

Here, we conceptualized and developed a system for the autonomous intelligent exploration, discovery, and optimization of nanomaterials (AI-EDISON), which aims for both discovery and reproducible multistep synthesis of novel nanomaterials, with their unique digital signatures derived from physical properties and synthetic procedures (*47*). The experimental architecture performs parallel synthesis of nanomaterials together with real-time spectroscopic characterization and is assisted by ML algorithms and an extinction spectrum simulation engine. AI-EDISON uses state-of-the-art quality-diversity algorithms to explore high-dimensional combinatorial synthetic space to perform open-ended exploration and then conducts targeted optimization to search optimal synthetic conditions for nanomaterials with finely tuned optical properties. It can be further used to perform multistep synthesis of any desired nanoparticles it has found with a resource-efficient directed graph strategy coupled with real-time characterization. Using the directed graph approach, the complete multistep nanoparticle synthesis can be efficiently represented as a robust digital procedure, avoiding irreproducibility because of operation errors. With AI-EDISON, we investigated three chemical synthetic spaces connected by the seed-mediated synthesis of gold nanoparticles (AuNPs), where nanoparticles synthesized from the lower-level space were used as seeds in the higher-level space. By using UV-Vis spectroscopy as a primary characterization technique, we started with the hypothesis that increasing the diversity in the spectra could lead to the efficient exploration of the chemical space with distinct nanostructures. After exploration, a simulation engine was used to create the targets to further optimize the optical properties of AuNPs. These linked chemical spaces initialized from a single physical seed with intermediate exploration and optimization steps at various levels are represented in Fig. 1A.

The overall closed-loop algorithmic scheme used for the discovery of nanomaterials consists of two different modes: exploration and optimization (Fig. 1B). For each complete closed loop, AI-EDISON performs three different steps corresponding to nanoparticle synthesis, UV-Vis characterization and cleaning, and designing new experiments using ML algorithms, respectively. In the exploration mode, the structural diversity of the nanoparticles is achieved by searching for diversity in the behavior space. This behavior diversity is derived from the features observed in the UV-Vis spectra, such as peak number and position. The fitness, which is a numerical indication of the sample’s performance, is evaluated on the basis of peak prominence and broadness that correlate with the yield and monodispersity of the nanoparticles. A new batch of experiments is generated from previous synthetic conditions leading to higher-performance samples and diversified features. The process including the three steps iterates until the exploration is completed. After the exploration, TEM is used as a secondary characterization technique to check the morphologies of nanoparticles that have high-performance UV-Vis features. In the optimization mode, a target spectrum is defined by the extinction spectrum simulation of the nanoparticle with the shape derived from electron micrographs. This strategy with the extinction spectrum simulation extends the optimization targets to nanostructures with features that are not directly available in the exploration. Because of the lack of one-to-one mapping between UV-Vis spectra and nanostructures, various morphologies could lead to similar spectra to the target. Hence, the algorithm considers similarity to the target spectrum and the sampling density in the synthetic space to find multiple optimal conditions as solutions to the optimization problem.

## RESULTS

### AI-EDISON: Autonomous nanomaterials synthesis robot and characterization

**Fig. 2.**
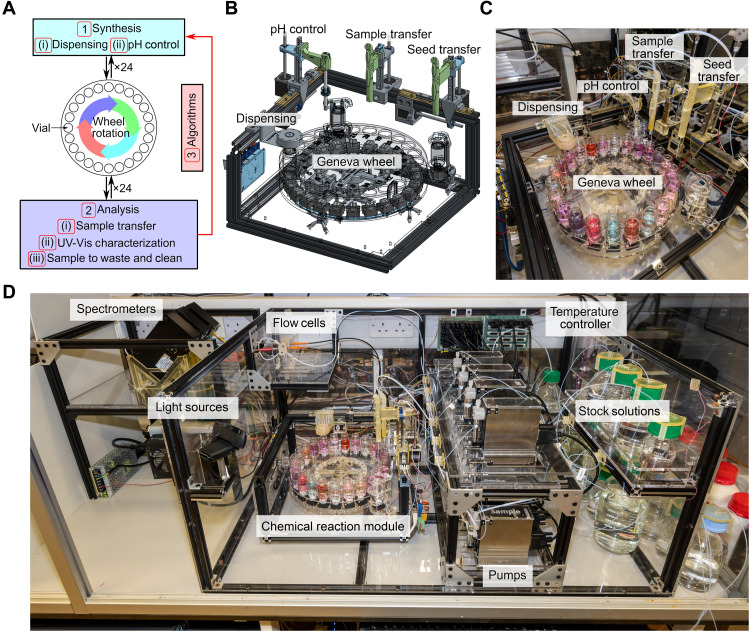
The autonomous nanomaterials discovery platform. (**A**) Workflow of the closed loop including synthesis, analysis, and design of new experiments by the algorithms. Three steps (1) to (3) are required to establish the closed loop, while the robotic operations included in the steps are labeled from (i) to (iii). (**B** and **C**) CAD design and the experimental setup of the chemical reaction module driven by the Geneva wheel with units for liquid dispensing, pH control, and solution transfer. (**D**) Overall setup of the autonomous platform with temperature controller, stock solutions, pumps, chemical reaction module, flow cells, light sources, and spectrometers.

The core robotic hardware consists of a chemical reaction module capable of performing parallel synthesis up to 24 reactors (*22*). The modular architecture uses the rotation of the Geneva wheel, which is synchronized with both parallel/sequential liquid dispensing and stirring of reagents to conduct the synthesis efficiently. Using a combination of high-precision syringe pumps, the control system performs liquid handling, mixing, cleaning, dynamic pH control, sample extraction/transfer, and in-line spectroscopic analysis. Except for spectrometers and light sources, the chemical reaction module together with stock solutions is contained in a temperature-controlled box for the fine-tuning of the reaction conditions to ensure reproducibility. The module is equipped with a seed extraction system for sample storage to run new reactions from the previously synthesized nanoparticles. For the discovery of AuNPs, the closed loop incorporates three steps including (1) parallel seed-mediated synthesis for a batch of reaction conditions suggested by algorithms that requires liquid dispensing and dynamic pH control, (2) spectroscopic analysis of the products together with cleaning steps to prepare for the next synthesis, and (3) data analysis involving feature extraction to generate new reaction conditions using ML algorithms. The complete iteration cycle, chemical reaction module, and overall experimental platform are shown in Fig. 2. Full details of the platform design, construction, and operations can be found in section S1 and movie S1.

### AI-EDISON: Quality-diversity algorithms for nanomaterials discovery

The exploration and optimization strategies are used to discover different nanostructures. They are based on the MAP-Elites and global search with local sparseness algorithms, respectively (section S2.1). In the exploration, AI-EDISON aims to facilitate the diversity in the behavior space, which is derived from the UV-Vis spectra of the nanoparticles. Inspired by the MAP-Elites algorithm (*37*), the complete behavior space is discretized into finite intervals called classes. Each sampling point corresponding to an experiment is classified, and a predefined fitness function is evaluated. The sampling points with the highest fitness in each class are defined as elites, which are then used as the parents to generate new sampling points via mutation, crossover, and random sampling that are commonly used in evolutionary algorithms (fig. S12).

In the context of our exploration, the sampling points represent the synthetic conditions, and the spectral wavelength range (400 to 950 nm) is discretized into multiple intervals. To increase the diversity of the UV-Vis spectra, a set of fitness functions are defined to facilitate relative prominences of spectral signals, e.g., to lead to spectra with a single dominant peak or two prominent peaks. By combining the intervals where dominant UV-Vis peaks are located and together with the selected fitness function, the sampling points are classified. As a final step, the fitness functions of the sampling points are evaluated to select the highest performance sample from each class. The selected samples will form the elite set that can be used as the parents to create the new synthetic conditions that will be further evaluated. The emergence of samples with high fitness values in various classes enables the search for nanoparticles with both diversified and optimal morphologies. This complete process iterates until the exploration is finished (see more details in section S2.1.2).

In the optimization mode, AI-EDISON searches synthetic conditions to produce samples toward a predefined target spectrum. The target spectrum can be the available spectrum from literature or the simulated spectrum of an estimated nanostructure from electron micrographs. The latter strategy uses the structural information from exploration and offers more practical targets. Because of the lack of a unique linkage between the morphology and UV-Vis spectrum, multiple nanostructures sharing similar spectral features can be fabricated in the same synthetic space with varied conditions. AI-EDISON searches multiple synthetic conditions by considering the similarity metric that quantifies the difference between sample and target spectra, together with the local sparseness of sampling points in the synthetic space. The local sparseness indicates the local sampling density and is calculated by estimating the average Euclidean distance between the sampling point and its *K*-nearest neighbors. To enable the global search, the fitness function for a sampling point is defined by a linear combination of the similarity metric and local sparseness. The top *N* sampling points with the highest fitness are selected as parents, and new synthetic conditions are generated via mutation, crossover, and random sampling (see more details in section S2.1.3).

### In silico evaluation of the performance of exploration and optimization algorithms

**Fig. 3.**
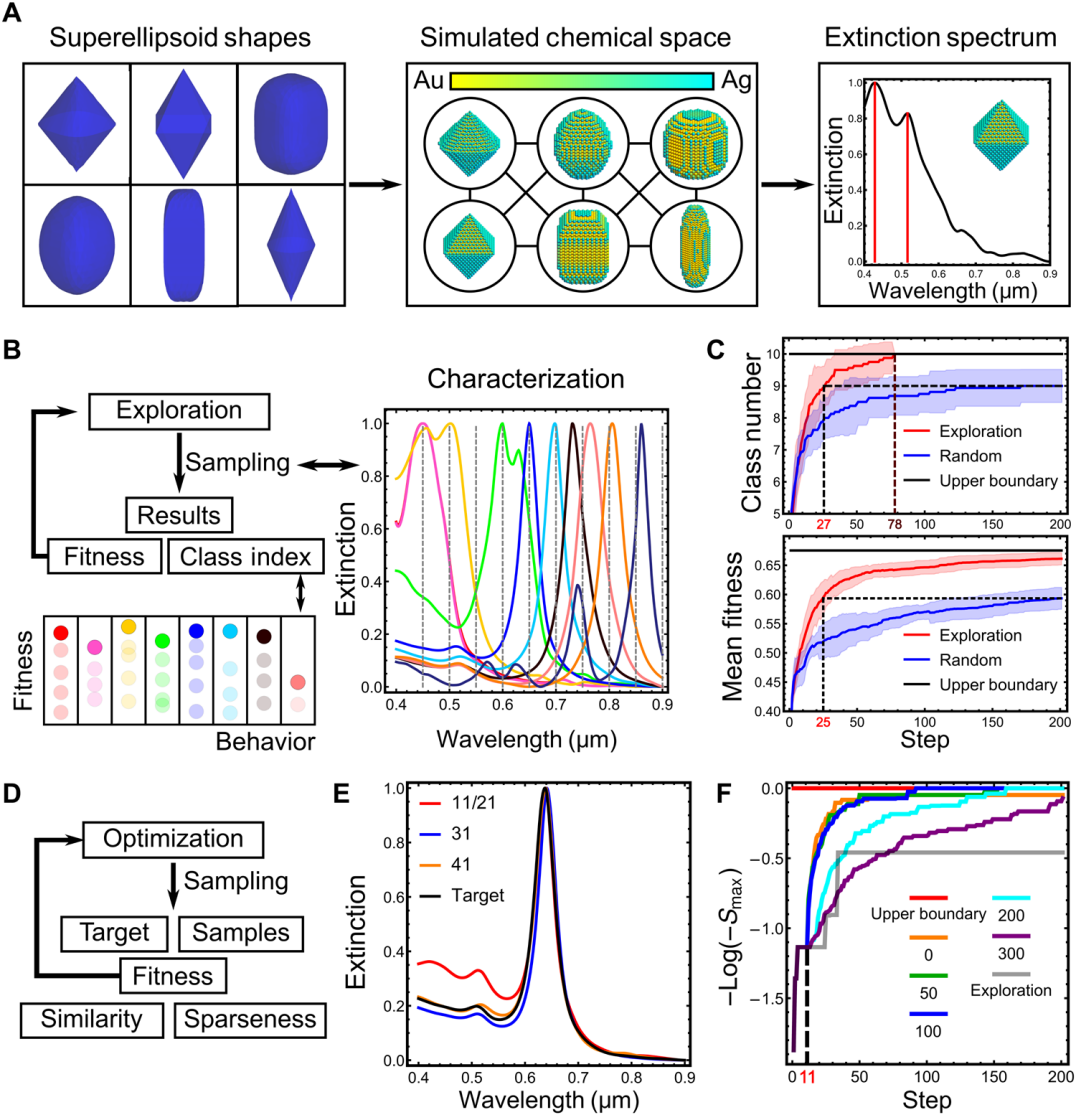
Exploration and optimization in the simulated chemical space. (**A**) Simulated chemical space with various shapes, Au/Ag compositions, and shape yields, with their corresponding simulated UV-Vis spectra through discrete-dipole approximation. (**B**) Investigation of the chemical space with the exploration algorithm from AI-EDISON. The class index (from 1 to 10 with an additional class of 0 for nonfeatured samples) is assigned depending on the peak position. The fitness values of the samples are evaluated to update the parent set iteratively. (**C**) Comparison of the performance in both discovering samples of previously unknown classes (top) and increasing the fitness of the highest performance samples within various classes (bottom) between the exploration algorithm from AI-EDISON and random search with 16 repeats. After 200 steps, random search still cannot find samples of all the classes with a standard deviation (SD) of 0.5 among the repeats. The exploration algorithm outperforms the final result from random search after 27 steps and can find samples belonging to all classes after 78 steps. (**D**) Investigation of the chemical space with the optimization algorithm from AI-EDISON after exploration. (**E**) UV-Vis spectra of solutions with the highest similarity after the exploration of 11, 21, 31, and 41 steps, indicating the unsuccessful search toward a target purely by exploration. (**F**) Increase of the similarity metric during the optimization with varied importance of the local sparseness, along with exploration strategy. The linear coefficient (*k*) is changed from 0 to 300.

The exploration and optimization algorithms in AI-EDISON were benchmarked in a simulated chemical space with calculated spectral properties. The simulated space contains parameters describing the three-dimensional (3D) solid mimicking the nanoparticle shape, metal composition (Au/Ag), and yield (Fig. 3A). The input chemical space comprises five parameters (*v*_1_, *v*_2_, *v*_3_, *v*_4_, *v*_5_), where (*v*_1_, *v*_2_, *v*_3_) describes the nanoparticle shape using superellipsoid as the shape descriptor, *v*_4_ represents relative silver concentration, and *v*_5_ describes the nanoparticle yield in the presence of octahedral Au-Ag bimetallic NPs as by-products (see sections S2.3 and S2.4). The observation space of UV-Vis spectra was generated through the extinction spectrum simulation. The scheme for the exploration to facilitate the UV-Vis diversity and to optimize spectral features is available in Fig. 3B. The exploration algorithm demonstrates a very efficient discovery of diverse and high-performance samples in the simulated chemical space outperforming random search, and the average fitness of the highest performance samples from different classes eventually reaches 98% to the estimated maximum (Fig. 3C). Full details for the benchmark of the exploration are available in section S2.5.

The optimization strategy toward a target spectrum continued on the basis of the dataset gathered during exploration. Considering the nonuniqueness of the UV-Vis spectra to a specific morphology of nanostructure, the optimization is set up to find multiple sampling points corresponding to global and local maxima in the similarity landscape (Fig. 3D). Without setting an explicit target, it is unlikely that the exploration algorithm can accidentally find a solution with similar UV-Vis to the target, as shown in Fig. 3 (E and F). Thus, a fitness function is crucial to guide the directive optimization. The fitness (*F*) is defined by the linear combination of the similarity metric (α) and the local sparseness (β) by *F* = α + *k*β, where the linear coefficient *k* tunes the weights between enabling similarity to the target and sampling in the less explored region. To evaluate the performance of the optimization algorithm, the input space was first explored for 11 steps, then a target spectrum was assigned, and optimization was performed by setting different values of the linear coefficient *k*. The selection of the optimal *k* value is crucial for the efficiency of the optimization, which is shown in Fig. 3F. With the lower *k* values of 0 and 50, the optimization strategy attempted to find the global maximum with a high probability of getting trapped in a local maximum. With higher values such as *k* = 100, the optimization algorithm found the global maximum efficiently. However, further increasing *k* ≥ 200 led to exploration in the sparsely sampled space with a less preference for similarity, causing a decrease in optimization efficiency. Furthermore, the local sparseness term encourages the search in less sampled input space and helps to target multiple local maxima with respect to the similarity metric (fig. S27). The full details for the benchmark of the optimization algorithm are available in section S2.6.

### Exploration of chemical spaces of AuNPs using AI-EDISON

With AI-EDISON, three hierarchically linked chemical spaces with potential 10^23^ experiments (see section S3.5) were explored with varied synthetic conditions, where diversified morphological features emerged in the seed-mediated synthesis. A single exploration step consists of 23 reactions unless explicitly mentioned, each of which has experimental constraints such as constant total volume, temperature, and synthesis interval. An additional well-defined experiment was performed at each step to verify the stability and precision of the control hardware and characterization. Because of different observations in UV-Vis spectra in the three chemical spaces, the fitness functions and definition of classes were modified accordingly.

### Chemical space 1: Seed-mediated synthesis on cuboctahedron single crystals

In the first chemical space, exploration of diverse nanostructures was performed using a single-crystal cuboctahedron seed (ca. 2 nm), which was prepared from the fast reduction of gold (III) chloride trihydrate (HAuCl_4_) using NaBH_4_ in the presence of hexadecyltrimethylammonium bromide (CTAB) (*48*). The parameters for the first chemical space were defined by the volumes of CTAB, silver nitrate (AgNO_3_), HAuCl_4_, and ascorbic acid while keeping the volume of seed solution fixed at 0.5 ml. A total volume of 12 ml was introduced as an additional constraint by adding water if required. At each exploration step, the sampling points were distributed to single- or multiple-peak features based on the analysis of their spectra. With peak number, peak positions, and the selection of fitness functions, they were classified into respective classes. On the basis of the exploration criteria toward a single dominant and two prominent peaks, the set of fitness functions leading to different classes is described in section S3.

**Fig. 4.**
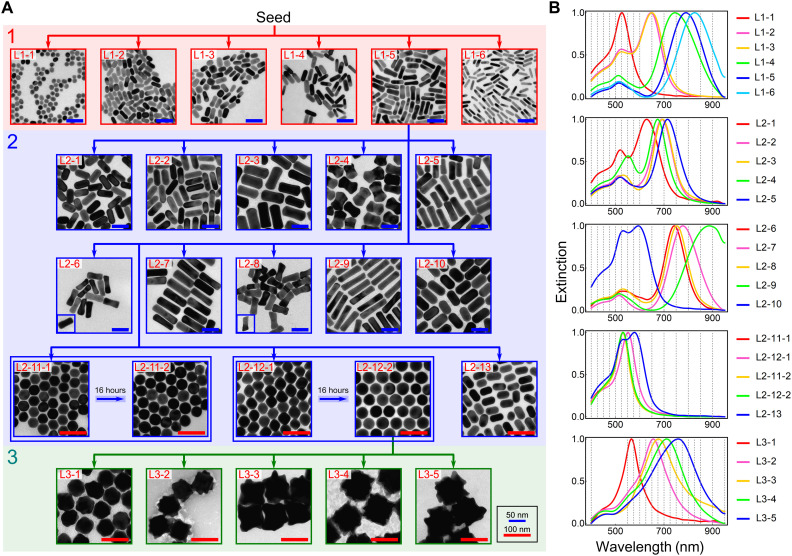
The exploration to discover uniquely shaped AuNPs in three linked chemical spaces. (**A**) Electron micrographs of the obtained AuNPs and their synthetic trajectories in the seed-mediated synthesis. L1-5 and L2-12-2 were used as the seeds after exploration. The three chemical spaces are indicated by the red, blue, and green background. Scale bars, 50 nm (blue) and 100 nm (red), respectively. (**B**) UV-Vis spectra corresponding to the AuNPs from three chemical spaces. It should be noted that L2-11-1, L2-11-2, and L2-12-2 showed very similar UV-Vis spectra. The gray dashed lines indicate the discrete subregions to facilitate the UV-Vis diversity during exploration.

Starting with random sampling at the first step, the observed highest performance samples within various classes with both single-peak and multiple-peak features were used as the parents to generate new sampling points in synthetic space. The samples of classes that did not exist after the initial random sampling can be generated by mutation and crossover from the parent set and random sampling. The exploration ran for 10 steps with a total of 230 experiments. During the exploration, the crossover and mutation operations updated the parent set 42 times. Only four events (<10%) were observed, where a new elite with higher performance or belonging to a previously nonexistent class was generated via crossover or mutation from previous parents with different peak numbers to it, indicating relatively weak interactions between single- and multiple-peak features. At this stage, electron micrographs obtained from the secondary characterization of TEM confirmed the presence of Au nanospheres and nanorods. Hence, as an additional phase, we extended the exploration further by orienting toward constrained exploration and exploitation using a new fitness function to improve the performance observed in diversified samples. The new fitness function was selected to primarily increase the contribution of a single peak and lower the secondary peak, where the absorption from the by-products was considered explicitly. Six additional steps were performed with different coefficients in the fitness function, which were used among steps of 11 to 14 and 15 and 16. A total of six distinct AuNPs with synthetic conditions leading to high yield and monodispersity were found (Fig. 4, L1-1 to L1-6 with additional images available in figs. S38 to S43). The exploration in this chemical space found symmetry breaking as a key phenomenon toward the emergence of observed nanospheres and nanorods.

### Chemical space 2: Overgrowth of Au nanorods

In the second chemical space, the sample of Au nanorods (L1-5) found from the previous chemical space was selected as seed because of its relatively high aspect ratio and the presence of the concave features on the surface. Hydroquinone was used as the reductant, and the pH of the growth solution was introduced as an additional variable. Because of the relatively weak interactions between single- and multiple-peak features as observed during the exploration in the first chemical space, these features were explored sequentially. Starting with multiple-peak features, the exploration was performed toward a single dominant and two comparable peaks by using peak position and relative prominences similarly to previous chemical space. The exploration ran for 10 steps (a total of 230 reactions) excluding single-peak outcomes, and three classes of nanorods were found: (i) rods with spherical caps, (ii) rods with rectangular caps, and (iii) irregular rods resembling dog bones. The total of 10 found nanorods of different sizes and belonging to three classes are shown in Fig. 4 L2-1 to L2-10 with additional images available in figs. S48 to S57.

To increase further diversity in single-peak feature, the exploration of single-peak feature ran for 10 steps initialized with the data from the multiple-peak exploration. New single-peak classes were defined by discretizing the wavelength of 400 to 600 nm with 25-nm interval and 600 to 950 nm with 50-nm interval. Previously, there were four classes found from the initial dataset and the total number of discovered classes increased to 10 at the end of the exploration. With the single-peak feature, the synthetic conditions for three additional morphologies of (i) spherical polyhedra, (ii) bicones, and (iii) rods with low aspect ratios were found. The spherical polyhedra and bicones were transformed into highly monodispersed spheres after being aged for 16 hours. See Fig. 4 L2-11 to L2-13 for these nanostructures and the respective UV-Vis spectra, with additional TEM images available in figs. S58 to S62. The discovery of various anisotropic and symmetric nanoparticles seeded from smaller nanorods demonstrates the existence of different overgrowth phenomena leading to the emergence of diverse nanostructures.

### Chemical space 3: Overgrowth of Au nanospheres

In the third chemical space, the sample of spherical nanoparticles (L2-12-2) was selected as the seed because of their high monodispersity and smooth surface. The 5D input chemical space was defined by volumes of hexadecyltrimethylammonium chloride (CTAC), AgNO_3_, HAuCl_4_, ascorbic acid, and hydrochloric acid (HCl). The volume of seed solution used was fixed to 0.5 ml, and the total volume was constrained to 12 ml. The exploration algorithm ran for 10 steps (231 experiments with 24 from the initial random sampling) focusing only on the single-peak feature while sampling points leading to multiple peaks were discarded. The classes were defined by defining the region between 400 and 550 nm as a single class and discretizing 550 to 800 nm with 25-nm interval and 800 to 950 nm with 50-nm interval. The algorithm found 11 high-performance samples of different classes and found synthetic conditions leading to a series of nanostars with sizes ranging between 60 and 95 nm and various tip features (see Fig. 4 L3-1 to L3-5 and the corresponding UV-Vis spectra). Additional TEM images of these structures are available in section S3.4. The morphology of L3-1 comprises a ca. 60-nm core with tiny tips on the surface leading to lower peak absorbance (ca. 560 nm). The peak position redshifts with the increase in core size as evidenced by the absorbance peaks of UV-Vis spectra of nanostars. The algorithmic discovery of the existence of nanostars with variable core sizes and tip features with high yield and monodispersity occurred because of the presence of distinct peak absorbances in the UV-Vis spectra with optimal broadness.

The successful search of a variety of uniquely shaped AuNPs with high yield and monodispersity using AI-EDISON validates the initial hypothesis that the structural diversity of nanostructures can be achieved by increasing the diversity of the spectra. It also demonstrates that amplifying UV-Vis features like prominence or broadness can improve the yield and monodispersity of the synthesized nanostructures.

### Targeted optimization in the explored chemical space

The exploration algorithm found synthetic conditions of nanoparticles with distinct UV-Vis behaviors in a coarse-grained way, which can be limited by class intervals without a specific target. To efficiently search synthetic conditions toward finely tuned optical properties using the previously explored dataset, the optimization algorithm with specific targets should be used. Considering the nonuniqueness of UV-Vis spectra toward a single nanostructure, the fitness function is defined by a combination of local sparseness and similarity toward the target spectrum. The target spectrum can be defined either from a literature report or generated in silico after creating a 3D nanostructure derived from electron micrographs, which offers the more practical targets in the chemical space. To demonstrate efficient optimization in high-dimensional space with multiple possible solutions, two target spectra were generated in silico in the first and third chemical space (see section S4 for more details).

**Fig. 5.**
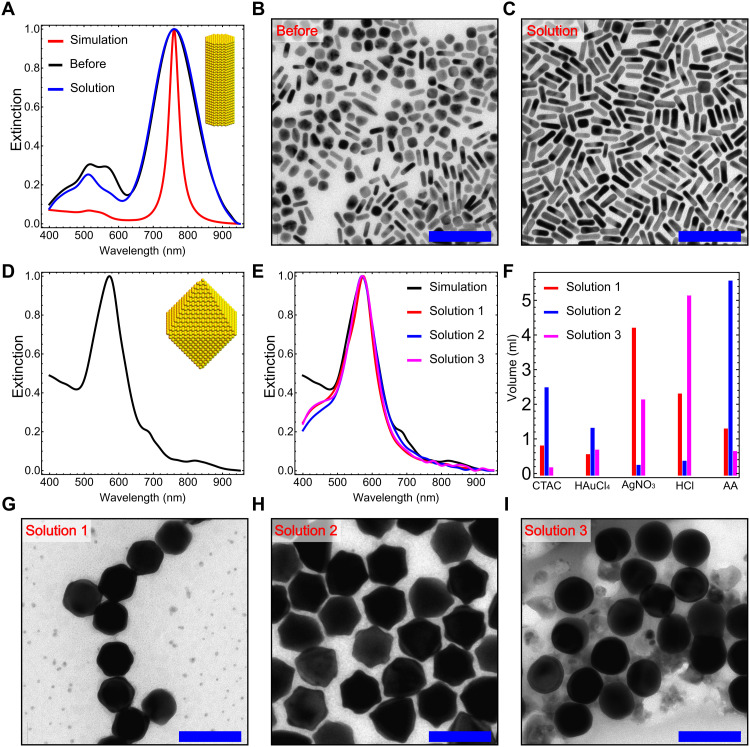
The optimization toward target spectra from the extinction spectrum simulation of AuNPs. (**A**) Comparison of the UV-Vis spectra from the simulation of target Au nanorods, the most similar sample before optimization, and the optimal solution after optimization. (**B** and **C**) Electron micrographs of the best sample before optimization with a yield of ca. 57% and the optimal solution after optimization with a yield of ca. 95%. (**D**) Target UV-Vis spectrum derived from Au octahedra. (**E**) Comparison of the UV-Vis spectra of the simulated Au octahedral target and three solutions after optimization. (**F**) Comparison of the synthetic conditions of the three solutions after the optimization. AA, ascorbic acid. (**G** to **I**) Electron micrographs of solutions 1 to 3, which corresponds to octahedral, concave octahedral, and smooth polyhedral nanoparticles. Scale bars, 100 nm (B and C and G to I).

In the first case, the existence of Au nanorods was observed in chemical space 1 during exploration. The target spectrum was simulated using a cylindrical Au nanorod with a diameter and length of 11 and 33 nm, respectively, to precisely control the longitudinal peak. After the exploration, although the sample with the highest similarity shared the same longitudinal peak position, the presence of a shoulder peak around 570 nm indicated the existence of by-products. The optimization ran for five steps (115 reactions), and two synthetic conditions leading to a smaller by-product peak were obtained (fig. S97). They showed similar morphological features of nanorods because of the relatively unique transverse and longitudinal UV-Vis peaks (figs. S99 and S100). Figure 5A shows the UV-Vis spectra of the target, the best solution before optimization, and one of the solutions with the higher nanorod yield after optimization. The corresponding electron micrographs are shown in Fig. 5 (B and C), indicating the increase of shape yield in the solution from 57 to 95%.

In the second case, during the exploration in the third chemical space, although only a small portion of Au octahedra was observed (fig. S66), the structural features of the sample, as well as L3-1, suggested a high propensity toward the emergence of octahedral nanoparticles. Hence, an octahedral Au nanostructure with an edge length of ca. 57 nm was created, and the target spectrum was simulated (Fig. 5D) accordingly. The spectrum has an intrinsically broad peak because of its geometry and is independent of the size distribution. The presence of this intrinsic broadness decreases the uniqueness of UV-Vis toward a specific nanostructure. A variety of nanostructures including spheres, octahedra, other polyhedral shapes of various sizes, and their mixtures can share similar UV-Vis features. Hence, it is necessary for the optimization strategy to search for multiple solutions well separated in a high-dimensional chemical synthetic space, which are likely to correspond to different structures. The synthetic space for the optimization was selected similar to chemical space 3 during exploration except for the concentration of HAuCl_4_, which was halved. This reduction was based on the observation that the top five sampling points in the combinatorial space with the highest similarity to the target after the exploration had a small volume of HAuCl_4_ (<1.00 ml). The optimization algorithm ran for five steps (115 reactions), and multiple solutions with high spectral similarities but distinct synthetic conditions to the target were found (Fig. 5, E and F). These synthetic conditions corresponded to local maxima in the fitness landscape and resulted in different nanostructures including octahedral, concave octahedral, and smooth polyhedral nanoparticles (Fig. 5, G to I), which demonstrated the successful search of distinct nanostructures with similar UV-Vis spectra to the target through the optimization strategy.

### Multistep nanoparticle synthesis using AI-EDISON architecture

**Fig. 6.**
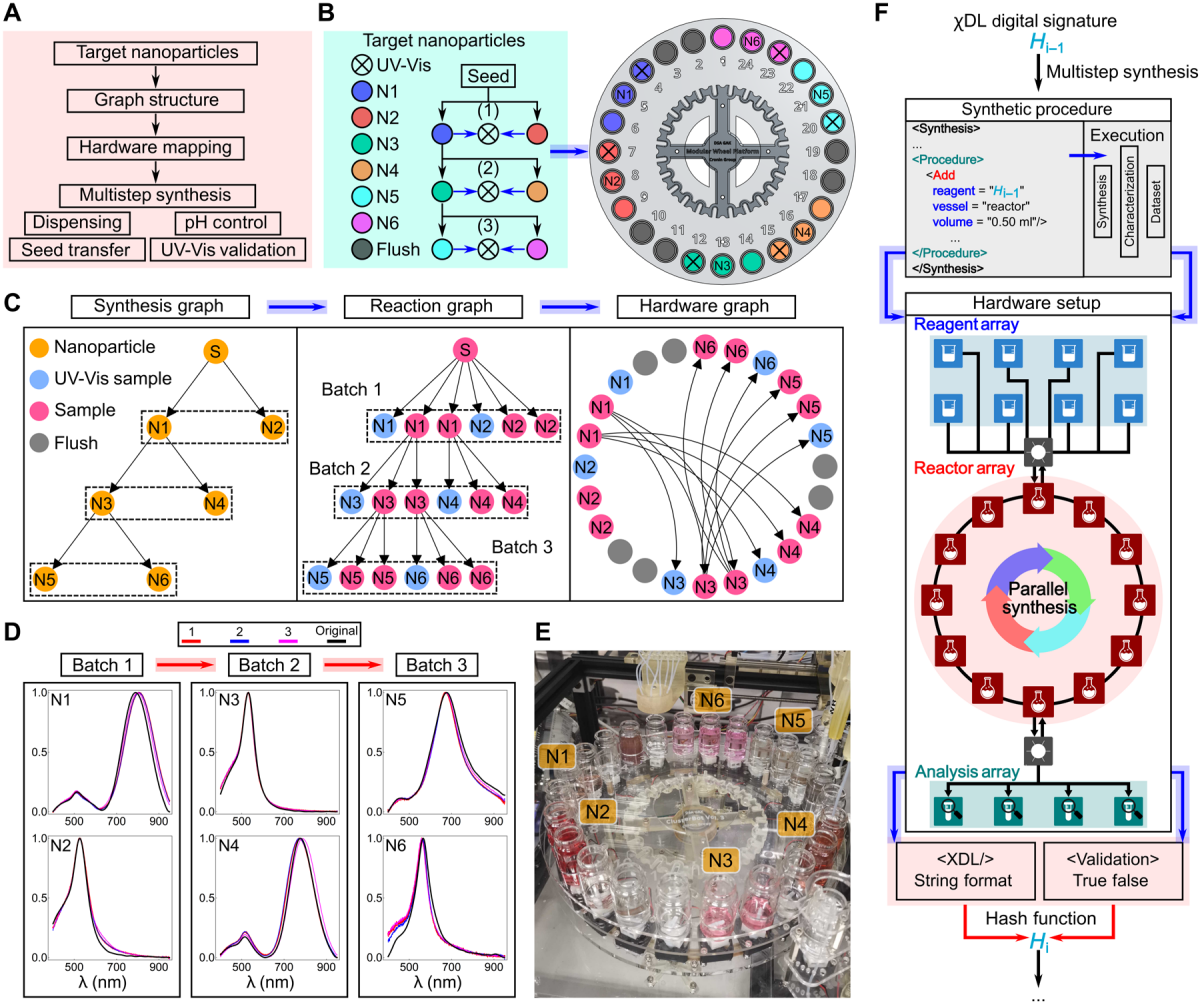
The fully autonomous multistep synthesis with directed graph structure. (**A**) Workflow of the autonomous multistep synthesis of AuNPs. A graph structure is used to allocate the available hardware resources to samples on the chemical reaction module and design the operations to be executed for the synthesis. (**B**) Six target AuNPs, their hierarchical relations, and distribution on the chemical reaction module. They are labeled from N1-N6, which correspond to L1-5, L1-1, L2-12-2, L2-7, L3-3, L3-1, respectively. (**C**) Synthesis, reaction, and hardware graph for the multistep synthesis of the six AuNPs. The number of repeated samples is estimated on the basis of the volume required for seeding, characterization, and desired final volume. (**D**) UV-Vis spectra of samples from three repeats and the original sample. The most prominent peak position is 799.6 ± 6.5, 525.9 ± 0.9, 530.0 ± 1.2, 777.0 ± 6.9, 673.4 ± 2.6, and 561.0 ± 3.4 nm from N1 to N6, respectively. (**E**) Actual distribution of the samples on the chemical reaction module. (**F**) Procedure to generate the unique digital signatures of nanoparticles through chemical description language (χDL) and product validation in the multistep synthesis. The combined string of χDL and the validation result were encoded with utf-8 and further converted to the unique digital signature using the hash function of SHA-256.

The modularity of the platform allows conducting parallel multistep synthesis using a generic directed graph structure together with required characterization at each step to ensure synthesis reproducibility (Fig. 6A). This approach requires three graphs: synthesis, reaction, and hardware, which was illustrated by synthesizing six uniquely shaped AuNPs found from the previous exploration (Fig. 6B). As shown in Fig. 6C, the synthesis graph represents the multistep synthetic procedure of nanoparticles, where each node represents a unique nanoparticle and each directed edge implies the hierarchical relation between nanoparticles. To map the synthesis graph to the robotic platform, a reaction graph that constitutes the required robotic operations is generated. Furthermore, a hardware graph is derived from the reaction graph to allocate the available resources of the chemical reaction module. Each node in the reaction and hardware graph represents an actual sample to be prepared, and the directed edges represent the transfer steps required for seeding from one sample to another.

The parallel synthesis of all six AuNPs was repeated three times to demonstrate reproducibility. The observed standard deviation (SD) in the UV-Vis spectra is 3 to 4 nm with a maximum deviation around 7 nm, where all the peak positions of the repeats are within two SDs from the mean values (Fig. 6, D and E). Although unique identifiers of chemicals like CAS number exist, the equivalent unique signatures for nanomaterials corresponding to their distinctive synthetic protocols are still lacking. A promising approach to represent unique identifiers for nanoparticles could be generated from the digital representations of synthetic procedures. The universal chemical description language χDL (*47*) could be used to create the unique digital signatures for nanomaterials. Hardware-independent synthetic procedures in the standard format represented by χDL were used to describe the reliable synthesis of nanoparticles with expected properties. The validation of the synthesized nanoparticles can be performed by various techniques, and UV-Vis was selected here because of the plasmonic effect of AuNPs. Later, the unique digital signatures of AuNPs were created from the combination of their synthetic procedures and validations of the products through a hash function (Fig. 6F). See section S5 and movie S2 for a complete description of the implementation.

## DISCUSSION

In summary, we have developed a unified architecture AI-EDISON that includes a fully autonomous closed-loop synthesis robot that incorporates state-of-the-art ML algorithms and an extinction spectrum simulation engine. Using quality-diversity algorithms, we explored three linked chemical spaces and found AuNPs including spheres, rods, spherical polyhedra, bicones, and stars with diversified features. Although UV-Vis cannot offer detailed structural information of nanoparticles like crystallographic phases or electron density distributions compared to electron microscopy, it was sufficient to target distinct plasmonic nanostructures. By exploration with UV-Vis as a primary characterization tool, we proved our hypothesis that structural diversity can be achieved by increasing the spectral diversity and that demonstrated nanoparticles with high yield and monodispersity can be obtained by amplifying specific spectral features. After coarse-grained exploration of high-dimensional chemical space, the system performed optimization for finely tuned optical properties with a target spectrum generated using electron microscopy and extinction spectrum simulation. The optimization strategy found multiple synthetic conditions that led to distinct nanostructures with high yield, monodispersity, and similar UV-Vis features, which were not directly available from the exploration. Using the modularity and capacity to perform parallel operations and synthesis on AI-EDISON, we demonstrated a highly efficient and fully digitized approach toward the complex multistep synthesis of nanomaterials with their unique digital signatures derived from χDL.

## MATERIALS AND METHODS

### Chemical reagents

All solutions were prepared with type I water. CTAB (>99%), CTAC (>99%), and hydroquinone (99.5%) were purchased from Acros Organics. HAuCl_4_ (99.9%), ascorbic acid (99.9%), and AgNO_3_ (99.9999%) were purchased from Sigma-Aldrich. HCl (37%) and sodium hydroxide (98 to 100.5%) were purchased from Honeywell Fluka. Orion pH buffer solutions (4.0, 7.0, and 10.0) were purchased from Thermo Fisher Scientific. All the reagents were used as received. The procedure to prepare the stock solutions is available in sections S3 to S5.

### Platform

The platform was constructed in-house from a range of 3D printed, laser-cut, and commercially available components. A full bill of materials and assembly instructions can be viewed in section S1. The software control of the platform for basic operations was written in Python 3. Full details of implementation can be found at https://github.com/croningp/NanoDiscovery.

### The extinction spectrum simulation engine (PyDScat-GPU)

The software was used for graphics processing unit (GPU) accelerated extinction spectrum simulation of metallic nanoparticles based on the discrete-dipole approximation method and written in Python 3 using TensorFlow 2. The full details are available in section S2 and https://github.com/croningp/NanoDiscovery.

### Algorithms and data analysis

The details of benchmarking the algorithms in the simulated chemical spaces are described in section S2, where the codes were written in Mathematica 12.0. The details of the spectral data analysis and the algorithms in the experimental exploration and optimization of the three chemical spaces are available in sections S3 and S4. Extra TEM images for the nanoparticles discussed during exploration and optimization were shown in sections S3 and S4, respectively. The codes for the experimental implementation of both algorithms were written in Python 3 (https://github.com/croningp/NanoDiscovery) and connected to the control software of the platform to establish a closed loop.

### Directed graph structure and hash representation

The code to generate directed graphs was written in Python 3 using NetworkX. The control software can read the directed graph and execute the operations defined in the graph. The digital signatures of the nanoparticles were generated from the string format of the chemical synthetic procedures written in χDL. Full details are available in section S5 and https://github.com/croningp/NanoDiscovery.

## Supplementary Materials

### This PDF file includes:

Supplementary Materials Figs. S1 to S115 Tables S1 to S22 References

## References

[1] L. Cheng, C. Wang, L. Feng, K. Yang, Z. Liu, Functional nanomaterials for phototherapies of cancer. Chem. Rev. 114, 10869–10939 (2014). 25260098 10.1021/cr400532z

[2] B. Radisavljevic, A. Radenovic, J. Brivio, V. Giacometti, A. Kis, Single-layer MoS_2_ transistors. Nat. Nanotechnol. 6, 147–150 (2011). 21278752 10.1038/nnano.2010.279

[3] S. Cao, F. F. Tao, Y. Tang, Y. Li, J. Yu, Size- and shape-dependent catalytic performances of oxidation and reduction reactions on nanocatalysts. Chem. Soc. Rev. 45, 4747–4765 (2016). 27276189 10.1039/c6cs00094k

[4] T. B. Hoang, G. M. Akselrod, M. H. Mikkelsen, Ultrafast room-temperature single photon emission from quantum dots coupled to plasmonic nanocavities. Nano Lett. 16, 270–275 (2016). 26606001 10.1021/acs.nanolett.5b03724

[5] I. O. Sosa, C. Noguez, R. G. Barrera, Optical properties of metal nanoparticles with arbitrary shapes. J. Phys. Chem. B 107, 6269–6275 (2003).

[6] Y. H. Yu, C. C. M. Ma, C. C. Teng, Y. L. Huang, S. H. Lee, I. Wang, M. H. Wei, Electrical, morphological, and electromagnetic interference shielding properties of silver nanowires and nanoparticles conductive composites. Mater. Chem. Phys. 136, 334–340 (2012).

[7] L. Yang, Z. Wang, L. Ma, A. Li, J. Xin, R. Wei, H. Lin, R. Wang, Z. Chen, J. Gao, The roles of morphology on the relaxation rates of magnetic nanoparticles. ACS Nano 12, 4605–4614 (2018). 29672022 10.1021/acsnano.8b01048

[8] S. E. Lohse, C. J. Murphy, The quest for shape control: A history of gold nanorod synthesis. Chem. Mater. 25, 1250–1261 (2013).

[9] W. Shepherd, M. Wilms, J. Van Embden, E. D. Gaspera, Accurate control of stoichiometry and doping in barium stannate perovskite oxide nanoparticles. Chem. Commun. 55, 11880–11883 (2019). 10.1039/c9cc04838c 31528881

[10] L. Scarabelli, A. Sánchez-Iglesias, J. Pérez-Juste, L. M. Liz-Marzán, A “tips and tricks” practical guide to the synthesis of gold nanorods. J. Phys. Chem. Lett. 6, 4270–4279 (2015). 26538043 10.1021/acs.jpclett.5b02123

[11] A. Ali, H. Zafar, M. Zia, I. U. Haq, A. R. Phull, J. S. Ali, A. Hussain, Synthesis, characterization, applications, and challenges of iron oxide nanoparticles. Nanotechnol. Sci. Appl. 9, 49–67 (2016). 27578966 10.2147/NSA.S99986 PMC4998023

[12] E. M. Marlett, Electrochemical synthesis of organometallics. Ann. N. Y. Acad. Sci. 125, 12–24 (1965).

[13] K. Esumi, K. Matsuhisa, K. Torigoe, Preparation of rodlike gold particles by UV irradiation using cationic micelles as a template. Langmuir 11, 3285–3287 (1995).

[14] H. E. Lee, R. M. Kim, H. Y. Ahn, Y. Y. Lee, G. H. Byun, S. W. Im, J. Mun, J. Rho, K. T. Nam, Cysteine-encoded chirality evolution in plasmonic rhombic dodecahedral gold nanoparticles. Nat. Commun. 11, 1–10 (2020). 31937767 10.1038/s41467-019-14117-x PMC6959252

[15] B. Nikoobakht, M. A. El-Sayed, Preparation and growth mechanism of gold nanorods (NRs) using seed-mediated growth method. Chem. Mater. 15, 1957–1962 (2003).

[16] H. L. Wu, H. R. Tsai, Y. T. Hung, K. U. Lao, C. W. Liao, P. J. Chung, J. S. Huang, I. C. Chen, M. H. Huang, A comparative study of gold nanocubes, octahedra, and rhombic dodecahedra as highly sensitive SERS substrates. Inorg. Chem. 50, 8106–8111 (2011). 21797229 10.1021/ic200504n

[17] I. Ojea-Jiménez, N. G. Bastús, V. Puntes, Influence of the sequence of the reagents addition in the citrate-mediated synthesis of gold nanoparticles. J. Phys. Chem. C 115, 15752–15757 (2011).

[18] G. Mountrichas, S. Pispas, E. I. Kamitsos, Effect of temperature on the direct synthesis of gold nanoparticles mediated by poly(dimethylaminoethyl methacrylate) homopolymer. J. Phys. Chem. C 118, 22754–22759 (2014).

[19] R. Baber, L. Mazzei, N. T. K. Thanh, A. Gavriilidis, An engineering approach to synthesis of gold and silver nanoparticles by controlling hydrodynamics and mixing based on a coaxial flow reactor. Nanoscale 9, 14149–14161 (2017). 28905060 10.1039/c7nr04962e

[20] J. H. Lee, K. J. Gibson, G. Chen, Y. Weizmann, Bipyramid-templated synthesis of monodisperse anisotropic gold nanocrystals. Nat. Commun. 6, 7571 (2015). 26113393 10.1038/ncomms8571 PMC4491807

[21] S. Steiner, J. Wolf, S. Glatzel, A. Andreou, J. M. Granda, G. Keenan, T. Hinkley, G. Aragon-Camarasa, P. J. Kitson, D. Angelone, L. Cronin, Organic synthesis in a modular robotic system driven by a chemical programming language. Science 363, eaav2211 (2019). 30498165 10.1126/science.aav2211

[22] D. S. Salley, G. A. Keenan, D. L. Long, N. L. Bell, L. Cronin, A modular programmable inorganic cluster discovery robot for the discovery and synthesis of polyoxometalates. ACS Cent. Sci. 6, 1587–1593 (2020). 32999934 10.1021/acscentsci.0c00415 PMC7517417

[23] J. Li, S. G. Ballmer, E. P. Gillis, S. Fujii, M. J. Schmidt, A. M. E. Palazzolo, J. W. Lehmann, G. F. Morehouse, M. D. Burke, Synthesis of many different types of organic small molecules using one automated process. Science 347, 1221–1226 (2015). 25766227 10.1126/science.aaa5414 PMC4687482

[24] B. Burger, P. M. Maffettone, V. V. Gusev, C. M. Aitchison, Y. Bai, X. Wang, X. Li, B. M. Alston, B. Li, R. Clowes, N. Rankin, B. Harris, R. S. Sprick, A. I. Cooper, A mobile robotic chemist. Nature 583, 237–241 (2020). 32641813 10.1038/s41586-020-2442-2

[25] J. Chang, P. Nikolaev, J. Carpena-Núñez, R. Rao, K. Decker, A. E. Islam, J. Kim, M. A. Pitt, J. I. Myung, B. Maruyama, Efficient closed-loop maximization of carbon nanotube growth rate using bayesian optimization. Sci. Rep. 10, 1–9 (2020). 32493911 10.1038/s41598-020-64397-3 PMC7271124

[26] R. W. Epps, M. S. Bowen, A. A. Volk, K. Abdel-Latif, S. Han, K. G. Reyes, A. Amassian, M. Abolhasani, Artificial chemist: An autonomous quantum dot synthesis bot. Adv. Mater. 32, 1–9 (2020). 10.1002/adma.202001626 32495399

[27] S. Langner, F. Häse, J. D. Perea, T. Stubhan, J. Hauch, L. M. Roch, T. Heumueller, A. Aspuru-Guzik, C. J. Brabec, Beyond ternary OPV: High-throughput experimentation and self-driving laboratories optimize multicomponent systems. Adv. Mater. 32, e1907801 (2020). 32049386 10.1002/adma.201907801

[28] H. Tao, T. Wu, S. Kheiri, M. Aldeghi, A. Aspuru-Guzik, E. Kumacheva, Self-driving platform for metal nanoparticle synthesis: Combining microfluidics and machine learning. Adv. Funct. Mater. 2106725, 1–9 (2021).

[29] P. Raccuglia, K. C. Elbert, P. D. F. Adler, C. Falk, M. B. Wenny, A. Mollo, M. Zeller, S. A. Friedler, J. Schrier, A. J. Norquist, Machine-learning-assisted materials discovery using failed experiments. Nature 533, 73–76 (2016). 27147027 10.1038/nature17439

[30] A. O. Oliynyk, E. Antono, T. D. Sparks, L. Ghadbeigi, M. W. Gaultois, B. Meredig, A. Mar, High-throughput machine-learning-driven synthesis of full-heusler compounds. Chem. Mater. 28, 7324–7331 (2016).

[31] D. Salley, G. Keenan, J. Grizou, A. Sharma, S. Martín, L. Cronin, A nanomaterials discovery robot for the Darwinian evolution of shape programmable gold nanoparticles. Nat. Commun. 11, 1–7 (2020). 32488034 10.1038/s41467-020-16501-4 PMC7265452

[32] H. Tao, T. Wu, M. Aldeghi, T. C. Wu, A. Aspuru-Guzik, E. Kumacheva, Nanoparticle synthesis assisted by machine learning. Nat. Rev. Mater. 6, 701–716 (2021).

[33] J. Li, J. Li, R. Liu, Y. Tu, Y. Li, J. Cheng, T. He, X. Zhu, Autonomous discovery of optically active chiral inorganic perovskite nanocrystals through an intelligent cloud lab. Nat. Commun. 11, 1–10 (2020). 32341340 10.1038/s41467-020-15728-5 PMC7184584

[34] A. G. Kusne, H. Yu, C. Wu, H. Zhang, J. Hattrick-Simpers, B. DeCost, S. Sarker, C. Oses, C. Toher, S. Curtarolo, A. V. Davydov, R. Agarwal, L. A. Bendersky, M. Li, A. Mehta, I. Takeuchi, On-the-fly closed-loop materials discovery via Bayesian active learning. Nat. Commun. 11, 1–11 (2020). 33235197 10.1038/s41467-020-19597-w PMC7686338

[35] J. K. Pugh, L. B. Soros, K. O. Stanley, Quality diversity: A new frontier for evolutionary computation. Front. Robot. AI. 3, 1–17 (2016).

[36] J. Lehman, K. O. Stanley, Evolving a diversity of virtual creatures through novelty search and local comp, in *Proceedings of the 13th Annual Conference on Genetic and Evolutionary Computation* (GECCO, 2011) pp. 211–218.

[37] J.-B. Mouret, J. Clune, Illuminating search spaces by mapping elites. arXiv: 1504.04909(2015).

[38] K. Arulkumaran, A. Cully, J. Togelius, Alphastar: An evolutionary computation perspective, in *Proceedings of the Genetic and Evolutionary Computation Conference Companion* (GECCO, 2019), pp. 314–315.

[39] R. Kaushik, P. Desreumaux, J. B. Mouret, Adaptive prior selection for repertoire-based online adaptation in robotics. Front. Robot. AI. 6, 151 (2020). 33501166 10.3389/frobt.2019.00151 PMC7805922

[40] J. Verhellen, J. Van Den Abeele, Illuminating elite patches of chemical space. Chem. Sci. 11, 11485–11491 (2020). 34094392 10.1039/d0sc03544k PMC8162856

[41] J. Grizou, L. J. Points, A. Sharma, L. Cronin, A curious formulation robot enables the discovery of a novel protocell behavior. Sci. Adv. 6, eaay4237 (2020). 32064348 10.1126/sciadv.aay4237 PMC6994213

[42] A. Rao, M. Schoenenberger, E. Gnecco, T. Glatzel, E. Meyer, D. Brändlin, L. Scandella, Characterization of nanoparticles using atomic force microscopy. J. Phys. Conf. Ser. 61, 971–976 (2007).

[43] A. E. Vladár, V. D. Hodoroaba, *Characterization of nanoparticles by scanning electron microscopy* (Elsevier Inc., 2019);http://dx.doi.org/10.1016/B978-0-12-814182-3.00002-X).

[44] Z. L. Wang, Transmission electron microscopy of shape-controlled nanocrystals and their assemblies. J. Phys. Chem. B 104, 1153–1175 (2000).

[45] T. Zheng, S. Bott, Q. Huo, Techniques for accurate sizing of gold nanoparticles using dynamic light scattering with particular application to chemical and biological sensing based on aggregate formation. ACS Appl. Mater. Interfaces 8, 21585–21594 (2016). 27472008 10.1021/acsami.6b06903

[46] T. Li, A. J. Senesi, B. Lee, Small angle x-ray scattering for nanoparticle research. Chem. Rev. 116, 11128–11180 (2016). 27054962 10.1021/acs.chemrev.5b00690

[47] S. Hessam, M. Craven, A. I. Leonov, G. Keenan, L. Cronin, A universal system for digitization and automatic execution of the chemical synthesis literature. Science 370, 101–108 (2020). 33004517 10.1126/science.abc2986

[48] M. J. Walsh, S. J. Barrow, W. Tong, A. M. Funston, J. Etheridge, Symmetry breaking and silver in gold nanorod growth. ACS Nano 9, 715–724 (2015). 25572634 10.1021/nn506155r

[49] K. Gui, J. Zheng, K. Wang, D. Li, S. Zhuang, FDTD modelling of silver nanoparticles embedded in phase separation interface of H-PDLC. J. Nanomater. 2015, 1–7 (2015).

[50] J. Marcheselli, D. Chateau, F. Lerouge, P. Baldeck, C. Andraud, S. Parola, S. Baroni, S. Corni, M. Garavelli, I. Rivalta, Simulating plasmon resonances of gold nanoparticles with bipyramidal shapes by boundary element methods. J. Chem. Theory Comput. 16, 3807–3815 (2020). 32379444 10.1021/acs.jctc.0c00269 PMC7584360

[51] B. T. Draine, P. J. Flatau, Discrete-dipole approximation for scattering calculations. J. Opt. Soc. Am. A 11, 1491–1499 (1994).

[52] N. B. Piller, O. J. F. Martin, Increasing the performance of the coupled-dipole approximation: A spectral approach. IEEE Trans. Antennas Propag. 46, 1126–1137 (1998).

[53] M. A. Yurkin, M. Min, A. G. Hoekstra, Application of the discrete dipole approximation to very large refractive indices: Filtered coupled dipoles revived. Phys. Rev. E Stat. Nonlinear, Soft Matter Phys. 82, 1–12 (2010). 10.1103/PhysRevE.82.036703 21230209

[54] P. Van Rysselberghe, Remarks concerning the clausius-mossotti law. 36, 1152–1155 (1932).

[55] Q. Wang, Z. Wang, Z. Li, J. Xiao, H. Shan, Z. Fang, L. Qi, Controlled growth and shape-directed self-assembly of gold nanoarrows. Sci. Adv. 3, e1701183 (2017). 29098180 10.1126/sciadv.1701183 PMC5659655

[56] V. Thambi, A. Kar, P. Ghosh, D. Paital, A. R. S. Gautam, S. Khatua, Synthesis of complex nanoparticle geometries via pH-controlled overgrowth of gold nanorods. ACS Omega 4, 13733–13739 (2019). 31497690 10.1021/acsomega.9b01119 PMC6714510

[57] K. S. Lee, M. A. El-Sayed, Dependence of the enhanced optical scattering efficiency relative to that of absorption for gold metal nanorods on aspect ratio, size, end-cap shape, and medium refractive index. J. Phys. Chem. B 109, 20331–20338 (2005). 16853630 10.1021/jp054385p

