# Supplementary material for: An artificial intelligence enabled chemical synthesis robot for exploration and optimization of nanomaterials
Source: Sci Adv. 2022 Oct 7;8(40):eabo2626. doi: 10.1126/sciadv.abo2626 (PMC9544322; doi:10.1126/sciadv.abo2626)
Supplement: Supplementary file 1 — Supplementary Materials Figs. S1 to S115 Tables S1 to S22 References [file sciadv.abo2626_sm.pdf]

Science Advances
AAAS

Supplementary Materials for

An artificial intelligence enabled chemical synthesis robot for exploration and optimization of nanomaterials

Yibin Jiang *et al.*

Corresponding author: Leroy Cronin, lee.cronin@glasgow.ac.uk

Sci. Adv. **8**, eabo2626 (2022)
DOI: 10.1126/sciadv.abo2626

The PDF file includes:

Supplementary Materials
Legends for movies S1 and S2
Figs. S1 to S115
Tables S1 to S22
References

Other Supplementary Material for this manuscript includes the following:

Movies S1 and S2

Supplementary Materials

1. Platform hardware 1
1.1. Overview 1
1.2. The chemical reaction module and pump control 3
1.3. Performing reactions, pH measurement and control 6
1.4. Sample analysis 8
1.5. Seed transfer and storage 10
1.6. Temperature control 11
2. In silico exploration of Au and Au-Ag bimetallic nanoparticles 14
2.1. Algorithms 14
2.1.1. Definitions 14
2.1.2. Exploration algorithm based on MAP-Elites 15
2.1.3. Optimisation algorithm based on global search with local sparseness (GS-LS) 16
2.2. Discrete-Dipole Approximation 17

2.2.1. Methods....18

2.2.2. Results....20

2.3. Au/Au-Ag bimetallic nanoparticles using superellipsoid as the shape descriptor and their spectroscopic properties....22

2.4. Simulated chemical space from uniquely-shaped nanoparticles....27

2.4.1. Simulated chemical space 1....27

2.4.2. Simulated chemical space 2....27

2.5. Exploration in the simulated chemical space....28

2.5.1. Method....28

2.5.2. Class distribution and interconnectivity in the simulated spaces....29

2.5.3. Results....31

2.6. Optimisation in the simulated chemical space....32

2.6.1. Method....33

2.6.2. Results....35

3. Exploration of the experimental chemical spaces in the seed-mediated synthesis of Au nanoparticles....45

3.1. Chemical reagents....49

3.2. Chemical space 1: Seed-mediated synthesis on cuboctahedron single crystals....49

3.2.1. Experimental details....49

3.2.2. Data processing and algorithm....52

3.2.3. Results and discussions....57

3.3. Chemical space 2: Overgrowth of Au nanorods....70

3.3.1. Experimental details....70

3.3.2. Data processing and algorithm....73

3.3.3. Results and discussions....75

3.4. Chemical space 3: Overgrowth of Au nanospheres....96

3.4.1. Experimental details....96

3.4.2. Data processing and algorithm....97

3.4.3. Results and discussions 99
3.5. Estimating the possible experiments in exploration 111
3.6. Mutation, crossover, and random sampling in the exploration algorithm 112
3.7. Monodispersity analysis during exploration 113
3.7.1. Chemical Space 1 113
3.7.2. Chemical Space 2 116
3.8. Time cost analysis during exploration 123
4. Optimisation towards specific optical properties 125
4.1. Similarity, local sparseness, and fitness 127
4.2. Target UV-Vis from rods 128
4.3. Target UV-Vis from octahedra 134
5. Autonomous synthesis of nanoparticles via multistep growth 147
5.1. The directed synthesis graph, reaction graph and hardware graph 147
5.2. The autonomous synthesis of desired nanoparticles 149
5.2.1. Experimental details 150
5.2.2. The graph representation of N1 to N6 151
5.3. The unique digital signature of nanoparticles 151

Supplementary Movies (S1-S2):

Supplementary Movie S1: Basic operations of the platform. The movie contains the operations of liquid dispensing, pH control, liquid transfer, UV-Vis characterisation and vial cleaning on the platform.

Supplementary Movie S2: The autonomous multistep synthesis of six AuNPs with the platform. The movie shows a complete recording of the reproducible synthesis of the six nanostructures relevant to Figure 6.

1. Platform hardware

1.1. Overview

The overall platform architecture of the Autonomous Intelligent Exploration, DIScovery and Optimisation of Nanomaterials (AI-EDISON) system consists of three main assemblies: a central chemical reaction module (CRM), a series of high accuracy syringe pumps and a flow spectroscopic suite**.** The CRM is an upgraded version of the liquid handling platform called the Modular Wheel Platform (MWP) (*22*) with advanced capabilities required for this work. This custom reaction platform was constructed using a combination of 3D printed, laser cut and commercially available components, capable of reaction dispensing, stirring, pH control and sample extraction for analysis/storage. This advanced MWP and analysis hardware allowed us to create the closed-loop system for the algorithm-driven exploration and optimisation of gold nanoparticles (AuNPs). As a basic version of the MWP has been reported previously in detail, it will only be briefly discussed here. There after we will focus on the new, additional modules that make up the CRM here.

The system is controlled using a Lenovo ThinkCentre (Intel i5, 8GB RAM) running on Linux/Ubuntu. Custom hardware responsible for liquid handling/performing reactions was controlled by the in-house Commanduino software library (https://github.com/croningp/commanduino) via RAMPS v1.4 shield connected to Arduino MEGA 2560 prototype board. Syringe pumps (Tricontinent Ltd, C-3000 series) used for liquid handling operations were controlled using our in-house developed python library (https://github.com/croningp/pycont). All software and modules to control the complete platform were written in Python 3. A high-performance QE-PRO spectrometer was used in this system in combination with a PEEK FIA-Z-SMA 905 flow cell (10 mm path length) and DH-2000-S light source, all from Ocean Insight Ltd. The system is also equipped with a NIR-Quest IR high-performance spectrometer, and a second QE-PRO configured for Raman. Control of this spectrometer was achieved via the SeaBreeze python library from Ocean Insight Ltd. pH measurement was carried out using a standard VWR semi-micro probe pH electrode and a data logger (DrDAQ, Pico Technology Ltd) was used for data acquisition. The procedure to control the pH of reaction solutions was detailed in **Section 1.3**. 1/16" and 1/8" PTFE tubing from Cole Palmer was used in combination with PFA flangeless fittings and Luer connections for all liquid transfer. Structural hardware for the platform is built using commercially available OpenBuilds profile and fixtures as well as laser-cut acrylic sheets. For moving parts and general custom hardware, a range of commercially available components were used in combination with 3D printed parts developed as part of the platform's modular architecture. All 3D printed components were designed using Onshape (https://www.onshape.com/en/), cloud-based CAD software and printed using an Objet500 Connex from Stratasys in standards FullCure720 RGD material. Laser cutting of 4- and 6-mm acrylic was

performed on a Monster laser ML1060 with a 130W CO2 laser from Radecal Machines. All design (STL, DXF) and construction files can be found at https://github.com/croningp/NanoDiscovery.

The complete platform can be seen in **Figure S1**. A flow diagram showing the platform capabilities during a 24-reaction batch can be seen in **Figure S2**. Detailed descriptions of each hardware module's concept and design are included in this section in the order they appear in this flow diagram.

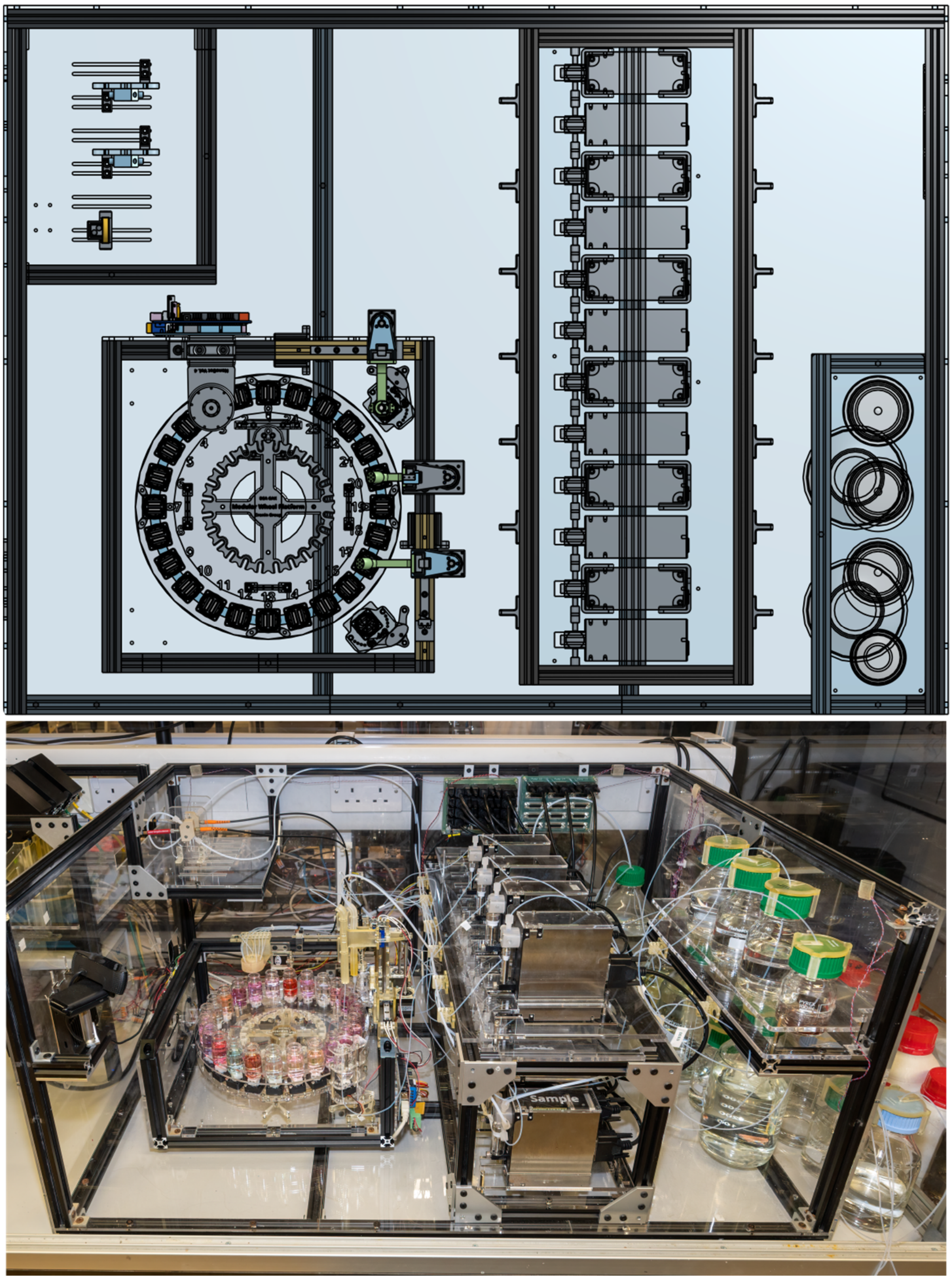

Figure S1. The full autonomous platform. CAD top view (above). Real image of the platform used for this work (below).

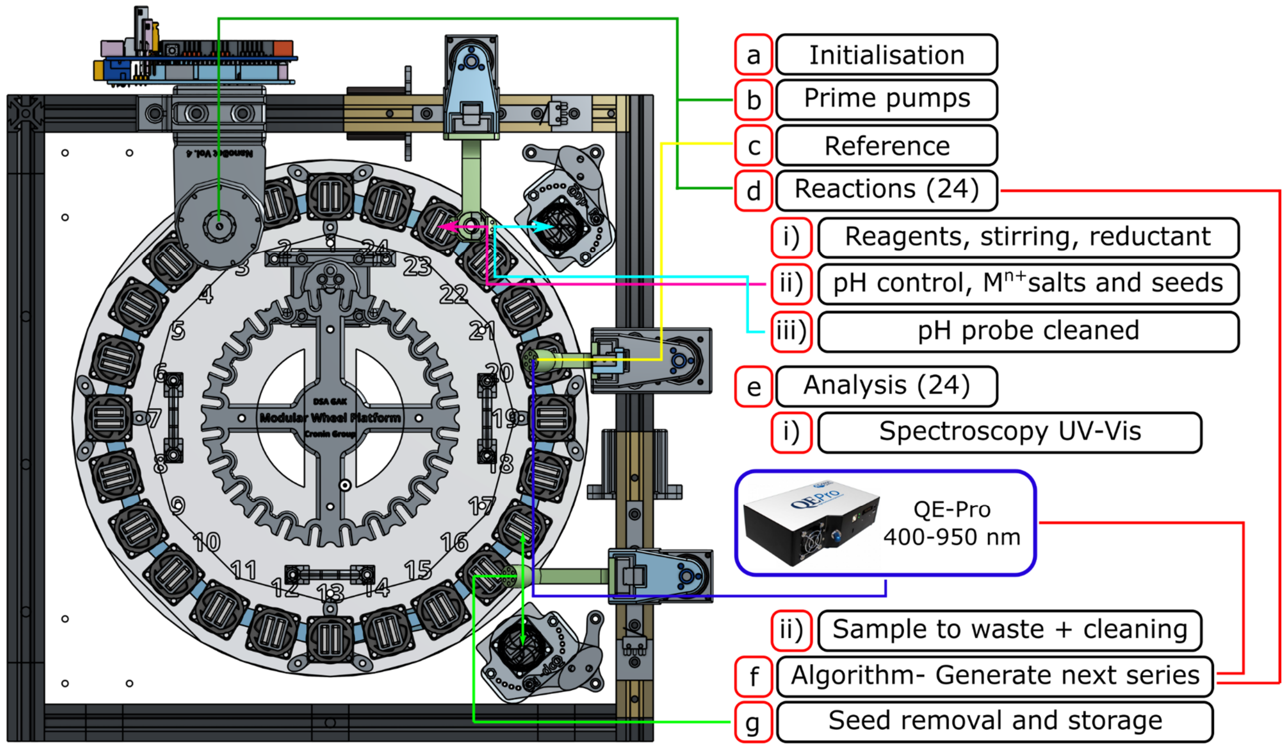

Figure S2. Flow diagram of the operations of the platform in the CRM. (a) The initialisation of the hardware loads the platform configuration (devices connected and their attributes), homes moving components with positioning sensors, ensures access to spectrometers and aligns the pump valves to their default positions. (b) Pumps are primed with reagents to eliminate dead volume dispensing from the start. (c) A water UV-Vis reference is obtained for later analysis and removed. (d) Reactions are performed in which: (d)[i] reagents are dispensed, stirred and reductant added, (d)[ii] pH is measured/controlled, and metallic salts, as well as premade gold seeds, are dispensed and (d)[iii] in parallel, the pH probe is moved to and cleaned at its wash once the pH control is finished. (e) After a growth period, (e)[i] the samples are transferred to the flow cells and analysed. After analysis (e)[ii] the sample is moved to waste and the flow apparatus is cleaned to get ready for the next sample. (f) Data is fed into an algorithm to produce the next series of reactions. (g) During a multistep synthesis process, certain samples can be moved to other samples as the seeds or stored temporarily using the seed transfer unit. The synthetic procedure of adding reagents in (d) can vary during exploring different chemical spaces and will be discussed respectively.

1.2. The chemical reaction module and pump control

Reactions are performed on the chemical reaction module, and a GitHub repository providing full details of this base unit's construction, bill of materials and software can be accessed at https://github.com/croningp/NanoDiscovery. In short, the platform uses a Geneva drive to turn a tray of 24 reaction vessels, each stirred from below using a magnetic stirring mechanism. Pumps provide reaction materials at any desired position of the wheel via several 3D printed dispensing units. Multiple modules can be attached to the v-slot profile frame of the platform to access any position around this reaction tray. The functions of these modules can range from dispensing, probe analysis, cleaning, etc., all of which are custom-designed for the system and can be added and removed with ease in terms of both hardware and control software. For the chemical reaction used here: dispensing, pH measurement and control, probe cleaning, sample transfer for analysis and vial/flow cells cleaning

were required to complete a reaction sequence, which typically consisted of 24 reactions. The CAD render of this version of the chemical reaction module is included below alongside the complete unit in **Figure S3**. Each module for the functions listed above will be detailed individually.

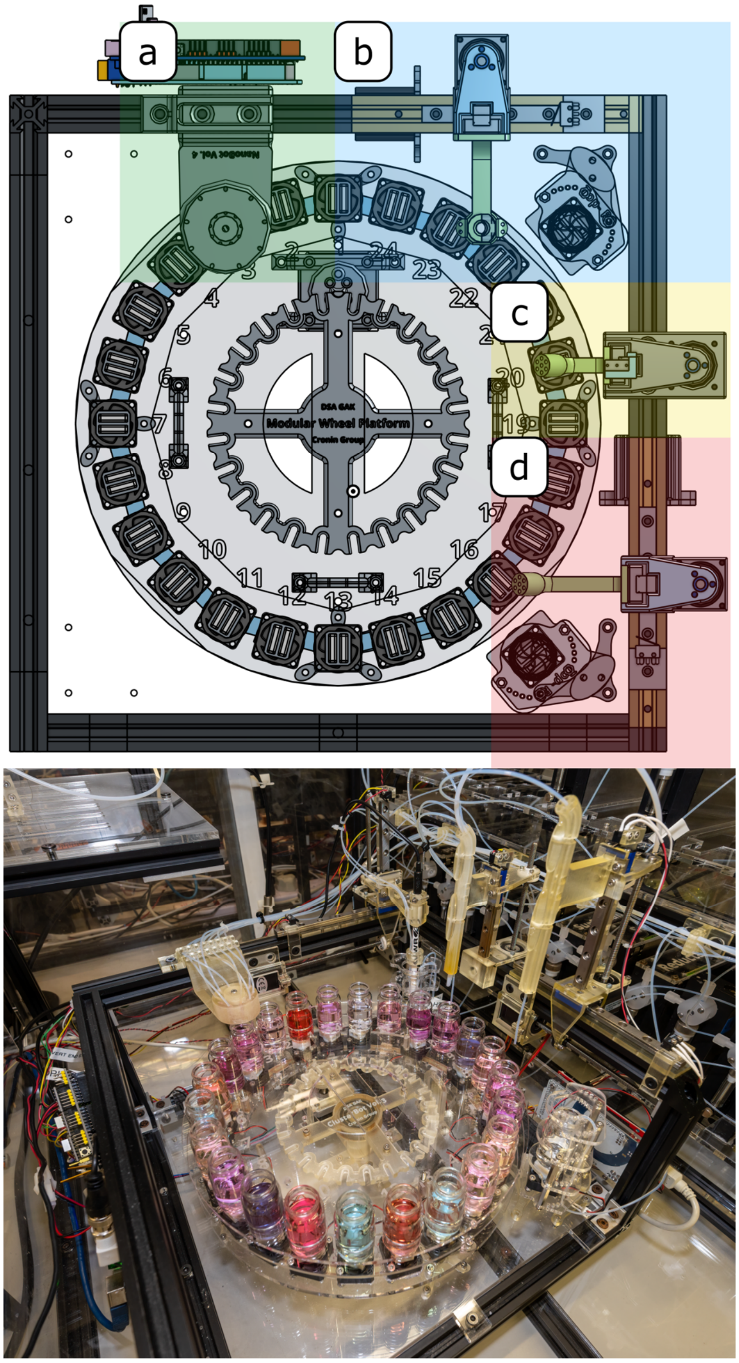

Figure S3. The chemical reaction module. Top view in CAD (above): (a) 3D printed dispensing unit. (b) pH control module and wash station. (c) Sample transfer module 1 for analysis. (d) Sample transfer module 2 for seed. Real image of the working platform (below).

A series of four- and six-way valve C3000 series syringe pumps from TriContinent were used with syringe volumes ranging from 100 µL to 5 mL as requested. The pumps are connected to TriCont hubs and are controlled directly with our in-house PyCont software library. The pumps and TriCont hubs used in this work can be seen in **Figure S4**.

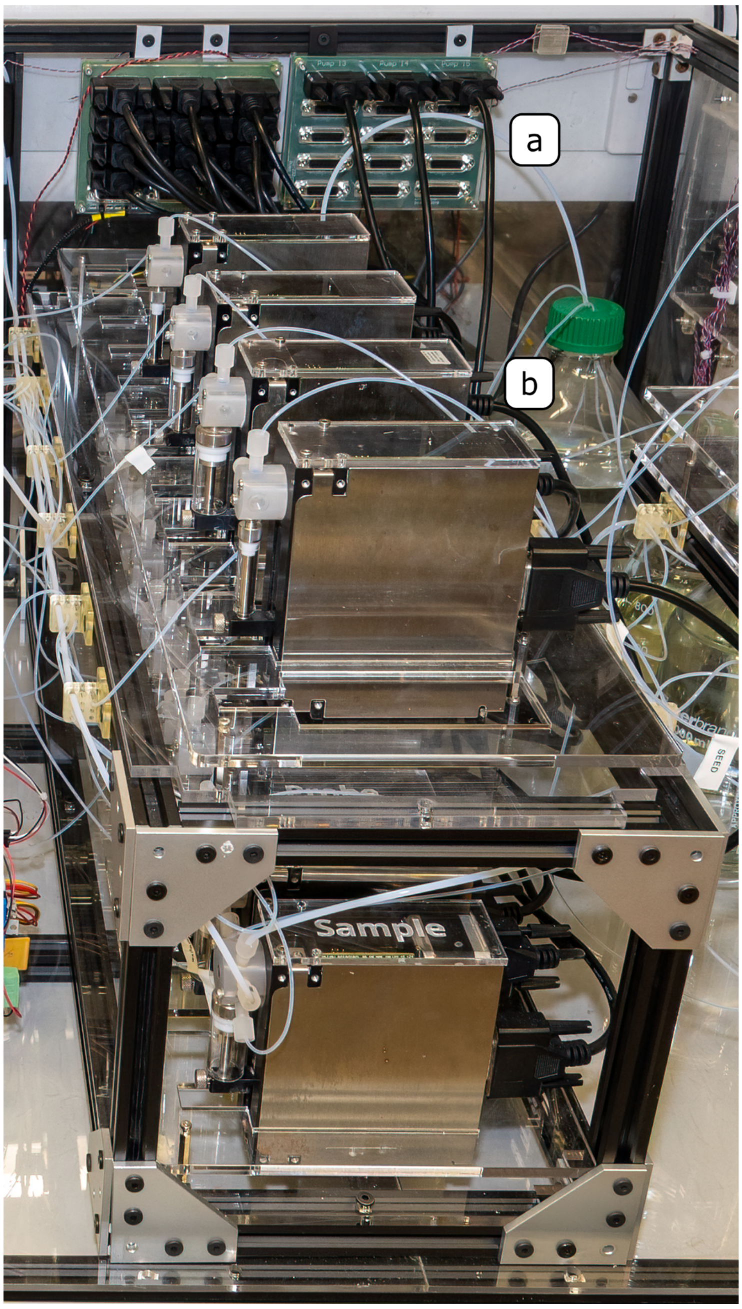

Figure S4. The set-up for the pumps. (a) Pump communication hubs. (b) TriContinent syringe pumps.

Up to 15 pumps can be powered and controlled via a single custom-designed hub (TriCont hub), created in-house by Dr. Sergey Zalesskiy. TriContinent C3000 pumps use a standard DA-15 connector for both data and power. They implement three different protocols for communication - RS-232, RS-485 and CAN-signals which are available in the output connector. To make the whole pump assembly and the cabling more compact and avoid manual daisy-chain cable crimping, a simple hub unit was made. The hub consists of a PCB sandwiched between two sheets of acrylic (4 mm thick for the front; 6 mm thick for the back) acting as a case. The acrylic sheets are fixed to the board by means of standard PCB standoffs. The PCB itself carries 15 standard straight DA-15 female connectors in a 5 × 3 matrix. This number is governed by the address limitation of the pumps

themselves - the address is set with the rotary switch at the back which has 15 possible positions. Multiple hubs could be used for more than 15 pumps. The RS-485 A and B signals from all connectors are connected in series and length-matched. No termination was implemented as the pump has an embedded switchable line terminator. The power pins in the connectors are connected to the top and bottom planes of the PCB which carry VCC and GND polygons. The top acrylic sheet has cut openings for the DA-15 connectors. The pumps are connected to the board using standard DA-15 female-to-female cables. The board is connected to the computer by means of a USB to UART converter cable from FTDI Ltd.

1.3. Performing reactions, pH measurement and control

The order of addition of reagents in nanoparticle synthesis is crucial to produce high-quality species. In a typical seed-mediated synthesis of the type used throughout this work (e.g., synthesis of Au nanorods), $HAuCl_4$ is reduced in the presence of a concentrated surfactant solution, using a weak reducing agent, in our case ascorbic acid or hydroquinone. Next, a symmetry-breaking agent may be added e.g., $AgNO_3$ and finally, a pre-synthesised Au seed is added. In our system, we wanted to accurately control the pH of the growth solution in which the particle forms. To avoid the direct reduction of metallic species on the pH probe, the measurement and control of the pH was performed before the addition of any metallic species. To do this, we move the current working vial, four positions clockwisely from the dispensing position to be accessed by a probe (**Figure S5**). The probe is mounted on a module capable of horizontal and vertical motion, reaching two pre-set positions: the reaction vial position which is four positions away from the dispensing position, and a wash station positioned alongside the vial tray. The module comprises two Nema11 lead screw motors to achieve the X and Z motion along the platform frames. 3D prints bind the lead screws of these motors to precision steel rails and carriages that provide smooth motion. Mechanical endstop switches define the home positions of these motors. Attached to the Z motion, the 3D print is a holder for the pH probe as well as tube guides for the acid and base solutions used to modulate the pH. Details of the strategy developed to reach the desired pH as efficiently as possible can be found in **Section 3.3**. Once a reaction has reached the target pH, the module returns to the cleaning vial containing Type I ultrapure water as a cleaning solution. The vial is stirred from the bottom using the same mechanism as the main vial tray. This vial is cleaned, and the solution is replenished in parallel to the operations of the wheel to prevent contamination between samples and also acts as a storage position of the pH probe once the sequence is finished by dispensing saturated KCl (3 M). **Figure S6** shows both a CAD image of the design alongside the built unit as well as the cleaning vial station. The pH probe is calibrated autonomously each day by performing a similar series of actions between three buffer solutions on the vial try and the wash station.

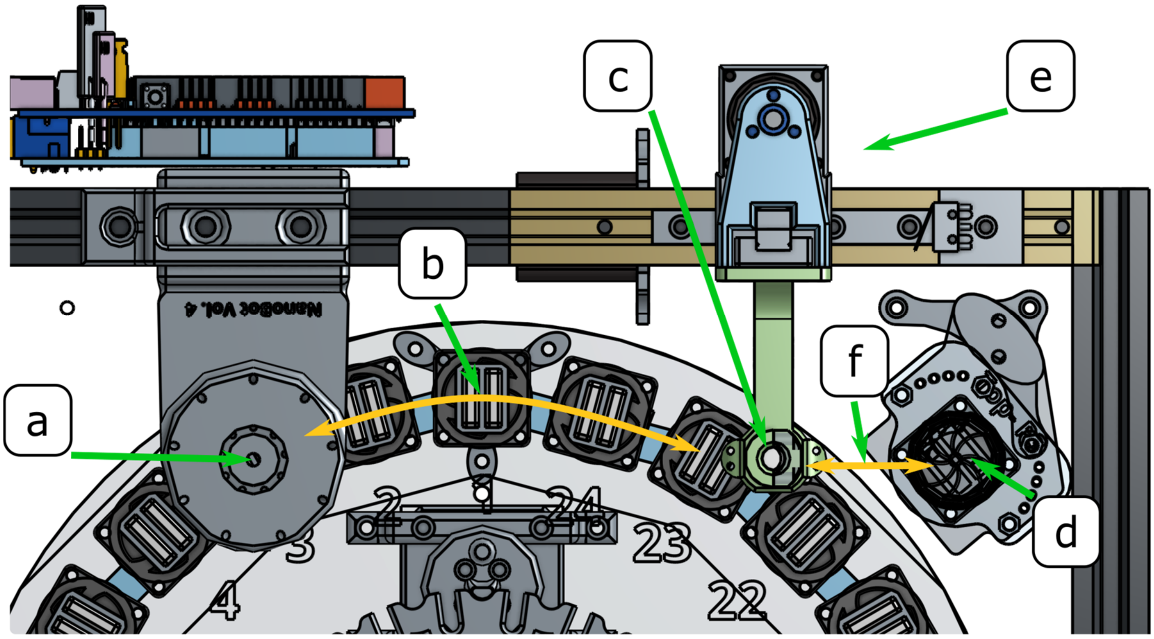

Figure S5. Reaction and pH control sequence. (a) Dispensing position. (b) Four vial position shifts of the current working vial for pH control before returning to position (a) for seed addition. (c) pH probe position. (d) pH probe wash station. (e) X and Z motion module holding pH probe. (f) The motion of the pH probe between the reaction vial and the cleaning position.

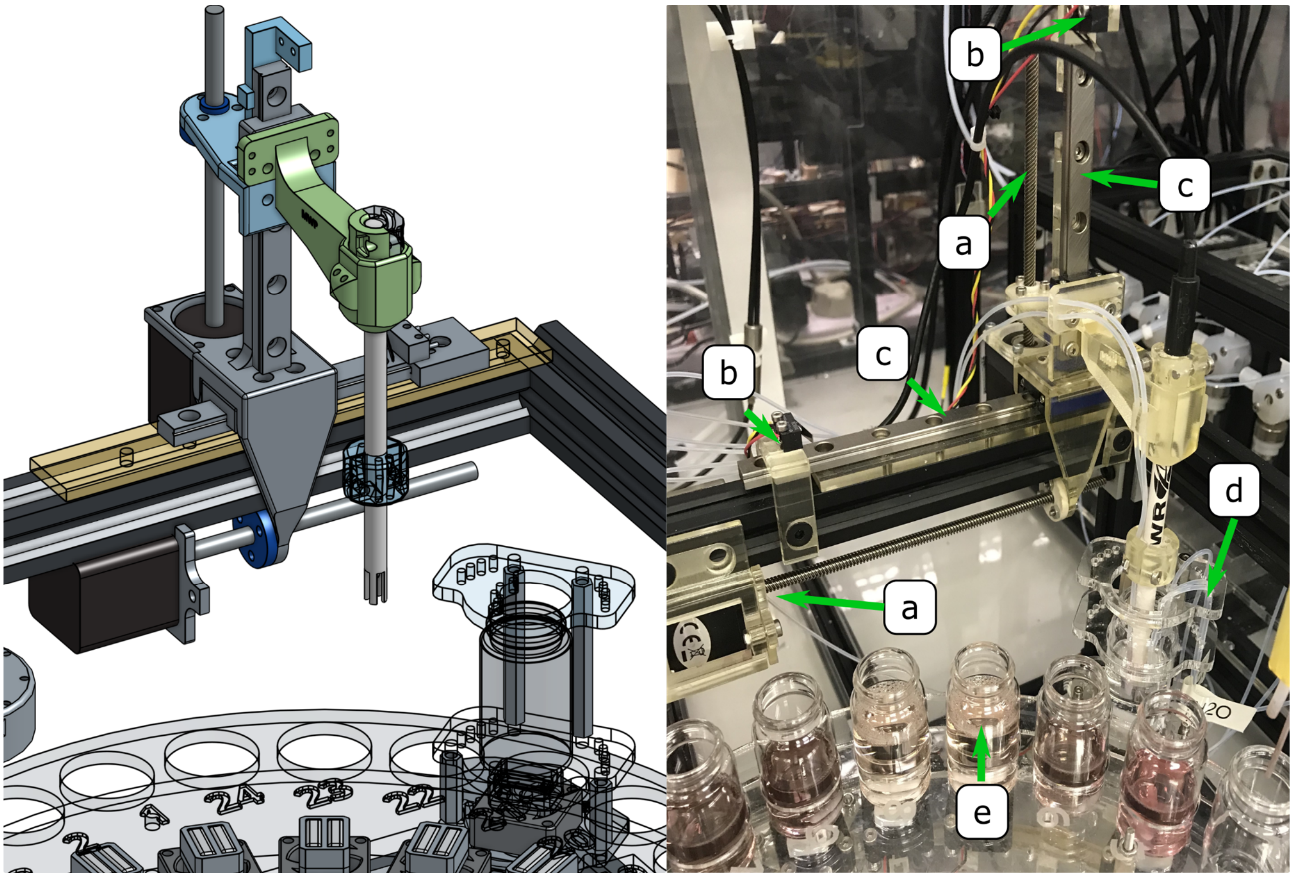

Figure S6. The pH control module in CAD (left) and real (right). (a) Nema 11 stepper motor with a lead screw. (b) Mechanical endstop switch. (c) Precision rail. (d) Wash station with cleaning and storage solution. (e) pH control vial position.

1.4. Sample analysis

Once a pre-defined growth period has passed, the samples are analysed autonomously by UV-Vis spectroscopy. A modular syringe driver (MSD) is secured to the platform frame at vial position seven (position one being the dispensing position with position index increased clockwisely), with a 3D printed multichannel tube attachment. This unit houses tubes which lead to different locations like the UV-Vis flow cells or are used for stock solutions in flow cell wash cycles. For sample extraction, the unit is lowered into the reaction solution and a dedicated pump moves 5.0 mL of material through the UV-Vis line first. Then the pump drives another 5.0 mL of the sample through the flow cell at high speed to prevent bubbles from remaining in the sample lines. The volume was varied to 3.5 mL in chemical space 2 considering the possible smallest volume of the sample.

The outlet of the flow cell is directed back into the multichannel attachment to return the sample to its vial. The UV-Vis spectra are recorded using a high-performance QE-PRO absorbance spectrometer from Ocean Insight (400-950 nm). The sample is then removed to waste from the same position and the vial is filled with Type I ultra-pure water that will be flowed through the sample path multiple times. This wash cycle repeats five times. The MSD is removed from the vial and the next sample moves to the analysis position. This cycle is repeated for all samples in a step of 24 experiments. **Figure S7** shows the multichannel extraction attachment and the unit set up on the platform itself.

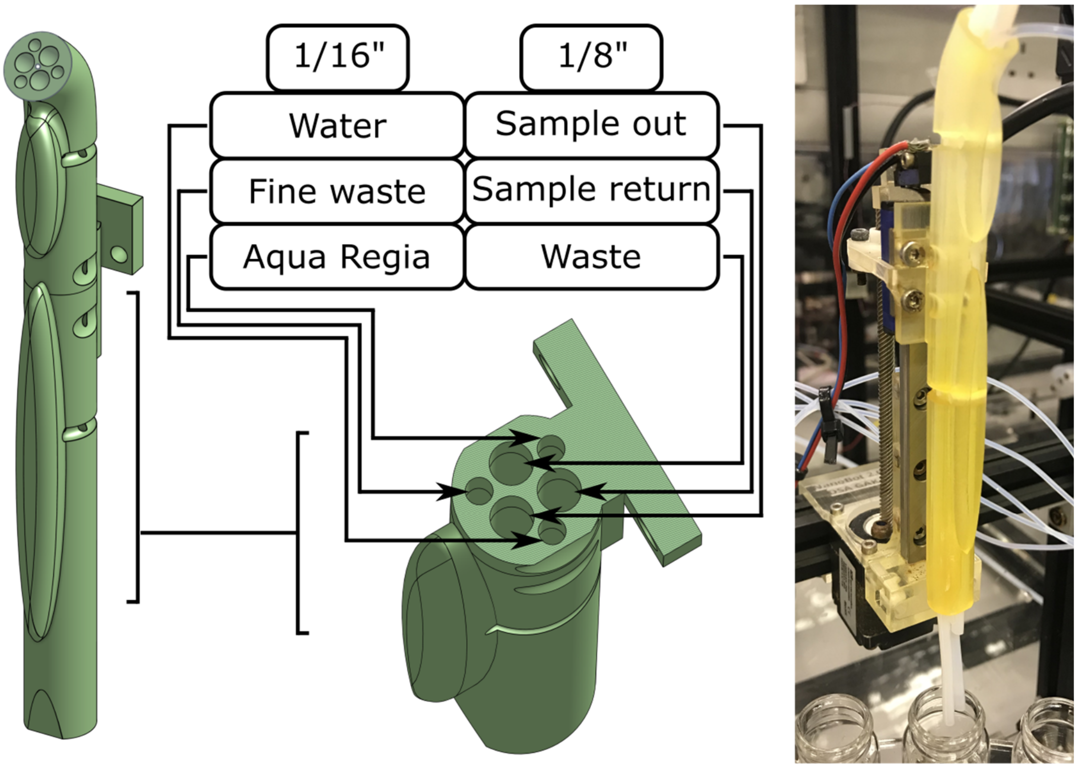

Figure S7. Cross-section view and real image of the multi-channel extraction MSD. The tube channels are shown in the figure.

The platform is built to accommodate multiple flow cell/spectrometer assemblies despite only using UV-Vis for this work. The flow cells are housed inside the platform's temperature-controlled box, above the liquid handling unit. Short optics join the cells to the spectrometers housed outside the box to avoid the spectrometer cooling systems fighting the excessive temperature within the box. The current design has UV-Vis, IR (900-1700 nm) and Raman spectrometers in the assembly. The light and laser sources for these units are housed beneath the spectrometer shelf, with the incident optics being fed into the box. **Figure S8** shows the set-up.

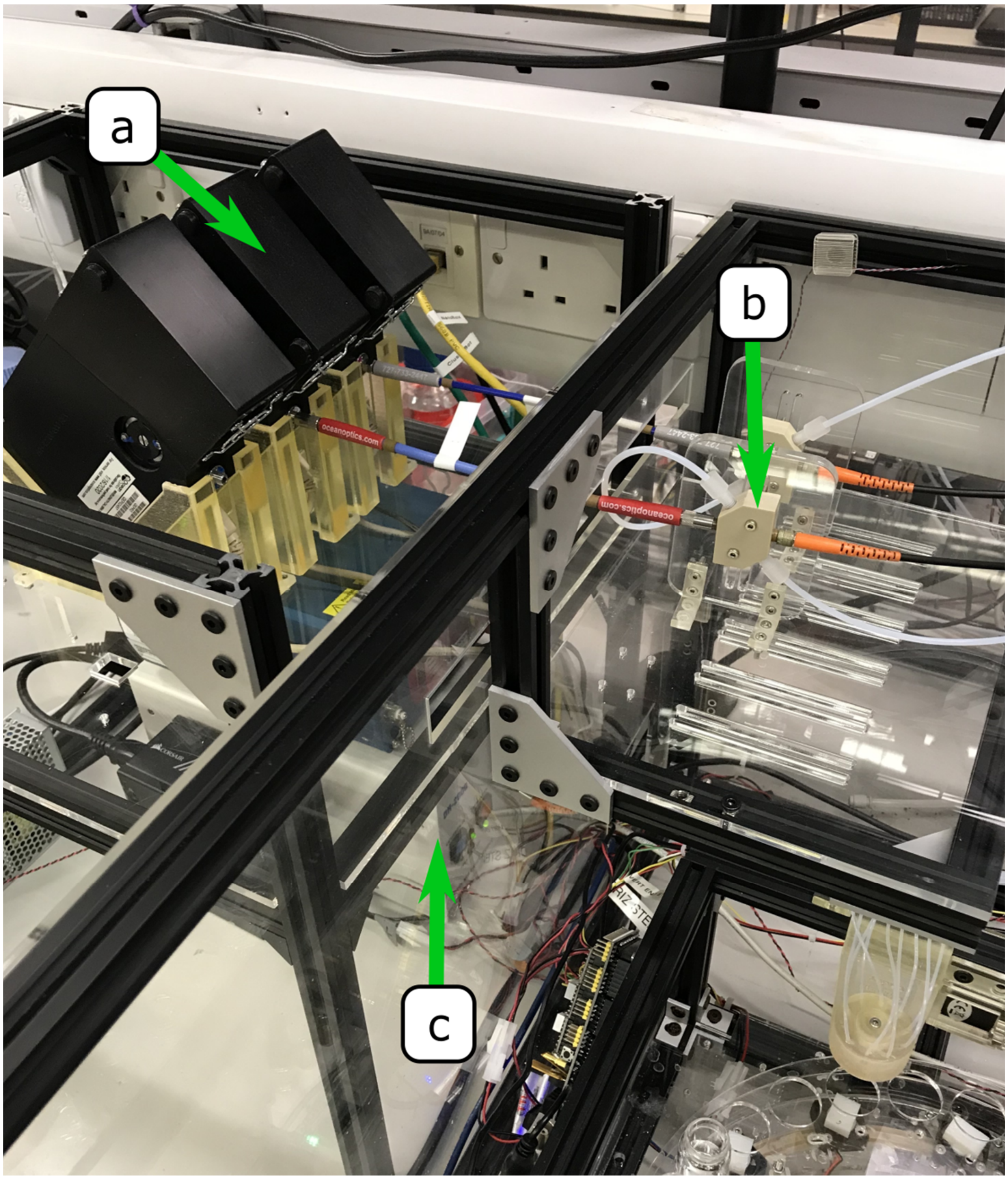

Figure S8. Analysis set-up. (a) Three high-performance spectrometers for UV-Vis, IR and Raman. (b) Flow cell assemblies housed inside the temperature-controlled box. (c) Light and laser sources.

1.5. Seed transfer and storage

In performing the multistep synthesis, many of the samples produced are both products and future reagents as seeds. For each nanoparticle, several repeats of the sample can be performed on the wheel (More details in **Section 5**). One or more of these samples needed to be transferred to an off-wheel location for later use. For this, a module that is a combination of the sample extraction and pH modules was built. It requires the same X and Z motion of the pH assembly and a similar 3D printed multichannel part to accommodate extraction and cleaning tube. Before the addition of seed, the seed transfer unit would move to a vial position, remove a portion of the sample and store the sample in a pump temporarily. By turning the wheel and moving the unit back to the vial position, the stored solution can be used as seeds. Once this seed solution was used, the tube in the module and the pump connecting to it will be cleaned in a wash station. **Figure S9** shows this set-up.

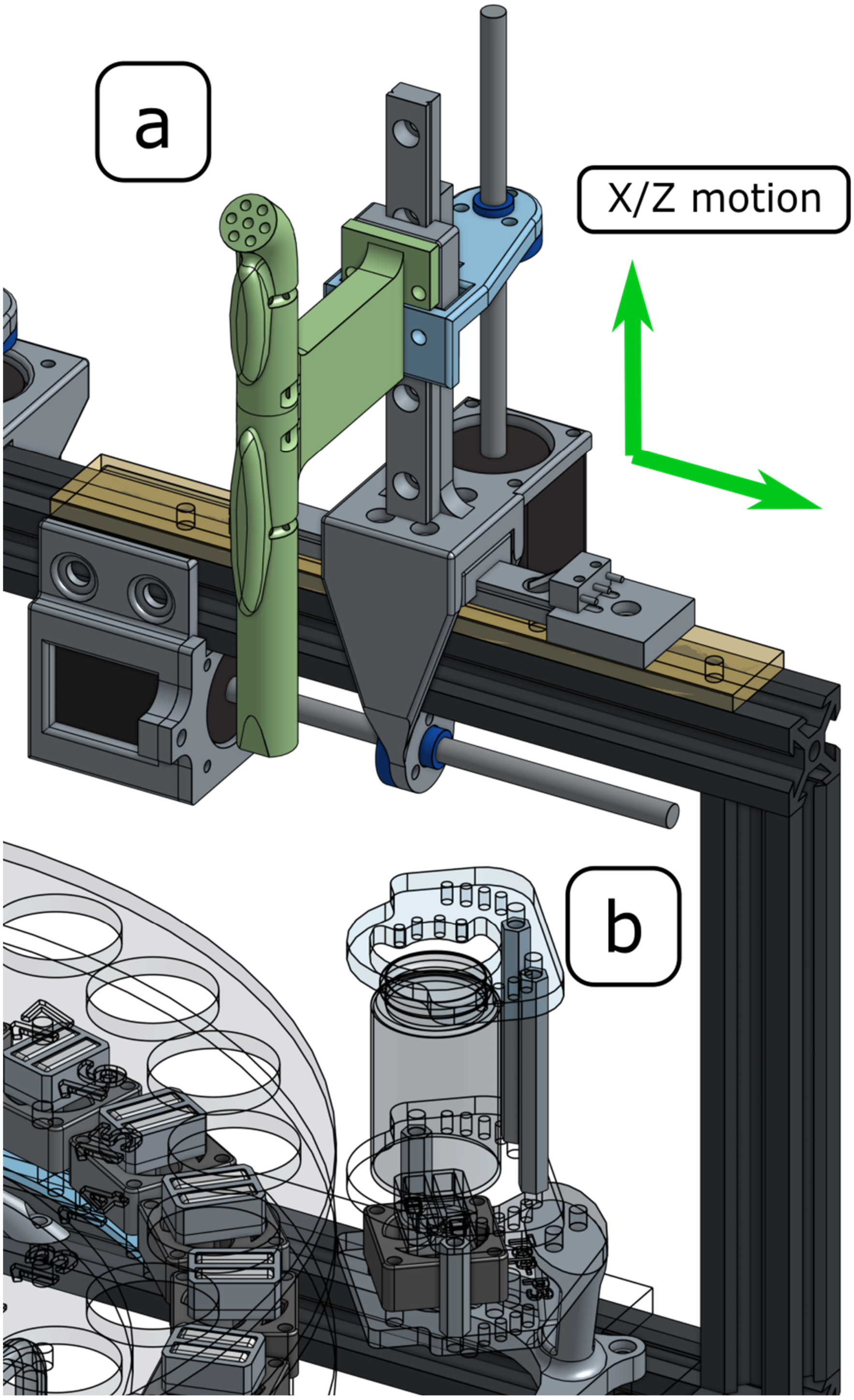

Figure S9. Sample extraction and cleaning module for future seeds. (a) Multichannel 3D print for tubing. (b) Wash station of the seed transfer unit.

1.6. Temperature control

The entire liquid handling system is contained in a temperature-controlled box set to 30°C. There are many reasons for this including preventing surfactants in stock or reaction solutions from precipitation, keeping the growth temperatures identical throughout the discovery process and for the reproducibility of samples. The box itself is a simple structure of v-slot aluminum rails with 4 mm acrylic sheets as boundaries. The temperature inside the platform box was controlled using an RE72

PID temperature controller from Lumel (configuration with T-type thermocouple input). The PID settings were determined upon first start-up using the in-built autotune function (Ziegler-Nichols method). T-type thermocouples were used as sensors as they have better accuracy compared to more commonly used K-type thermocouples. To provide an average reading over the whole box, five individual thermocouples were connected in parallel and secured evenly around the interior of the box. All the thermocouples were two meters long, however, an extra swamping resistor was connected in series with each thermocouple to compensate for possible differences in resistance. The on/off output of the PID controller was fed into a Crydom D2410 solid-state relay controlling the fan heater. The fan heater was mounted on the ceiling with the air flow directed to the side wall to cause minimal disturbance inside the box. The PID controller and all accompanying electronics were in a small acrylic box mounted outside the heated volume. The connections for the fan heater and the thermocouples were fed into the main box through the brush plate pass-through. The system was started at least one hour before the actual experiment started to allow the temperature inside the box to become steady. **Figure S10** shows the heater (real) mounted inside the box and the control unit, and **Figure S11** shows the box, mounted heated and thermocouple locations (designed).

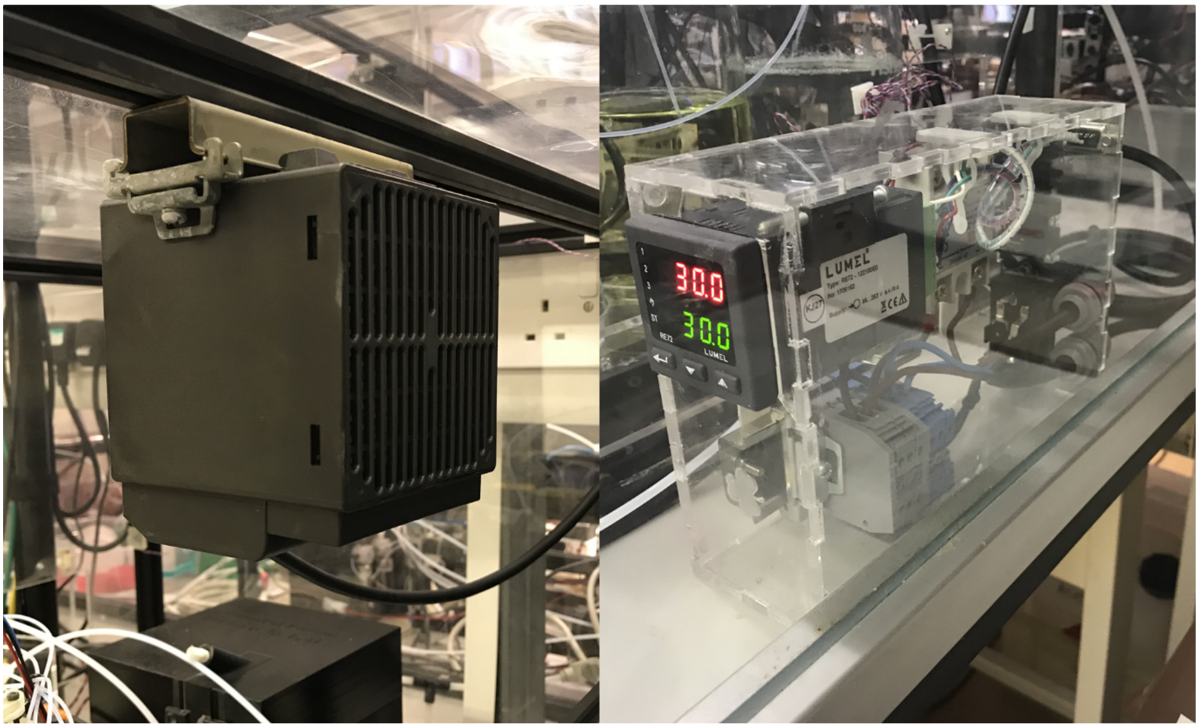

Figure S10. Fan heater and control unit for temp control of the sealed assembly. The fan and the controller are shown on the left and right respectively. The target temperature and current temperature are shown at the top and bottom of the controller.

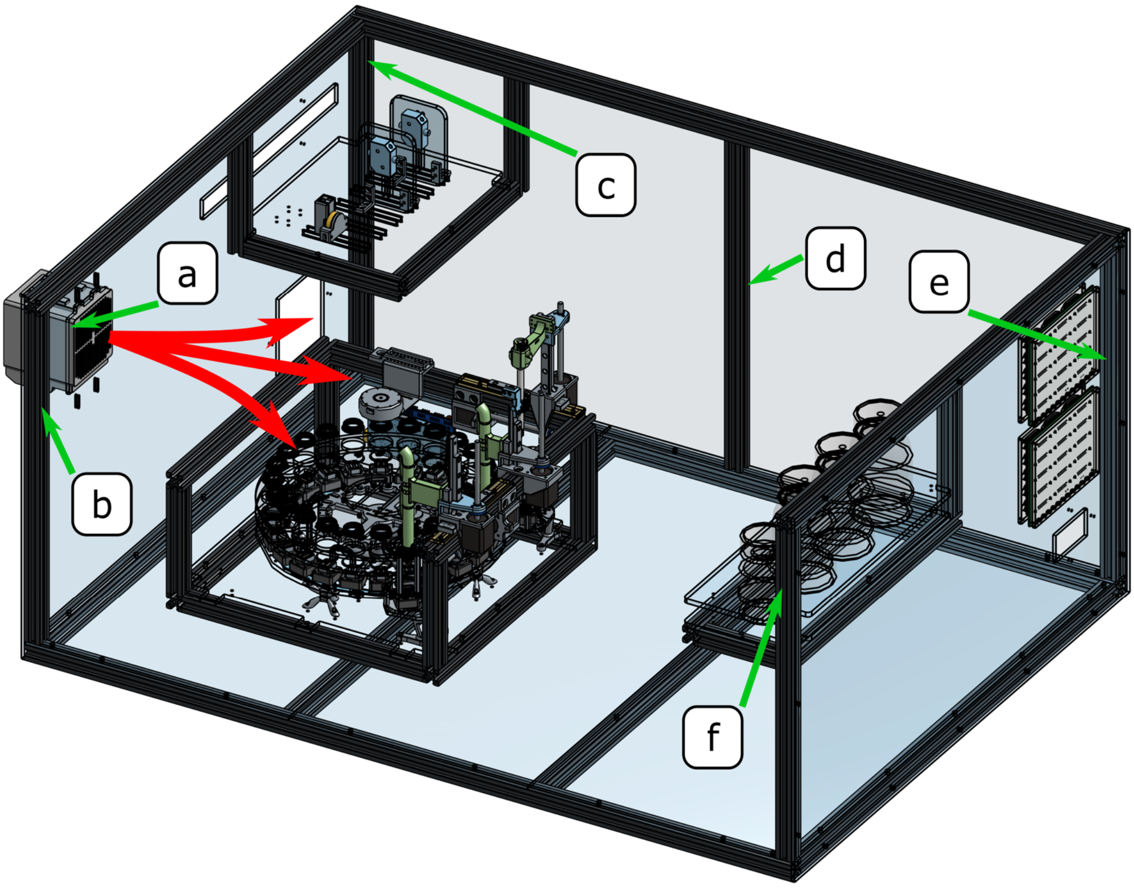

Figure S11. Temperature-controlled box with the direction of heating flow. (a) Stego fan heater. (b-f) the five thermocouple positions.

2. In silico exploration of Au and Au-Ag bimetallic nanoparticles

In this section, the exploration and optimisation algorithms based on the Multi-dimensional Archive of Phenotypic Elites (MAP-Elites) and Global Search with Local Sparseness (GS-LS) to search nanoparticles were benchmarked in simulated chemical spaces. In **Section 2.1**, we will give a brief introduction to both algorithms. **Section 2.2** will describe a general discrete-dipole approximation (DDA) method to simulate the extinction spectra of arbitrarily shaped metallic nanoparticles. Based on it, a GPU-accelerated Python (PyDScat-GPU) package was developed utilising Tensorflow 2 for efficient extinction simulations. In **Section 2.3**, a family of different shapes were generated with superellipsoid as the shape descriptor. Using these shapes as templates, the same shaped Au and Au-Ag bimetallic nanoparticles were created *in silico* and the corresponding extinction spectra were simulated with DDA. In **Section 2.4**, the Au and Au-Ag bimetallic nanoparticles and their spectra from **Section 2.3** were used to create two simulated chemical spaces respectively. In **Section 2.5**, the two simulated chemical spaces were explored with the exploration algorithm, with its performance benchmarked to Random Search, where the whole space is sampled by a uniform distribution. With all the data available from the exploration, we further benchmarked the optimisation algorithm to tune the sophisticated UV-Vis features in the Au-Ag bimetallic space in **Section 2.6**.

2.1. Algorithms

2.1.1. Definitions

Considering an N-dimensional input chemical space that needs to be searched, any sampling point in the space is represented by a vector $\boldsymbol{x} = \{x_1, x_2, .., x_N\}$, where x_i is the input value in the ith dimension. This vector precisely defines the synthetic conditions with their corresponding operations, e.g., the adding volumes of a series of reagents. The input set of existing samples is defined as $\boldsymbol{X} = \{\boldsymbol{x}_1, \boldsymbol{x}_2, \boldsymbol{x}_3, \dots, \boldsymbol{x}_M\}$, where $\boldsymbol{x}_j$ is a vector representing the input of the jth sample. The spectra of the samples are available by sampling the space, which are defined as $\boldsymbol{Y} = \{\boldsymbol{y}_1, \boldsymbol{y}_2, \boldsymbol{y}_3, \dots, \boldsymbol{y}_M\}$, where $\boldsymbol{y}_j$ represents the spectrum of the jth sample.

The behaviour of the sample can be estimated by their attributes ($\boldsymbol{a}(\boldsymbol{y})$) based on the spectral observation, which includes the number of the UV-Vis peaks and the positions. The set of the samples' attributes is denoted as $\boldsymbol{A} = \{\boldsymbol{a}_1, \boldsymbol{a}_2, \boldsymbol{a}_3, \dots, \boldsymbol{a}_M\}$. The attributes were used for classification, and the set including the classes is denoted as $\boldsymbol{C} = \{c_1, c_2, c_3, \dots, c_M\}$, where c_j is the class index of the jth sample. Based on the spectra, the performance of the sample can be quantified by a fitness function ($F(\boldsymbol{y})$). This fitness function is correlated with the desired UV-Vis features during the search. Thus, a set of fitness values is defined as $\boldsymbol{F} = \{F(\boldsymbol{y}_1), F(\boldsymbol{y}_2), F(\boldsymbol{y}_3), \dots, F(\boldsymbol{y}_M)\}$, where $F(\boldsymbol{y}_j)$ represents the fitness value of the jth sample. The fitness function (F) can be defined as either dependent or

independent of the class index, which will be discussed when the algorithms were implemented below. **The sample with the highest fitness (performance) within one class was defined as an elite, which guides the exploration process later**.

2.1.2. Exploration algorithm based on MAP-Elites

Inspired by the literature on evolutionary algorithms, MAP-Elites (*37*) is an illuminating search algorithm designed to explore a feature space. The feature space was defined by both the performance and the behaviour of the samples. The procedure was as follows:

1. For samples ($\boldsymbol{X}$), obtain their spectra ($\boldsymbol{Y}$) by sampling the space.
2. Based on the spectra, obtain the samples' attributes ($\boldsymbol{A}$) like peak numbers or positions.
3. Classify the samples according to their attributes ($\boldsymbol{A}$) to obtain the corresponding class set ($\boldsymbol{C}$).
4. Calculate the fitness ($\boldsymbol{F}$) of the samples according to their spectra ($\boldsymbol{Y}$) and classes ($\boldsymbol{C}$).
5. Select the sample with the highest fitness **within one class** as an elite. Get a set of elites ($\boldsymbol{E}$) from different classes.
6. Use the elites as the parents to crossover and mutate their input variables, which will generate a set of new samples. A small portion of random sampling can be added.

The above procedure defines one step of exploration. It iterated multiple steps until the exploration was complete, see **Figure S12**. Its implementation in the benchmark using the simulated chemical space, or exploring the experimental chemical space will be discussed in **Section 2.5** and **Section 3** respectively.

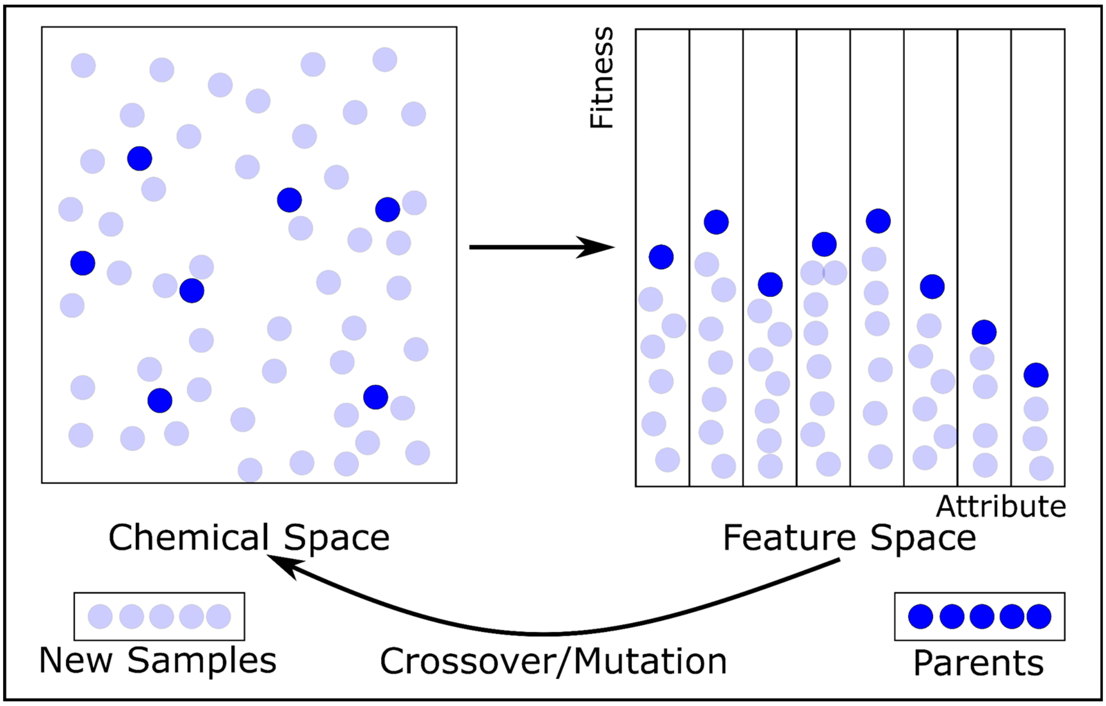

Figure S12. The scheme for the exploration algorithm based on MAP-Elites. All the samples in the chemical space are projected to the feature space according to their fitness and attribute. Depending on the attribute, certain criteria were used to classify samples with different behaviours. Here we discretize the behaviour space into multiple subregions, and the classification was conducted by selecting which subregion the sample are located in. The sample with the highest fitness within each class is regarded as an elite and added to the parent set. New experiments are generated by the crossover and mutation of the parents, and the chemical space was explored by iterating this process.

2.1.3. Optimisation algorithm based on global search with local sparseness (GS-LS)

The optimisation algorithm was based on global search with local sparseness (GS-LS), which was inspired by the novelty-search algorithm (*36*) that considers the novelty of a sample by measuring its local sparseness in a behaviour space. However, in our system we have focused on this measure of local sparseness in the input space instead. Local sparseness in the input space is defined as the local sampling density around the current sample of interest. The algorithm then considers the local spareness of data points around a given sample as part of the overall fitness measure. We then use the lack of or abundance of samples in particular regions to encourage the search in less sampled regions for a global search.

In the optimisation, the desired UV-Vis spectrum was set as the target. The aim is to find multiple conditions that locally have the most similar UV-Vis spectra to the target. By considering the local sparseness in the input space and adding it to the fitness function, the algorithm was encouraged to search less-sampled regions, which helps to escape from local maxima and also to search for samples that are separated in the input space but still show high fitness. The local sparseness (S) near a sampling point ($\boldsymbol{x}$) in the input space was measured by the average distance from its K-nearest neighbours ($\boldsymbol{x'}_i$) as shown in Eq. (1). A similarity metric (M_S) considering the difference of peak positions and the whole spectrum between the sample and the target was defined to guide the optimisation (Eq. (2)). A linear summation of both local sparseness (S, Eq. (1)) and similarity between the target and the sample (M_S, Eq. (2)) was used to define the fitness (F, Eq. (3)).

$$S(x) = \frac{1}{K}\sum_{i=1}^{K} dist(\boldsymbol{x}, \boldsymbol{x'}_i) \quad (1)$$

where $dist(\boldsymbol{x}, \boldsymbol{x'}_i)$ measures the Euclidean distance between $\boldsymbol{x}$ and $\boldsymbol{x'}_i$ in the input space, and $\boldsymbol{x'}_i$ is the ith closest neighbour to $\boldsymbol{x}$.

$$M_S = -\left|p - p_{target}\right| - k_1 \sum_i \left|I_{x,i} - I_{target,i}\right| + k_2 \quad (2)$$

where p and p_{target} are the peak positions of the highest peak in the UV-Vis spectra of the sample and the target. $I_{x,i}$ and $I_{target,i}$ are the ith intensity of the UV-Vis data of the sample and the target respectively. k_1 is used to tune the importance between constraining the peak position and increasing

the overall similarity of the spectra. When two identical UV-Vis spectra are found, both $|p - p_{target}|$ and $\sum_i |I_{x,i} - I_{target,i}|$ reduces to 0 and k_2 puts an upper boundary of the similarity metric.

$$F = M_S + k_3 S \tag{3}$$

where k_3 is a coefficient to tune the relative importance between M_S and k_3.

Then an optimiser can be used to increase the fitness. We used the evolutionary algorithm (EA) as the optimiser in this paper.

After the optimisation, all the samples' UV-Vis similarities to the target were quantified. Multiple solutions were selected so that for every solution, compared to its K-nearest neighbours, it has the most similar UV-Vis to the target according to the similarity metric (M_S). They can represent the local optimal solutions in the observation set. The values of K in calculating the local sparseness and selecting the solutions are not necessarily the same. Depending on the target UV-Vis and chemical space, these solutions can be close or distinct in the input space, which can correspond to nanoparticles of similar or completely different shapes. The implementation of the optimisation algorithm in the benchmark using the simulated chemical space, or optimising the experimental chemical space will be discussed in **Section 2.6** and **Section 4** respectively.

2.2. Discrete-Dipole Approximation

The electromagnetic properties of metallic nanoparticles are closely correlated to their structures due to the plasmon resonance effect. Theoretical tools to study the electromagnetic field of arbitrarily shaped nanoparticles include the finite difference time-domain (FDTD) (*49*), the boundary element method (BEM) (*50*) and the discrete-dipole approximation (DDA) (*51*). Here we used the DDA to simulate the extinction spectra of nanoparticles. The DDA, with a publicly available software DDSCAT developed by B.T. Draine and P. J. Flatau (*51*), is widely used to study the optical properties of nanostructures. In this method, the nanoparticle was discretized into N point dipoles as an approximation. Every dipole is induced by the incident beam as well as the electric field from other dipoles. The system composed of dipoles is self-consistent and can be solved. It requires solving a linear system with 3N equations. In DDSCAT, the complex-conjugate gradient (CCG) and fast Fourier-transform (FFT) method is used to solve the linear system where the time cost is $O(\mathrm{N}^3)$. With the development of graphics processing units (GPUs), the operations on the matrix are faster and we can alternatively solve the linear system by directly matrix inverse. Taking the advantages of GPUs, we developed a Python package, PyDScat-GPU, to simulate the UV-Vis spectrum efficiently.

2.2.1. Methods

First, the nanostructure was discretized into N polarizable cubic lattices which represent the point dipoles. Every point dipole's polarizability was associated with the local dielectric constant. The dipole was induced by the incident beam and also the electric field from the rest of the dipoles. To solve the dipoles and make them self-consistent, the system can be described by simplifying the Maxwell equations into a set of linear equations (Eq. (4)).

$$\mathbf{AP} = \mathbf{E} \tag{4}$$

where $\mathbf{E}$ is a 3N vector describing the local electric field of the incident wave in every dipole position, $\mathbf{A}$ is a 3N by 3N matrix depending on the geometry and materials of the nanoparticle and $\mathbf{P}$ is a 3N vector describing the solutions for the individual dipoles. See reference (*51*) for full details of this linear system.

After solving $\mathbf{P}$, we can further evaluate the local electric field distribution, extinction and absorption cross-sections via the following Eq. (5), Eq. (7), and Eq. (8) respectively.

$$\mathbf{E}_i = \mathbf{E}_{i,inc} + \sum_{j}^{N} \mathbf{E}_j \tag{5}$$

where $\mathbf{E}_i$ denotes the electric field at the position $\boldsymbol{r}_i$, $\mathbf{E}_{i,inc}$ is the electric field from the incident beam and $\mathbf{E}_j$ is the contribution from the jth dipole located at $\boldsymbol{r}_j$ and can be formulated as

$$\mathbf{E}_j = -\frac{e^{ikr_{i,j}}}{r_{i,j}{}^3}\left\{k^2\boldsymbol{r}_{i,j} \times \left(\boldsymbol{r}_{i,j} \times \mathbf{P}_j\right) + \left(\frac{1 - ikr_{i,j}}{r_{i,j}{}^2}\right) \times \left[r_{i,j}{}^2\mathbf{P}_j - 3\boldsymbol{r}_{i,j}\left(\boldsymbol{r}_{i,j} \cdot \mathbf{P}_j\right)\right]\right\} \tag{6}$$

where $\boldsymbol{r}_{i,j} = \boldsymbol{r}_i - \boldsymbol{r}_j$, k is the wavenumber and $\mathbf{P}_j$ is the jth solved dipole.

$$\mathrm{C}_{ext} = \frac{4\pi\mathrm{k}}{|\mathrm{E}_{inc}|^2}\sum_{j}^{N} \mathrm{Im}\left(\mathbf{E}_{inc,j}{}^* \cdot \mathbf{P}_j\right) \tag{7}$$

where C_{ext} is the extinction cross-section, $\mathrm{Im}(x)$ denotes the imaginary part of x and x^* is the conjugate of x.

$$\mathrm{C}_{abs} = \frac{4\pi\mathrm{k}}{|\mathrm{E}_{inc}|^2}\sum_{j}^{N} \left\{\mathrm{Im}\left[\mathbf{P}_j \cdot \left(\alpha_j{}^{-1}\right)^* \mathbf{P}_j{}^*\right] - \frac{2}{3}\mathrm{k}^3\mathbf{P}_j \cdot \mathbf{P}_j{}^*\right\} \tag{8}$$

where C_{abs} is the absorption cross-section and α_j is the polarizability of the jth dipole. In this paper, we use the "filtered coupled dipole" (FCD) method (*52*, *53*) to calculate the polarizability (Eq. (9)-(11)).

$$\alpha_j = \frac{\alpha_{j,CM}}{1 + D} \tag{9}$$

where $\alpha_{j,CM}$ is the Clausius-Mossotti polarizability (*54*) defined below:

$$\alpha_{j,CM} = \frac{3d^3}{4\pi}\frac{\left(m_j{}^2 - 1\right)}{\left(m_j{}^2 + 2\right)} \tag{10}$$

where m_j is the complex refractive index of the jth dipole and d is the dipole length. The D term in Eq. (9) is defined as:

$$D = \frac{\alpha_{j,CM}}{d^3}\left[\frac{4}{3}(kd)^2 + \frac{2}{3\pi}\ln\left(\frac{\pi - kd}{\pi + kd}\right)(kd)^3 + \frac{2}{3}i(kd)^3\right] \tag{11}$$

where k is the wavenumber of the incident beam.

When considering multiple-metallic nanostructures, the polarizability of the dipoles should be modified accordingly. In PyDScat-GPU, the refractive indexes of individual dipoles were estimated and further used to calculate the polarizability via the FCD method. By default, a simple and empirical relation was used to determine the refractive index depending on the components and their portions:

$$m_j = \sum_k w_{j,k} m_{j,k} \tag{12}$$

where $w_{j,k}$ is the portion of component k in the jth dipole. The polarizability can also be defined by the user, which might be from experimental measurement.

Once C_{ext} and C_{abs} are available, the extinction and absorption efficiency factor can be transformed from the corresponding cross-sections via Eq. (13).

$$Q_{ext/abs} = \frac{C_{ext/abs}}{\pi r_{eff}{}^2} \tag{13}$$

where r_{eff} is the effective radius of a sphere with the same volume as the nanoparticle.

The extinction efficiency factors from several orientations of the simulated nanoparticle are calculated and averaged in the simulation to give the final UV-Vis spectrum. To enable accuracy, the validation criterion (*51*) (Eq. (14)) is always satisfied in our calculations.

$$|m|kd < 0.5 \tag{14}$$

where m is the complex refractive index of the material, k is the wavenumber of the incident beam and d is the dipole length.

2.2.2. Results

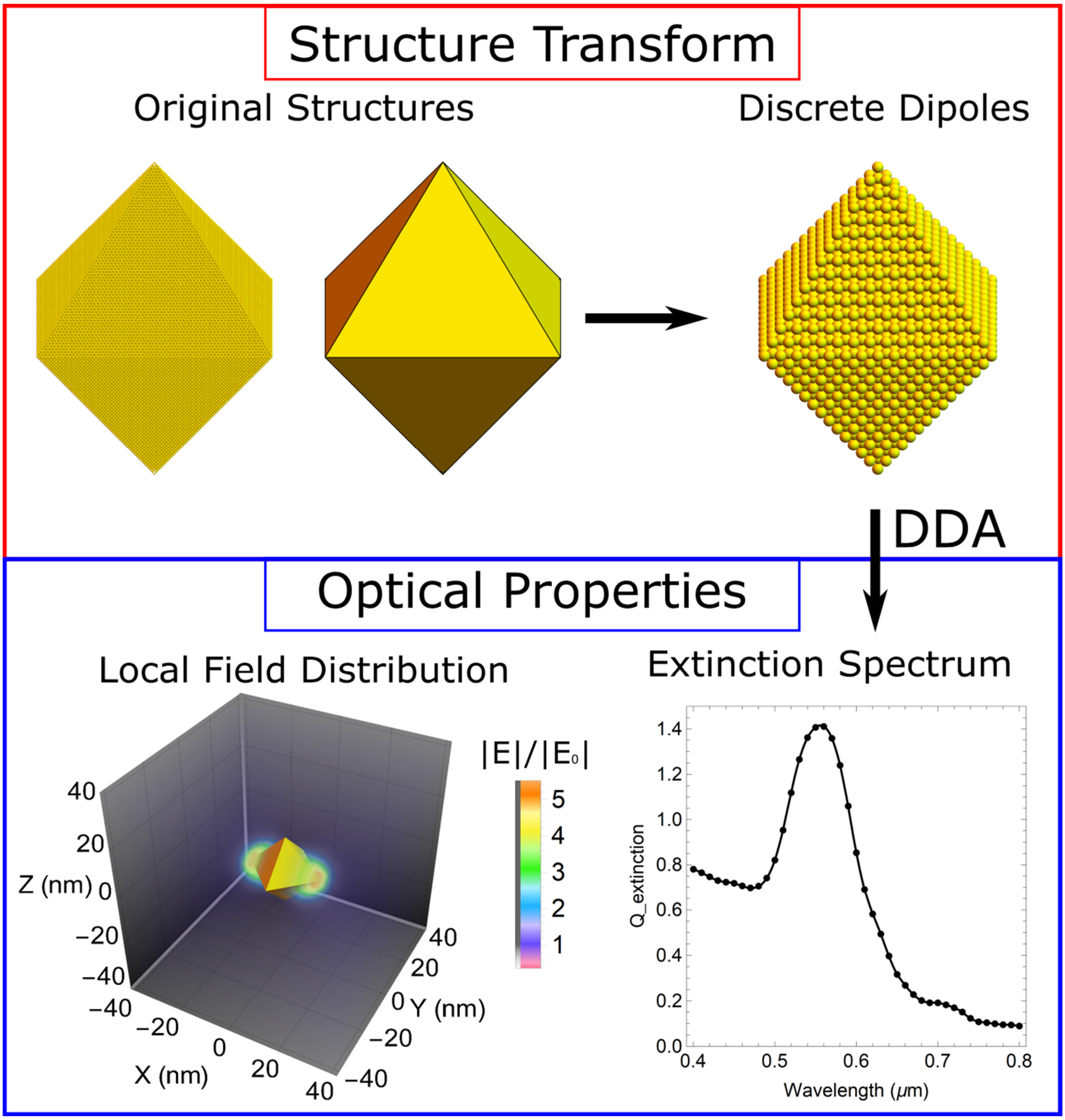

Figure S13. **A typical procedure to simulate the optical properties of nanoparticles.** The original atomic model or the continuum geometry is approximated by a set of dipoles and after solving the dipoles, the spectroscopic properties and local electric field distribution are further evaluated. Here we used Au octahedron (edge length ≈ 20 nm) as an example with its simulated spectrum. Its local electric field distribution at 560 nm is shown. For local electric field simulation, the incident direction is from +Z to -Z and the polarization direction is from -X to +X. Continuum geometry was used to generate the dipoles with a length of 1.0 nm.

An example of simulating the optical properties using PyDSCAT-GPU for Au octahedron (edge length ≈ 20 nm) can be seen in **Figure S13**. Either an atomic model or a continuum geometry can be initially transformed into a set of dipoles for further calculations. To validate our package, we further compared the extinction efficiency factors from it with those from DDSCAT on the same Au

nanoparticles including spheres (radius ≈ 10 nm), octahedra (edge length ≈ 20 nm) and both transverse and longitudinal modes of rods (10×10×30 nm cuboid). They all showed consistent results with neglectable differences (**Figure S14**).

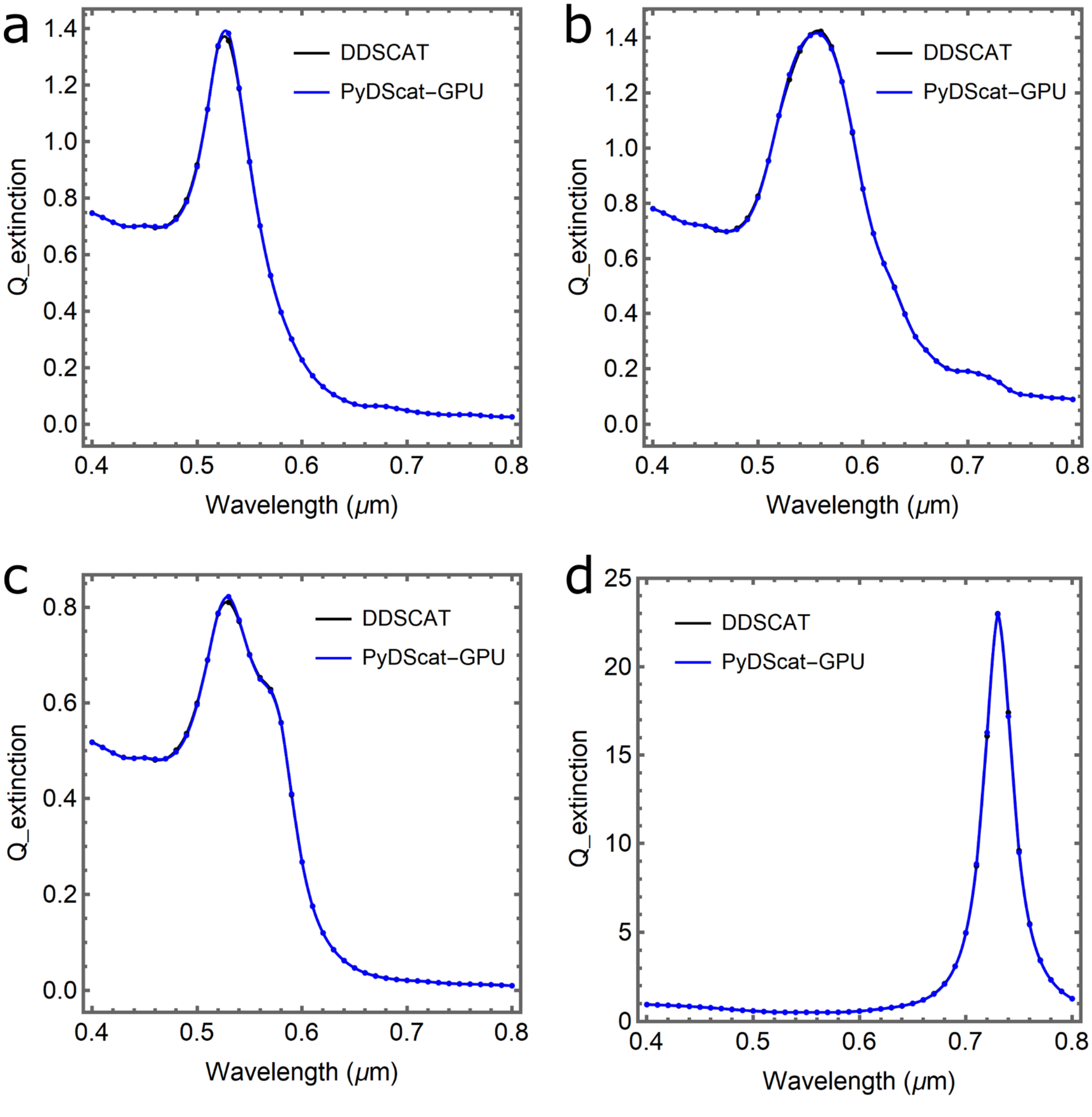

Figure S14. The comparison between DDSCAT and PyDScat-GPU. The extinction efficiency factors were calculated with these two methods (DDSCAT: black line; PyDScat-GPU: blue line) for different Au nanostructures including spheres (a), octahedra (b), transverse (c) and longitudinal (d) mode of rods. The raw data points are shown in dots with a range of [0.4, 0.8] μm with an interval of 0.01 μm and cubic spline interpolation was applied to smooth the curve. All the dipole sets were generated from continuum geometries.

Regarding the multiple-metallic nanostructures, another two examples are shown in **Figure S15**. In both examples, a bimetallic octahedron (edge length ≈ 20 nm) consisting of Au and Ag atoms was created.

In the first example, it is assumed the doping of Ag in the Au nanoparticle was uniform so that all the dipoles had the same portions of Au and Ag, thus sharing the same refractive index and polarizability. In the simulation, the peak blue-shifted with an increased portion of Ag (**Figure S15a**).

In the second example, we simulated the UV-Vis spectra for a series of Au@Ag core-shell structures, where the dipoles belonging to the core were composed purely of Au and the shell purely of Ag. The shape of the nanoparticle was an octahedron with an edge length of 20 nm. The core was set to be an octahedron with its edge length decreased gradually from 20 nm to 10 nm, and the simulation showed the emergence of the Ag feature peaks between 400 nm and 500 nm due to the expansion of the Ag shell (**Figure S15b**).

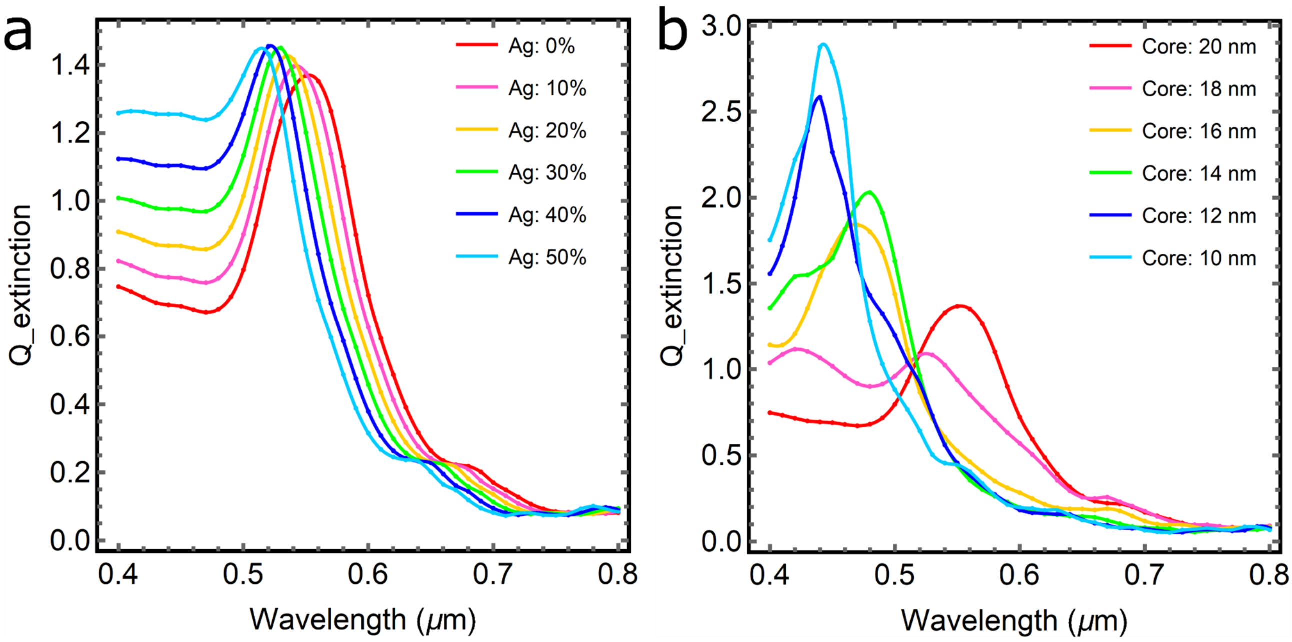

Figure S15. The simulated UV-Vis spectra of bimetallic Au-Ag nanoparticles. (a) The Ag portion of the Au-Ag octahedron (edge length ≈ 20 nm) is increased from 0% to 50%, with a blue-shifted peak in the simulated spectra resulting from the polarizability change. (b) The Au core size is decreased from 20 nm to 10 nm as the thickness of the Ag shell increases. The original peak corresponding to the Au feature disappears, and new Ag feature peaks appear during this process.

2.3. Au/Au-Ag bimetallic nanoparticles using superellipsoid as the shape descriptor and their spectroscopic properties

Before creating the simulated chemical space, we created a geometry set consisting of a variety of shapes originating from the superellipsoid, which can be defined by Eq. (15).

$$\left(\left|\frac{x}{a}\right|^{r}+\left|\frac{y}{b}\right|^{r}\right)^{t/r}+\left|\frac{z}{c}\right|^{t}\leq 1 \tag{15}$$

where (a, b, c) and (r, t) tunes size and the curvature of the superellipsoid respectively.

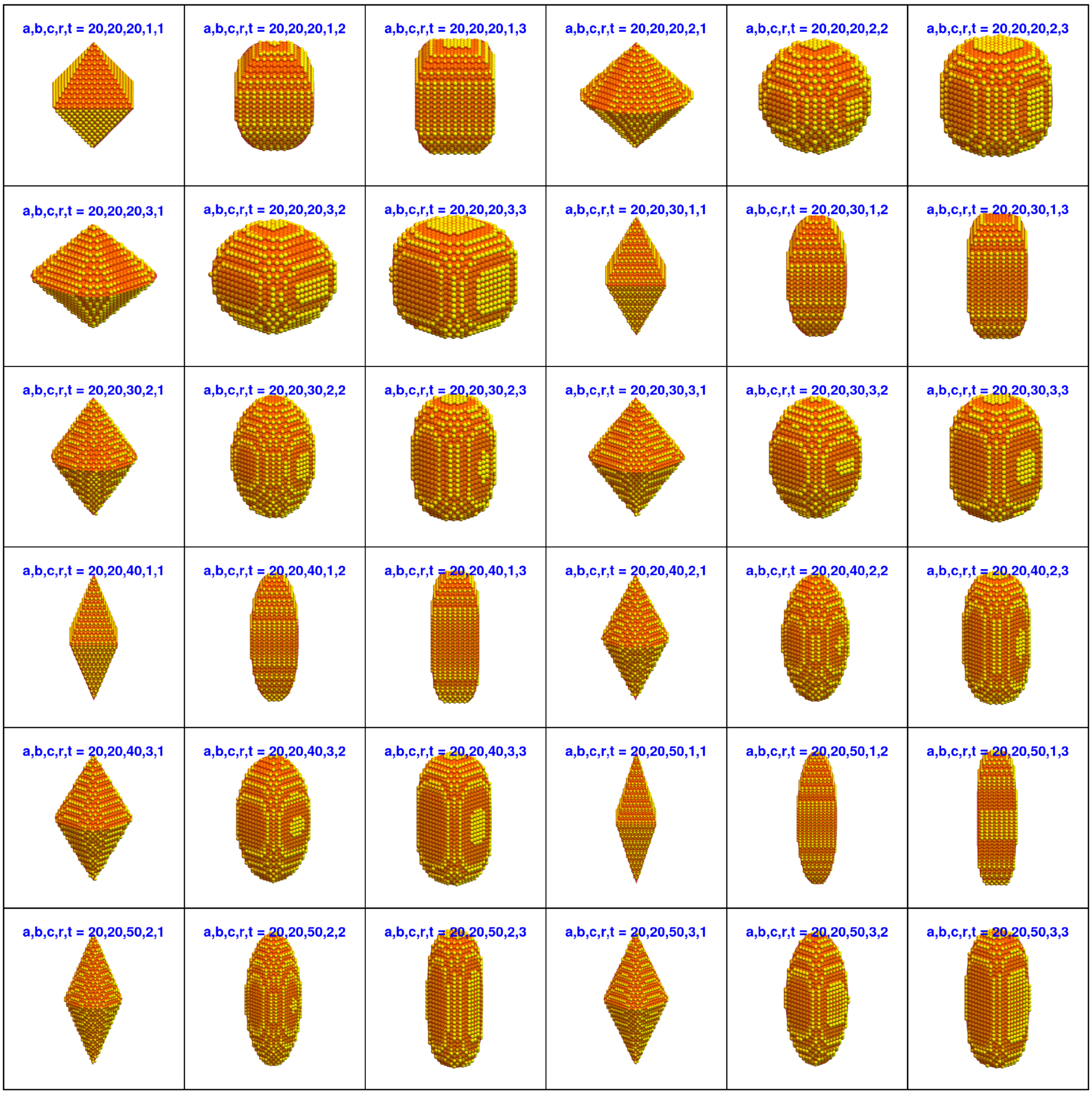

Figure S16. Examples of the shapes originated from superellipsoid by tuning the parameter set $(\boldsymbol{a}, \boldsymbol{b}, \boldsymbol{c}, \boldsymbol{r}, \boldsymbol{t})$. The corresponding parameters are labelled with the individual shape, together with the dipoles (yellow dots) and contour (red) in this figure. c was used to elongate the shape and (r, t) can change the curvature gradually from smooth to sharp. For every shape, a set of dipoles was created for further DDA simulation.

In our simulation, we set $a = b = 20$ nm and varied c in the range of [20, 60] nm with a constant interval of 5 nm to introduce anisotropy. The curvature of the shape was tuned by changing both r and t in the range of [1,3] with an interval of 0.2. By changing the parameter set (c, r, t) and keeping $a = b = 20$ nm in Eq. (15), we created a 9×11×11 geometry set including spheres, octahedra, rods, bipyramids, etc., (See **Figure S16** for examples). The dipoles were all approximated from the continuum geometries. Then the corresponding extinction spectra of nanoparticles with the same shapes can be simulated with PyDScat-GPU as described in **Section 2.2**. Note geometric error in

representing the original shape with dipoles could be introduced when converting a continuum geometry into the dipoles.

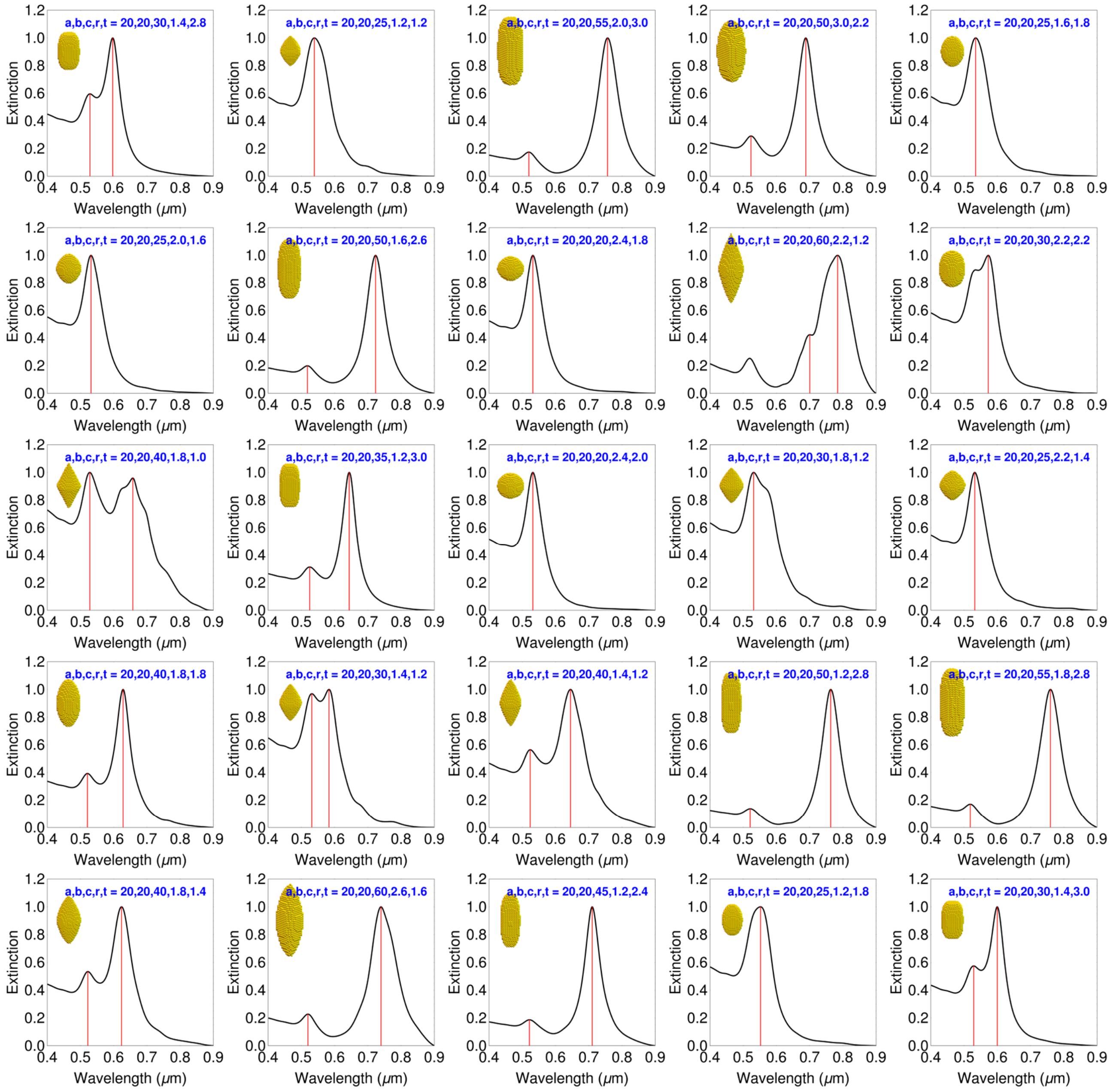

Figure S17. Examples of the shapes of the set of Au nanoparticles and the corresponding UV-Vis spectra in simulated chemical space 1. The dipoles are purely composed of Au. The corresponding parameter set (a, b, c, r, t) for the shape is labelled in the figure. The dipole size was selected and enabled to satisfy Eq. (14) and spectra in multiple orientations were calculated and averaged for the final UV-Vis spectrum. The wavelength range is [0.4, 0.9] μm with an interval of 0.01 μm and cubic spline interpolation was applied to smooth the curve. The extinction was normalized to the range of [0,1].

For the first simulated chemical space, the dipole component is purely Au. Thus, a 9×11×11 set of Au nanoparticles originating from the geometry set can be created and will be used to define the first simulated chemical space (See **Figure S17** for examples and their corresponding UV-Vis spectra).

For the second simulated chemical space, the dipole is composed of both Au and Ag, where the portions of one dipole depend on a dipole component function (DCF). Thus, a set of nanoparticles with not only different shapes but also compositions can be created. For a single dipole in the nanoparticle with its geometry defined by the parameter set (a, b, c, r, t), we first calculate its "relative distance" d_R via Eq. (16), which indicates the relative position of the dipole to the centre:

$$d_R = \left(\left|\frac{\mathrm{x}}{a}\right|^r + \left|\frac{\mathrm{y}}{b}\right|^r\right)^{t/r} + \left|\frac{\mathrm{z}}{c}\right|^t \tag{16}$$

where $(\mathrm{x}, \mathrm{y}, \mathrm{z})$ is the dipole position.

The Ag and Ag portions in the dipole are determined by the relative distance (d_R) through Eq. (17) and (18).

$$p_{Ag} = \frac{1}{1 + e^{-v_{DCF,1}(d_R - v_{DCF,2})}} \tag{17}$$

$$p_{Au} = 1 - p_{Ag} \tag{18}$$

where $v_{DCF,1}$ and $v_{DCF,2}$ are changeable coefficients. These equations defined a higher portion of Ag in the outer layer of nanostructures.

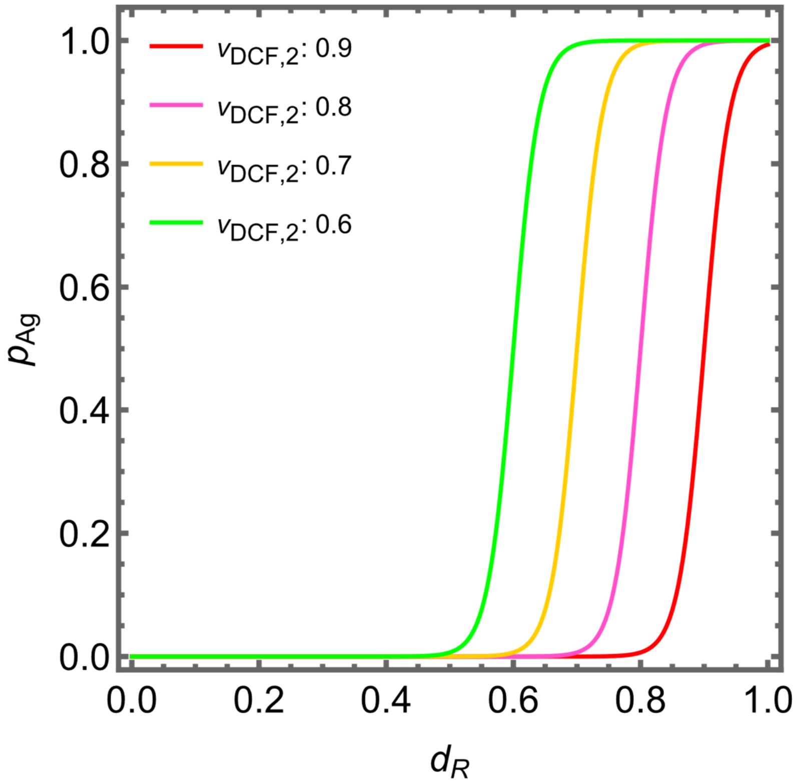

Figure S18. The portion of Ag (p_{Ag}) as a function of the relative distance (d_R). Different curves were drawn by setting $v_{DCF,1}$ as 50 and changing $v_{DFC,2}$ from 0.9 to 0.6 with an interval of 0.1. With the decreased value of $v_{DCF,2}$, the portions of Ag in the outside dipoles are increased.

By keeping $v_{DCF,1}$ as 50 and varying $v_{DCF,2}$ from 0.9 to 0.6 with an interval of 0.1, the distribution of Ag in the out layer in the nanostructures was changed (**Figure S18**). Thus, a 9×11×11×4 set of Au-Ag bimetallic nanostructures was created with all the shapes originating from the geometry set but distinct $v_{DCF,2}$. Their extinction spectra were further simulated to create the second simulated chemical space, which will be discussed below (See **Figure S19** for examples and their corresponding UV-Vis spectra).

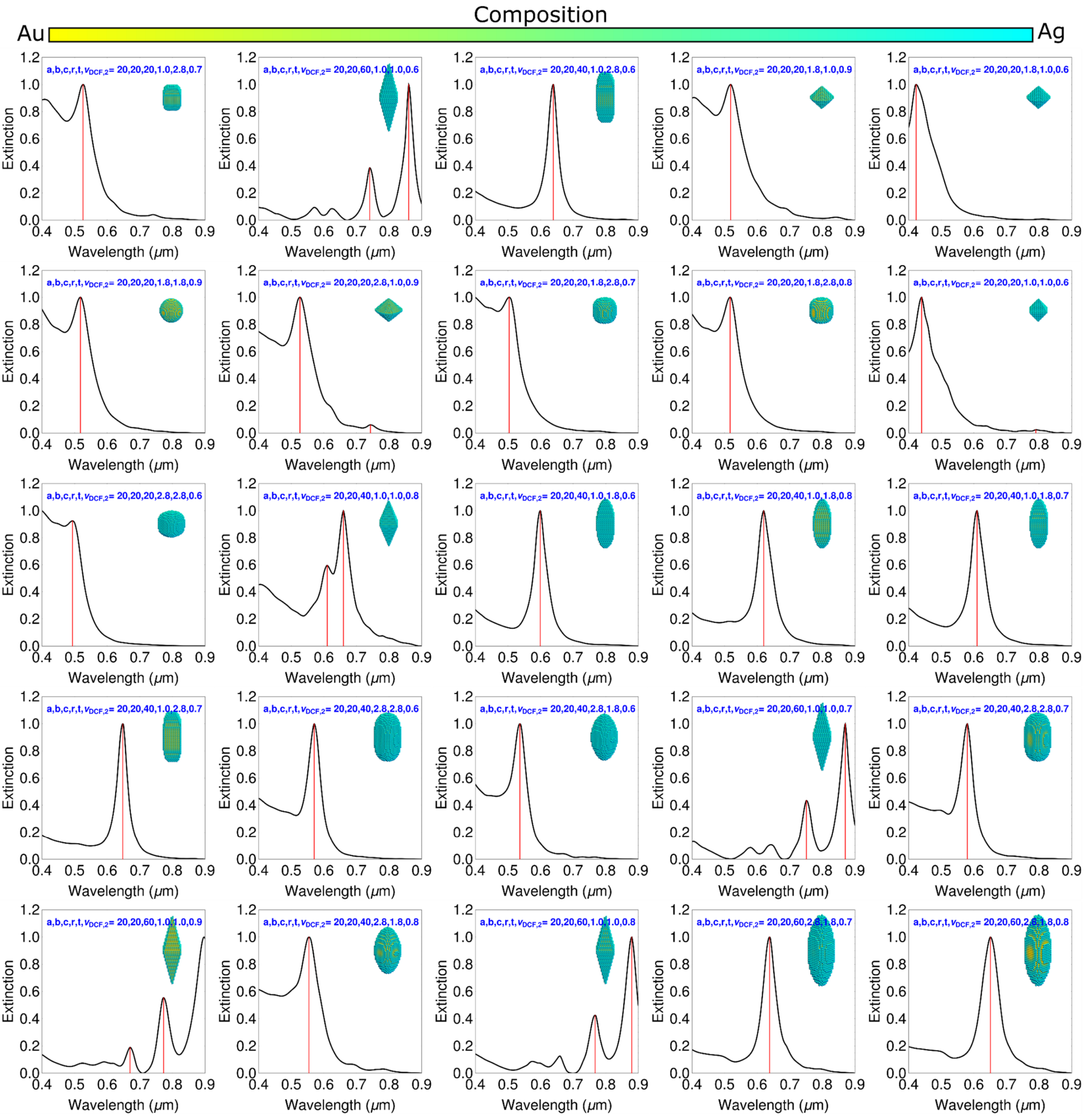

Figure S19. Examples of the shapes of the set of Au-Ag nanoparticles and the corresponding UV-Vis spectra in simulated chemical space 2. The dipoles are composed of Au and Ag with their portions in the dipole indicated by the colour. The corresponding parameter set $(a, b, c, r, t, v_{DCF,2})$ for the shape is labelled in the figure. The dipole size was selected to satisfy Eq. (14) and several orientations were calculated and averaged for the final UV-Vis spectrum. The sampling range is [0.4,

0.9] μm with an interval of 0.01 μm and cubic spline interpolation was applied to smooth the curve. The extinction was normalized to the range of [0,1].

2.4. Simulated chemical space from uniquely-shaped nanoparticles

2.4.1. Simulated chemical space 1

The first chemical space was created utilising the 9×11×11 Au nanoparticle set, which is generated by varying the superellipsoid parameters (c, r, t) as described in **Section 2.3**. The input variables in the chemical space include three variables (v_1, v_2, v_3) that can change the values of (c, r, t), thus varying the shapes of the nanoparticles. A fourth variable v_4 was used to introduce the Au octahedra as by-products. All the variables of (v_1, v_2, v_3, v_4) are in the ranges from 0 to 1.

Since the Au nanoparticle set is discrete and originates from different geometries with values of (c, r, t), a linear transformation and a piece-wise rounding function were used to map the first three input variables (v_1, v_2, v_3) to the geometry parameters (c, r, t) as follows:

First, each of the (c, r, t) variables was sorted in ascending order. For the sampling point in the input space with (v_1, v_2, v_3), we need to determine which (c, r, t) values this point corresponds to. The ith value of c, j^{th} value of r and k^{th} value of t were chosen according to the values of (v_1, v_2, v_3), with the indexes being determined by Eq. (19), (20) and (21) respectively.

$$i = 1 + \lceil 8v_1 \rfloor \tag{19}$$

$$j = 1 + \lceil 10v_2 \rfloor \tag{20}$$

$$k = 1 + \lceil 10v_3 \rfloor \tag{21}$$

where $\lceil x \rfloor$ outputs the nearest integer to x.

For a given point (v_1, v_2, v_3, v_4), the sample is composed of $(1 - v_4)$ amount of the Au nanoparticles with their geometry determined by (v_1, v_2, v_3) as described above, together with v_4 amount of Au octahedra with an edge length of 20 nm as by-products. To obtain the extinction spectrum of the sample, the original extinction spectra of the nanostructure and the by-products were simulated, and their weighted summation was used as the final spectrum of the sample. This final spectrum was normalized to the range of 0 to 1 for further data processing.

2.4.2. Simulated chemical space 2

In the second simulated chemical space, an extra dimension to control the doping of Ag into Au nanostructures was added as described in **Section 2.3**. The input variables were defined as $(v_1, v_2, v_3, v_4, v_5)$ with (v_1, v_2, v_3) determining the geometry in the same way as that in the first

simulated chemical space by Eq. (19), (20) and (21). v_4 modified the distribution of Ag in nanostructures by selecting the $v_{DFC,2}$ for the dipole component function (Eq. (16), (17) and (18)). Since there are four values of $v_{DFC,2}$ in creating the set of Au-Ag bimetallic nanoparticles, the hth value of $v_{DFC,2}$ was used for a given v_4 through Eq. (22). The discrete values of $v_{DFC,2}$ were sorted in descending order from 0.9 to 0.6 first.

$$h = 1 + \lfloor 3v_4 \rfloor \tag{22}$$

The effect of by-products was also introduced via v_5 through the same way as that in the first simulated chemical space. For a given $(v_1, v_2, v_3, v_4, v_5)$, the sample is composed of $(1 - v_5)$ amount of the Au-Ag bimetallic nanostructures depending on (v_1, v_2, v_3, v_4) as described above, together with v_5 amount of Au-Ag bimetallic octahedra with an edge of 20 nm and $v_{DCF,2} = 0.9$ as the by-products.

2.5. Exploration in the simulated chemical space

The exploration algorithm was tested and benchmarked with Random Search on both simulated chemical spaces. Compared to Random Search, the exploration algorithm based on MAP-Elites showed a better performance in both exploring the chemical space to find diversified samples and optimise the performance of individual elite.

2.5.1. Method

The behaviour space to classify samples was based on the position of the most prominent peak. First, the wavelength range of [0.4, 0.9] μm was discretized into 10 subregions, with a region width of 0.05 μm. Then for a given sampling point of (v_1, v_2, v_3, v_4) (for simulated chemical space 1) or $(v_1, v_2, v_3, v_4, v_5)$ (for simulated chemical space 2), the corresponding extinction spectrum was simulated to give the peak prominences and positions.

In searching the peaks, the lowest threshold for peak prominence was set to 0.01. If the total peak number was lower than 2, the sample was no further processed and discarded. The class index of these discarded samples was set as 0. This class was not considered during the exploration process and was not included in the analysis of the benchmark results. Depending on which subregion the most prominent peak is located in, a class index was assigned to the sample (from 1 to 10 with increased wavelength). The fitness of the sample was further calculated through the percentage of the extinction area within w range near the most prominent peak, which enabled the search to get rid of other peaks except for the most prominent one (Eq. (23)).

$$F = \frac{\int_{x_{peak}-w}^{x_{peak}+w} I_x dx}{\int I_x dx} \tag{23}$$

where F is the fitness function, I_x is the absorption of the normalized UV-Vis spectrum at wavelength x, x_{peak} is the position of the most prominent peak, and w is a parameter to define the region near the peak. Here we set w to 0.05 μm, which has a similar range to the absorption area of one peak.

An absorption boundary condition in the input space was defined so that if the input variable is smaller or larger than the lower or upper boundary, the variable will be replaced by the lower or upper boundary. This boundary condition was used in all the tests. The initial sampling number was 10. The batch size for one step was 23 for both the exploration algorithm and Random Search, which is consistent with the batch size that will be used in the actual nanoparticle synthesis. In the exploration algorithm, among these 23 samples, 10 samples were mutated from the parent set, 10 samples were from crossover among parents with a further 40% chance of mutation and 3 samples were randomly generated in the input chemical space. This setting distributed resources equally to mutation and crossover, with a small portion of random sampling to avoid being trapped locally. In the mutation process, we sampled a vector from a multi-Gaussian distribution with a mean of 0 and the same standard deviation for all dimensions and then added this vector to the original sampling point. The standard deviations were 0.08 and 0.15 in the first and second simulated chemical space, respectively. The standard deviation is higher in the second chemical space due to its increased dimensionality and complexity. The estimated upper boundaries for both the mean fitness among all the elites and elite number were from a grid search with a 0.05 interval of mixture rate (v_4 for simulated chemical space 1 and v_5 for simulated chemical space 2) from 0 to 1 for all the shapes and compositions in the nanoparticle set.

2.5.2. Class distribution and interconnectivity in the simulated spaces

In both spaces, for a given mixture rate, the input space was discretized because of the round functions in Eq. (19)-Eq. (22). Varying the input parameters within the discretized regions does not change the output spectrum. By assigning each region with a class index based on its spectrum (**Section 2.5.1**) and summing the volume of the regions belonging to the same class, the phase volumes of the classes in the input space can be calculated. It should also be noted that the volumes of the regions at the input boundaries of 0 and 1 are smaller considering the definition of the round functions (Eq. (19)-Eq. (22)).

The interconnectivity among classes can be estimated by the interconnectivity of the discrete regions. For example, to estimate the interconnectivity between class X and Y, we can obtain all the discrete regions that belong to class X and their neighbouring regions. The contact area of the neighbouring regions that belong to class Y was a good estimation of the interconnectivity between class X and Y.

However, the chemical space is not discrete in the dimension of the mixture rate, and no discrete regions can be defined in this dimension. To estimate the volume in the input space and the interconnectivity of different classes, we sampled discretely in the dimension of the mixture rate with an interval of 0.05 from 0 to 1. Consequently, the discrete regions for both simulated spaces were created respectively. By summating the volumes of the regions that belong to the same class, we estimated the phase volumes of different classes in the input space. By calculating the contact area from the neighbouring regions as described above, we estimated the interconnectivity among classes (see **Figure S20** and **Figure S21** for simulated chemical space 1 and 2 respectively).

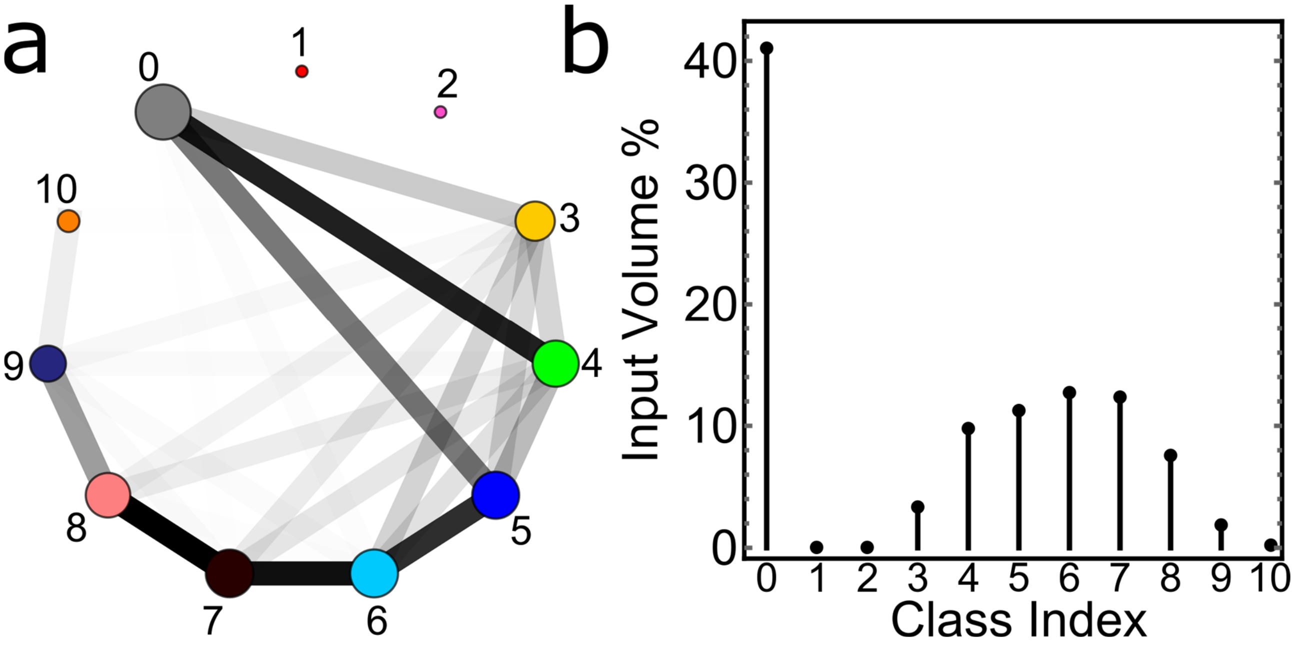

Figure S20. The analysis of simulated chemical space 1. (a) The pictorial representation of the class and their interconnectivity in simulated chemical space 1. The sizes of the nodes indicate the phase volumes of the classes, and the thicknesses of the edges indicate the interconnectivity. Class 0 indicates the samples with a peak number smaller than 2. (b) The estimated percentages of the volumes in the input space for different classes. They are 41.04%, 0%, 0%, 3.26%, 9.78%, 11.21%, 12.77%, 12.31%, 7.50%, 1.93% and 0.20% from class 0 to class 10 respectively. Note no sample belonging to class 1 and 2 in the first chemical space, indicating no double-peak system can have the most prominent peak in the range of [0.4, 0.5] μm.

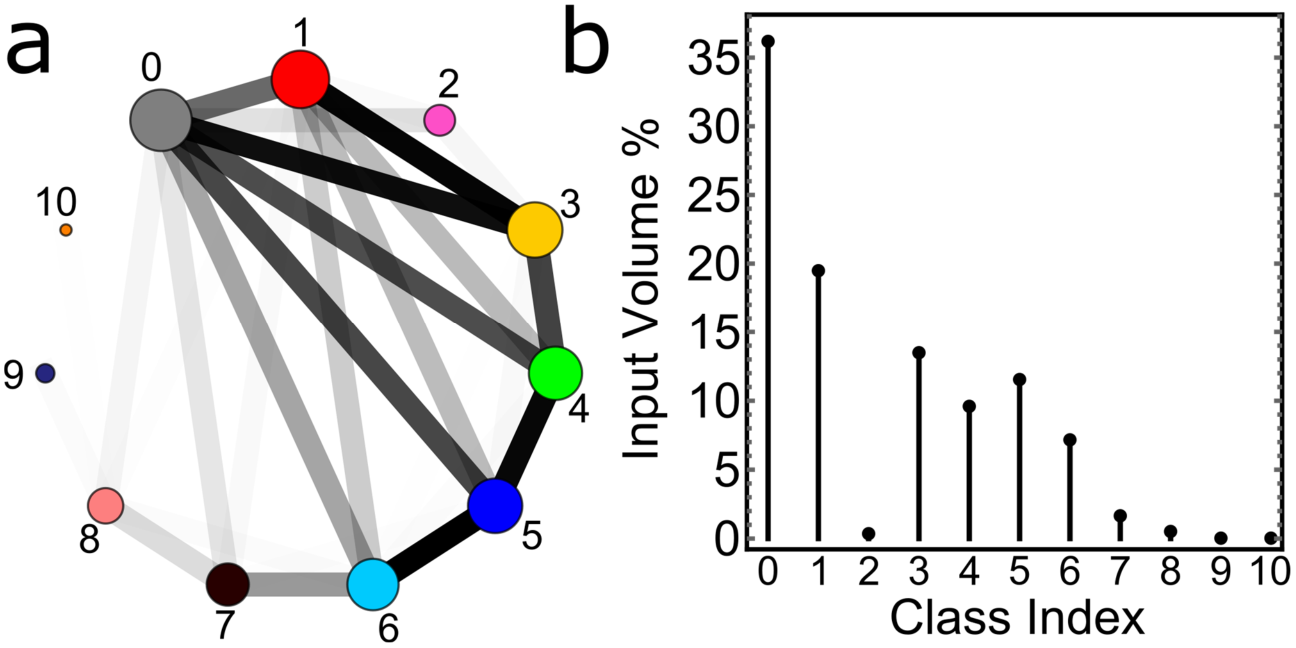

Figure S21. The analysis of the simulated chemical space 2. (a) The pictorial representation of the class and their interconnectivity in simulated chemical space 2. The sizes of the nodes indicate the phase volumes of the classes, and the thicknesses of the edges indicate the interconnectivity. Class 0 indicates the samples with a peak number smaller than 2. (b) The estimated percentages of the volumes in the input space for different classes. They are 36.18%, 19.42%, 0.28%, 13.49%, 9.68%, 11.55%, 7.12%, 1.66%, 0.56%, 0.04% and 0.01% from class 0 to class 10 respectively.

2.5.3. Results

We randomly sampled 10 points as the initial data set. Then we further explore simulated chemical space 1 and 2 for another 50 and 200 steps respectively. Since both the exploration strategy based on MAP-Elites and Random Search contain stochastic effects from choosing random variables, the search was repeated 16 times to elaborate their capability in exploring the chemical space. In each repeat, the mean fitness among all the elites and the elite number were measured.

The results from simulated chemical space 1 are shown in **Figure S22** together with their standard deviation for every step. The upper boundaries are estimated from the grid search. The mean fitness is defined by averaging the fitness values of different elites. In looking for elites belonging to different classes, Random Search almost converged after 51 steps and still cannot find all kinds of elites in the space, while with the exploration algorithm, all the possible elites were found after 15 steps. And the average mean fitness of Random Search at the end (0.564 ± 0.012) is lower than both that from the exploration algorithm (0.595 ± 0.003) and the estimated upper boundary (0.603). The exploration algorithm exceeded the final average mean fitness of Random Search within 8 steps and ended up with an average mean fitness of ca. 99% of the estimated upper boundary.

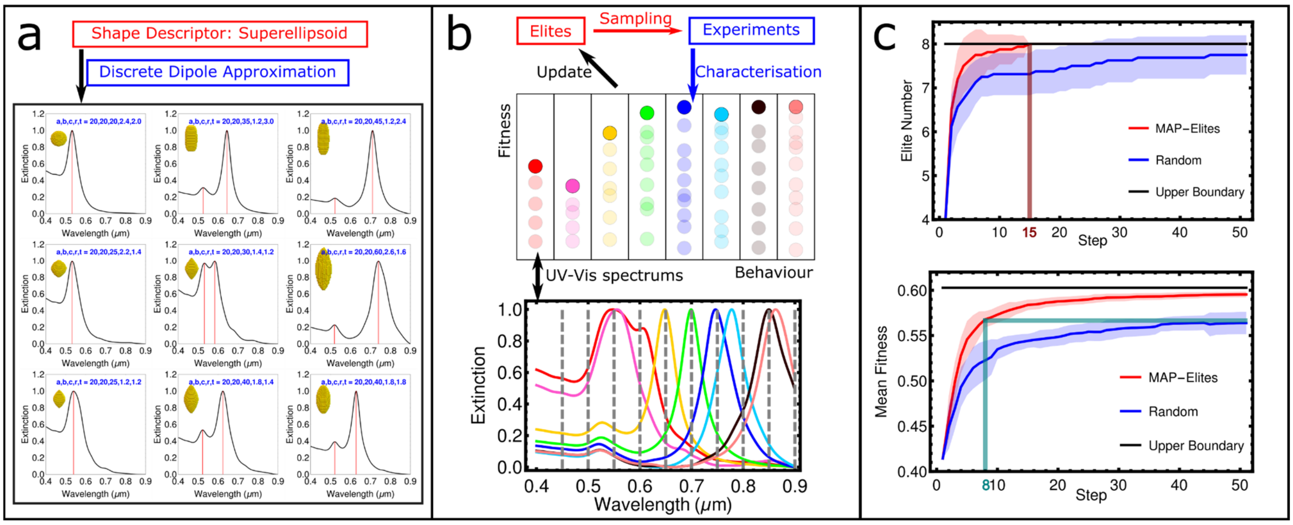

Figure S22. In silico exploration of nanoparticles with exploration algorithm based on MAP-Elites in simulated chemical space 1. (a) A simulated chemical space was based on superellipsoid shape descriptor and simulated extinction spectra. (b) The scheme for exploration. The results were characterised and classified according to their UV-Vis behaviour, and only the elites were selected as parents to generate the next set of samples. (c) The results of exploration with both the exploration algorithm based on MAP-Elites (red) and Random Search (blue) with the estimated upper boundary (black) in the simulated chemical space. The performance of the exploration algorithm after 15 (to find elites) or 8 (to increase fitness) steps is better than the final results of Random Search after 51 steps.

The results from exploring chemical space 2 are shown in **Figure 3** in the manuscript, where we found a set of high-quality and diversified UV-Vis spectra. Since this space is more complicated, after the initial sampling, the number of steps for exploration was increased from 50 to 200. After the exploration, Random Search still cannot find all the elites, with a large standard deviation of 0.5 among the repeats. The exploration algorithm outperformed it after 27 steps and can find all the elites after 78 steps (**Figure 3C top**). After 25 steps, the average mean fitness from the exploration algorithm exceeded the final mean fitness from Random Search (0.593 ± 0.019), and eventually reached 0.661 ± 0.010, which is ≈ 0.014 smaller than the upper boundary (0.675) (**Figure 3C bottom**) and ca. 98% of it.

This test benchmarked the capability of the exploration algorithm in searching the feature space over Random Search, showing it can return a set of high-performance and diversified solutions. When we applied the exploration algorithm in real experiments in an autonomous platform, more modifications were introduced and will be discussed in the following sections.

2.6. Optimisation in the simulated chemical space

After the exploration, fine-tuning the optical properties of the nanoparticles to approach a given target was necessary. Here we implemented the optimisation algorithm based on global search with local sparseness (GS-LS) and utilised the evolutionary algorithm (EA) as the optimiser after the space was explored. With the optimisation algorithm, it is demonstrated that not only the global maximum, but

also multiple optimal solutions of different nanostructures can be found successfully by considering the local sparseness in the simulated space.

2.6.1. Method

The goal of optimisation is to find the sample with the highest fitness but also several other samples with moderate fitness that are separated in the input space. In our experiment, the fitness (F) (Eq. (3)) was a linear summation of the local sparseness term (Eq. (1)) and the similarity metric (M_S) (Eq. (2)). The local sparseness term was used to quantify the local sampling density (Eq. (1)) while the similarity metric measures the absolute spectrum difference as well as the peak position difference of the highest peak (Eq. (2)).

$$S(x) = \frac{1}{K}\sum_{i=1}^{K} dist(x, y_i) \tag{1}$$

where $dist(x, y)$ measures the distance between x and y in the input space, and y_i is the ith closest sample to x.

$$M_S = -|p - p_{target}| - k_1 \sum_i |I_{x,i} - I_{target,i}| + k_2 \tag{2}$$

where p and p_{target} are the peak positions of the highest peak in the UV-Vis spectra of the sample and the target. $I_{x,i}$ and $I_{target,i}$ are the ith intensity of the UV-Vis data of the sample and the target respectively. k_1 is used to tune the importance between constraining the peak position and increasing the overall similarity between the spectra, and was set as 0.2. It should be noted the unit of the wavelength ($|p - p_{target}|$) here is micrometer, thus this term is trivial and optimisation was mainly aimed to find the same target spectrum, which was different from the experimental optimisation later, where we focused more to find samples with the same peak positions. When two identical UV-Vis spectra are found, both $|p - p_{target}|$ and $\sum_i |I_{x,i} - I_{target,i}|$ reduce to 0 and k_2 puts an upper boundary of the similarity metric, which was set as 1 when plotting the fitness function.

$$F = M_S + k_3 S \tag{3}$$

where S is the local sparseness term defined in Eq. (1). k_3 is used to tune the importance of the local sparseness and is varied from 0 to 300 with an interval of 50 considering the scale of S in the benchmark (See below).

The optimisation was conducted in simulated chemical space 2. The same absorption boundary condition in the input space as that in the exploration algorithm was used. Exploration was conducted as described above to see if it was able to find the intended target. For benchmarking, the optimisation started after 11, 21, 31 and 41 steps of the exploration (including the initial random sampling in the

exploration algorithm), and stopped when a complete 201 steps including both exploration and optimisation was reached. The data from exploration were used as the initial dataset for optimisation.

In optimisation, the five samples with the highest fitness (Eq. (3)) from all the available data (including those from previous steps) were selected as the parents for crossover and mutation. Ten unique nearest neighbours (including the sample itself) are used to calculate the local sparseness. The local sparseness term was updated from step to step. There are 23 samples generated per step and among these 23 samples, 10 samples were mutated from the parent set, 10 samples were from crossover among parents with a further 40% chance of mutation and 3 samples were randomly generated in the input chemical space. In the mutation process, a vector from a multi-Gaussian distribution with a mean of 0 and a standard deviation of 0.15 for all dimensions was sampled and then added to the original sampling point. Note all the UV-Vis spectra were normalized before data processing. The upper boundary of the similarity metric is k_2, which is set as 1.

One target spectrum was set for the benchmark. Its parameters to control the nanostructure was listed in **Table S1**. The way that chemical space 2 was defined makes the similarity landscape intrinsically flat (e.g., varying v_1 from 0.6875 to 0.8125 does not change the similarity, because it is $\lceil 8v_1 \rfloor$ that matters). The target spectrum can be achieved by sampling in a region and the variable ranges of this region are also listed in **Table S1**. Note that v_5 defines the mixture rate and is continuous.

**Target**	$\boldsymbol{c}$ **(nm)**	$\boldsymbol{r}$	$\boldsymbol{t}$	$\boldsymbol{v_{DCF,2}}$	$\boldsymbol{v_5}$
**1**	50	2.6	2.6	0.9	0
**Target**	$\boldsymbol{v_1}$	$\boldsymbol{v_2}$	$\boldsymbol{v_3}$	$\boldsymbol{v_4}$	$\boldsymbol{v_5}$
**1**	[0.6875, 0.8125]	[0.75, 0.85]	[0.75, 0.85]	[0, 0.16667]	0

Table S1. The corresponding parameters of (c, r, t) to define the nanostructure for the target spectrum. The input parameter ranges that correspond to the target in the optimisation.

The input difference between the highest-performance sample and the input variable range of the target (**Table S1**) was monitored during the optimisation. From v_1 to v_4, if the value of the sample is within the variable range of the target, the difference is 0. If the input value of the best sample is out of the range, its absolute differences with both the upper and lower boundaries were calculated, and the smaller value was taken as the difference. For v_5, the absolute difference is calculated normally. The difference will be discussed below.

2.6.2. Results

Although exploration based on MAP-Elites can find the elites with optimal absorption peaks, it is not enough to fine-tune the optical properties due to the wavelength window when we defined the subregions (e.g., if we require the target with an absorption peak at 654 nm, the algorithm will treat all the samples with absorption peak from 650 nm to 700 nm as the same class). Furthermore, it distributes the resources to the exploration and parallel optimisation of the multiple elites. Therefore, purely exploration can increase the UV-Vis similarity to the target but will converge after several steps and is unlikely to find the global maximum.

An optimiser that aims to increase the similarity between the sample and the target is necessary if we want to fine-tune the optical properties. However, the UV-Vis spectrum is not a unique characteristic of the nanostructures and multiple different structures can share similar UV-Vis signals. Thus, many local maxima regarding the similarity can coexist in the chemical space, each of which is as valid as the others. By increasing the weight of the local sparseness, the search was encouraged in less-sampled regions further, which helps to avoid being trapped in any single local maximum. However, if the weight of the local sparseness is too high, the algorithm prefers the region with fewer samples and focuses less on increasing the similarity, while increasing the similarity metric is the actual task. The phenomena were observed during the benchmark.

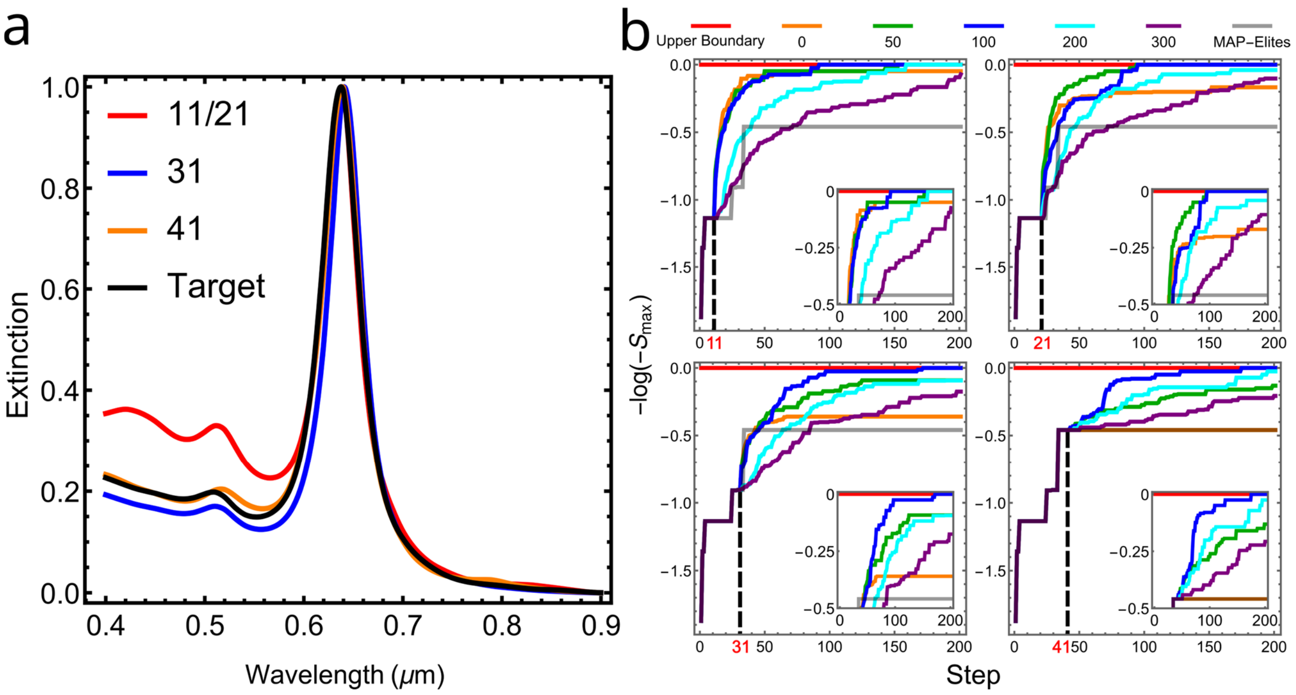

Figure S23. In silico optimisation in simulated chemical space 2. (a) The UV-Vis spectra with the highest similarity to the target after running exploration for 11, 21, 31 and 41 steps, as well as the target spectrum itself. (b) The increase of the similarity with varied coefficients (k_3 in Eq. (3)) of local sparseness and different initial data sets. The step where we started the optimisation process was labelled as red in the figure. The base of the logarithmic scale is 10. Note the results from optimisation

were averaged among 16 parallel repeats. The results from the exploration algorithm based on MAP-Elites are indicated by the grey line.

Figure S23 shows the results of optimisation towards the target. The initial data set for optimisation was created by running exploration for 11, 21, 31, and 41 steps respectively. The total step number including both exploration and optimisation was controlled to be 201. The exploration algorithm was also run for 201 steps as a reference, showing it cannot sufficiently reach the global maximum regarding the similarity metric. All the tests of the optimisation algorithm were repeated 16 times independently with the same initial data set, and the average results of the repeats are shown. **Figure S23a** shows the target and the most similar UV-Vis spectra to it after 11, 21, 31 and 41 steps of exploration. **Figure S23b** shows the increase of the highest similarity metric from the samples found by the optimisation algorithm with varied sparseness coefficient k_3 and initial data set during the optimisation. When the k_3 was set as 0, the local sparseness did not functionalize at all, and the algorithm became GA. The solutions were trapped in the local maximum due to the complexity of the search space and the bias from the initial data set. With more data points from exploration, the final similarity after optimisation was decreased because more local maxima acted as the parents and tended to dampen the search. When k_3 was set as 50, the local sparseness term encouraged the global search. Thus, the efficiency of finding global maximum can be increased (see the case with starting step of 21). But it is still possible to be trapped in the local maxima during the optimisation, which damps the overall efficiency (see the case with starting step of 11) or forbids the convergence to the global maximum (see the cases with starting step of 31 or 41). When k_3 was set as 100, the optimisation algorithm can always find the global maximum efficiently. When k_3 was further increased to 200 or 300, it tended to explore the unsampled region more, so the increase of the similarity metric is slower. The average absolute differences (among 16 repeats) for the five variables in the input space during the optimisation are shown in **Figure S24**.

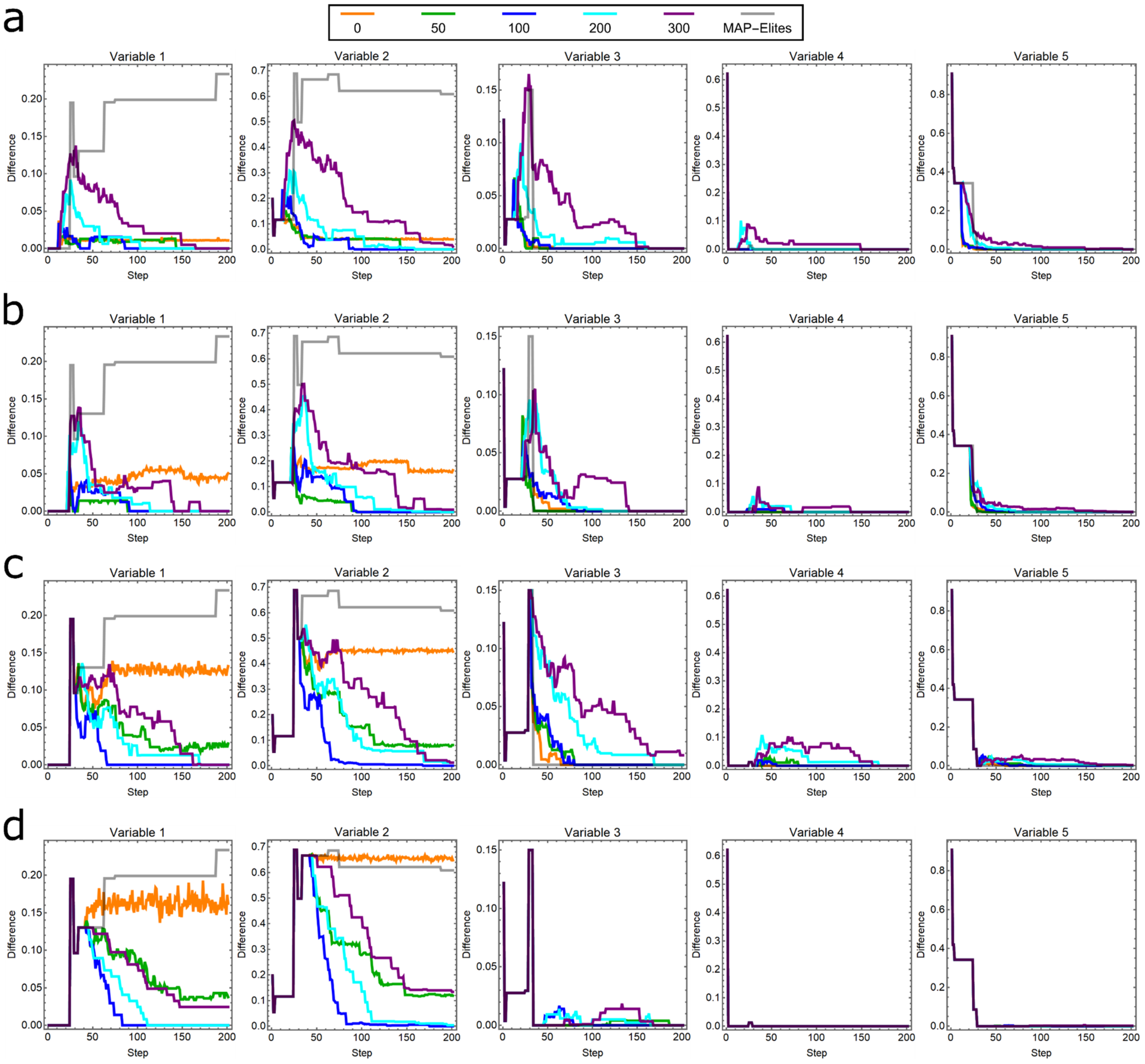

Figure S24. The difference in the input space defined by $(v_1, v_2, v_3, v_4, v_5)$ between the best solution and the target. The optimisation algorithm with varied k_3 was initialised after running exploration for 11 (a), 21 (b), 31 (c) and 41(d) steps. The differences were calculated as discussed above and averaged among 16 parallel repeats. The differences from the exploration algorithm based on MAP-Elites are indicated by the grey line.

Another important purpose of the optimisation algorithm is to find multiple solutions with similar UV-Vis but are separated in the input space $(v_1, v_2, v_3, v_4, v_5)$, which represents the local maxima in the similarity landscape. Here we will analyse the result from one repeat where the starting step of optimisation was 11 and $k_3 = 100$.

Because the implementation of the round function ($\lceil v_i \rfloor$, where v_i is an input space variable for i from 1 to 4), the chemical space is intrinsically flat, i.e., samples within a vicinity in the input space can have the same nanostructure and mixture rate, so that the same similarity metric. This feature created multiple local maximum regions instead of points in the input space. To check the structural diversity

of the samples from the optimisation, sampling points corresponding to the same nanostructure and mixture rate from the same vicinity were removed. The K-nearest neighbour criterion was used to filter solutions that are close to the same local maximum. After that, sampling points were selected as the solutions so that the solution's similarity metric is the highest among its ten nearest neighbours in the input space.

Two questions will be discussed below:

1. Are these local maximum regions sampled by the optimisation strategy?
2. Does the K-nearest neighbour filtering strategy increase the structural diversity in the solutions?

Since the first four variables in the input space (v_1, v_2, v_3, v_4) controls the nanostructure according to Eq. (19) to (22), the nanostructure parameter space can be defined as $(c, r, t, v_{DCF,2}, v_5)$, where v_5 still represents the mixture rate. The local maxima in this parameter space correspond to the local maximum regions in the input space. To find the local maxima, a grid search was conducted in all the possible combinations of $(c, r, t, v_{DCF,2})$ as well as v_5 in the range from 0 to 1 with an interval of 0.05. The following analysis will be in the parameter space of $(c, r, t, v_{DCF,2}, v_5)$.

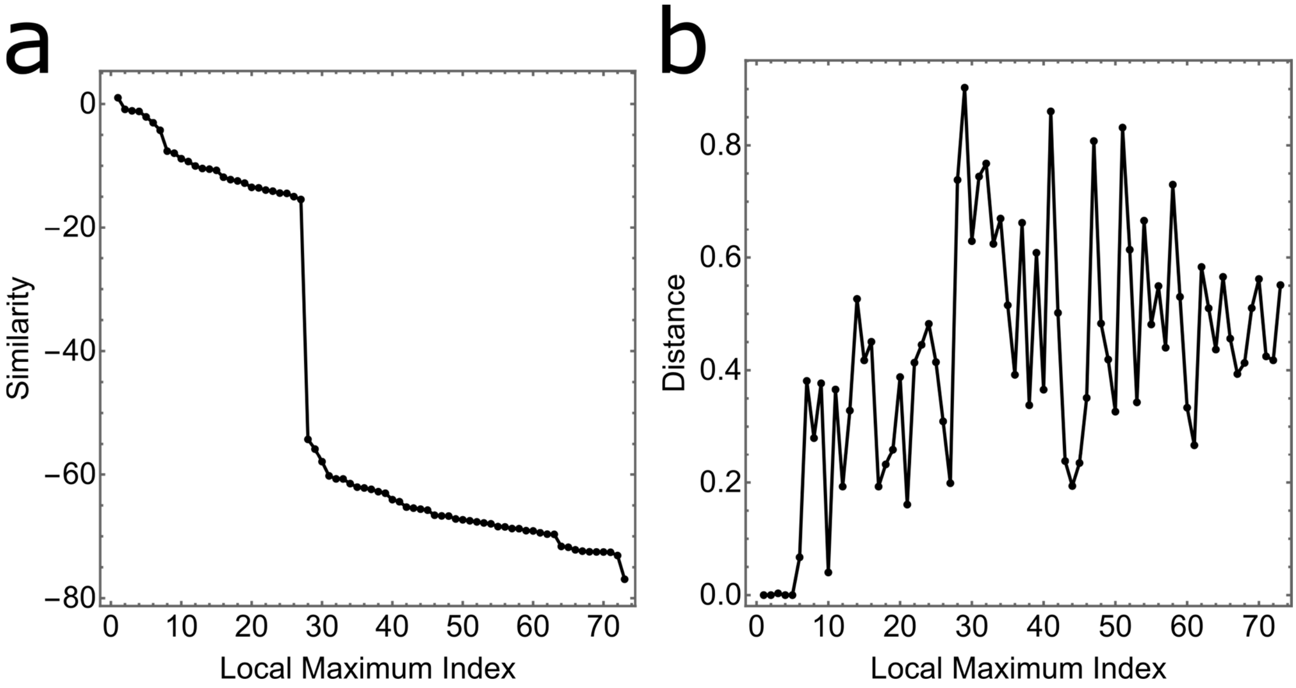

Figure S25. The distribution of the similarity metrics of the local maxima, and their distance to the closest solutions from optimisation. (a) The similarity metrics of the local maxima. They are sorted in descending order regarding their similarity metrics. (b) The smallest distance between the solutions and the local maxima calculated in the parameter space of $(c, r, t, v_{DCF,2}, v_5)$. In calculating the distance, every dimension was normalized from 0 to 1 by a linear transformation.

Multiple local maxima (73 in total) exist in this simulated space. The optimisation was guided by the similarity metric so that local maxima with higher similarity are more likely to be found. The distance between the local maximum and its closest solution (structure) indicates how well this local maximum is optimised. The local maxima were sorted in a descending order regarding their similarity metrics (**Figure S25a**), and the increased distances of these local maxima to the closest samples are shown in **Figure S25b**. These results indicated the preference for searching local maxima with higher similarities during the optimisation. In calculating the distance, to make the scale consistent, every dimension in the space of $(c, r, t, v_{DCF,2}, v_5)$ was normalized from 0 to 1 by a linear transformation.

The top five local maxima with the highest similarity metrics are shown in **Table S2**. To check if the optimisation algorithm found these local maxima, the solutions that are closest to them are shown in **Table S3**, with the neglectable difference from the local maxima (only a difference of 0.030 of v_5 in solution 4).

**Local maximum**	c **(nm)**	r	t	$v_{DCF,2}$	v_5
**1**	50	2.6	2.6	0.9	0
**2**	40	1.2	2.6	0.9	0
**3**	40	1.0	2.2	0.8	0.1
**4**	45	1.6	2.6	0.9	0
**5**	45	1.4	2.2	0.9	0
**Local maximum**	v_1	v_2	v_3	v_4	v_5
**1**	[0.6875, 0.8125]	[0.75, 0.85]	[0.75, 0.85]	[0, 0.16667]	0.0
**2**	[0.4375, 0.5625]	[0.05, 0.15]	[0.75, 0.85]	[0, 0.16667]	0.0
**3**	[0.4375, 0.5625]	[0.00, 0.05]	[0.55, 0.65]	[0.16667, 0.50]	0.1
**4**	[0.5625, 0.6875]	[0.25, 0.35]	[0.75, 0.85]	[0, 0.16667]	0.0
**5**	[0.5625, 0.6875]	[0.15, 0.25]	[0.55, 0.65]	[0, 0.16667]	0.0

Table S2. The local maxima in the nanostructure parameter space and their corresponding local maximum regions in the input space.

**Solution**	c **(nm)**	r	t	$v_{DCF,2}$	v_5
**1**	50	2.6	2.6	0.9	0.0000
**2**	40	1.2	2.6	0.9	0.0000
**3**	40	1.0	2.2	0.8	0.1030

**4**	45	1.6	2.6	0.9	0.0000
**5**	45	1.4	2.2	0.9	0.0000

Table S3. The parameters of the nanostructures from the solutions closest to the five local maxima. Other parameters to control the nanostructure are constant: $a = b = 20$ nm and $v_{DCF,1} = 50$.

To visualize the top five local maxima and their corresponding solutions in the high-dimensional space of $(c, r, t, v_{DCF,2}, v_5)$, a Gaussian process (GP) was trained with the data from optimisation. For every local maximum, the similarity distributions on the planes that are composed of any of the two dimensions and pass the local maximum are shown in **Figure S26**. The local maxima are indicated by the red points. Note the range of the similarity metrics was normalized from 0 to 1 by a linear transformation. The nearest solution to the local maximum was projected into the plane and indicated by the cross mark. These results demonstrate the successful search of high-performing local maxima with the optimisation strategy.

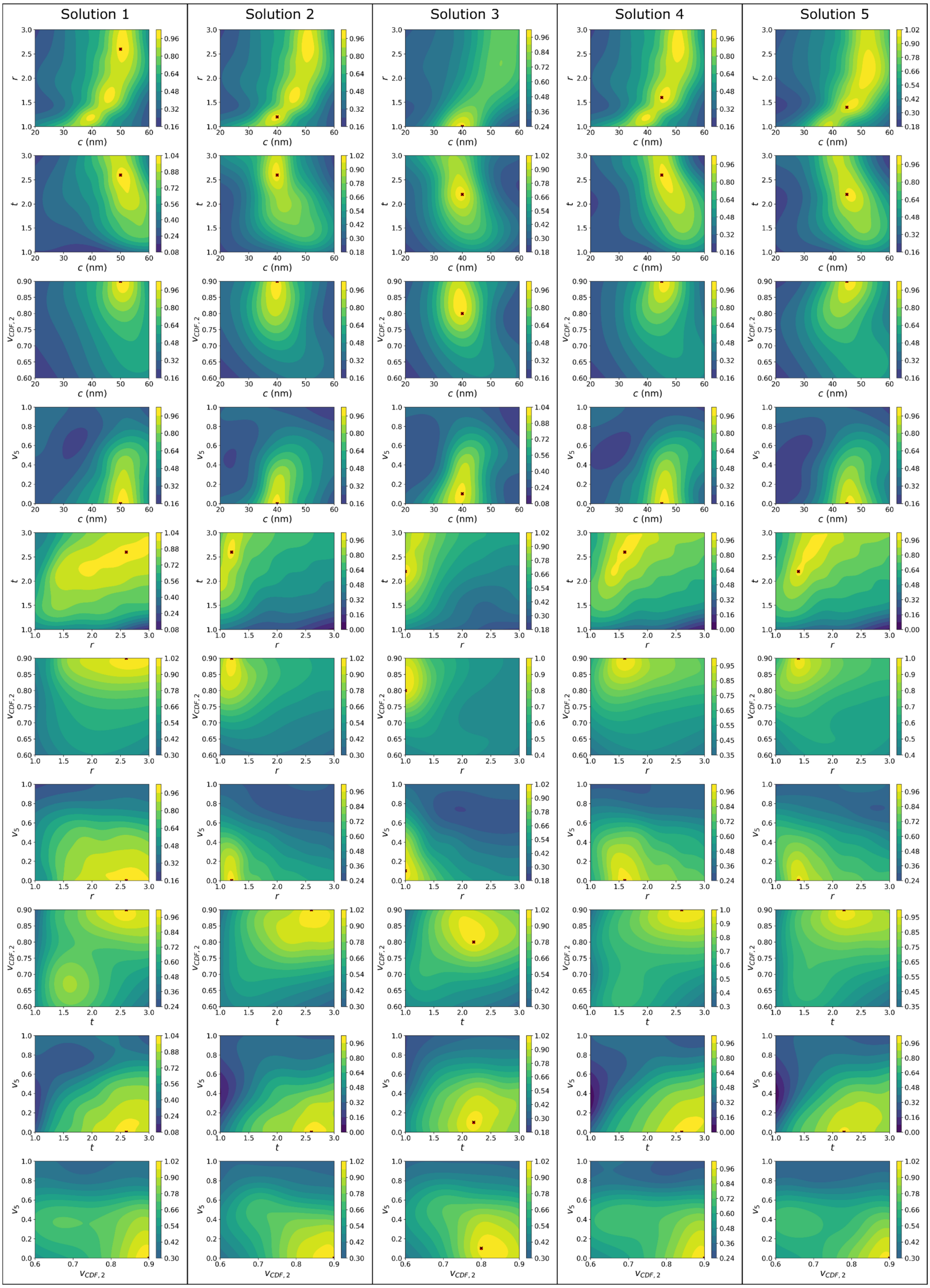

Figure S26. The distributions of the similarity metrics on the planes passing through the local maxima in the nanostructure parameter space of $(c, r, t, v_{DCF,2}, v_5)$. The red points indicate the local maxima, and the cross indicates the projection of the nearest solution. The colour bar was used

to show the similarity distribution on the two-dimensional plane. The similarity metrics were normalized to the range from 0 to 1 by a linear transformation.

Solution	v_1	v_2	v_3	v_4	v_5
**1**	0.8044	0.8441	0.8133	0.0000	0.0000
**2**	0.6891	0.8441	0.8133	0.1035	0.0409
**3**	0.7732	0.7472	0.7332	0.1155	0.0000
**4**	0.4764	0.0989	0.8084	0.0000	0.0000
**5**	0.4583	0.0000	0.5670	0.4820	0.1030
**Solution**	c **(nm)**	r	t	$v_{DCF,2}$	v_5
**1 (Local maximum 1)**	50	2.6	2.6	0.9	0.0000
**2**	50	2.6	2.6	0.9	0.0409
**3**	50	2.4	2.4	0.9	0.0000
**4 (Local maximum 2)**	40	1.2	2.6	0.9	0.0000
**5 (Local maximum 3)**	40	1.0	2.2	0.8	0.1030

Table S4. The input variables and their corresponding parameters of the nanostructures from the solutions when the starting step was 11 and $k_3 = 100$. Other parameters to control the nanostructure are constant: $a = b = 20$ nm and $v_{DCF,1} = 50$.

Despite the successful search of the multiple local maxima, multiple sampling points can correspond to the same local maxima. Simply sorting the sampling points according to their similarity does not enable diversity in the solution set. Another step of filtering out the sampling points near the same local maximum to get a set of solutions with more chance to correspond to more different local maxima is necessary. Here we used the K-nearest neighbour criterion. The solutions were selected so that every solution has the highest similarity among its ten nearest neighbours (including itself). Without the ten-nearest neighbour criterion, the top five solutions with the highest fitness are all in the vicinity of the global maximum. With the ten-nearest neighbour criterion, the five samples correspond to three different local maxima (including the global maximum). The input variables of the solutions and their corresponding nanostructure parameters are shown in **Table S4**. Their UV-Vis spectra and the discrete dipole representation of the nanostructures without by-products are shown in **Figure S27**.

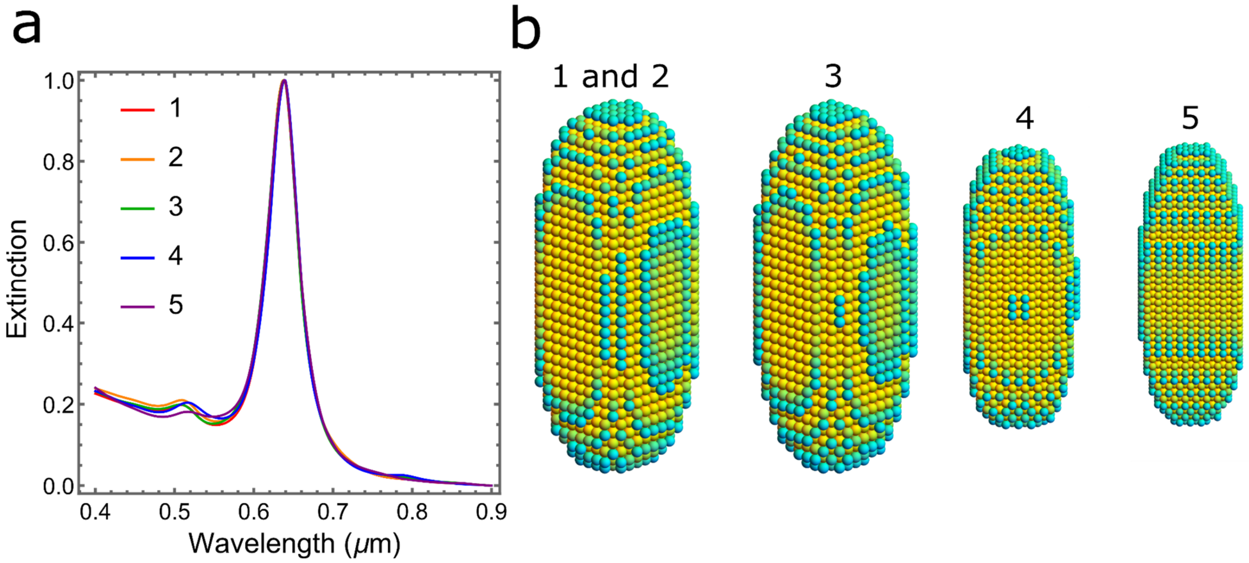

Figure S27. The results from the optimisation. The UV-Vis spectra (a) and the corresponding nanostructures (b) from the top five solutions from optimisation with the starting step of 11 and $k_3 = 100$ are shown. Solution 1 is the global maximum.

To further measure the diversity of the solution set, both the number of the unique local maxima ($N_L(x)$) and the least sampling number ($N_l(x)$) were defined as follows:

- For every solution, the local maximum that is closest to it is recorded.

1. $N_L(x)$: the number of the unique closest local maxima to the first x solutions.
2. $N_l(x)$: at least the first N_l solutions need to be selected so that the number of the solutions' closest local maxima is no less than x.

The two functions of $N_L(x)$ and $N_l(x)$ were compared before and after the selection of ten-nearest neighbours (**Figure S28**). With the selection criterion, the number of unique local maxima (N_L) closest to the given top x solutions was increased (**Figure S28a**). The required number of solutions (N_l) so that they correspond to different local maxima was decreased (**Figure S28b**). Both results demonstrated the increased solution diversity from the vicinity of different local maxima after the implementation of K-nearest neighbour criterion.

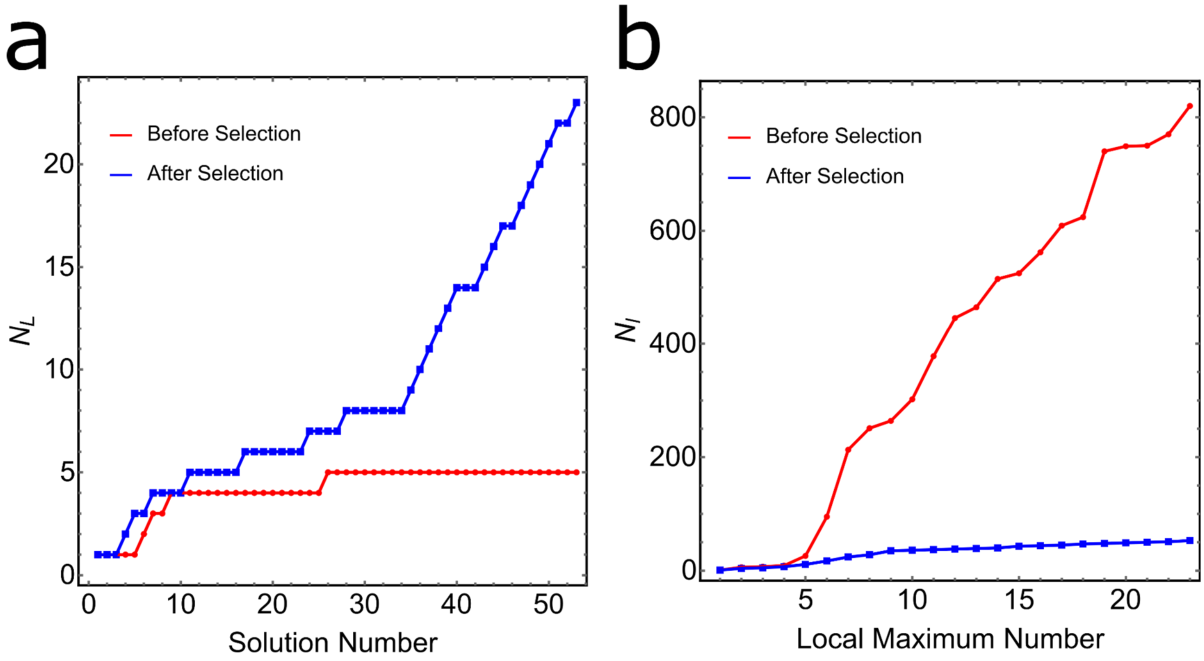

Figure S28. The results before and after the selection. The unique local maximum number N_L (a) and the least sampling number N_l (b) before (red) and after (blue) ten-nearest neighbour selection are shown respectively.

3. Exploration of the experimental chemical spaces in the seed-mediated synthesis of Au nanoparticles

In this section, we applied the multistep growth strategy to explore Au nanostructures (**Figure S29**). With the seed-mediated method and overgrowth of Au nanoparticles, multiple hierarchically-linked chemical spaces are generated and investigated. The resulting nanoparticles discovered in a given chemical space can be selected and used as seeds for the next level of exploration in the hierarchical series. The exploration algorithm based on MAP-Elites as described above was implemented to investigate three chemical spaces to get a diversified set of nanostructures.

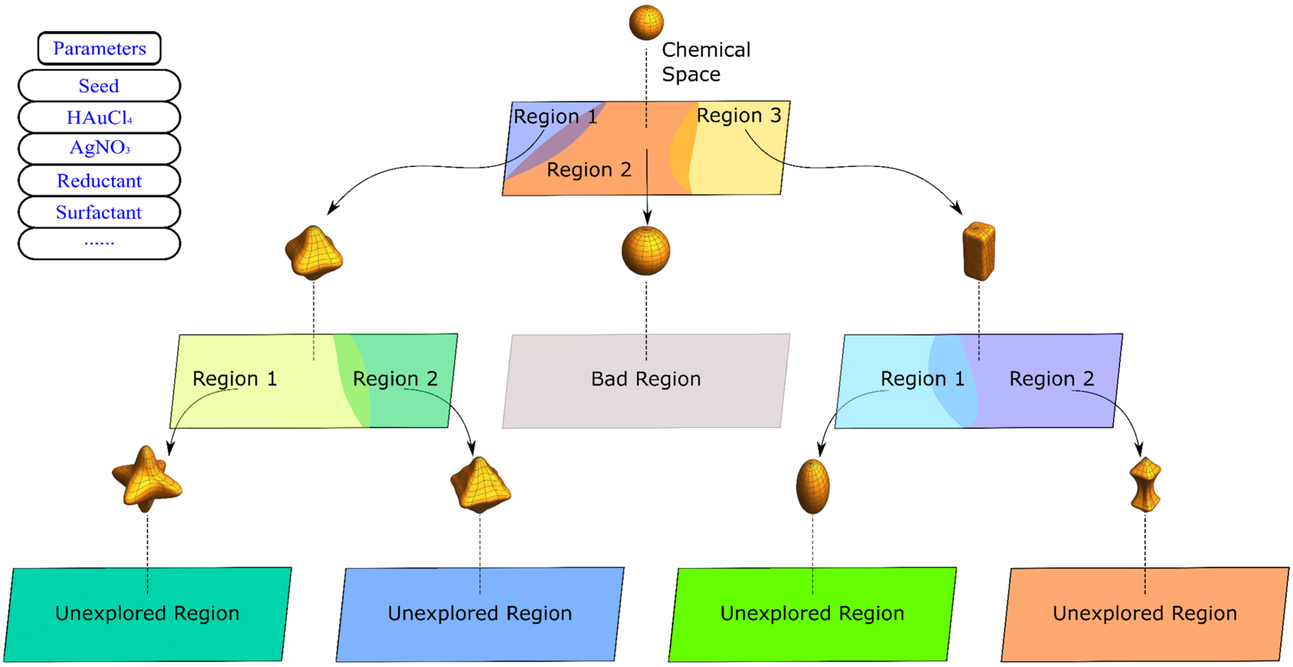

Figure S29. The scheme of the multistep synthesis strategy. Starting with an initial seed, the chemical space was explored and different nanostructures were discovered. The chemical space was further expanded using the found nanostructures as new seeds to create a diversified map of nanostructures.

Instead of detecting the nanostructures directly with TEMs, UV-Vis spectra were used to indicate the diversity of nanostructures during the search. The flexible manipulation of various UV-Vis features was demonstrated by varying the definition of classes and fitness in the exploration of the three chemical spaces, which resulted in the emergence of multiple uniquely-shaped nanoparticles (**Section 3.2, Section 3.3** and **Section 3.4**).

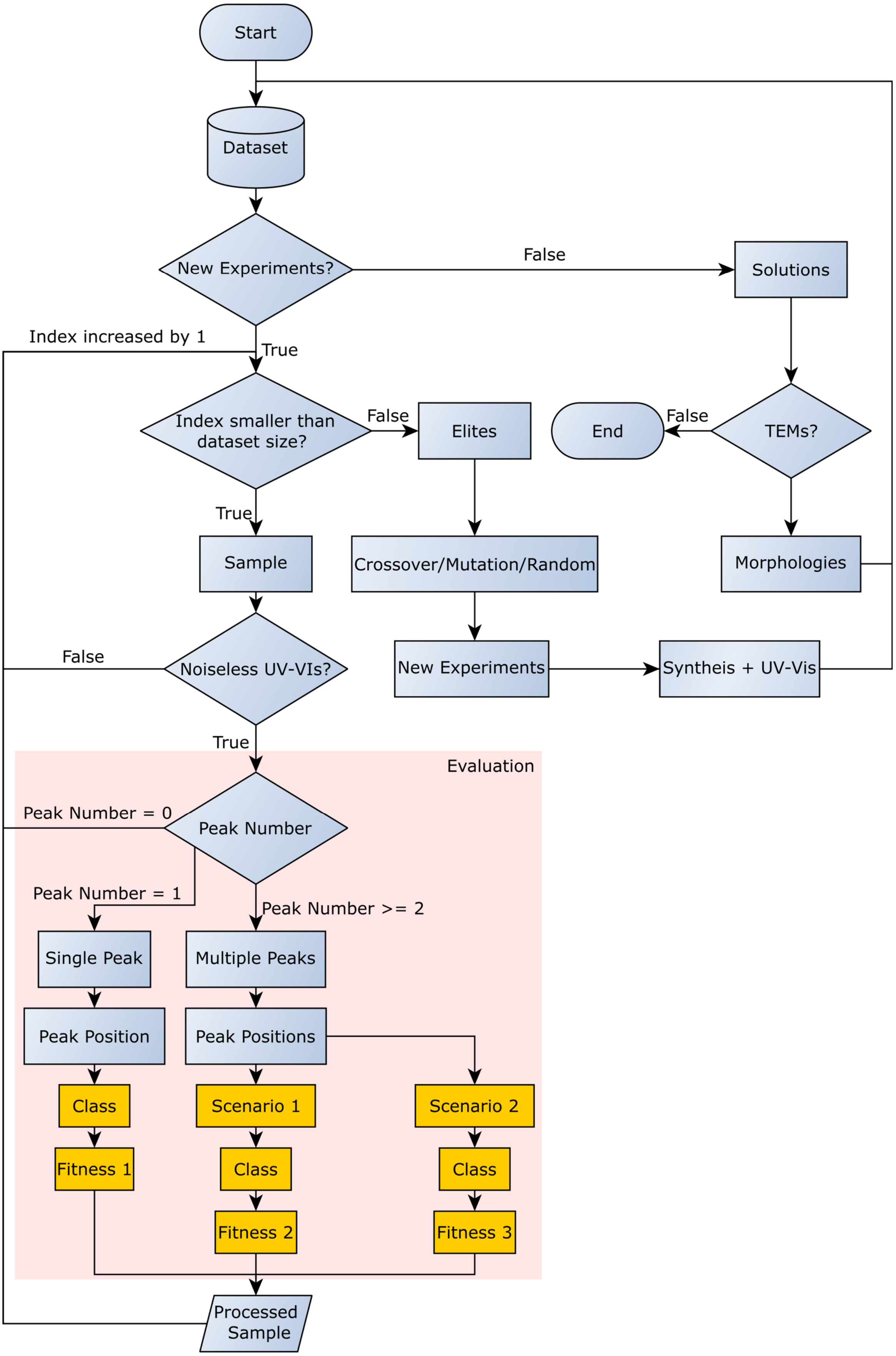

Figure S30. The flow diagram of the exploration strategy with the autonomous platform.

The flow diagram of the exploration strategy is shown in **Figure S30**. The dataset is updated during the investigation and used to design new experiments. If there is no prior knowledge of the chemical space and the dataset is empty, random sampling will be applied in designing the first batch of experiments. Otherwise, the samples in the dataset will be evaluated to allocate their classes and fitness with certain criteria, which will generate new experiments. The evaluation of the samples is described below:

1. The sample's UV-Vis spectrum is obtained and will be further processed if the following two criteria are met:
    a. The sample spectrum is not too noisy (which will be discussed in the experimental section below).
    b. The UV-Vis peak number is > 0.
2. If there is only one peak, the sample has **the single peak feature** and belongs to **the single-peak system**. The full spectral wavelength range is discretized into multiple subregions first and depending on which subregion the sample's peak is located in, a class index will be assigned to it. Its fitness will be calculated further (F_1).
3. If there are more than two peaks, the sample has **the multiple peak features** and belongs to **the multiple-peak system**. The most prominent two peaks will be considered. Again, the full spectral wavelength range is discretized into multiple subregions. The subregions in which the sample's most prominent and second most prominent peaks are will be recorded. Two different fitness functions (F_2, F_3) are calculated independently for a given sample. These fitness functions correspond to two different scenarios: **scenario 1** to focus on the most prominent peak, making it the only signal in the spectrum and **scenario 2** to encourage the continued production of multiple signal peaks. Two class indexes were assigned to the sample depending on the allocated subregions for each peak, and the chosen fitness function. For example, considering a sample ($\boldsymbol{x}$) with its two peaks in the $\{i^{th}, j^{th}\}$ subregion, two fitness values were calculated (F_2, F_3) respectively. Then two class indexes as $\{i^{th}, j^{th}, 1\}$ and $\{i^{th}, j^{th}, 2\}$, which indicate the different scenarios, will be assigned to the sample (See **Figure S31**).

The samples in the dataset were evaluated as described above. For every unique class, the sample with the highest fitness in the class was selected and designated as an elite. The elites from different classes defined the elite set. This elite set was used as the parent set in designing new experiments. The new experiments were generated by a combination of crossover and mutation processes within the elite set, and also a small portion of random sampling. The single-peak and multiple-peak systems can be explored simultaneously, sequentially or selectively by constraining the classes that can be

added to the elite set. The new experiments are conducted by the autonomous platform as described in **Section 0**. The process including conducting experiments, analysing data and generating new experiments iterated many times as needed.

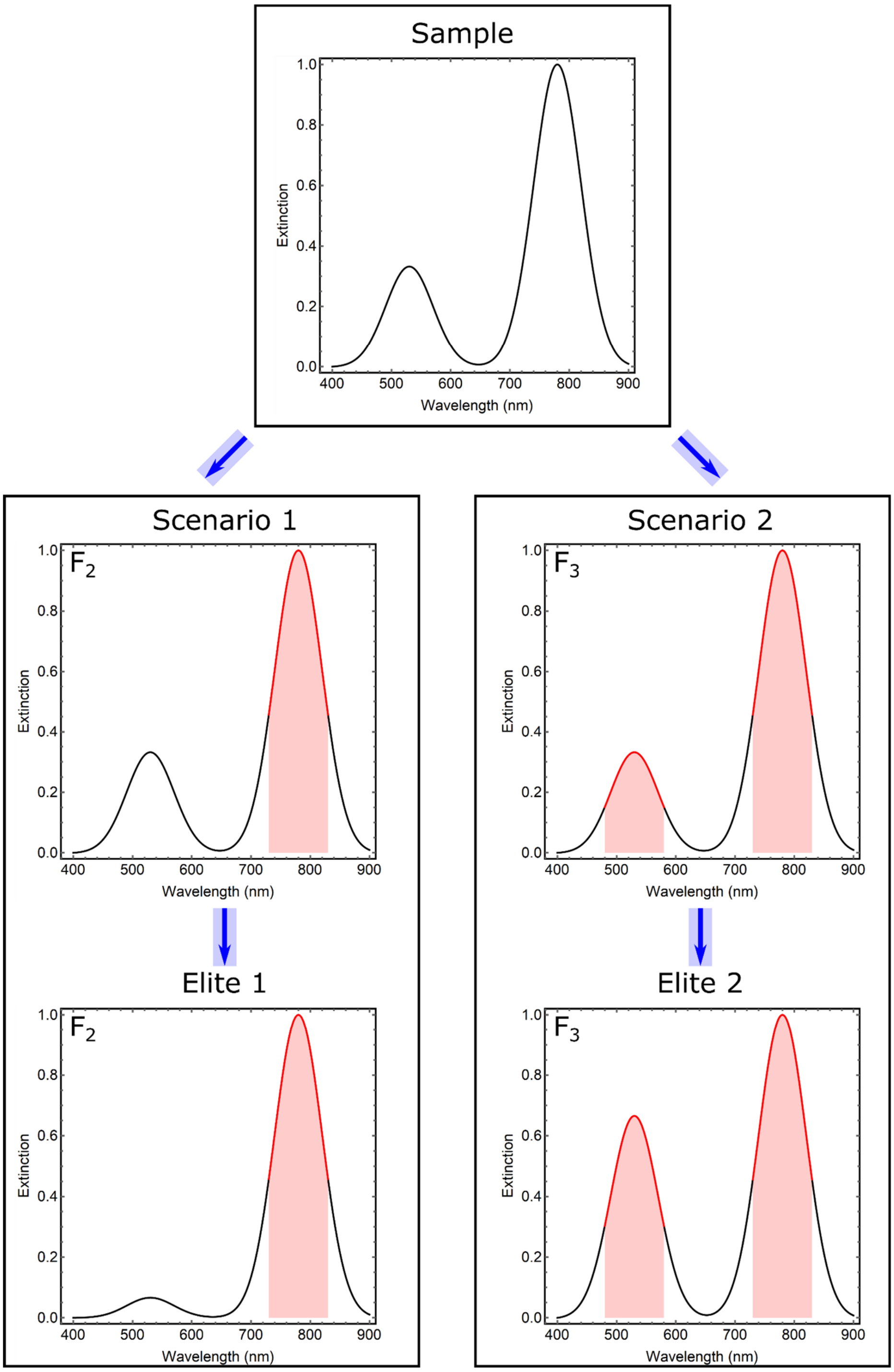

Figure S31. The scheme of the exploration strategy with two different scenarios in the multiple-peak system. Two different fitness functions (F_2, F_3) were used to facilitate the diversity of UV-Vis features, thus resulting into distinct elites belonging to two different classes.

The evaluation criteria of classification and fitness are essential because they directly affect the selection of the elites (parents), thus the exploration. Various general criteria can be defined depending on the UV-Vis features we wish to explore, all of which will be demonstrated with the

three chemical spaces detailed below. These criteria can be either static or dynamically changed during the exploration process. For example, considering the exploration of the single-peak system, the exploration can be focused by increasing the number of subregions in an area of interest within the spectral range whilst decreasing this number elsewhere. This change increases the number of the elites with a particular desired behaviour, thus allocating more resources to increase the performance of these elites. It makes the exploration more focused on elites with specific behaviours by scarifying the overall diversity of the elites. It can be done with greater confidence as the exploration proceeds and areas of the space are revealed to be less interesting.

Transmission electron microscopy offered detailed information of nanostructures directly and can be used to add extra information to the dataset after exploring the space for enough steps. The information from TEM images can help to modify the classification criteria to constrain the exploration for specific UV-Vis features. It can also help to modify the fitness function in a more explicit way, searching for samples which were difficult to reach before. This dynamic process was illustrated in exploring the first chemical space in three stages, each of which will be discussed in **Section 3.2**.

3.1. Chemical reagents

- All stock solutions were made using Type I ultrapure water (18.4 MΩ•cm).
- Hexadecyltrimethylammonium bromide, >99%, Acros Organics, CTAB.
- Hexadecyltrimethylammonium chloride, >99%, Acros Organics, CTAC.
- Gold (III) chloride trihydrate, 99.9%, Sigma-Aldrich, $HAuCl_4$.
- Ascorbic acid, 99.9%, Sigma-Aldrich.
- Silver nitrate, 99.9999%, Sigma-Aldrich, $AgNO_3$.
- Hydroquinone, 99.5%, Acros Organics.
- Hydrochloric acid, ACS Reagent, 37%, Honeywell Fluka™, diluted with Type I water.
- Sodium hydroxide, 98%-100.5%, Honeywell Fluka™, NaOH.
- Orion™ pH buffer solutions (4.0, 7.0 and 10.0), Thermo Scientific™.

3.2. Chemical space 1: Seed-mediated synthesis on cuboctahedron single crystals

The first chemical space is based on the 2 nm Au seed. It serves as our starting point of the exploration in the multistep growth. The reagent concentrations were tuned to suit the volumes of the syringe pumps.

3.2.1. Experimental details

Synthesis of the 2 nm Au seed:

1. $HAuCl_4$ (5 mL, 0.43 mM) and CTAB (5 mL, 0.2 M) were mixed in a 14 mL vial.

2. Under vigorous stirring ice-cold $NaBH_4$ (0.6 mL, 0.01 M) was added to the solution. The solution was stirred for 5 minutes.
3. The solution was aged for 25 minutes at 30°C.
4. The seed solution was diluted by adding 49.4 mL Type I ultrapure water for the accurate pump volume transfer.

Synthetic procedure:

Each reaction was performed with the followed order of addition by the platform, using volumes provided by the algorithm:

1. CTAB (0.2 M) added.
2. $HAuCl_4$ (0.86 mM) added.
3. $AgNO_3$ (0.25 mM) added.
4. Ascorbic acid (3.3 mM) added.
5. 10 seconds allowed for the reduction to complete.
6. Total volume constrained by addition of water if necessary.
7. 2 nm Au seed (0.50 mL) added.

The overall volume of the synthesised sample was constrained to 12.00 mL with boundary conditions in the algorithm and the addition of water. The boundary conditions will be discussed below. The volume of the seed solution for each reaction performed was fixed at 0.50 mL.

Each reaction solution was stirred during the synthetic procedure listed above. Once the synthetic procedure was over, all the solutions were kept undisturbed for 1 hour to complete the growth process before UV-Vis analysis.

UV-Vis characterisation and data processing:

All the samples from experiments were characterised with the QE-PRO UV-Vis spectrometer from Ocean Insight Ltd. The raw data was normalized to the range from 0 to 1, directed through a low-pass filter (with a passing frequency of 15) to remove noise, normalized again and further interpolated with a cubic spline. The average absolute difference before and after passing through the low-pass filter was calculated and if it was larger than a threshold (0.005), we regarded this sample as too noisy and discarded it. Samples without detectable peaks or with the largest peak prominence less than 0.2 were also discarded. The threshold of detecting peaks was set as 0.02 in the experiments. If not mentioned otherwise, the UV-Vis spectrum in this work was processed with the normalization, low-pass filter and interpolation steps as described above.

TEM:

Carbon coated 400 mesh copper grids (Agar Scientific/product code AGS160-4) were glow discharged using a Quorum Q150T ES high vacuum coater. Samples (5 µL) were drop-casted onto the carbon film surface and left to dry. JEOL1200 EX TEM was used at 80kV. Images were captured using a Cantega 2k × 2k camera and Olympus ITEM software.

Stability of the system:

To monitor and demonstrate the platform's ability to consistently reproduce results, 1 of 24 vials on the CRM was used to perform an identical reaction (**Table S5**) during each step of the entire 16-step exploration. These 16 samples were analysed alongside the exploration samples and served as standards for the stability of the whole system and the stock solutions being used. The spectra corresponding to these samples are shown in **Figure S32a**, with small differences in the shape yield and shift in the peak of the longitudinal mode (**Figure S32b**). Given the chemical sensitivity in nanomaterial synthesis, such minor differences can be attributed to inherent variation in chemistry as these are growth processes rather than strict mechanistic processes as well as the slight inconsistency of robotic operations, temperate, stock solutions and UV-Vis spectrometers, etc., but were found to be negligible on this system and consistently monitored.

Standard	CTAB (mL)	$HAuCl_4$ (mL)	$AgNO_3$ (mL)	Ascorbic acid (mL)	Water (mL)	Seed (mL)
**1**	4.40	2.50	1.80	1.10	1.80	0.50

Table S5. The input parameters of the standard samples to test the stability of the autonomous platform in chemical space 1. The concentrations of the reagents are available in the same section above.

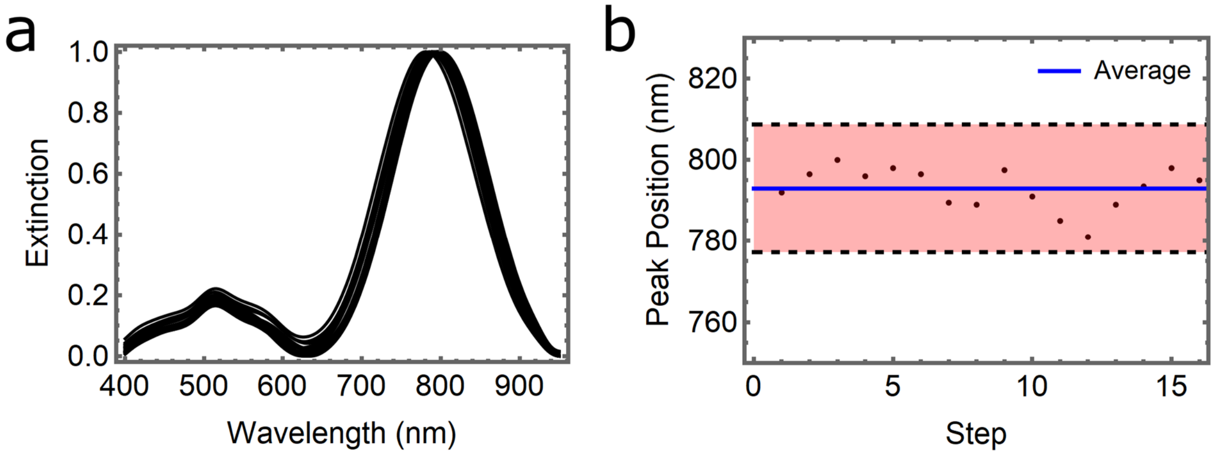

Figure S32. The demonstration of the stability of the platform. (a) The normalized UV-Vis spectra of the standard samples from different steps to demonstrate the stability of the autonomous platform during the exploration. (b) The different peak positions corresponding to the longitudinal mode of nanorods. The mean value is 793 nm with a standard deviation of 5 nm. All peak positions are within 3 standard deviations of their mean value as indicated by the red background.

3.2.2. Data processing and algorithm

To explore the chemical space and obtain the nanostructures with high qualities, the exploration algorithm was customized from several aspects including adding extra operations in mutation and crossover to satisfy the new boundary conditions, changing the definition of different classes in the behaviour space, and modifying the fitness function accordingly. More details will be discussed in the following context.

Input space, boundary conditions and sampling strategy

The input space is defined by the normalized variables with a range from 0 to 1 derived from the chemical reagent volumes (with the range from 0 to 11.5 mL as listed below) through a linear transform, and the overall volume of a sample should be constrained to avoid overflow, which applies boundary conditions in the input space. After crossover and mutation, a new set of experiments will be generated through the reverse linear transform to map the variables back to the reagent volumes. The ranges of the volumes for individual reagent and boundary conditions are listed below:

- CTAB (0.2 M): 0 to 11.50 mL
- $HAuCl_4$ (0.86 mM): 0 to 11.50 mL
- $AgNO_3$ (0.25 mM): 0 to 11.50 mL
- Ascorbic acid (3.3 mM): 0 to 11.50 mL
- Boundary conditions: $\sum_i^n v_i \leq 1$ and $v_i \in [0,1]$

where v_i is the normalized variable from the linear transformation of the reagent volume (CTAB, $HAuCl_4$, $AgNO_3$ and ascorbic acid). Note in every experiment, an extra amount of water was added to keep the overall volume constant if necessary.

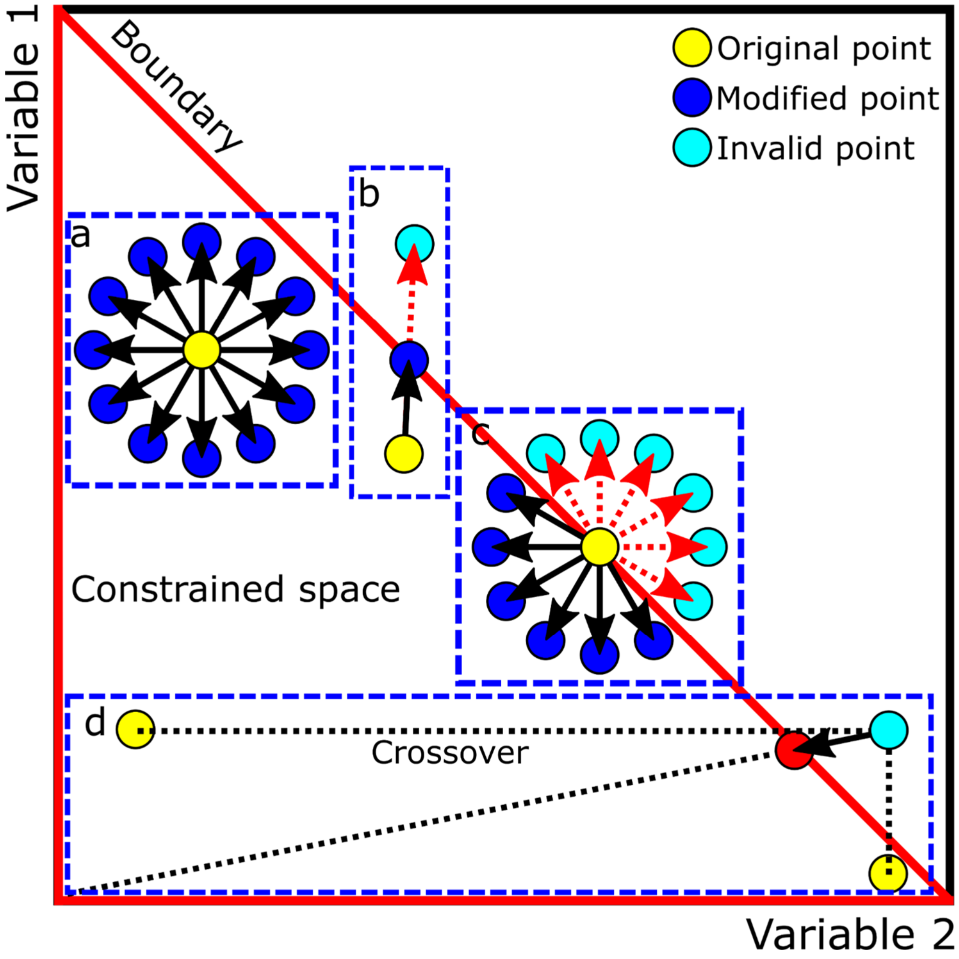

Figure S33. The scheme of the newly added operations in the exploration algorithm in a two-dimensional space. (a) The mutations based on the original sampling point in different directions without hitting any boundaries. (b) The mutation from an original sampling point that hits the boundary. The perturbation vector is thus scaled down. (c) Possible valid (black arrows) and invalid (red arrows) mutations of a sampling point on the boundary. (d) The crossover of two samples resulting in an offspring out of the boundary. The variables of the offspring are scaled down to the boundary. All the valid/invalid perturbations are represented by black/red arrows.

In the mutation, a vector will be generated from the multiple-dimensional Gaussian distribution and is then added to the original sampling point. If the addition breaks a boundary condition, an absorption boundary will be set: the perturbation vector will be scaled down so that it is still within the boundary (**Figure S33a-b**). For samples on the boundaries, invalid mutations can happen due to the scaling operations and absorption boundary conditions described above, which can trap the samples there. To mitigate this problem, a flag is created according to the chance of mutation first and if the sample should mutate, the mutation was performed until a valid outcome occurs (**Figure S33c**).

In the crossover, after exchanging variables between two samples, the boundary condition can be broken. In this case, all the input variables will be scaled down so that the summation of them is 1 to maintain the boundary condition and avoid overflow in the experiments (**Figure S33d**).

Algorithm parameters

The investigation of chemical space 1 was divided into three stages, each of which was subdivided into multiple steps. A single step of any stage was simply 24 reactions performed on the platform (including the standard sample). To begin, the first random sampling step and the subsequent 9 steps were for the open-ended exploration without bias, with multiple systems and general fitness functions (stage 1). TEM validation confirmed the existence of nanorods in multiple-peak systems when fitness was concerned with fitness scenario 1 (increasing the prominence of a single peak). The constrained exploration (stage 2) which focused on samples with multiple-peak features, with an explicit fitness function, was conducted for four steps. The new fitness function was defined so that the less prominent second peak was minimized. The final of the three stages was an exploitation process and was performed for an additional two steps. During exploitation, the absorbance signals from by-products were explicitly considered during the search. The function of this final stage was to decrease/remove signals from assumed by-products. The majority of samples with further increased absorption bands from the most prominent peaks were discovered after stage 2 and stage 3.

Among the 23 samples available per step of any given stage, 10 samples were mutated from the elite (parent) set, 10 samples were from crossover among elites each of which had a 40% chance of mutation and 3 samples were randomly generated in the input chemical space. This distributed equal resources for crossover and mutation processes in each step. As the dimensionality of the chemical space is high, the relatively small initial random dataset (23) has the potential to bias the exploration, therefore a small portion of random sampling was added in every step to reduce the initial bias. The standard deviations of the multi-dimensional Gaussian distribution were set to 0.05, 0.08, and 0.08 for stage 1, 2 and 3 respectively. The slight increase of the standard deviation in stage 2 and 3 was used to get rid of any local maxima from the open-ended exploration. Considering the dimensionality of the space, we set 1 step for initial random sampling and 15 steps for investigation using MAP-Elites.

Open-ended exploration of chemical space 1 (first 10 steps including random sampling)

The open-ended exploration of the chemical space was started by sampling 23 points randomly. These samples were then used to create an initial parent set.

Classes and fitness functions

In the open-ended exploration of the real chemical space, both single and multiple peaks should be considered. For samples with one single peak, the class was purely dependent on the subregion which its peak was located in. For samples with multiple peaks, the most prominent two peaks were selected for further evaluation. In several literature reports, many Au nanostructures have intrinsically two comparable prominent peaks (*55*, *56*). But also maximizing one of the peaks can lead to a higher yield

of one nanostructure if these peaks correspond to two different nanoparticles. As described above, for the multiple-peak systems, we evaluated a given sample's performance in two scenarios: if the UV-Vis absorption is purely from one peak (scenario 1, Eq. (25)) or from both peaks (scenario 2, Eq. (26)). It was necessary to include both scenarios in the exploration to enable diversity. In the multiple-peak systems, the UV-Vis peak positions were no longer the only criteria for classification and extra classes with varying fitness should be considered.

Wavelength region discretization:

- **Single-peak system:**
  - 400-600 nm at 25 nm intervals
  - 600-950 nm at 50 nm intervals
- **Multiple-peak system:**
  - 400-950 at 50 nm intervals.

In the single-peak system, the class assigned is entirely derived from the subregion the peak lies in. In the multiple-peak system, the subregion indexes in which the most and the second most prominent peaks are located were obtained, and used to determine the class. The two fitness functions were further evaluated which indicated if one of the peaks is dominant or both peaks contributed significantly to the spectrum respectively. Finally, this sample was assigned to two classes corresponding to the two fitness functions respectively. This strategy can create elites with similar peak positions but different UV-Vis features, which further enabled the diversity of our exploration.

The fitness functions (F) used in the single-peak system (Eq. (24)) and the two different scenarios of the multiple-peak system (Eq. (25) and (26)) are defined as follows:

Single-peak system:

$$F = \frac{\int_{x_{peak1}-w}^{x_{peak1}+w} I_x dx}{\int I_x dx} \tag{24}$$

Multiple-peak systems:

- Scenario 1: UV-Vis from one dominant peak:

$$F = \frac{\int_{x_{peak1}-w}^{x_{peak1}+w} I_x dx}{\int I_x dx} \tag{25}$$

- Scenario 2: UV-Vis from both peaks:

$$F = \frac{\int_{x \in A \cup B} I_x dx}{\int I_x dx} \tag{26}$$

where I_x is the absorption of the UV-Vis spectrum at wavelength x, x_{peak1} and x_{peak2} are the position of the most and the second most prominent peaks, the integration range in Eq. (26) is defined by the union of two sets ($A \cup B$), where $A = [x_{peak1} - w,\ x_{peak1} + w]$ and $B = [x_{peak2} - w,\ x_{peak2} + w]$, and w is a parameter to define the region near the peak and is tuneable for individual fitness functions. Here we used 50 nm for all the functions in the exploration.

Constrained exploration and exploitation in chemical space 1 (steps 11-16, including stage 2 and 3)

Following the initial 10 steps of exploration of chemical space 1, Au nanorods were found in the multiple-peak system when following scenario 1. Later, the criteria of classification were changed to put more focus on this scenario. During the initial steps of open-ended exploration, the focus of scenario 1 fitness function (Eq. (25)) was purposefully general. It was used to calculate the absorption area percentage near the dominant peak, however, this alone is not explicit enough to guide further search. A more explicit fitness function to decrease the secondary peak, decrease peak broadness and also to minimize the formation of by-products should be defined (Eq. (27)). The search continued for another 4 and 2 steps (stage 2 and 3 respectively) successively with different coefficients of the new fitness function (Eq. (27)). Overall, they correspond to the three stages with varying evaluation criteria for open-ended exploration (10 steps), constrained exploration (4 steps) and exploitation (2 steps).

The aim of constrained exploration and exploitation is to efficiently increase the original fitness value (Eq. (25)) by considering other explicit factors that influence the absorption band. The explicit features considered by (Eq. (27)) allowed us to increase not only fitness values in stages 2 and 3, but also the more general implicit fitness measure of Eq. (25) of the final individual elites. The original fitness function represents a general form of sample performance and the samples with the highest scores of it were selected for TEM validation.

Classes and fitness

The class definition in both constrained exploration and exploitation is similar to that in the simulated chemical space. To make the exploration focused, the possible number of classes was decreased. The wavelength ranging from 400 nm to 950 nm was discretized with an interval of 50 nm and the most prominent must be in a higher wavelength subregion compared to the second one. Depending on

which subregion the most prominent peak was in, a class index was assigned to it. The explicit fitness function (F) is defined below:

$$F = -k_1 w_1 - k_2 w_2 - k_3 \overline{I_{x,w}} - k_4 \overline{I_b} \tag{27}$$

where w_1 and w_2 are the widths at half prominence of the most and second most prominent peak, $\overline{I_{x,w}}$ is the average absorption near the lower peak position x within a range of w, $\overline{I_b}$ is the average absorption of a user-specified region that indicates the existence of by-products. Here this region was set to be from 550 nm to 600 nm and also $w = 50$ nm.

To initialise stage 2, all the available data were evaluated with $k_1 = 0.002, k_2 = 0, k_3 = 1, k_4 = 0$ to create the initial parent set. The coefficients were selected to decrease the composition of the lower peak and minimize the broadness of the most prominent peak. 4 steps were run with the same coefficient for constrained exploration. A further 2 steps were run with modified coefficients of $k_1 = 0.002, k_2 = 0.002, k_3 = 1, k_4 = 1$, which defined the fitness more explicitly by considering the by-product peak to increase purity. In the later 2 steps, the search is more focused on minimizing the peaks corresponding to spheres or potential polyhedra, while the broadness of the most prominent peak should be maintained, so that the monodispersity of the rods were not sacrificed. After the constrained exploration and exploitation, all the available samples would be re-evaluated by the original fitness (Eq. (25)) for the final solutions. Consequently, four new nanorod samples with increased fitness were found. The UV-Vis and TEM characterisation will be discussed below.

3.2.3. Results and discussions

After the initial 10 steps of open-ended exploration, 26 elites were found (**Figure S34a**). Depending on the peak numbers and the desired UV-Vis features, they were divided into three sets: 1. single-peak system; 2. multiple-peak system with scenario 1; 3. multiple-peak system with scenario 2. The final number of elites contributed from each set was 8, 9, and 9 respectively. The fitness values of different elites in the exploration are shown in **Figure S34b**. The UV-Vis spectra of the individual sets are shown in **Figure S34c-e**.

In the single-peak system, a variety of samples with their peak positions spreading from 420 nm to 650 nm were obtained.

For the multiple-peak systems with different scenarios, their elite numbers are the same in every step. The diversity of this system was extended so that the elites with similar peak positions can have different relative peak intensities (e.g., Elite 10 & 19 with peaks in 500-550 nm and 650-700 nm; 17 & 26 with peaks in 500-550 nm and 800-850 nm in **Figure S34**) by defining the two search scenarios as described above. The synthetic conditions for the elites are listed in **Table S6**.

It is essential to analyse the emergence of the new elites in the open-ended exploration. The elites can be newly found or get replaced by samples with higher fitness in the same class. As shown in **Figure S34g**, the new elites can be from the crossover, mutation or random sampling with increased fitness, indicating all the three sampling strategies contribute to generating new elites.

During the open-ended exploration, three sets were studied including 1. Single-peak system; 2. multiple-peak system with one peak contributing to the spectrum (scenario 1); 3. multiple-peak system with two peaks contributing to the spectrum (scenario 2). Thus, the elites can be divided into three sets, which are separated as region 1, 2 and 3 respectively in **Figure S34g**. Region 1 belongs to the single-peak system while region 2 and 3 belong to the multiple-peak systems. The number of times of generating new elites from previous elites of different systems defines their interactions as discussed in the manuscript.

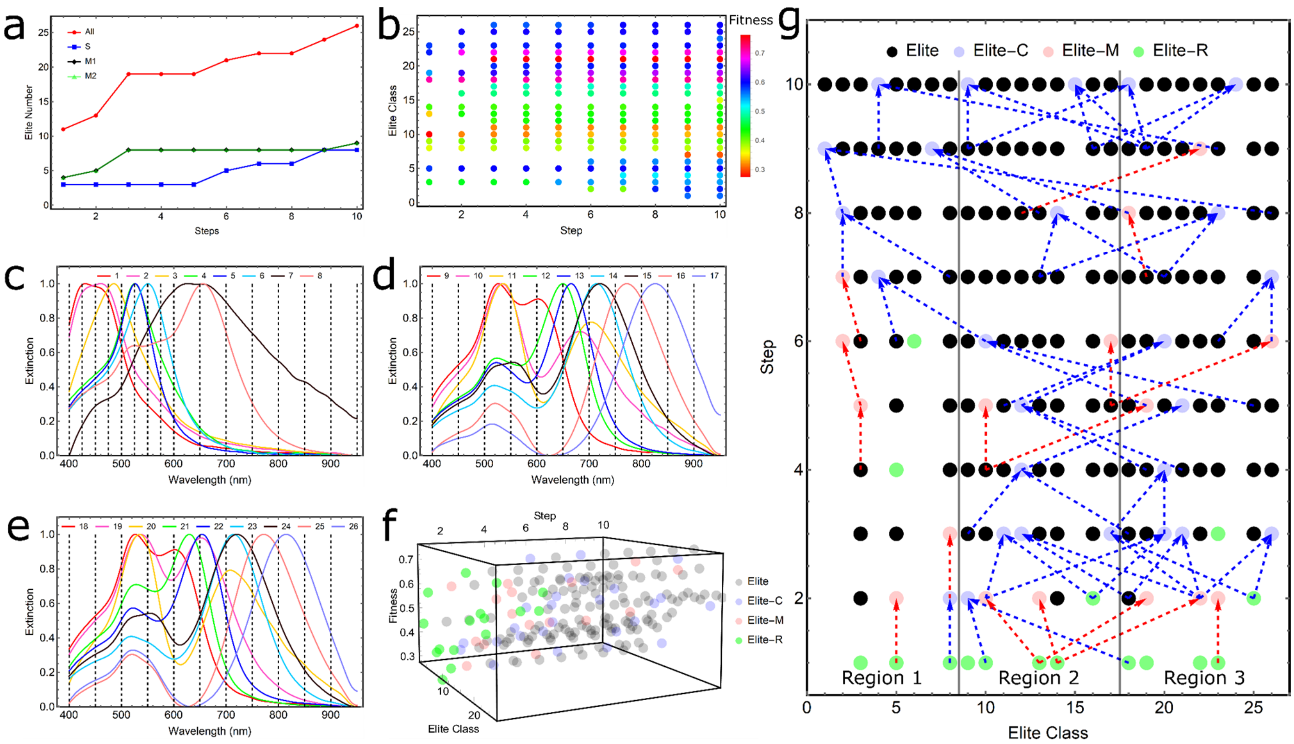

Figure S34. The open-ended exploration of the first chemical space with multiple systems. (a) The number of elites found during open-ended exploration. S (blue line), M1 and M2 were used to indicate the single and multiple-peak systems with the different scenarios respectively. (b) The distribution of fitness values of different elites throughout the exploration. Depending on the class of the elite, the fitness value can vary and several samples show a large increase during the exploration. (c) The final UV-Vis spectra of the elites in the single-peak system after exploration. (d) The final UV-Vis of the elites in the multiple-peak system (scenario 1) after exploration. (e) The final UV-Vis of the elites in the multiple-peak system (scenario 2) after exploration. (f) The elite distribution in the three-dimensional space defined by fitness value, elite class and steps. (g) The evolution of elites via crossover, mutation and random sampling in the exploration. The red and blue arrows indicate the propagation of experimental conditions to a new elite through mutation and crossover. The three regions correspond to three sets: S, M1 and M2. Note when the initial parent number is small, one elite can be sampled repeatedly in the crossover, but only its experimental conditions are used to

generate the new experiment. Elite-C, Elite-M and Elite-R in (f) and (g) represent the new elites from crossover, mutation and random sampling respectively.

Class Index	CTAB (mL)	$HAuCl_4$ (mL)	$AgNO_3$ (mL)	Ascorbic acid (mL)	Water (mL)	Seed (mL)
**1**	0.90	6.46	3.56	0.12	0.46	0.50
**2**	0.89	6.22	3.10	0.00	1.29	0.50
**3**	0.06	6.02	3.21	0.00	2.21	0.50
**4**	1.12	1.72	0.09	0.79	7.78	0.50
**5 (L1-1)**	1.51	2.53	0.09	7.13	0.24	0.50
**6**	1.62	1.05	2.52	5.54	0.77	0.50
**7**	0.00	3.31	0.53	2.75	4.91	0.50
**8**	2.70	1.74	0.41	6.05	0.60	0.50
**9**	0.78	3.28	0.29	4.31	2.84	0.50
**10**	0.78	1.72	3.29	0.79	4.92	0.50
**11**	1.31	1.72	3.29	0.79	4.39	0.50
**12**	1.31	4.42	0.48	2.88	2.41	0.50
**13**	0.65	3.71	0.48	1.84	4.82	0.50
**14**	1.12	3.57	0.67	1.64	4.50	0.50
**15**	3.47	2.72	2.33	2.58	0.40	0.50
**16**	5.23	1.72	3.29	0.79	0.47	0.50
**17 (L1-6)**	4.86	2.74	2.93	0.97	0.00	0.50
**18**	0.78	3.28	0.29	4.31	2.84	0.50
**19**	0.78	3.28	1.83	2.49	3.12	0.50
**20**	1.31	1.72	3.29	0.79	4.39	0.50
**21**	0.95	4.89	0.46	4.15	1.05	0.50
**22**	1.03	5.01	0.49	2.51	2.46	0.50
**23**	1.12	3.57	0.67	1.64	4.50	0.50
**24**	3.47	2.72	2.33	2.58	0.40	0.50
**25**	5.23	1.72	3.29	0.79	0.47	0.50
**26**	1.58	3.30	2.93	0.97	2.72	0.50

Table S6. The input parameters of the elites after exploring chemical space 1 for 10 steps. The concentrations of the reagents are available in the same section above.

After the open-ended exploration, nanorods were discovered in the multiple-peak system (scenario 1). The UV-Vis peak with a longer wavelength corresponds to the longitudinal mode of nanorods, while the shorter one corresponds to the transverse mode, together with by-products of spheres,

triangles or polyhedra. To increase the absorption band of the Au nanorods samples, further 4 steps for constrained exploration (stage 2) and 2 steps for exploitation (stage 3) were run with new classification criteria and fitness as described above. It is noted all the five elites corresponding to Au nanorods with different aspect ratios are available after the initial 10 steps of exploration (stage 1). Compared to the results from the open-ended exploration, the further search found samples with increased absorption band (Eq. (25)). Consequently, four new Au nanorods (R1-R4) with higher scores were available. The UV-Vis spectra of the best samples before and after the two stages are shown in **Figure S35** for comparison. The increase of the absorption band defined by the original fitness function (Eq. (25)) of these four classes during the overall 16 steps is shown in **Figure S36**.

The final UV-Vis spectra of Au nanospheres and nanorods are shown in **Figure S37**. The synthetic conditions for the nanorods as well as the nanospheres are shown in **Table S7**. Their labels in the manuscript were also shown in the same table. Note the two nanorod samples with low aspect ratios (R1 and R2) belong to different classes with peak positions near the boundary of the subregions. A similar phenomenon was also observed in the simulated chemical space (**Figure S22c**).

Label	CTAB (mL)	$HAuCl_4$ (mL)	$AgNO_3$ (mL)	Ascorbic acid (mL)	Water (mL)	Seed (mL)
**S1 (L1-1)**	1.51	2.53	0.09	7.13	0.24	0.50
**R1 (L1-2)**	1.31	4.42	0.48	2.88	2.41	0.50
**R2 (L1-3)**	1.70	5.14	0.58	2.91	1.17	0.50
**R3 (L1-4)**	4.28	2.90	1.26	1.04	2.02	0.50
**R4 (L1-5)**	4.66	2.83	1.27	1.17	1.57	0.50
**R5 (L1-6)**	4.86	2.74	2.93	0.97	0.00	0.50

Table S7. The input parameters of the final products in chemical space 1. The concentrations of the reagents are available in the same section above.

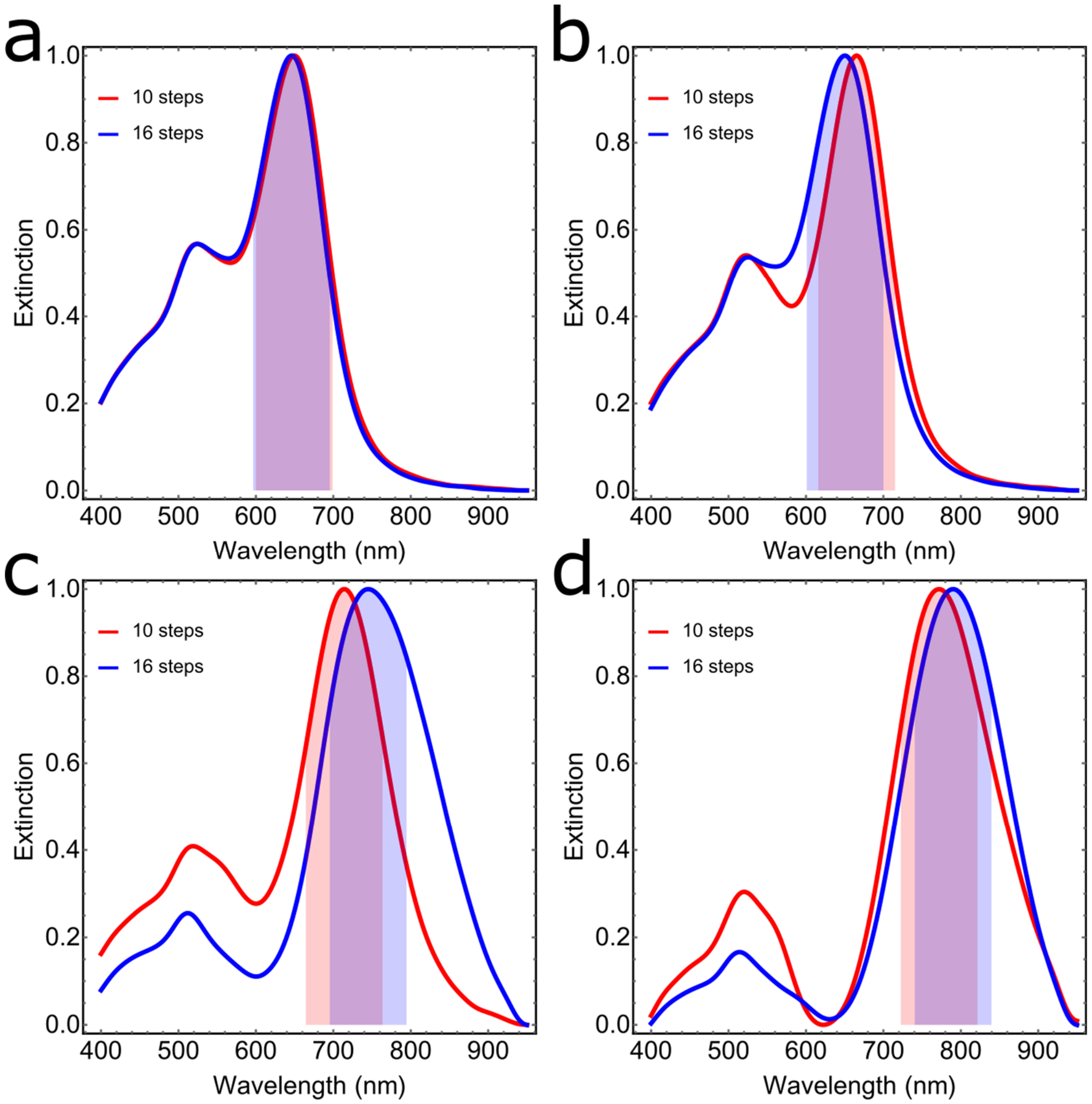

Figure S35. The comparison of the UV-Vis spectra of four Au nanorods with improved absorption band before (red) and after (blue) the two stages. (a) to (d) corresponds the best spectrum in the same class as R1 to R4 respectively. The comparison of the same class as R5 is not shown because the absorption band was not increased in the later 6 steps.

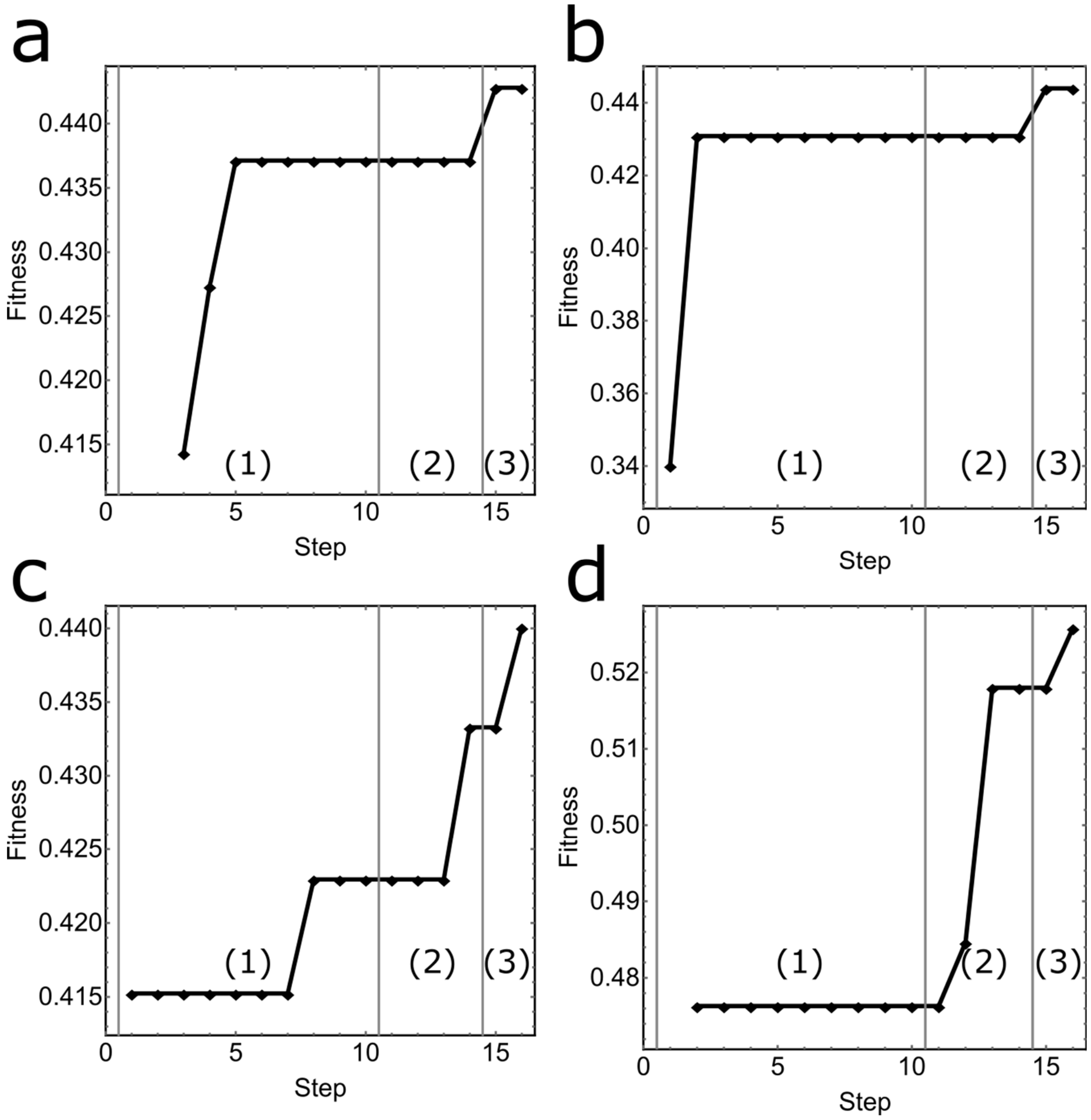

Figure S36. The increase of the highest absorption band defined in Eq. (25) during different stages with 10,4 and 2 steps respectively. (1)-(3) represents the open-ended exploration, constrained exploration and exploitation. (a) to (d) corresponds the increase of the fitness within the same class as R1 to R4 respectively.

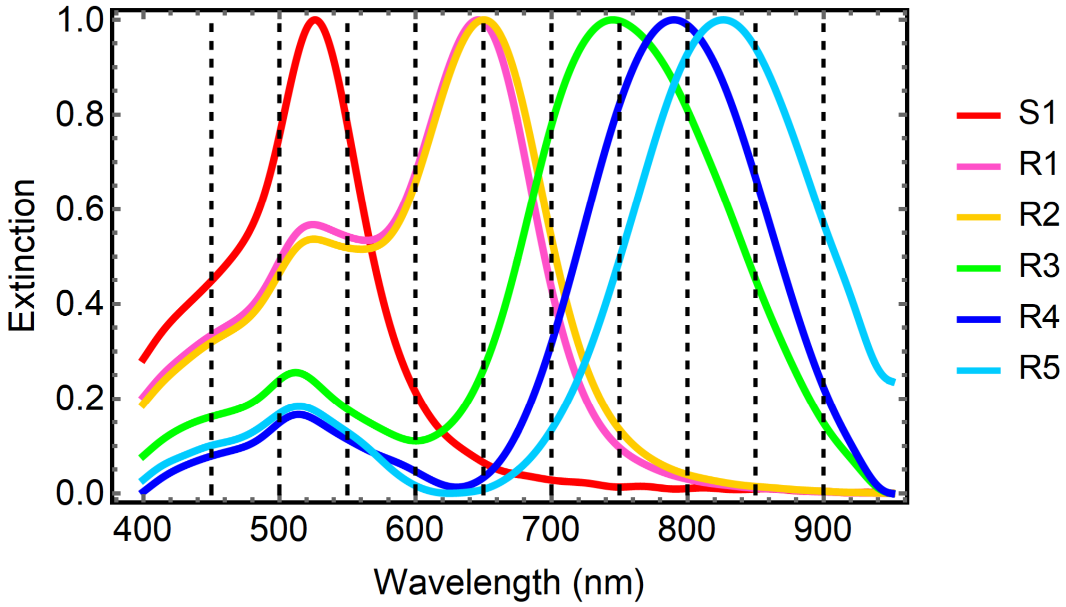

Figure S37. The UV-Vis spectra of the five Au nanorods labelled with R1 to R5. The UV-Vis peak corresponding to the longitudinal mode was ranged from around 650 nm to 820 nm.

Nanospheres and nanorods with different aspect ratios were found (**Figure S38-Figure S43**).

<u>Because the as-synthesised solutions would be used as the new seeds, to reflect the actual states of them, all samples for TEM characterisation were only centrifuged at 12000 rpm for 10 minutes and washed by water to concentrate them, without any further purification</u>. The exact instruction is below:

1. Centrifuge the sample at 12000 rpm for 10 minutes.
2. Get rid of the supernatant.
3. Dissolve the nanoparticles with pure water with the same original volume.
4. Centrifuge at 12000 rpm for 10 minutes again.
5. Get rid of the supernatant.
6. Dissolve the nanoparticles to the desired volume with pure water for TEM characterisation.

The fourth rod sample (R4) with a dominant peak around 790 nm has a relatively high aspect ratio (compared to R1 and R2) with a concave and sharper contour compared to R3 and R5 (**Figure S39-Figure S41**). It was used as the seed for the next level of exploration, assuming these features can lead to the emergence of novel nanostructures. The chemical space using nanorods as the seed will be discussed in the next section.

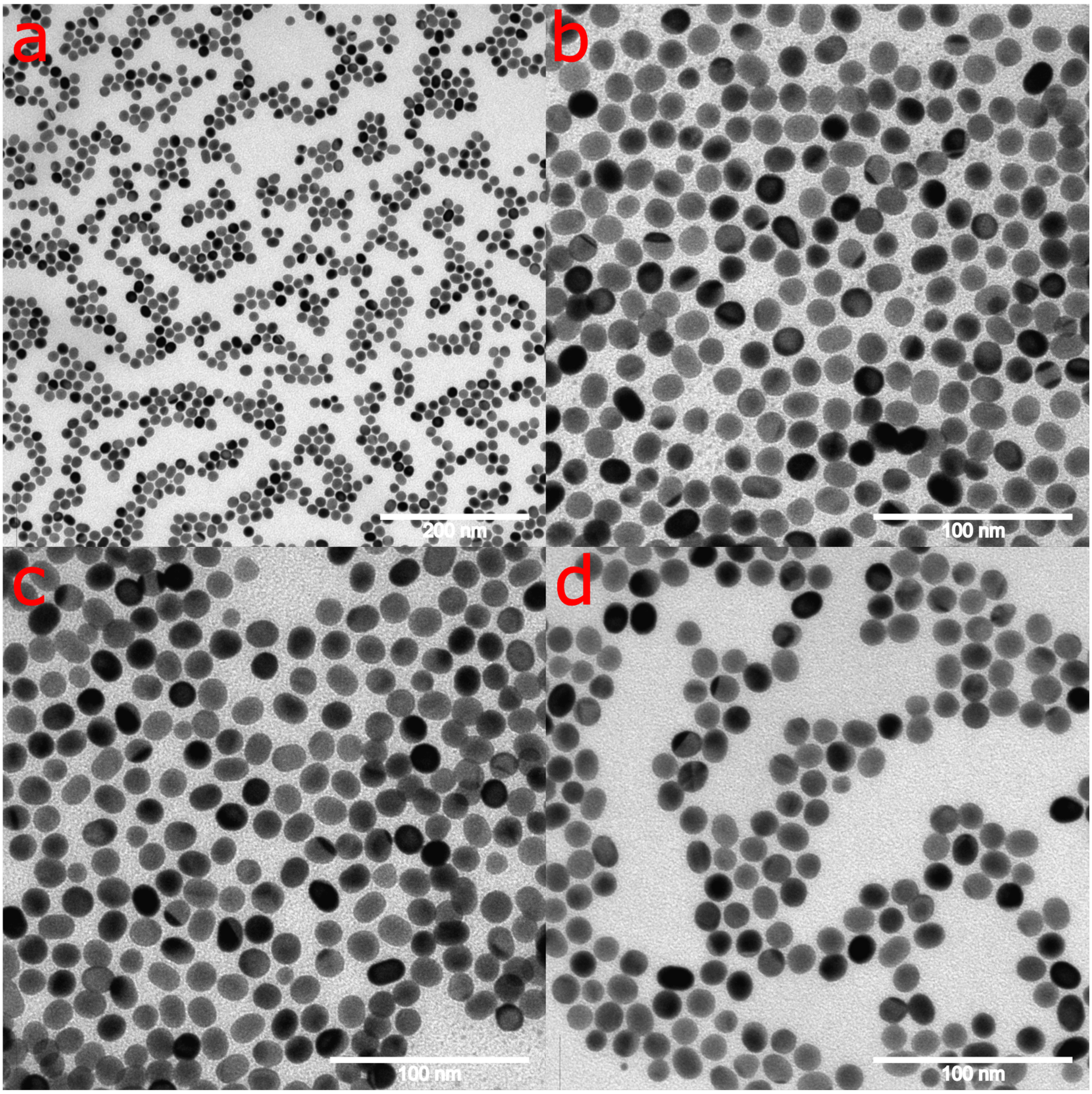

Figure S38. The TEM images for sample S1 of nanospheres (as labelled as L1-1 in the manuscript). The scale bars are shown in the images.

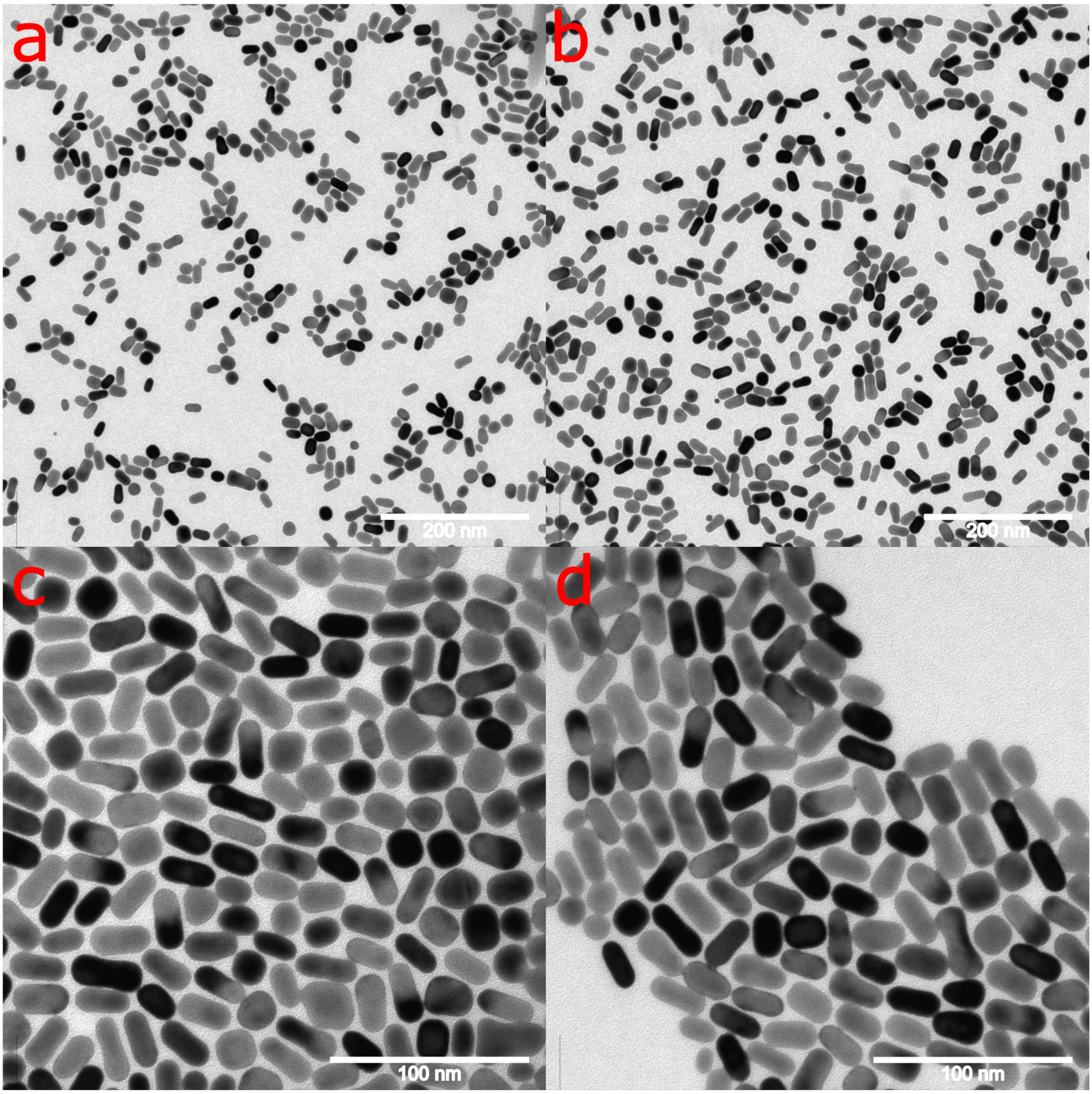

Figure S39. The TEM images for sample R1 with a low aspect ratio (as labelled as L1-2 in the manuscript). The scale bars are shown in the images.

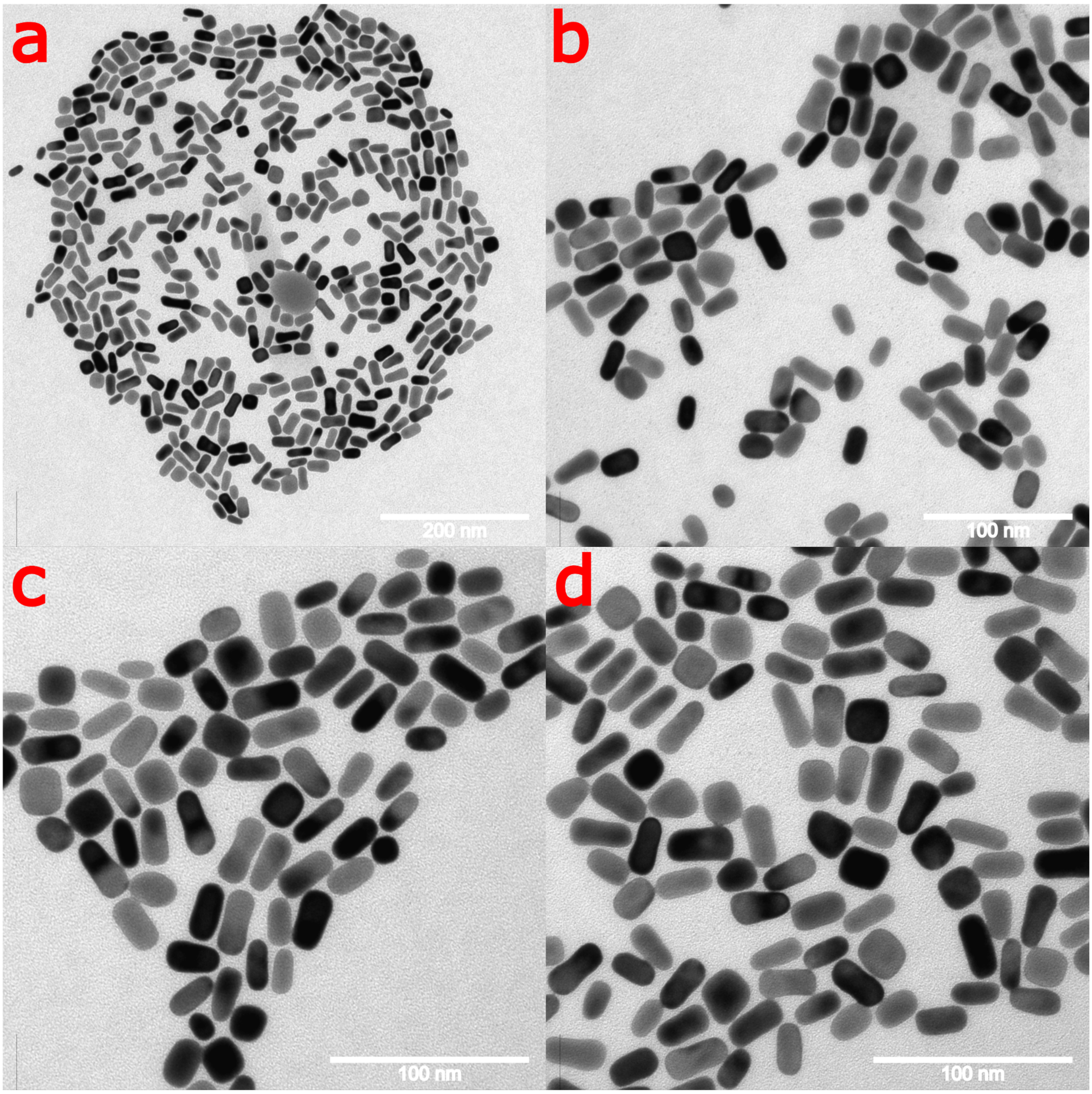

Figure S40. The TEM images for sample R2 with a low aspect ratio (as labelled as L1-3 in the manuscript). The scale bars are shown in the images.

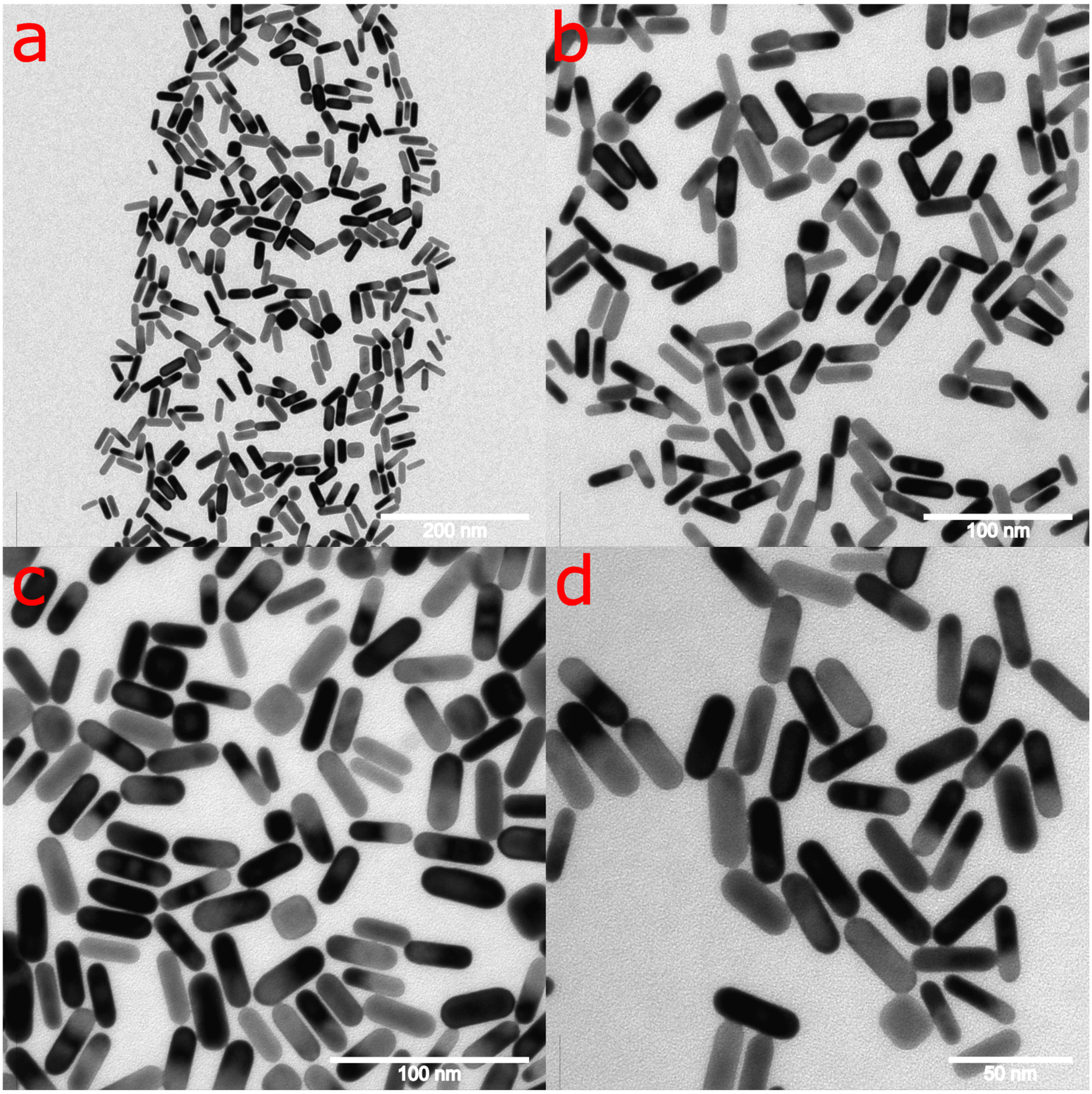

Figure S41. The TEM images for sample R3 with a medium aspect ratio (as labelled as L1-4 in the manuscript). The scale bars are shown in the images.

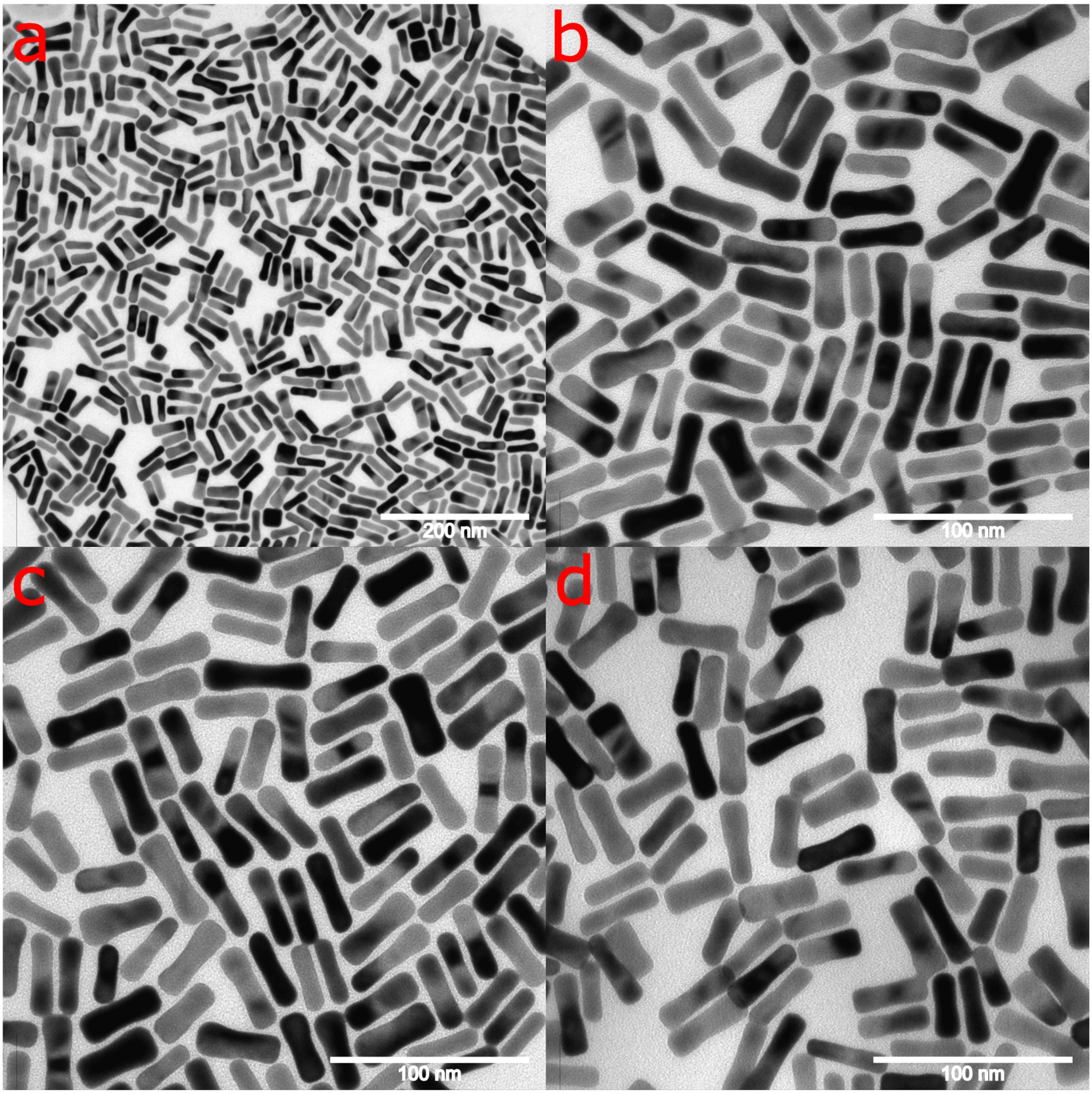

Figure S42. The TEM images for sample R4 (as labelled as L1-5 in the manuscript). This rod sample shows a concavity feature in the contour and will be used as the seed to explore the next chemical space. The scale bars are shown in the images.

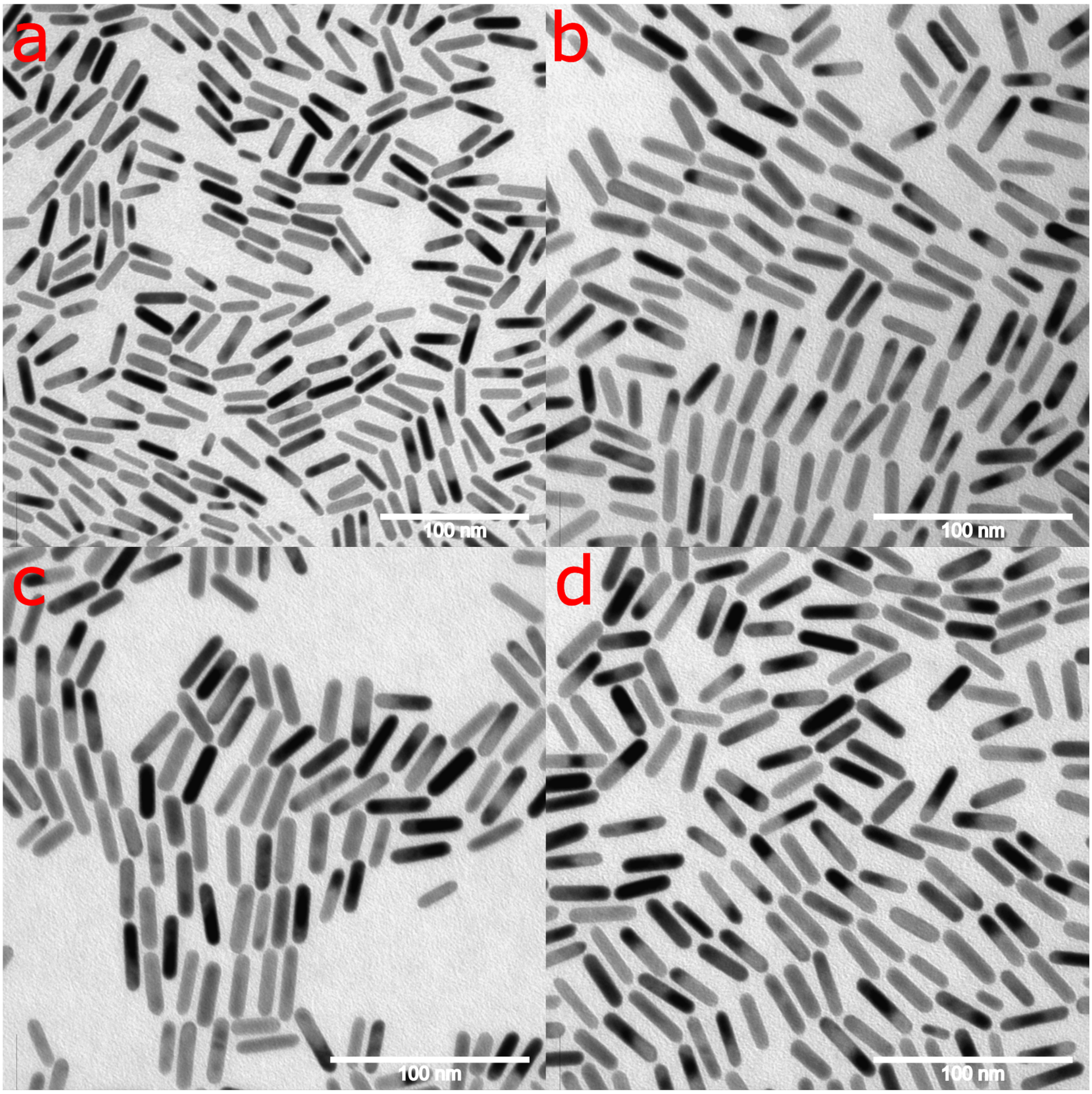

Figure S43. The TEM images for sample R5 with the highest aspect ratio (as labelled as L1-6 in the manuscript). The scale bars are shown in the images.

3.3. Chemical space 2: Overgrowth of Au nanorods

A sample of Au nanorods was used as the seed for the further exploration in the second chemical space, with an extra pH variable to influence the growth kinetics. The Au nanorod seed (R4, also labelled as L1-5 in the manuscript) with concave features was reproduced and used directly as the seed solution. Hydroquinone was used as the reductant, whose redox potential is sensitive to the solution pH and can influence the growth kinetics. In chemical space 1, the strong interactions within the multiple-peak systems but relatively weak interactions between the multiple-peak and single-peak systems were observed. To make the search focus on these two different systems respectively, the strategy was modified so that the exploration will only consider the multiple-peak systems first, then only the single-peak system, which means the exploration happens sequentially.

3.3.1. Experimental details

Synthesis of seed:

- Au nanorods (R4, also labelled as L1-5 in manuscript) were reproduced with the same conditions from studying the first chemical space and aged in 30°C for at least 90 minutes to complete the growth. The solution is used within 24 hours.

Synthetic procedure:

Each reaction was performed with the followed order of addition by the platform, using volumes and pH provided by the algorithm:

1. CTAB (0.2 M) added.
2. Hydroquinone (13.1 mM) added.
3. Water added to keep the overall volume as 7.00 mL.
4. pH measurement and control of the solution using HCl (0.1 M) and NaOH (0.1 M).
5. $AgNO_3$ (0.25 mM) added.
6. $HAuCl_4$ (0.86 mM) added.
7. 10 seconds allowed for the reduction to complete.
8. 0.50 mL of the Au nanorod solution (R4) added.

The pH was tuned to a required value (step 4) before adding any metallic salts because the nanoparticles can form spontaneously without any seed in very basic conditions and be adsorbed to the surface of the electrode, which can reduce the stability of the system. Before tuning the pH, the overall volume of the solution is constrained to a constant value, which can stabilize the liquid level and pH response. To enable the stability and reproducibility, the pH probe was calibrated daily with standard pH buffer solutions (4.0, 7.0 and 10.0). Before measuring the pH, the pH probe was immersed in the solution for 10 seconds to give enough time to reach the steady state.

In this chemical space, the pH was controlled with a proportional control logic with an overshoot mechanism for a given target pH (pH_T). It is described as follows:

1. The initial current pH (pH_C) was measured and the difference between it and the target was calculated via Eq. (28).
2. Depending on if the target pH is higher or lower than the current pH (pH_C), NaOH (0.1 M) or HCl (0.1 M) was added respectively. The volume (V_{add}) for the addition was calculated through the proportional control logic (Eq. (29)).
3. The current pH (pH_C) was measured again to see if the solution pH overshot the target pH after the addition. If it overshot, the proportional coefficient k to calculate the next addition volume would be reduced by a factor (f) via Eq. (30).
4. If the termination condition is reached, stop the tuning. Otherwise, go back to step 2. There are three cases for termination:
    a. The current pH was within a small range of the target pH ($pH_T \pm 0.2$). The current pH (pH_C) would be updated after 15 seconds and would still stay in this range (success).
    b. The overall time to tune the pH value was more than 2 minutes, which enables efficiency (failure).
    c. The total volume of the added acid and base was more than 3 mL, which avoids overflow (failure).

$$\Delta pH = pH_T - pH_C \tag{28}$$

$$V_{add} = k|\Delta pH| \tag{29}$$

$$k = f \times k \tag{30}$$

Step 2-4 was iterated until a termination condition is reached. We set the initial k to 25 µL and f to 0.6 in the experiment. The final actual pH was recorded and transformed to the input pH variable in the algorithms. Two test examples for the pH control are shown in **Figure S44a** and **b**. The target pHs and the final pHs in exploring chemical space 2 (460 samples in total) are shown in **Figure S44c** and **d**. The operations for the pH control in investigating chemical space 2 are recorded and used to reproduce the samples later.

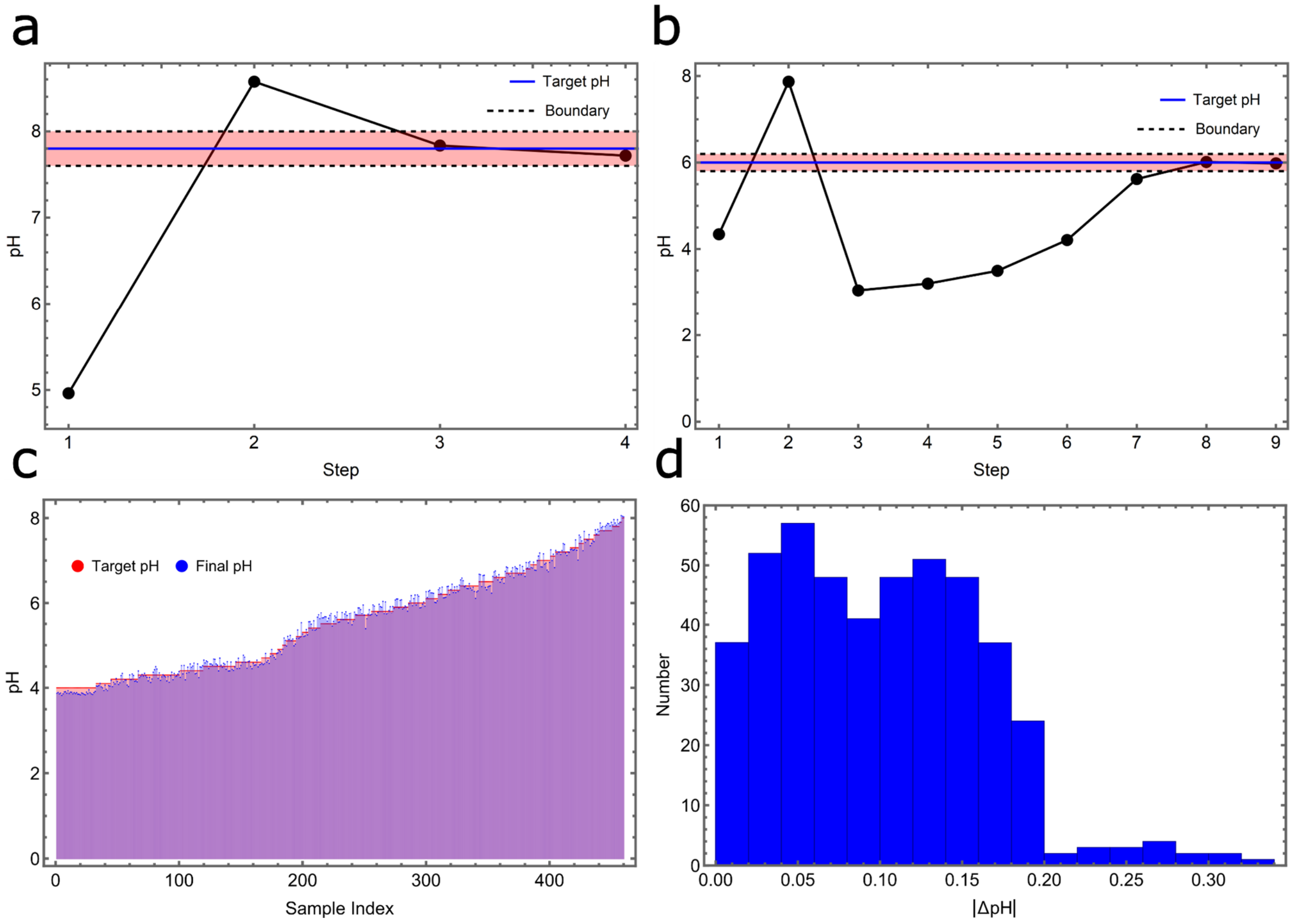

Figure S44. The pH control in chemical space 2. (a)-(b) Two examples of the pH control. The targets pHs are 7.80 and 6.00 respectively. The final pHs are 7.72 and 5.98 respectively. (c) The target pHs (red) and the corresponding final pHs (blue) from samples during exploring chemical space 2. The target pHs were sorted in ascending order. (d) The distribution of the absolute difference between target pH and final pH ($|\Delta pH|$). 443 (96%) samples have the final pH within 0.2 range of the target pH. The maximum difference between the target pH and the final pH is 0.32. Considering the difference, the final pH was transformed to the algorithm variable to design new experiments.

UV-Vis characterisation and data processing:

The same as described in chemical space 1.

TEM:

The same as described in chemical space 1.

Stability of the system:

Similar to that in chemical space 1, samples with the same synthetic condition were produced repeatedly throughout the exploration for each step. They served as standards to demonstrate the stability of the platform. Stock solutions can be reprepared during the repeats. The synthetic condition is listed in **Table S8**. The UV-Vis spectra of these standard samples were recorded to show the stability of the system (**Figure S45a** and **b**).

Standard	CTAB (mL)	Hydroquinone (mL)	Water (mL)	pH	$AgNO_3$ (mL)	$HAuCl_4$ (mL)	Seed (mL)
**2**	0.26	0.20	6.54	6.34	1.28	2.02	0.50

Table S8. The input parameters of the standard samples to test the stability of the autonomous platform in chemical space 2. The concentrations of the reagents are available in the same section above.

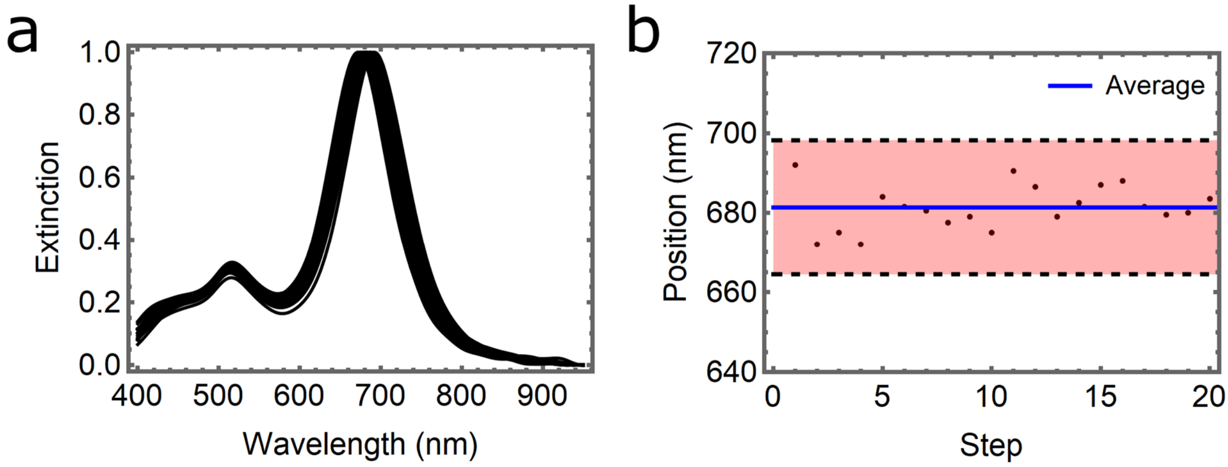

Figure S45. The demonstration of the stability of the platform. (a) The normalized UV-Vis spectra of the standard samples during exploration in different batches. (b) The highest peak positions from the standards. The mean value is 681 nm with a standard deviation of 5.6 nm. All peak positions are within 3 standard deviations of their mean value as indicated by the red background.

3.3.2. Data processing and algorithm

Input space, boundary conditions and sampling strategy

A linear transformation was applied to transform between volumes of chemicals and variables in the algorithm. These variables are always normalized between 0 and 1. The volume range for individual chemicals and pH are listed below:

- CTAB (0.2 M): 0 to 7.00 mL
- Hydroquinone (13.1 mM): 0 to 7.00 mL
- $HAuCl_4$ (0.86 mM): 0 to 5.00 mL
- $AgNO_3$ (0.25 mM): 0 to 5.00 mL
- Target pH: 4.0 to 8.0
- Boundary conditions: $v_{CTAB} + v_{HQ} \le 1$, $v_{HAuCl_4} + v_{AgNO_3} \le 1$and $v_i \in [0,1]$

where v_i is the normalized variable from a linear transformation from the volume of reagent i and also the solution pH. v_{HQ} means the variable corresponding to the volume of hydroquinone. Note that the boundary conditions were put so that the summations of v_{CTAB} and v_{HQ} as well as v_{HAuCl_4} and

v_{AgNO_3} are no larger than 1. In generating new experiments, the crossover, mutation and random sampling are the same as those discussed in **Section 3.2**.

Algorithm parameters

Among the 23 samples available per step, 10 samples were mutated from the elite (parent) set, 10 samples were from crossover among elites each of which had a 40% chance of mutation and 3 samples were randomly generated in the input chemical space. This distributed equal resources for crossover and mutation processes in each step. As the dimensionality of the chemical space is high, the relatively small initial random dataset (23) has the potential to bias the exploration, therefore a small portion of random sampling was added in every step to reduce the risk of bias. The standard deviations of the multi-dimensional Gaussian distribution were set to 0.08.

Exploration of the chemical space

The chemical space was explored by running 10 steps only considering the multiple-peak systems (with a random sampling number of 23 in the first step) and another 10 steps only considering the single-peak system. The exploration of the single-peak system was initialised with the data from the first 10 step exploration of the multiple-peak systems.

Classes and fitness

The classes were determined following the same procedure described for the single-peak system in the exploration of chemical space 1. For both single-peak and multiple-peak systems, from 400 nm to 600 nm the discretization was done with an interval of 25 nm and 600 nm to 950 nm with an interval of 50 nm.

The fitness (F) was defined in a similar way by considering the absorption band of the individual peak and its corresponding peak width. The fitness functions should guide multiple directions of exploration including:

1. For the single-peak system, the single peak should be sharp and dominant.
2. For the multiple-peak systems, the search should be making one of the peaks more dominant over the others (scenario 1), or two peaks comparable (scenario 2).

All peaks should be sharp to enable the monodispersity and purity of nanostructure populations. Based on these considerations, the fitness functions were defined as follows:

Single-peak system:

$$F = k_1 \frac{\int_{x_{peak1}-w}^{x_{peak1}+w} I_x dx}{\int I_x dx} - k_2\, w_1 \qquad (31)$$

where k_1 and k_2 are the coefficients to tune the importance of the individual terms. x_{peak1} is the peak position in the single-peak system and w defines the range of the absorption band and was set to 50 nm. w_1 is the peak width at its half prominence. We set $k_1 = 1$ and $k_2 = 0.002$ due to the scale of the two terms in the experiments.

Multiple-peak systems:

$$F = k_1 \left| \frac{\int_{x_{peak1}-w}^{x_{peak1}+w} I_x dx}{\int I_x dx} - \frac{\int_{x_{peak2}-w}^{x_{peak2}+w} I_x dx}{\int I_x dx} \right| - k_2(w_1 + w_2) \quad (32)$$

where x_{peak1} and x_{peak2} are the peak positions of the most prominent two peaks, w defines the absorption band near them and was set to 50 nm, w_1 and w_2 are the peak widths at half prominence for the peaks respectively and k_1 and k_2 are the coefficients controlling the importance of individual terms. The first term measures the difference between two peaks' absorption bands. Considering the scales and the relative importance of the two terms, we set $k_1 = 1$ and $k_2 = 0.002$ to amplify the difference in scenario 1, and $k_1 = -1$ and $k_2 = 0.002$ to minimize the difference between absorption bands in scenario 2.

3.3.3. Results and discussions

In chemical space 2, the pH-controlled overgrowth of Au nanorods was investigated (**Figure S46**). The pH value was tuned to influence the growth kinetics via a proportional control logic (**Figure S46a**) as discussed above. In chemical space 1, the interactions between the single-peak system and multiple-peak systems of either scenario, were weaker than those between the different scenarios of the multiple-peak systems, see Section "Chemical space 1: Seed-mediated synthesis on cuboctahedron single crystals" in the main text. The exploration was split into two stages:

1. Stage 1 consisted of 10 steps, in which the systems explored were only the multiple-peak systems with two different scenarios.
2. Stage 2 consisted of 10 steps also, in which the system explored was only the single-peak system. These 10 steps were initialised using all available data from stage 1.

Different elites were defined in these two stages respectively. The numbers of the elites belonging to multiple-peak systems in the first stage and the single-peak system in the second stage are shown in **Figure S46b** and **c**. The elites' UV-Vis spectra from both the multiple-peak systems, with two different scenarios, and the single-peak system are shown in **Figure S46d-f**.

Peak positions spreading from 500 nm to 900 nm were found in the exploration of the multiple-peak systems. The two scenarios that were explored discovered samples with similar peak positions however, the relative intensities of the peaks from one elite to another varied significantly (See Elite

9 & 26 with peaks ranges in 550-575 nm and 650-700 nm;10 & 27 with peak ranges in 500-525 nm and 700-750 nm as examples for comparison). The multiple-peak systems with different scenarios showed a strong interaction with crossover and mutation among elites to increase their fitness (**Figure S47a**). In the multiple-peak systems, the Au nanorod seed was found to transform into three types of nanorods with different aspect ratios (**Figure S48**-**Figure S57**) including:1. nanorods with spherical caps (Elite 6, 7 and 22 with TEMs shown in **Figure S48**, **Figure S49** and **Figure S57**, which correspond to L2-1, L2-2 and L2-10 in the manuscript respectively); 2. nanorods with rectangular caps (Elite 8, 10, 13 and 17 with TEMs shown in **Figure S50**, **Figure S52**, **Figure S54** and **Figure S56**, which correspond to L2-3, L2-5, L2-7 and L2-9 in the manuscript respectively); 3. Irregular nanorods similar to dog bones (Elite 9, 11 and 14 with TEMs shown in **Figure S51**, **Figure S53** and **Figure S55**, which correspond to L2-4, L2-6 and L2-8 in the manuscript respectively). The synthetic conditions for the nanoparticles in the multiple-peak systems are listed in **Table S9**.

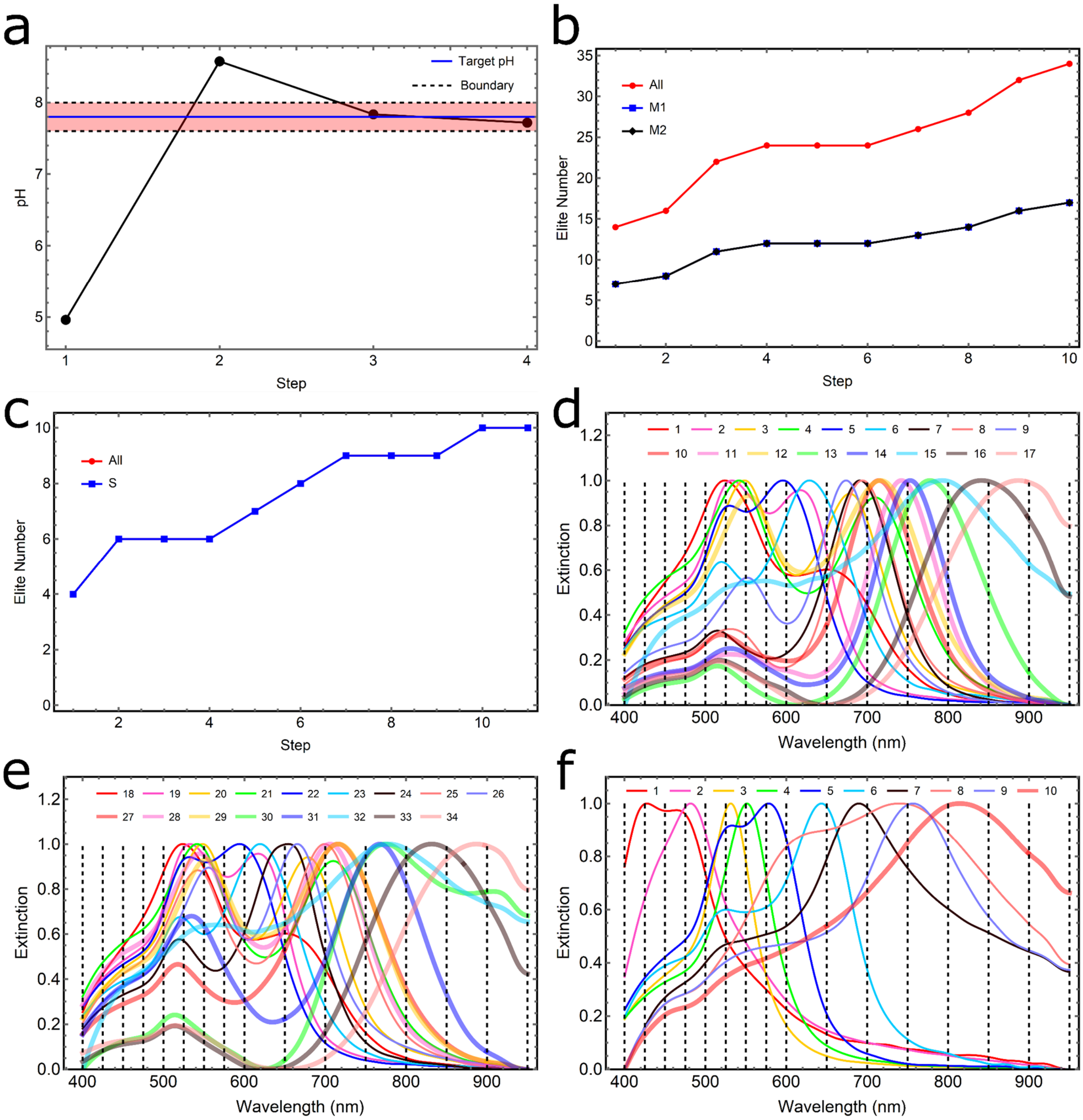

Figure S46. The exploration of chemical space 2. (a) An example of controlling pH via the proportional algorithm to reach the target pH. (b) The number of elites found via different steps in the 10 steps only considering the multiple-peak systems in stage 1. (c) The number of elites found via different steps in the 10 steps only considering the single-peak system in stage 2. S, M1 and M2 were used to indicate the single-peak system and the multiple-peak systems of different scenarios including making a single peak dominant or two peaks comparable respectively. Note the multiple-peak systems of different scenarios have the same number of elites. (d) The final UV-Vis of the elites in the multiple-peak system scenario 1 after exploration. (e) The final UV-Vis of the elites in the multiple-peak system scenario 2 after exploration. (f) The final UV-Vis of the elites in the single-peak system after exploration. The boundaries to define the subregions for different classes were drawn by dotted lines in (d) to (f).

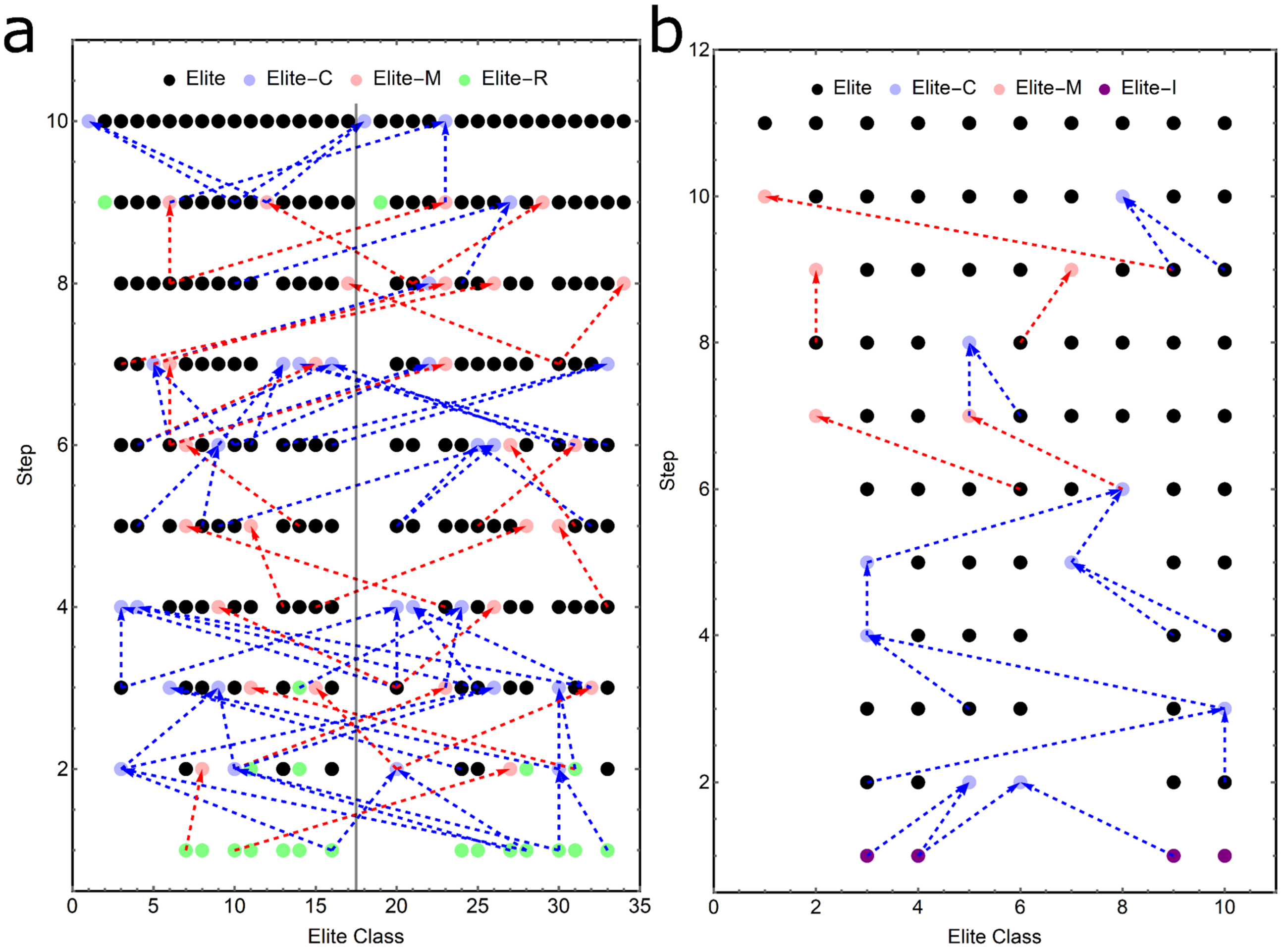

Figure S47. The evolution of the elites from different classes during exploring chemical space 2. In stage 1 (a), Elite, Elite-C, Elite-M and Elite-R correspond to elites without any change, elites from the crossover, elites from the mutation and elites from random sampling from the last step respectively. In stage 2 (b), Elite-I indicates the elites from the initial data set of the multiple-peak system exploration and there is no random sampling contributing to the exploration. In the multiple-peak system exploration, Elite 1 to 17 belong to the scenario 1 and 18-34 to scenario 2.

Class Index	CTAB (mL)	Hydroquinone (mL)	Water (mL)	pH	$AgNO_3$ (mL)	$HAuCl_4$ (mL)	Seed (mL)
**1**	1.93	5.07	0.00	7.90	0.69	0.51	0.50
**2**	2.54	3.96	0.50	7.10	0.13	3.63	0.50
**3**	0.26	6.23	0.51	4.03	2.69	2.31	0.50
**4**	0.04	6.36	0.60	4.17	2.47	1.55	0.50
**5**	2.63	1.54	2.83	4.61	0.45	3.32	0.50
**6 (L2-1)**	5.21	1.67	0.12	6.09	0.48	2.76	0.50
**7 (L2-2)**	0.13	3.00	3.87	4.24	0.00	0.85	0.50
**8 (L2-3)**	0.27	0.76	5.97	5.64	1.78	3.22	0.50
**9 (L2-4)**	0.44	5.88	0.68	4.16	1.76	2.73	0.50
**10 (L2-5)**	2.63	4.15	0.22	7.68	0.20	1.42	0.50

Class Index	CTAB (mL)	Hydroquinone (mL)	Water (mL)	pH	$AgNO_3$ (mL)	$HAuCl_4$ (mL)	Seed (mL)
**11 (L2-6)**	4.17	2.54	0.29	4.67	2.33	0.46	0.50
**12**	0.08	6.92	0.00	4.35	2.57	1.52	0.50
**13 (L2-7)**	4.17	2.54	0.29	4.22	2.30	2.70	0.50
**14 (L2-8)**	2.48	1.24	3.28	6.10	3.54	0.82	0.50
**15**	0.00	6.39	0.61	4.34	2.25	1.32	0.50
**16**	2.76	1.65	2.59	4.09	2.40	1.55	0.50
**17 (L2-9)**	3.47	0.99	2.54	4.53	2.38	1.68	0.50
**18**	1.93	5.07	0.00	7.90	0.69	0.51	0.50
**19**	2.54	3.96	0.50	7.10	0.13	3.63	0.50
**20**	0.26	6.23	0.51	4.03	2.69	2.31	0.50
**21**	0.04	6.36	0.60	4.17	2.47	1.55	0.50
**22 (L2-10)**	2.63	1.54	2.83	4.36	0.34	2.95	0.50
**23**	5.41	1.59	0.00	6.37	0.41	2.30	0.50
**24**	2.75	4.25	0.00	4.56	0.00	1.20	0.50
**25**	0.26	6.36	0.38	3.88	2.47	1.70	0.50
**26**	0.21	6.79	0.00	3.91	2.23	2.76	0.50
**27**	2.75	4.25	0.00	7.81	0.00	1.20	0.50
**28**	0.04	6.71	0.25	4.52	2.46	1.49	0.50
**29**	0.08	6.92	0.00	4.35	2.57	1.52	0.50
**30**	2.69	1.47	2.84	3.86	2.40	1.96	0.50
**31**	0.09	1.24	5.67	6.22	3.54	0.82	0.50
**32**	0.00	6.36	0.64	4.24	2.47	1.70	0.50
**33**	4.44	2.37	0.19	4.63	2.62	2.23	0.50
**34**	3.47	0.99	2.54	4.53	2.38	1.68	0.50

Table S9. The input parameters of the elites in chemical space 2 after exploring the multiple-peak systems. The concentrations of the reagents are available in the same section above. The pH is the final reached experimental value after pH control.

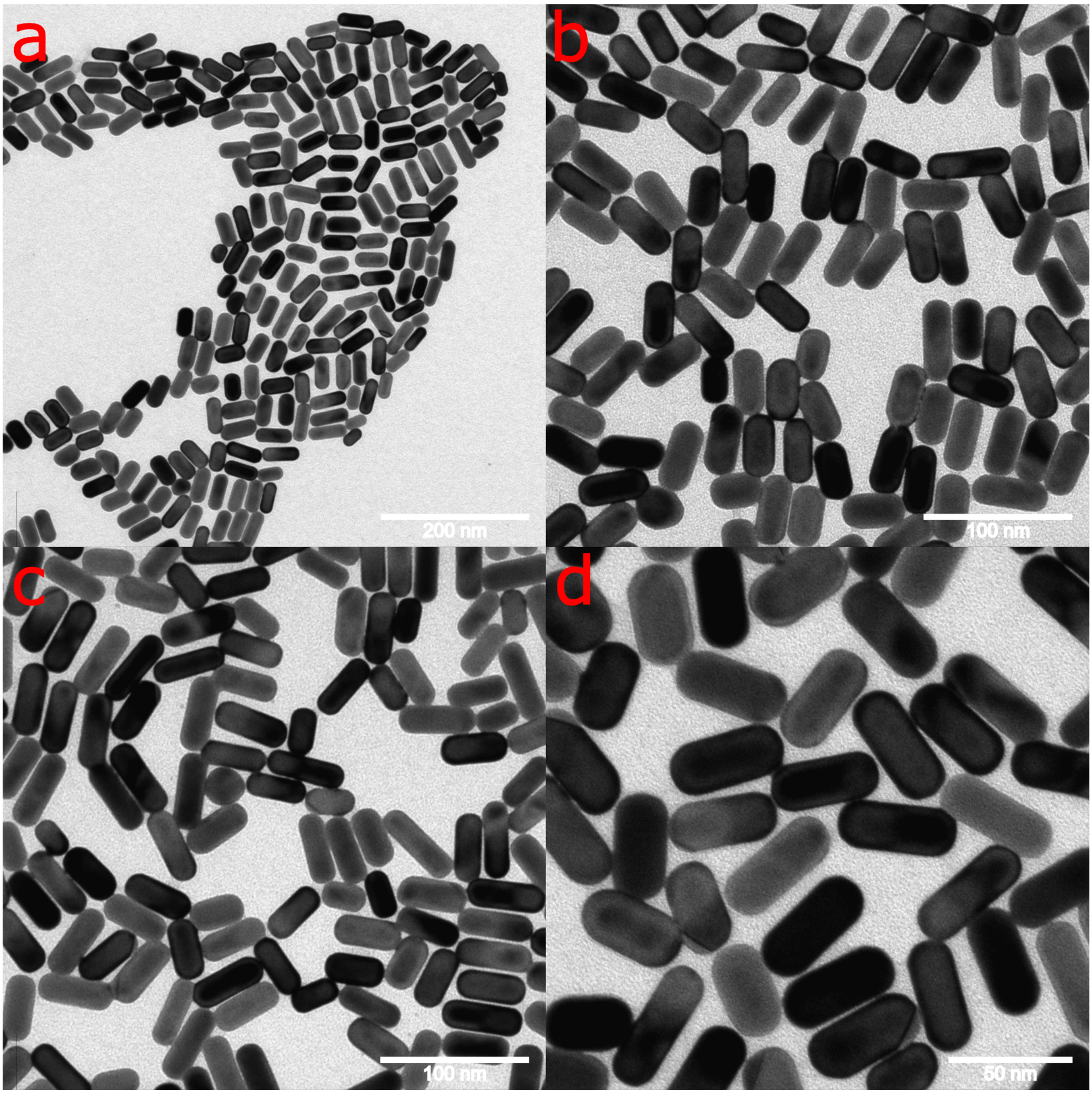

Figure S48. The TEM images of nanorods with spherical caps (Elite 6 in the multiple-peak systems, also labelled as L2-1 in the manuscript). The scale bars are shown in the images.

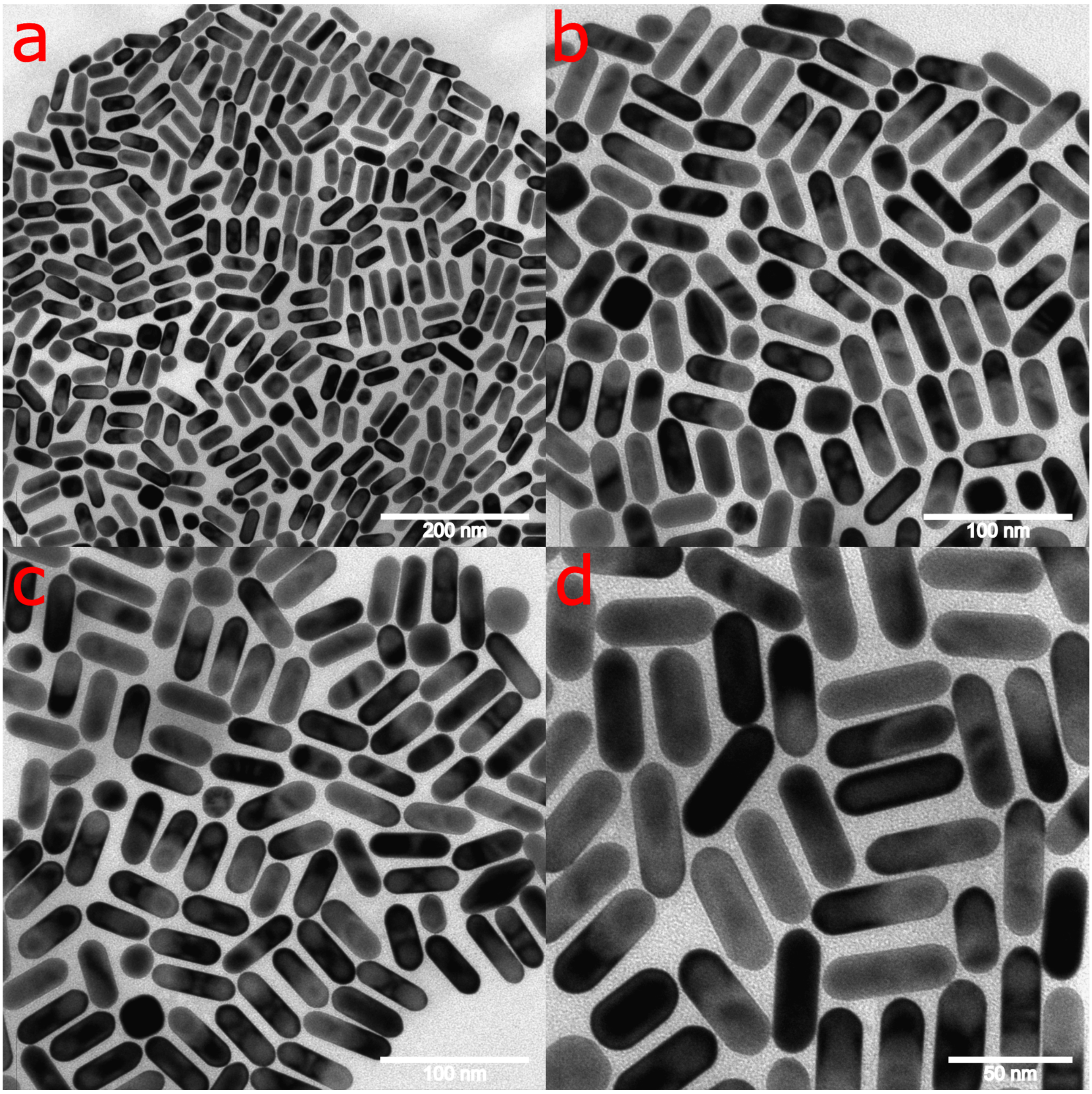

Figure S49. The TEM images of nanorods with spherical caps (Elite 7 in the multiple-peak systems, also labelled as L2-2 in the manuscript). The scale bars are shown in the images.

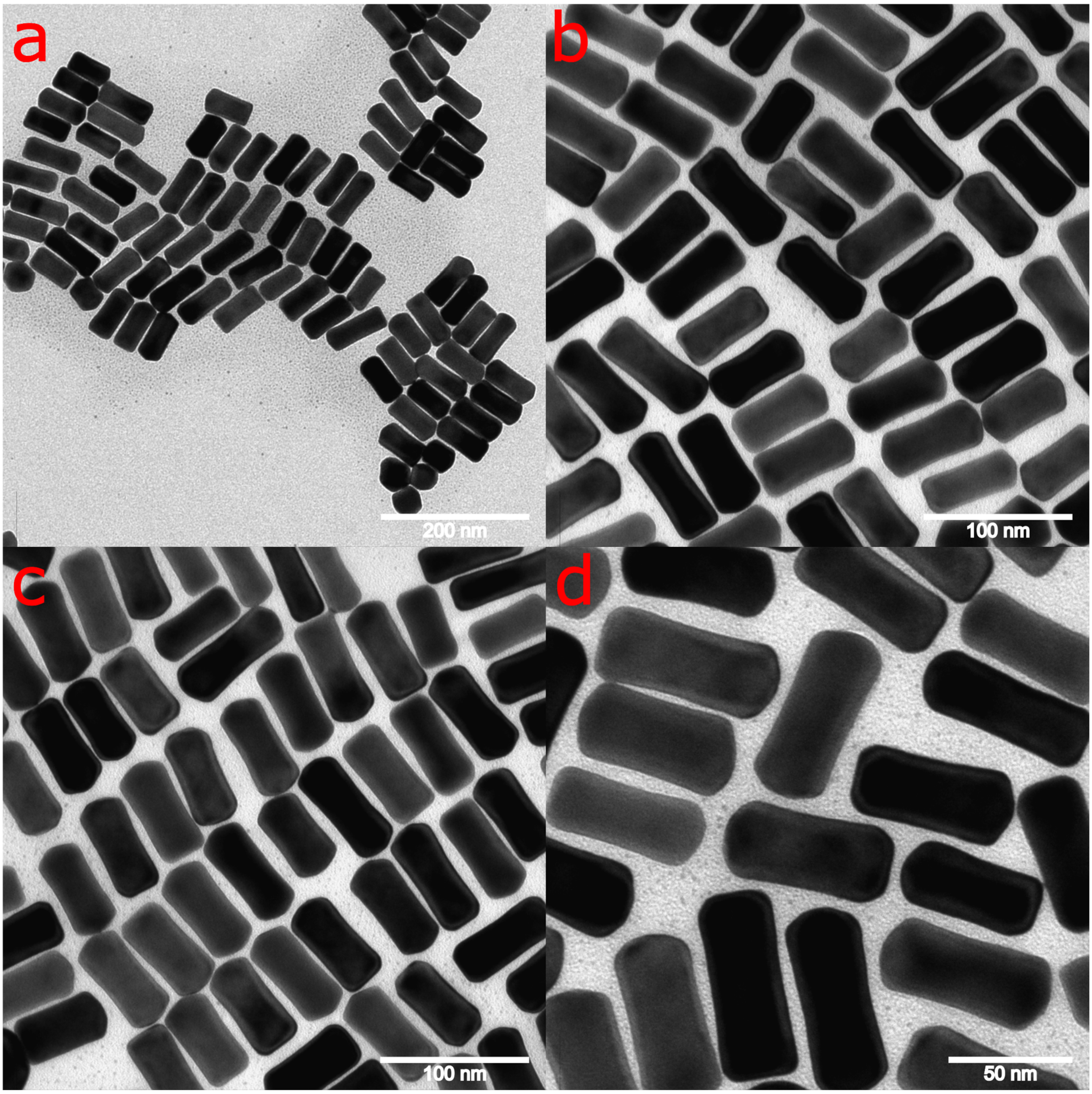

Figure S50. The TEM images of nanorods with rectangular caps (Elite 8 in the multiple-peak systems, also labelled as L2-3 in the manuscript). The scale bars are shown in the images.

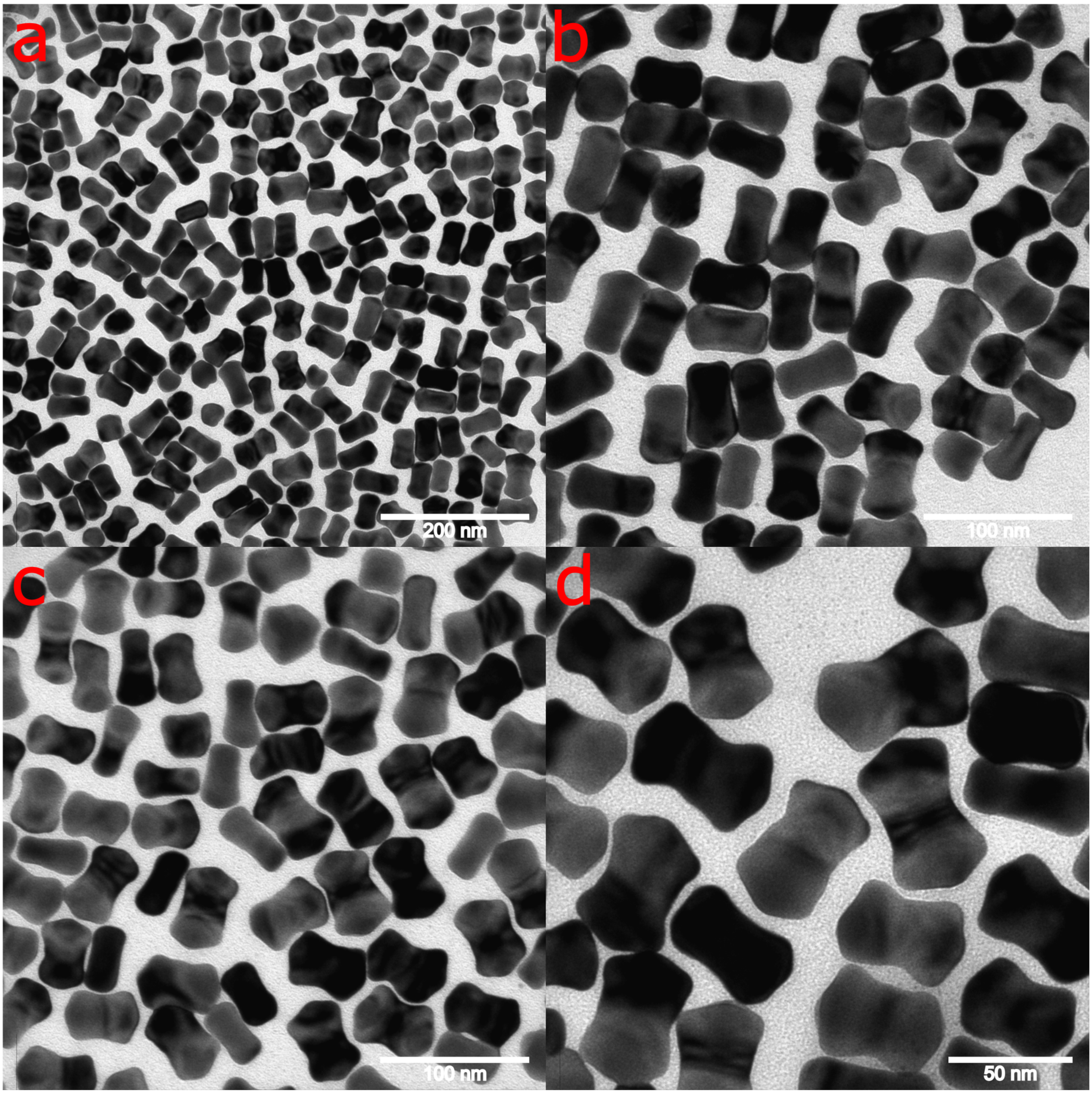

Figure S51. The TEM images of dog bones (Elite 9 in the multiple-peak systems, also labelled as L2-4 in the manuscript). The scale bars are shown in the images.

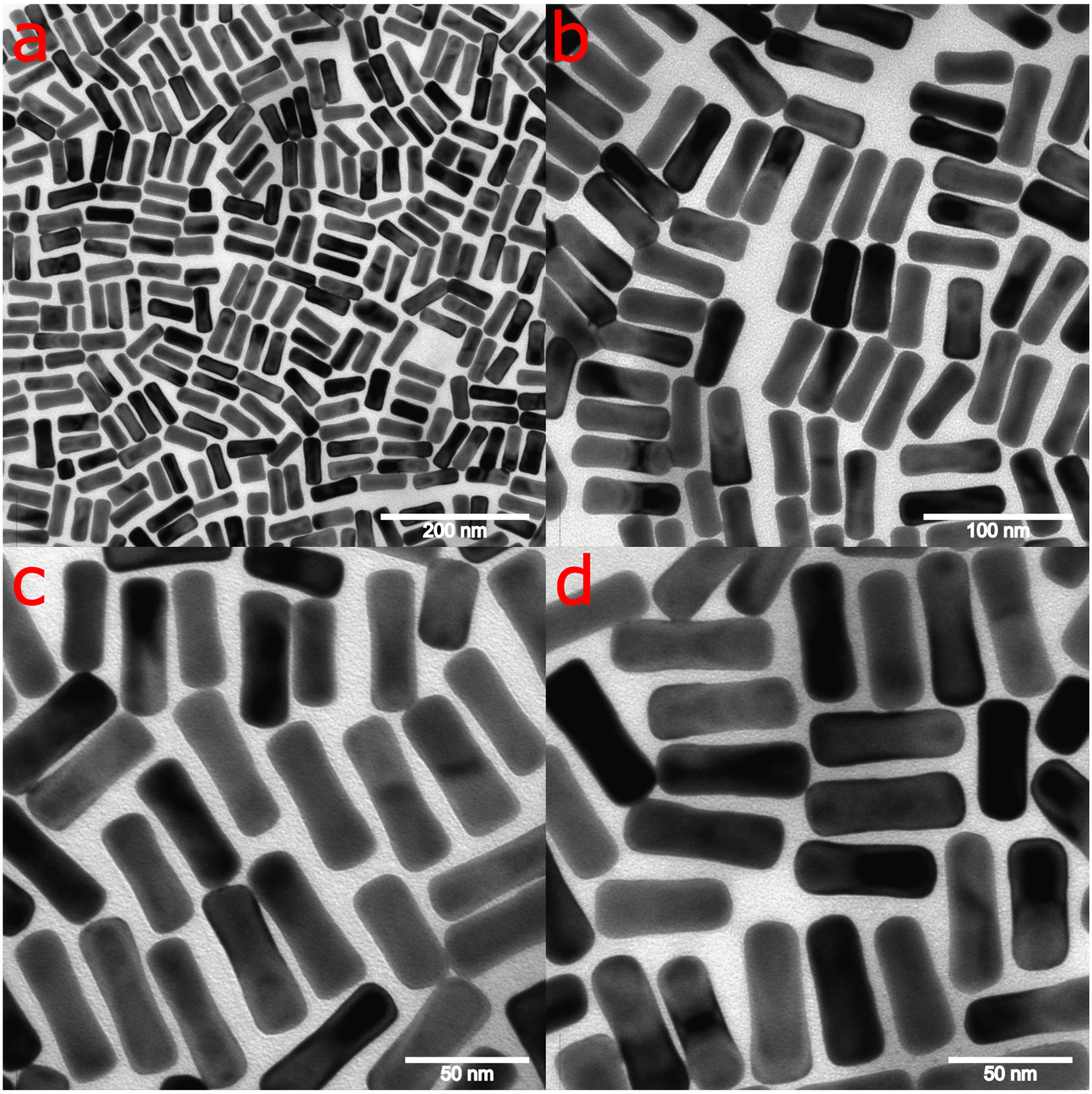

Figure S52. The TEM images of nanorods with rectangular caps (Elite 10 in the multiple-peak systems, also labelled as L2-5 in the manuscript). The scale bars are shown in the images.

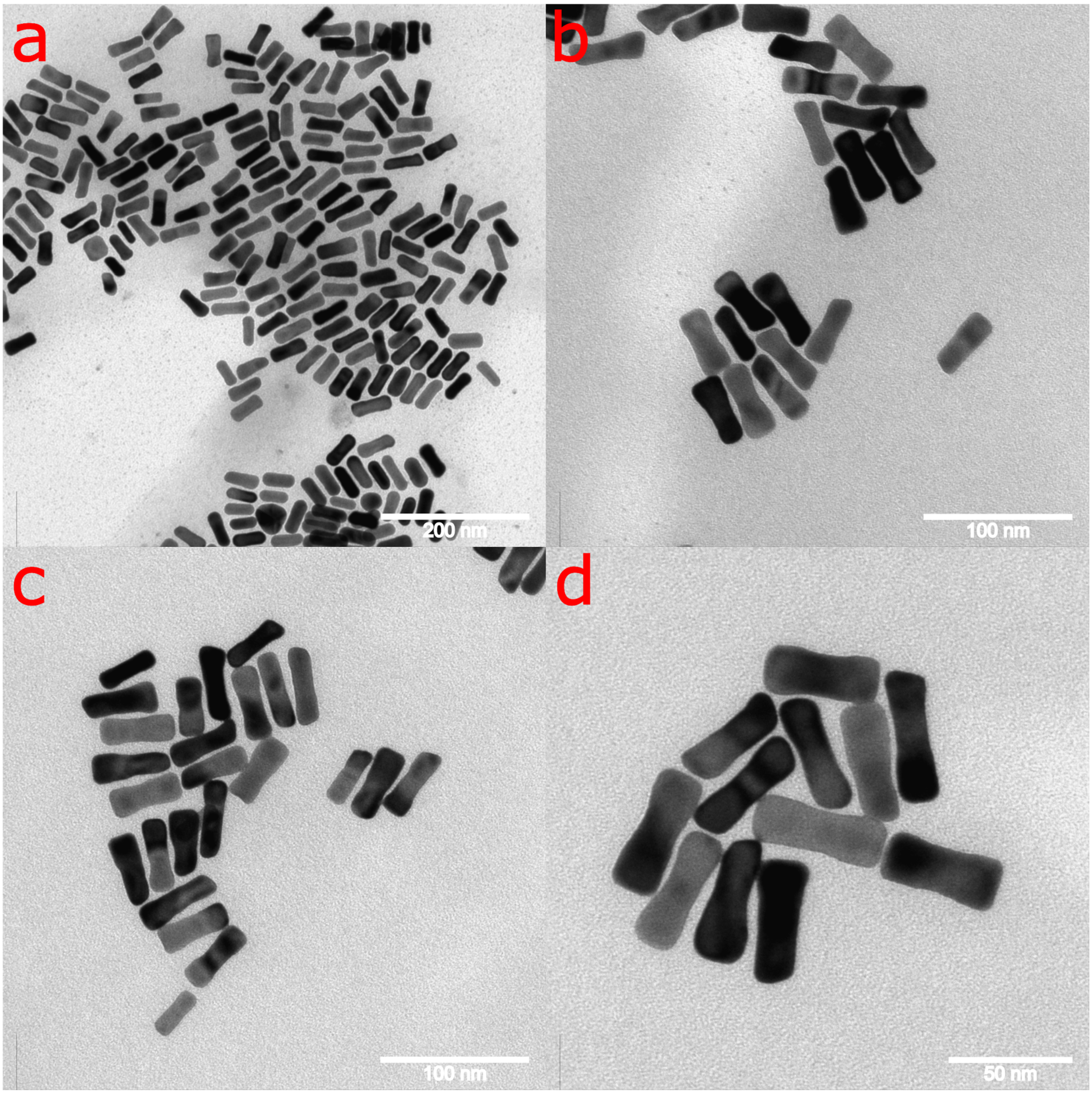

Figure S53. The TEM images of dog bones (Elite 11 in the multiple-peak systems, also labelled as L2-6 in the manuscript). The scale bars are shown in the images.

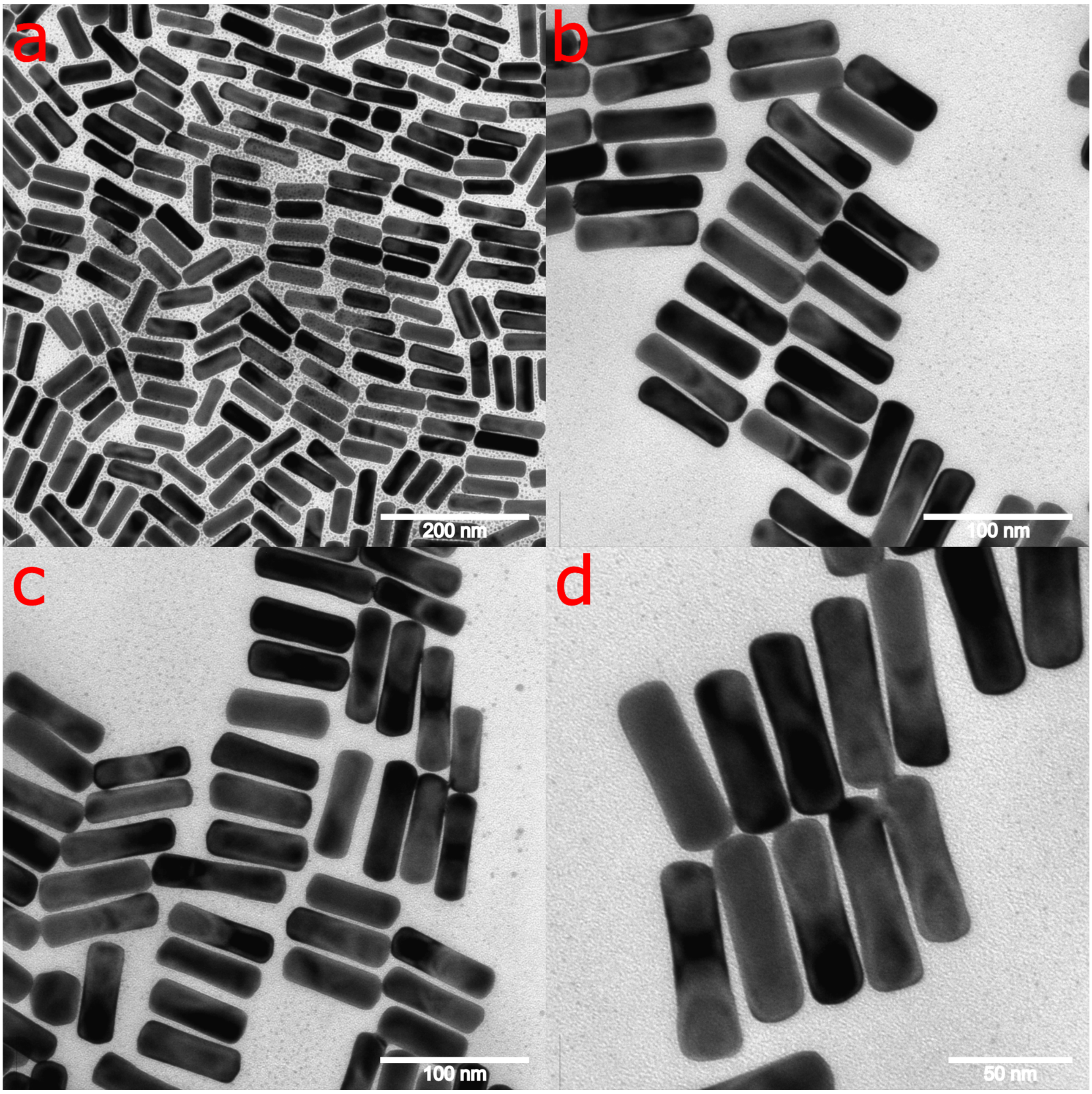

Figure S54. The TEM images of nanorods with rectangular caps (Elite 13 in the multiple-peak systems, also labelled as L2-7 in the manuscript). The scale bars are shown in the images.

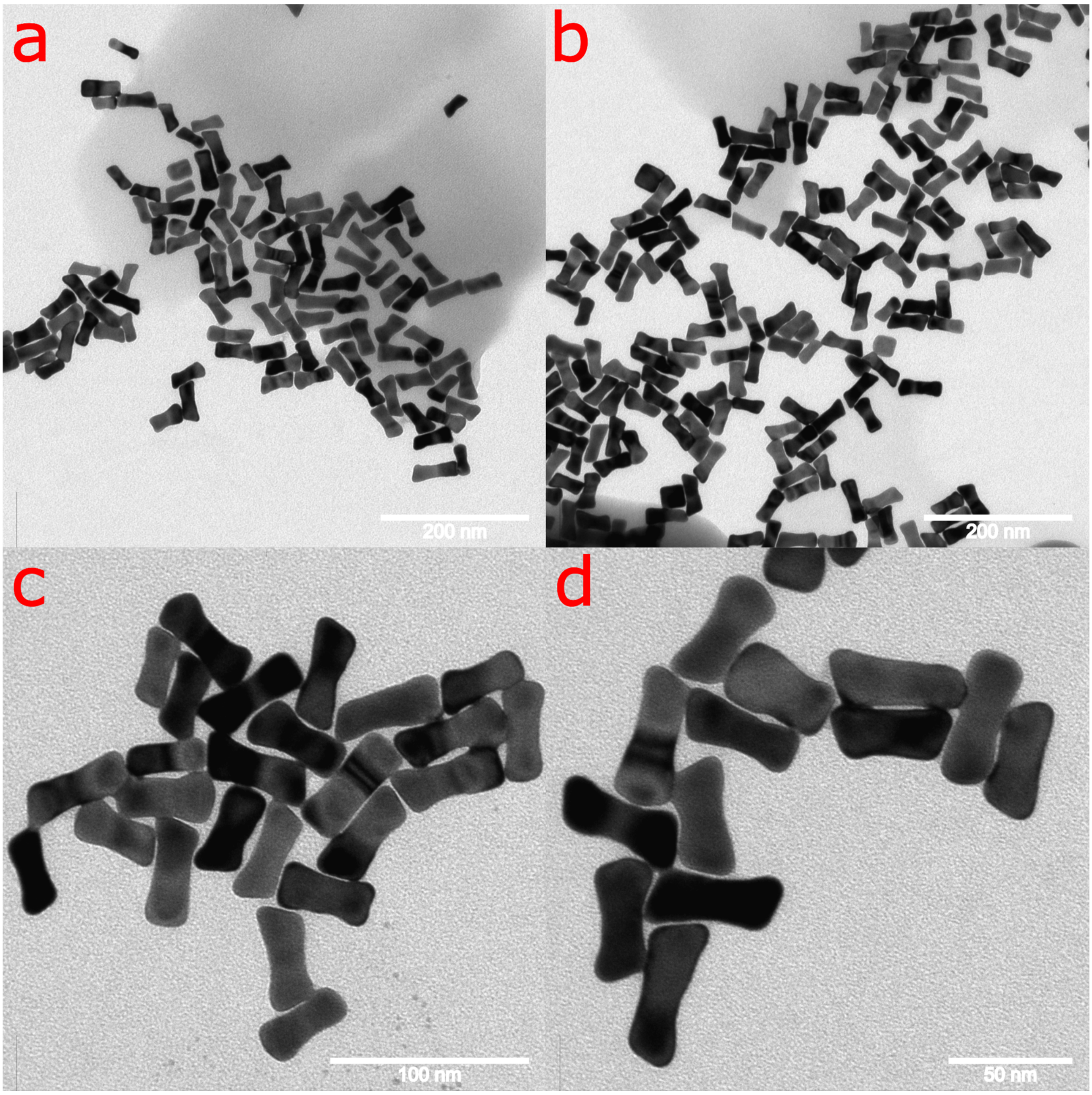

Figure S55. The TEM images of dog bones (Elite 14 in the multiple-peak systems, also labelled as L2-8 in the manuscript). The scale bars are shown in the images.

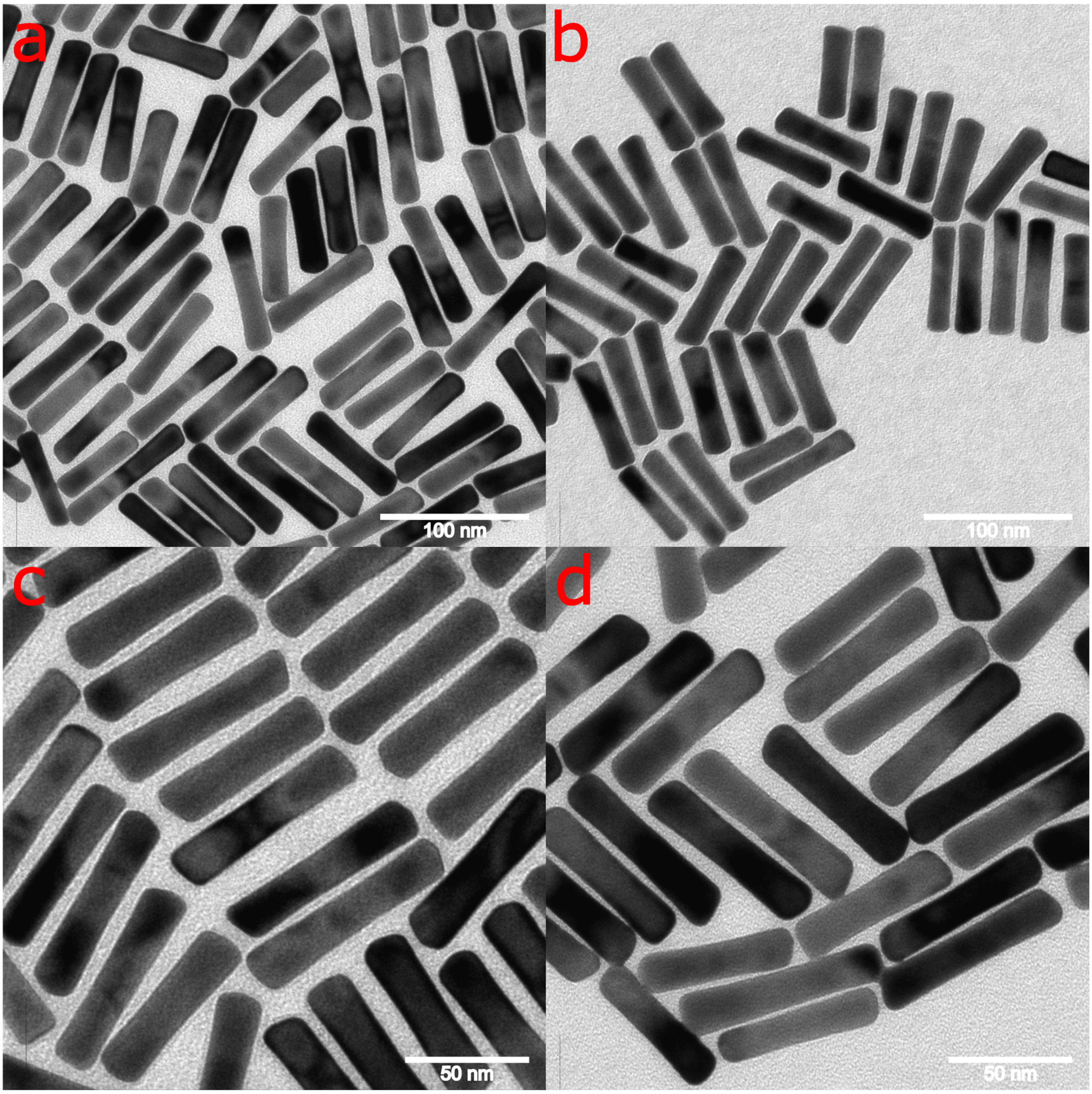

Figure S56. The TEM images of nanorods with rectangular caps (Elite 17 in the multiple-peak systems, also labelled as L2-9 in the manuscript). The scale bars are shown in the images.

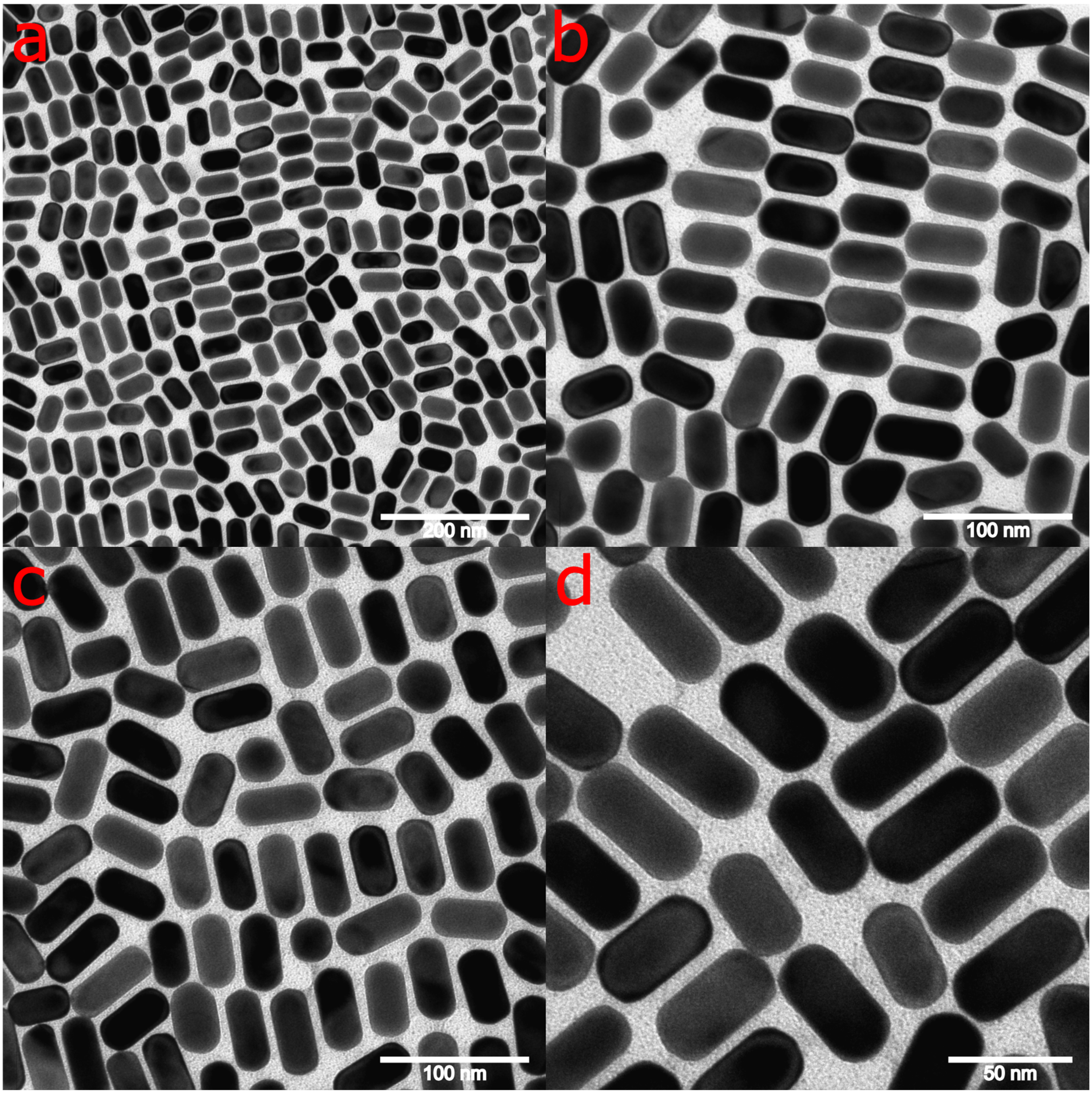

Figure S57. The TEM images of nanorods with spherical caps (Elite 22 in the multiple-peak systems, also labelled as L2-10 in the manuscript). The scale bars are shown in the images.

The single-peak system was initialised utilising the data from stage 1. The initial elite number was 4 and it increased to 10 after stage 2. Stage 2 exploration was purely driven by the crossover and mutation of the elites as shown in **Figure S47b**. In the single-peak system, the peak position can vary from 400 nm to 820 nm and the goal of the fitness was to make this peak more dominant. Samples of elites 6-10 showed strong aggregation and/or over-reduction to bulk gold, which would result in a cloudy solution with a large, broadened UV-Vis peak. Elites 1 and 2 that produced peak positions below 525 nm were found where a very small volume reductant was added to reduce $HAuCl_4$. We characterised the remaining elites and found uniquely-shaped AuNPs of spherical polyhedra (**Figure S58**), bicones (**Figure S59**) and low aspect-ratioed rods (**Figure S60**), with the spherical polyhedra

and bicones transforming into smooth spheres (**Figure S61** and **Figure S62**) after leaving them overnight (≈ 16 hours). Then the Au nanospheres from bicones with a peak position around 530 nm (**Figure S62**) were used as the seeds for further exploration (chemical space 3), which will be discussed in detail in the next section. The synthetic conditions for the elites found in the single-peak system are shown in **Table S10**.

**Class Index**	**CTAB (mL)**	**Hydroquinone (mL)**	**Water (mL)**	**pH**	**$AgNO_3$ (mL)**	**$HAuCl_4$ (mL)**	**Seed (mL)**
**1**	0.07	0.24	6.69	5.91	1.29	2.76	0.50
**2**	0.09	0.00	6.91	6.91	1.31	2.85	0.50
**3 (L2-11-1)**	2.95	1.15	2.90	4.30	0.00	2.65	0.50
**4 (L2-12-1)**	1.85	1.07	4.08	6.68	0.07	3.09	0.50
**5 (L2-13)**	0.17	0.51	6.32	6.45	0.84	3.07	0.50
**6**	0.01	0.34	6.65	6.38	1.32	2.65	0.50
**7**	0.00	0.35	6.65	6.40	1.85	2.16	0.50
**8**	0.00	1.20	5.80	4.32	0.00	3.22	0.50
**9**	0.00	0.76	6.24	5.78	1.78	3.22	0.50
**10**	0.00	1.20	5.80	4.25	0.00	2.65	0.50
**L2-11-2**	2.95	1.15	2.90	4.30	0.00	2.65	0.50
**L2-12-2**	1.85	1.07	4.08	6.68	0.07	3.09	0.50

Table S10. The input parameters of the elites in chemical space 2 after exploring the single-peak system. The concentrations of the reagents are available in the same section above. The pH is the final reached experimental value after pH control. Note after leaving L2-11-1 and L2-12-1 for 16 hours, they converted to L2-11-2 and L2-12-2 respectively.

Note Elite 5 has one dominant peak and a small shoulder peak which is under the threshold to be regarded as a separate peak. The sample is composed of low aspect-ratio nanorods with a longitudinal peak around 575 nm (**Figure S60**).

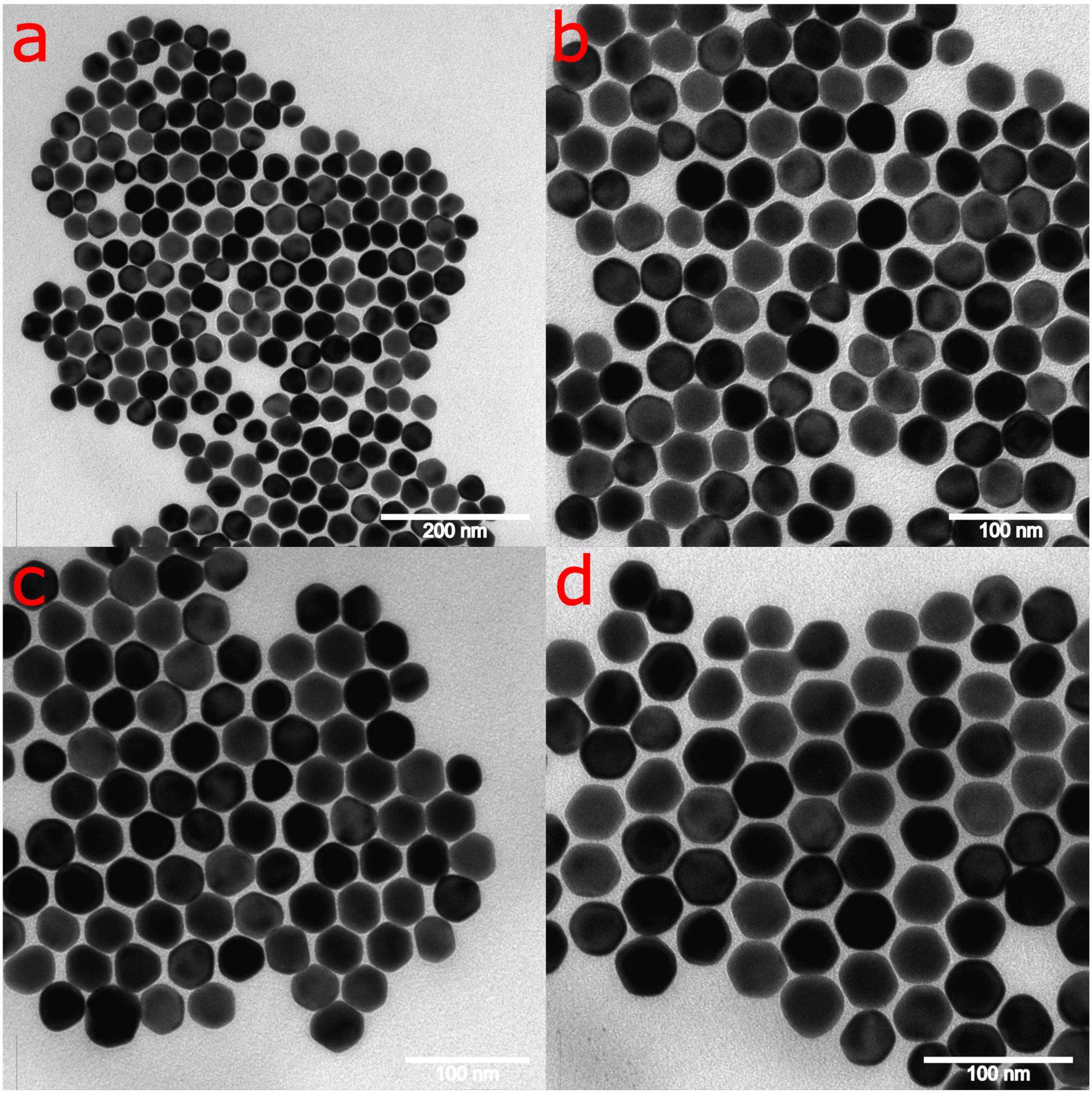

Figure S58. The TEM images of spherical polyhedra (Elite 3 in the single-peak system, also labelled as L2-11-1 in the manuscript). The scale bars are shown in the images.

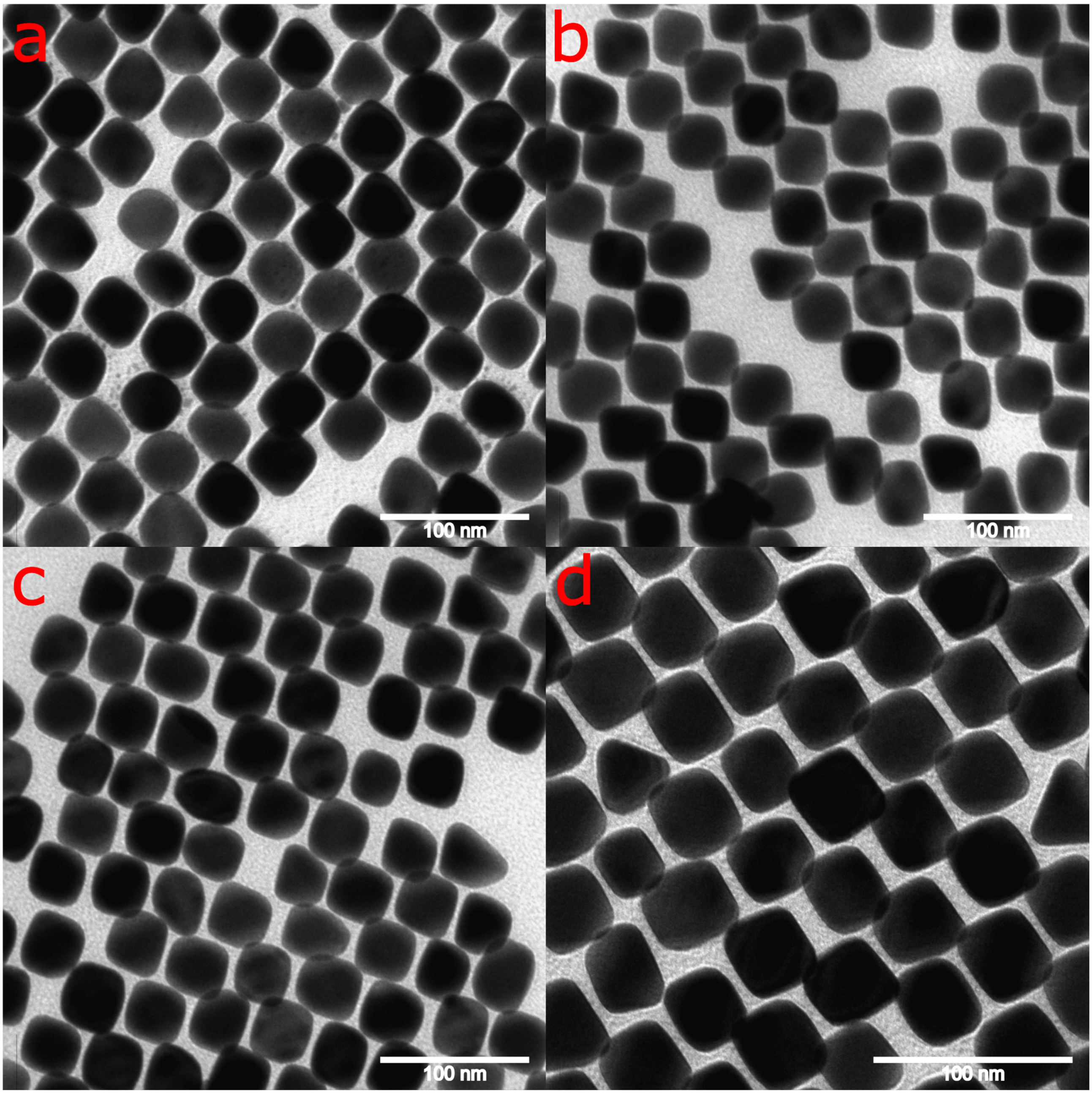

Figure S59. The TEM images of bicones (Elite 4 in the single-peak system, also labelled as L2-12-1 in the manuscript). The scale bars are shown in the images.

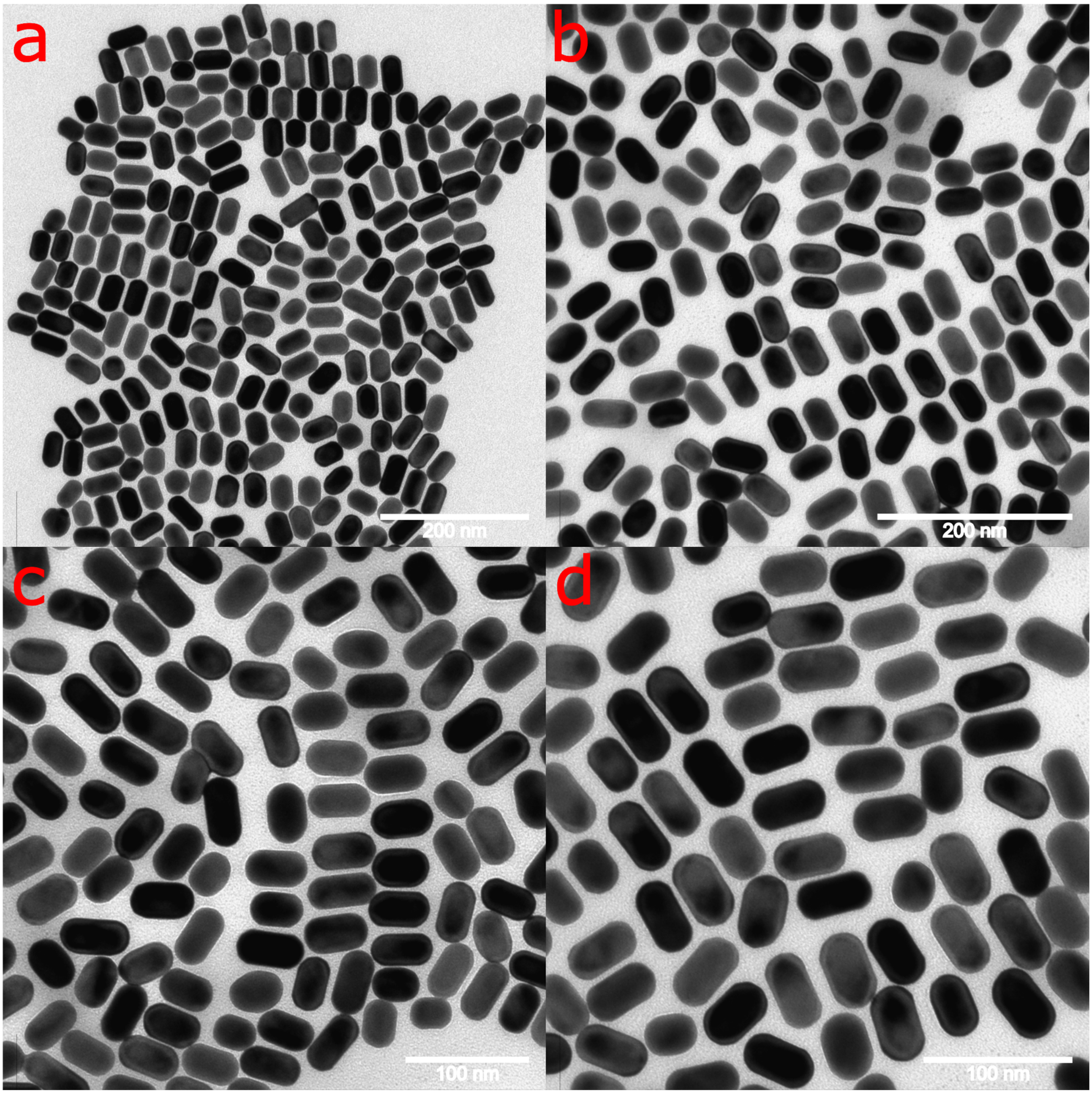

Figure S60. The TEM images of nanorods with a low aspect ratio (Elite 5 in the single-peak system, also labelled as L2-13 in the manuscript). The scale bars are shown in the images.

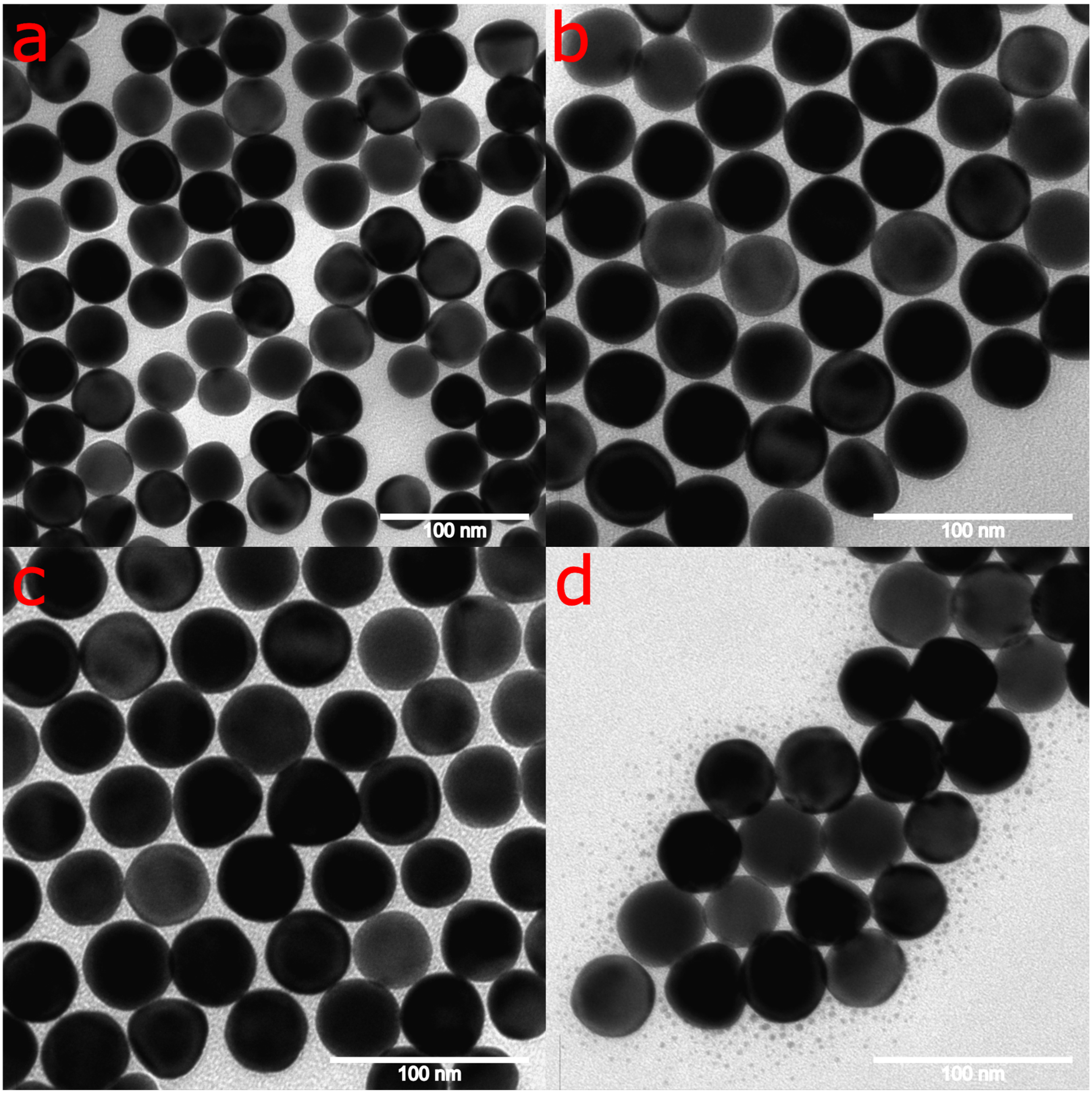

Figure S61. The TEM images of nanospheres from spherical polyhedra (also labelled as L2-11-2 in the manuscript). The scale bars are shown in the images.

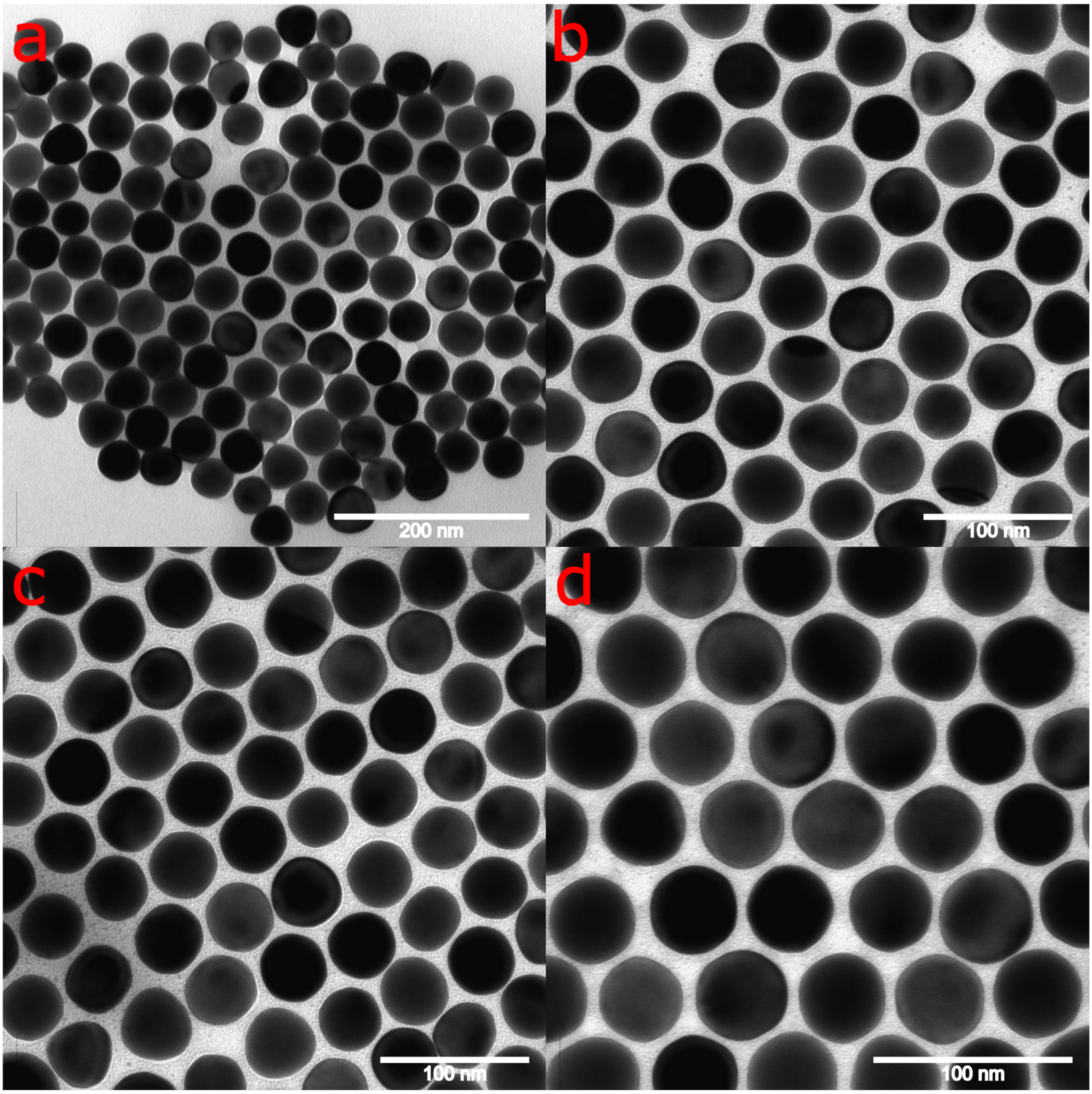

Figure S62. The TEM images of nanospheres from bicones (also labelled as L2-12-2 in the manuscript). The scale bars are shown in the images.

3.4. Chemical space 3: Overgrowth of Au nanospheres

The seed of the third chemical space is the Au nanosphere sample (L2-12-2, **Figure S62**) from chemical space 2. The five-dimensional input chemical space was defined by volumes of hexadecyl-trimethylammonium chloride (CTAC), $AgNO_3$, $HAuCl_4$, ascorbic acid and HCl. As a result, we managed to synthesise Au nanostars with different sizes and tip lengths.

3.4.1. Experimental details

Synthesis of the seed:

- Au spheres (L2-12-2) were reproduced using R4 (as labelled as L1-5 in the manuscript, see **Figure S42**) as the seed with the same conditions in the second chemical space by leaving it at 30°C to grow for around 16 hours.

Synthetic procedure:

Each reaction was performed with the following order of addition by the platform, using volumes provided by the algorithm:

1. CTAC (0.2 M) added.
2. $HAuCl_4$ (0.86 mM) added.
3. $AgNO_3$ (0.25 mM) added.
4. HCl (0.1 M) added.
5. Ascorbic acid (13.1 mM) added.
6. 10 seconds allowed for the reduction to complete.
7. Total volume constrained by addition of water if necessary.
8. 0.50 mL of the Au nanospheres solution (L2-12-2) as prepared in the second chemical space added.

The overall volume of the synthesised sample was constrained to 12.00 mL by adding Type I water, while the seed solution volume was kept as 0.50 mL. Note the concentration of ascorbic acid was four times as that in chemical space 1. These two solutions were prepared by dissolving 461.2 mg and 115.3 mg of ascorbic acid in 200 mL water respectively, thus with a concentration of ca. 13.1 mM and 3.3 mM.

UV-Vis characterisation and data processing:

The same as described in chemical space 1.

TEM:

The same as described in chemical space 1.

Stability of the system:

The standard samples were set to track the stability of the system. Unlike chemical space 1 and 2, no standard was set in the first step of random sampling due to the lack of knowledge of this chemical space. The stability of the system from the second step onwards was checked by selecting one sample from the first step as the standard sample. The UV-Vis spectra are shown in **Figure S63**. The synthetic condition for the standard sample is listed in **Table S11**.

Standard	CTAC (mL)	$HAuCl_4$ (mL)	$AgNO_3$ (mL)	HCl (mL)	Ascorbic acid (mL)	Water (mL)	Seed (mL)
**3**	1.57	2.86	1.37	0.60	5.00	0.10	0.50

Table S11. The input parameters of the standard samples to test the stability of the autonomous platform in chemical space 3. The concentrations of the reagents are available in the same section above. Note in chemical space 3, the concentration of ascorbic acid was four times as that in chemical space 1.

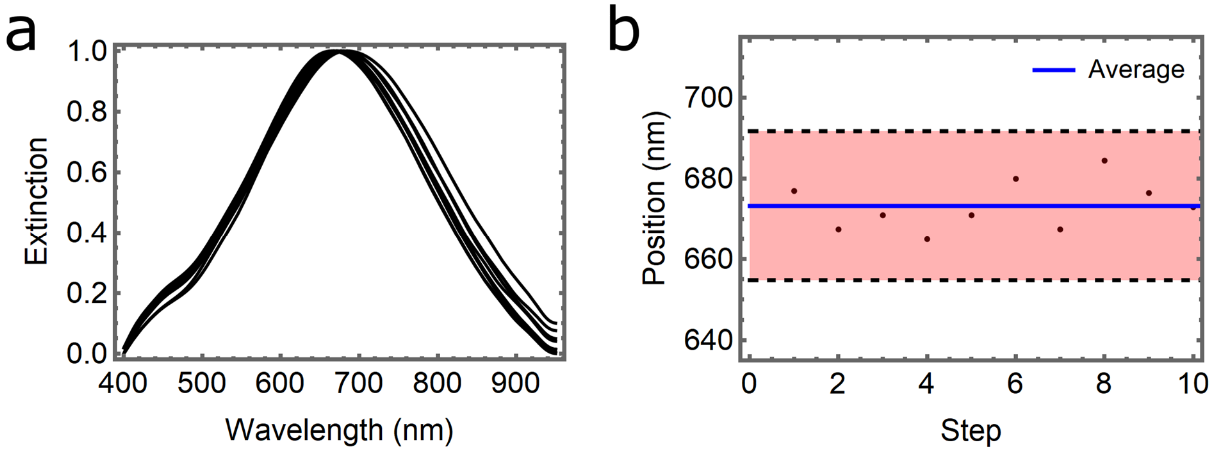

Figure S63. The demonstration of the stability of the platform. (a) The normalized UV-Vis spectra of the standard samples during exploration in different steps (including the original spectrum in the first step). (b) The different peak positions of the standard samples. The mean value is 673 nm with a standard deviation of 6.2 nm. All peak positions are within 3 standard deviations of their mean value as indicated by the red background.

3.4.2. Data processing and algorithm

The settings in chemical space 3 is almost the same as those in chemical space 2 with only modifications on the initial sampling number and classes which will be discussed below.

Input space, boundary conditions and sampling strategy

As in chemical space 1, a linear transformation is applied for the conversion between volumes of chemicals and variables in the algorithm. These variables are always normalized between 0 and 1. The volume range for individual chemicals and boundary conditions are listed below:

- CTAC (0.2 M): 0 to 11.50 mL

- $HAuCl_4$ (0.86 mM): 0 to 11.50 mL
- $AgNO_3$ (0.25 mM): 0 to 11.50 mL
- Ascorbic acid (13.1 mM): 0 to 11.50 mL
- HCl (0.1 M): 0 to 11.50 mL
- Boundary conditions: $\sum_i^n v_i \leq 1\ for\ v_i \in [0,1]$

where v_i is the normalized variable from a linear transformation from the volume of reagent i (CTAC, $HAuCl_4$, $AgNO_3$, ascorbic acid and HCl). The volume of the seed solution is fixed at 0.50 mL and extra water solution is added to constrain the overall volume (including seed solution) to be 12.00 mL.

Algorithm parameters

The initial random sampling number was 24 with no standard sample in the first step. The parameters for crossover, mutation and random sampling are the same as those in chemical space 2.

Exploration of the chemical space

Only the single-peak system was explored by 10 steps including the initial first random sampling step.

Classes and fitness

Only the single-peak system in this chemical space was explored. The classes were defined according to the subregion the single peak is located in, which is similar to the procedure described above in chemical space 2. The subregions were determined by putting 400 nm to 550 nm as a single subregion, discretizing 550 nm to 800 nm with an interval of 25 nm, and 800 nm to 950 nm with an interval of 50 nm respectively. This setting put more resources in exploring samples with a single peak in the range between 550 to 800 nm. The reason for choosing this approach is simply that the starting seed began with a signal at 530 nm, and therefore the overgrowth of this shape is most likely to exist around this range.

The fitness function is defined via Eq. (31) by setting $w = 50$ nm, $k_1 = 1$ and $k_2 = 0.002$ respectively.

3.4.3. Results and discussions

The results of exploring this chemical space are shown in **Figure S64**. By enabling the diversity of UV-Vis spectra, Au nanostars with different sizes and tip lengths were found. The UV-Vis peak positions spread from 560 nm to 800 nm. The total elite number at the conclusion of this exploration was 11. The synthetic conditions for the elites are listed in **Table S12**. Elite 1 was found where almost no reductant (0.06 mL) was added, thus it was not characterised by TEM. The rest of the elites were characterised by TEM as shown in **Figure S65**-**Figure S74**. Among them, Elite 2 and 3 showed a tendency to create octahedral features (**Figure S65** and **Figure S66**), and a small portion of Au octahedra was observed in Elite 3 (**Figure S66c**). These octahedral features will be further facilitated in the later optimisation discussed in the next section.

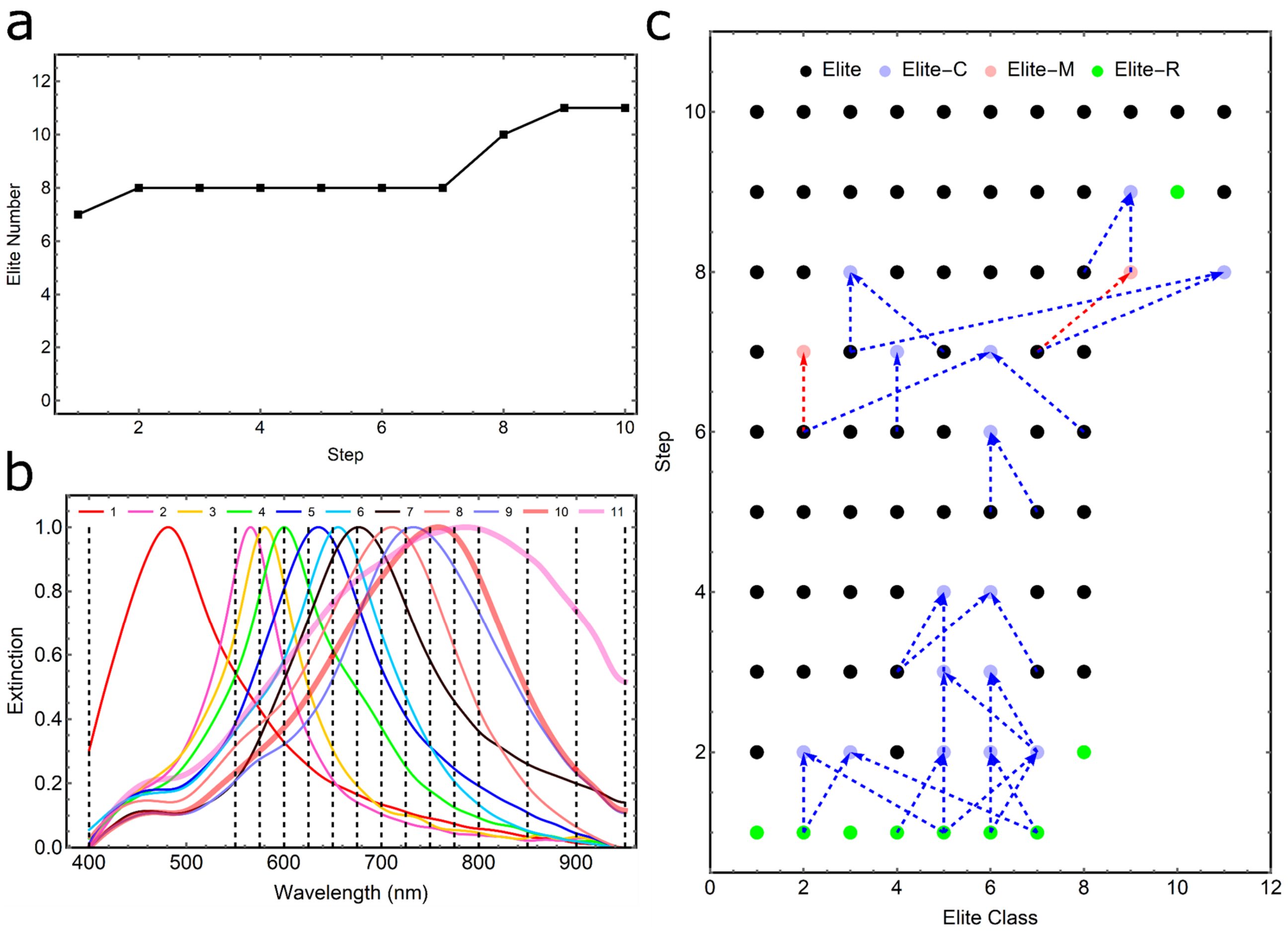

Figure S64. The exploration of the third chemical space based on Au nanospheres. (a) The number of elites found via different steps in the 10 steps. (b) The final UV-Vis of the elites with the boundaries of subregions to distinguish classes. (c) The evolution of elites via crossover, mutation and random sampling in the exploration. The red and blue arrows indicate the propagation of experimental conditions to a new elite through mutation and crossover. In (c), Elite, Elite-C, Elite-M and Elite-R correspond to elites without any change, elites from the crossover, elites from the mutation and elites from random sampling from the last step respectively.

Class Index	CTAC (mL)	$HAuCl_4$ (mL)	$AgNO_3$ (mL)	HCl (mL)	Ascorbic acid (mL)	Water (mL)	Seed (mL)
**1**	0.04	6.27	3.46	0.98	0.06	0.69	0.50
**2 (L3-1)**	2.66	0.32	0.00	0.74	5.25	2.53	0.50
**3**	0.21	0.29	0.42	2.18	3.82	4.58	0.50
**4**	2.24	1.54	4.01	2.18	0.33	1.20	0.50
**5**	2.24	1.54	0.02	2.18	3.82	1.70	0.50
**6 (L3-2)**	0.37	1.04	0.11	7.02	0.48	2.48	0.50
**7 (L3-3)**	6.81	1.92	0.03	1.19	0.60	0.95	0.50
**8 (L3-4)**	1.10	1.39	0.27	6.50	1.10	1.14	0.50
**9**	5.09	1.29	0.19	4.52	0.41	0.00	0.50
**10 (L3-5)**	3.78	2.49	0.80	3.58	0.54	0.31	0.50
**11**	7.72	2.24	0.12	0.28	1.14	0.00	0.50

Table S12. The input parameters of the elites after exploration in chemical space 3. Note in chemical space 3, the concentration of ascorbic acid was four times as that in chemical space 1.

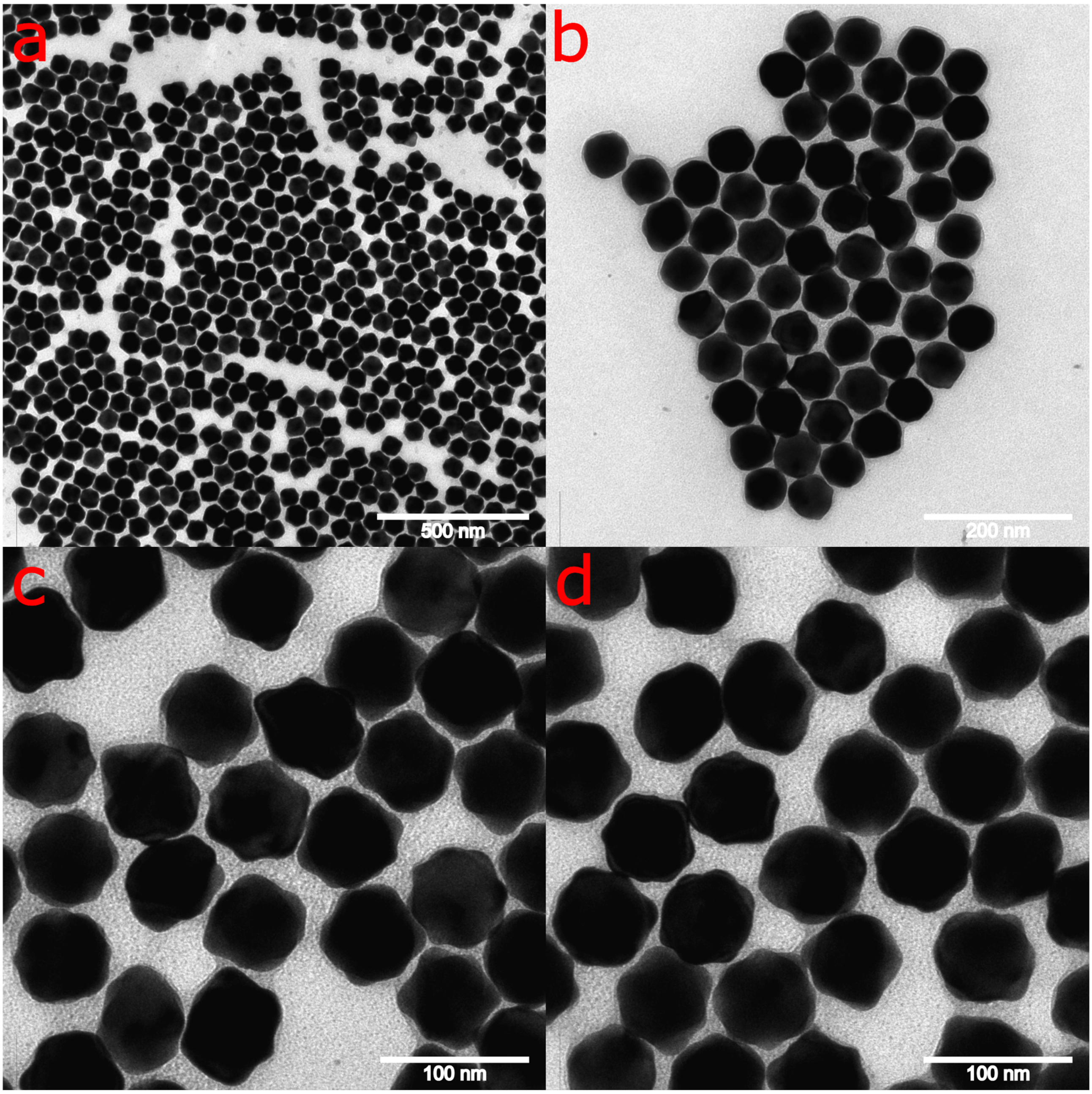

Figure S65. The TEM images of nanostars with small tips (Elite 2 in the single-peak system, also labelled as L3-1 in the manuscript). The scale bars are shown in the images.

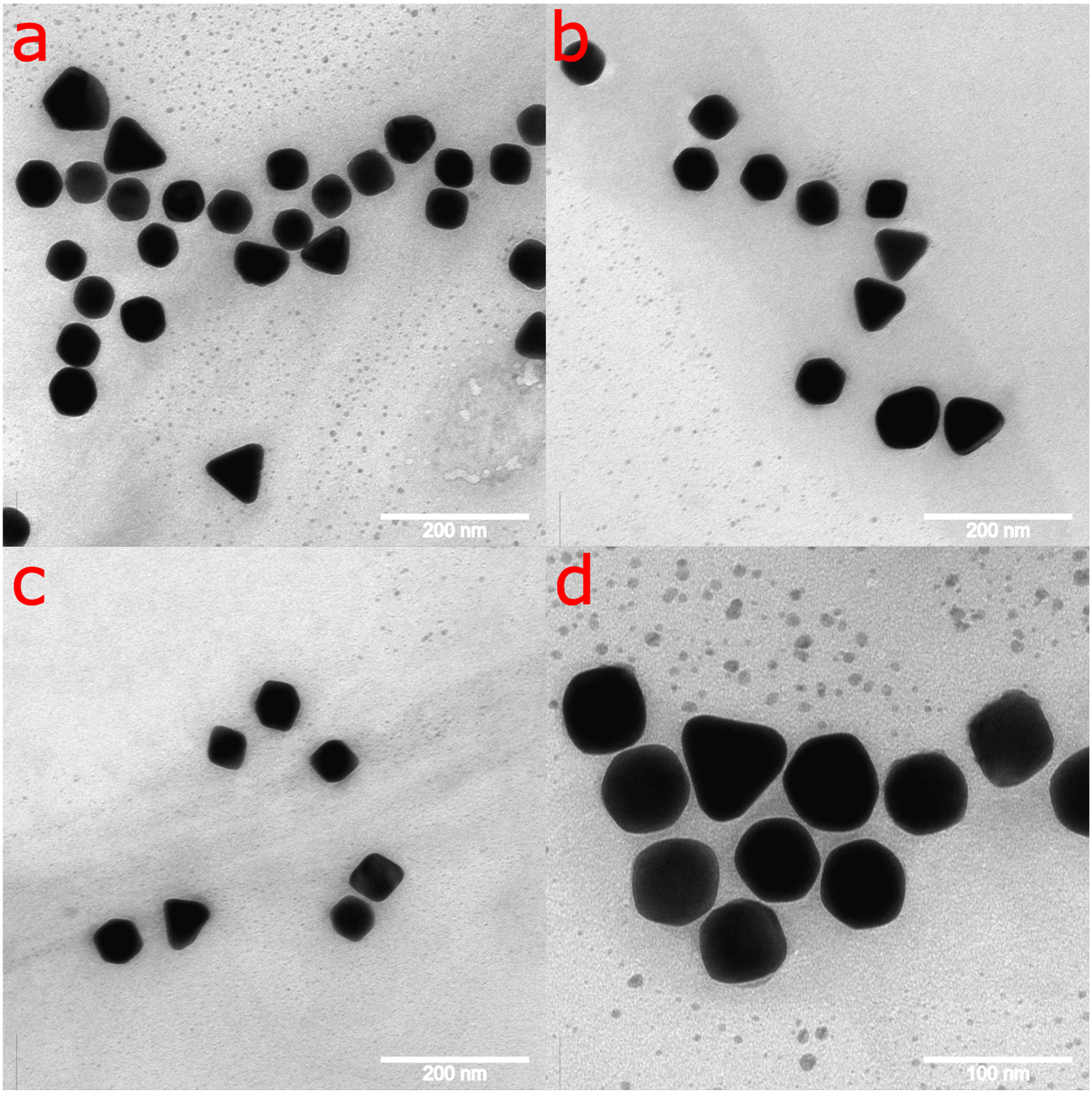

Figure S66. The TEM images of a mixture of spheres, octahedra and other by-products (Elite 3 in the single-peak system). Note the spherical nanoparticles already show the tendency to create octahedral features but are still smooth. A small portion of octahedral nanoparticles with distinct crystal faces are shown in (c). The scale bars are shown in the images.

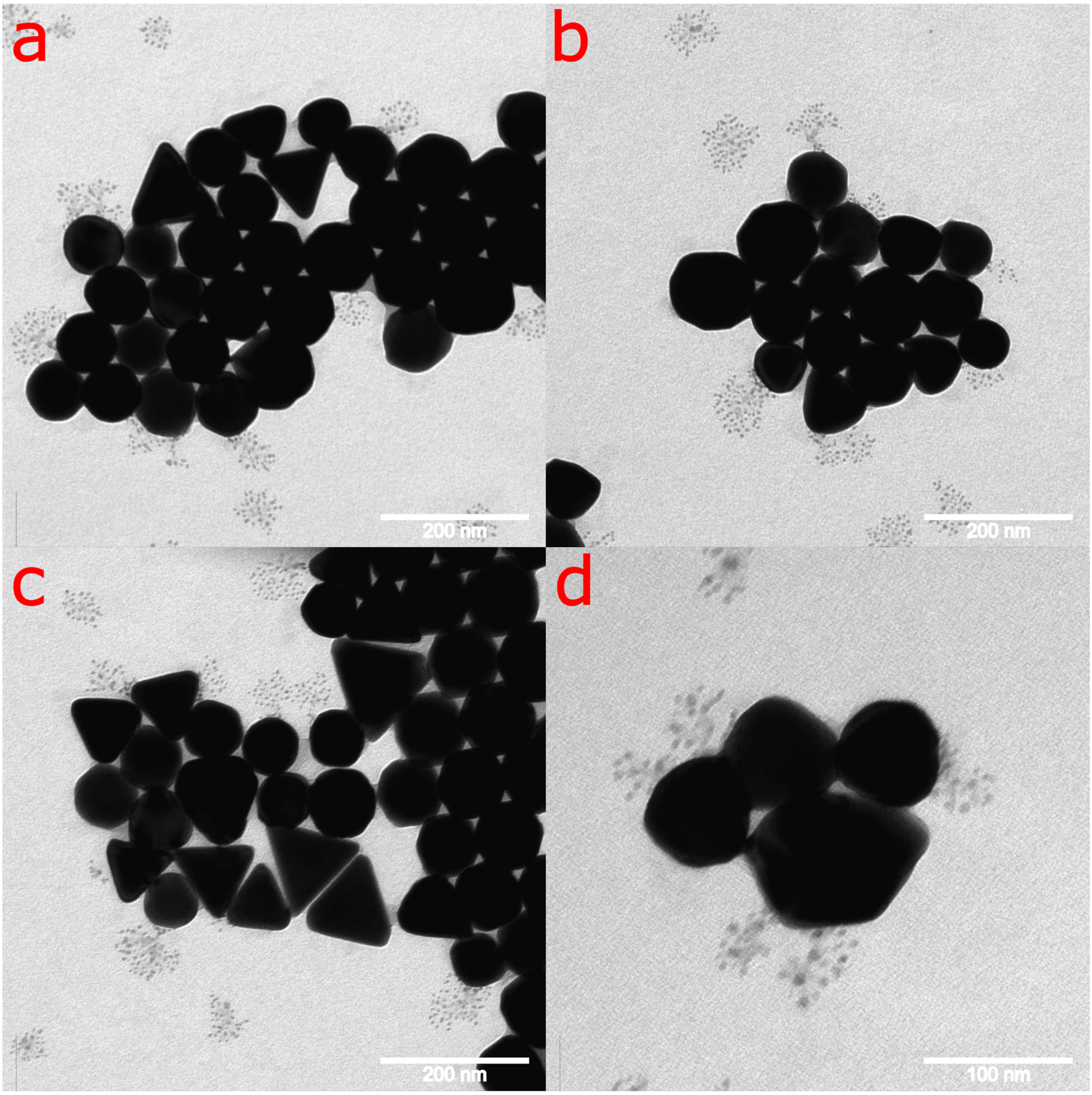

Figure S67. The TEM images of large polyhedral nanoparticles with prisms as by-products (Elite 4 in the single-peak system). The scale bars are shown in the images.

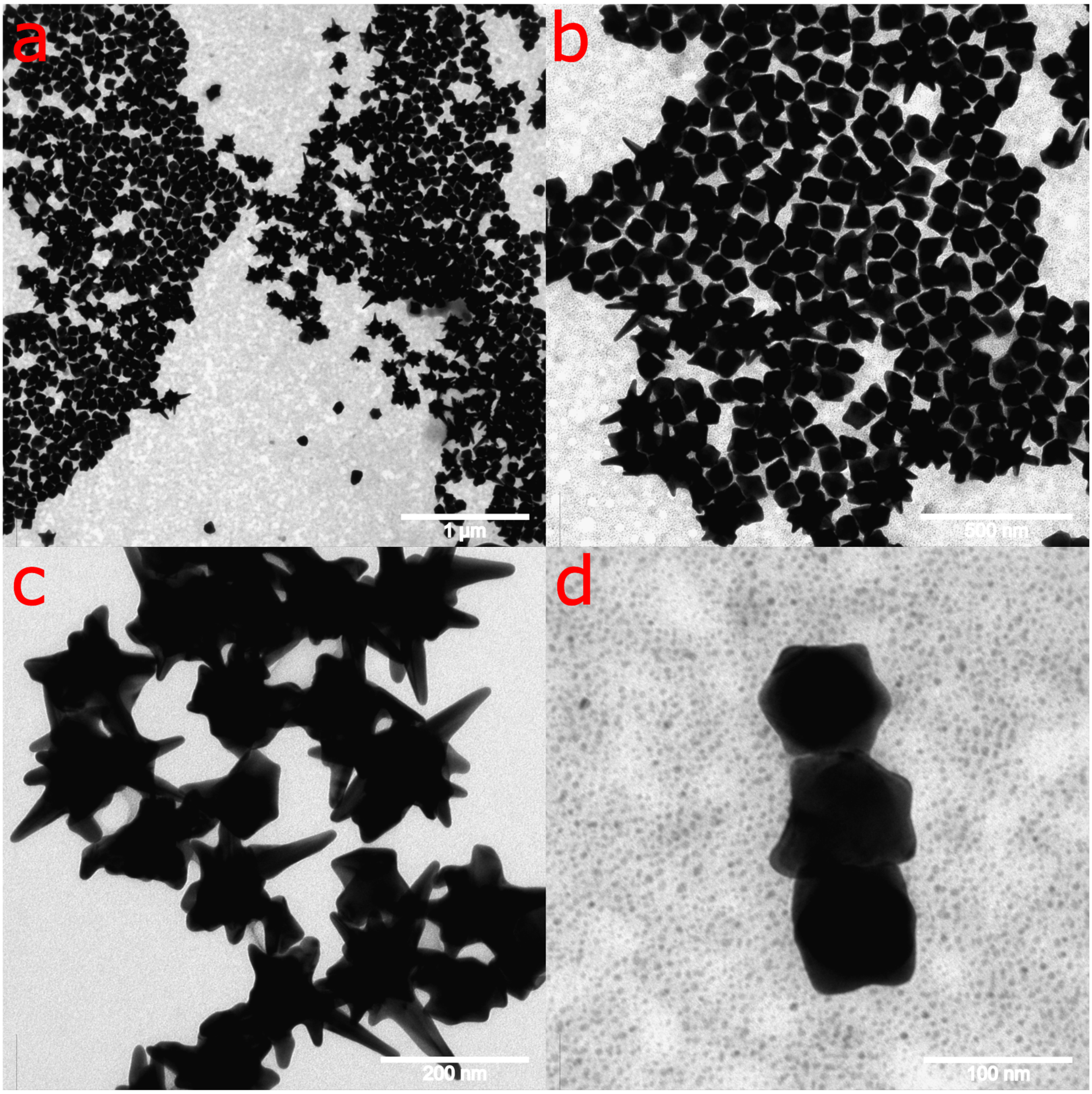

Figure S68. The TEM images of a mixture of nanostars and polyhedra (Elite 5 in the single-peak system). The scale bars are shown in the images.

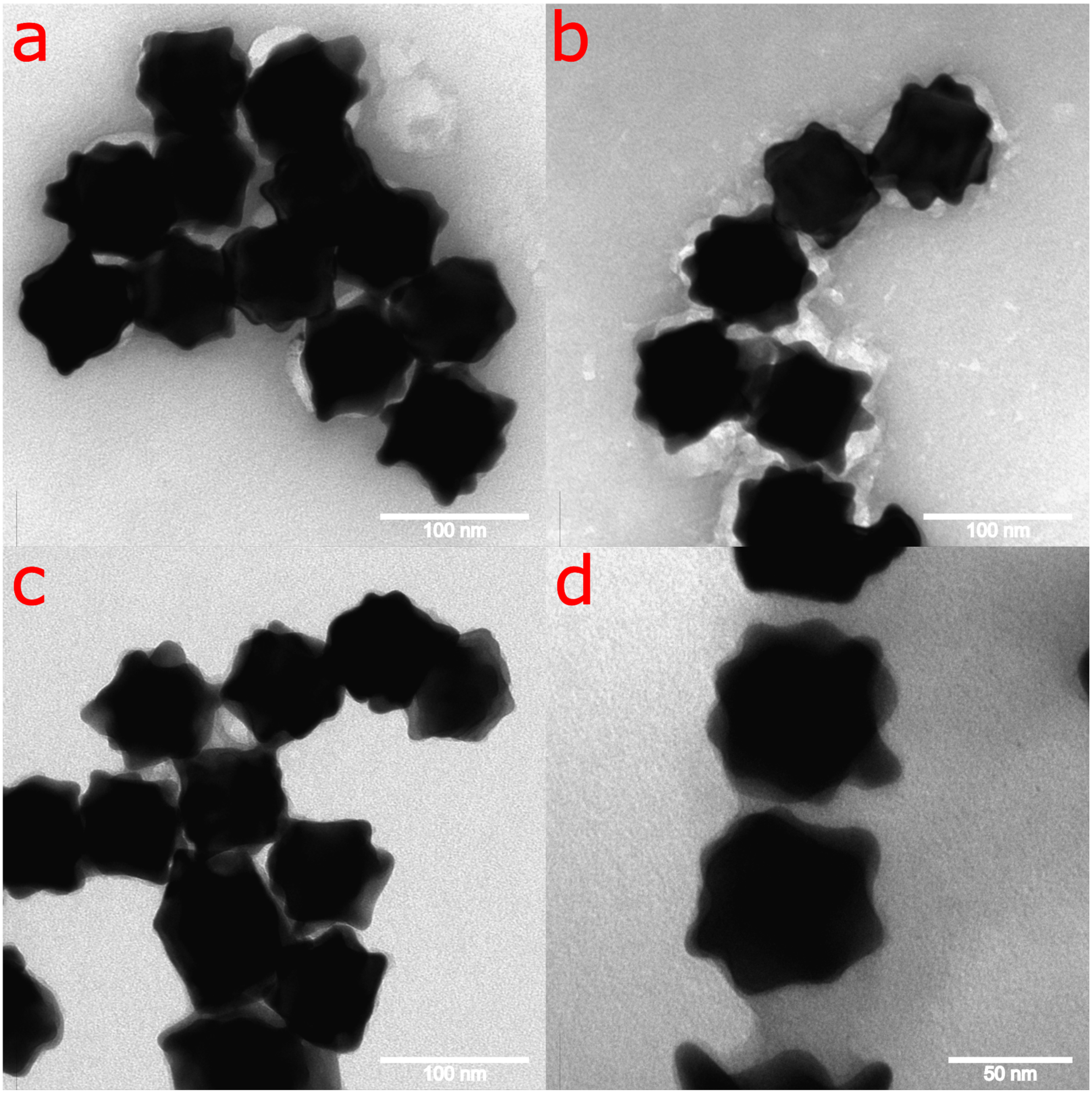

Figure S69. The TEM images of nanostars (Elite 6 in the single-peak system, also labelled as L3-2 in the manuscript). The scale bars are shown in the images.

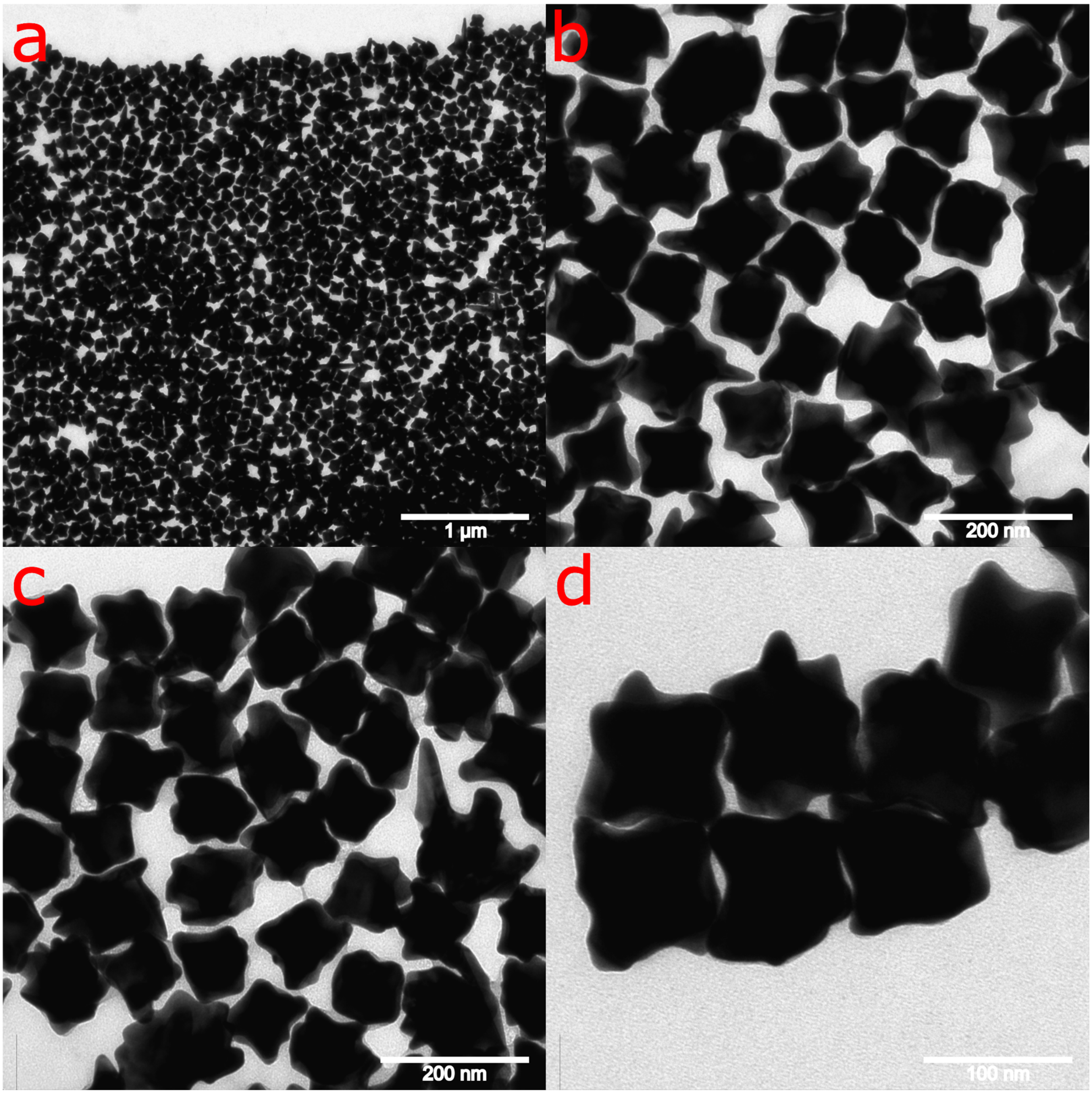

Figure S70. The TEM images of nanostars with medium size and tips (Elite 7 in the single-peak system, also labelled as L3-3 in the manuscript). The scale bars are shown in the images.

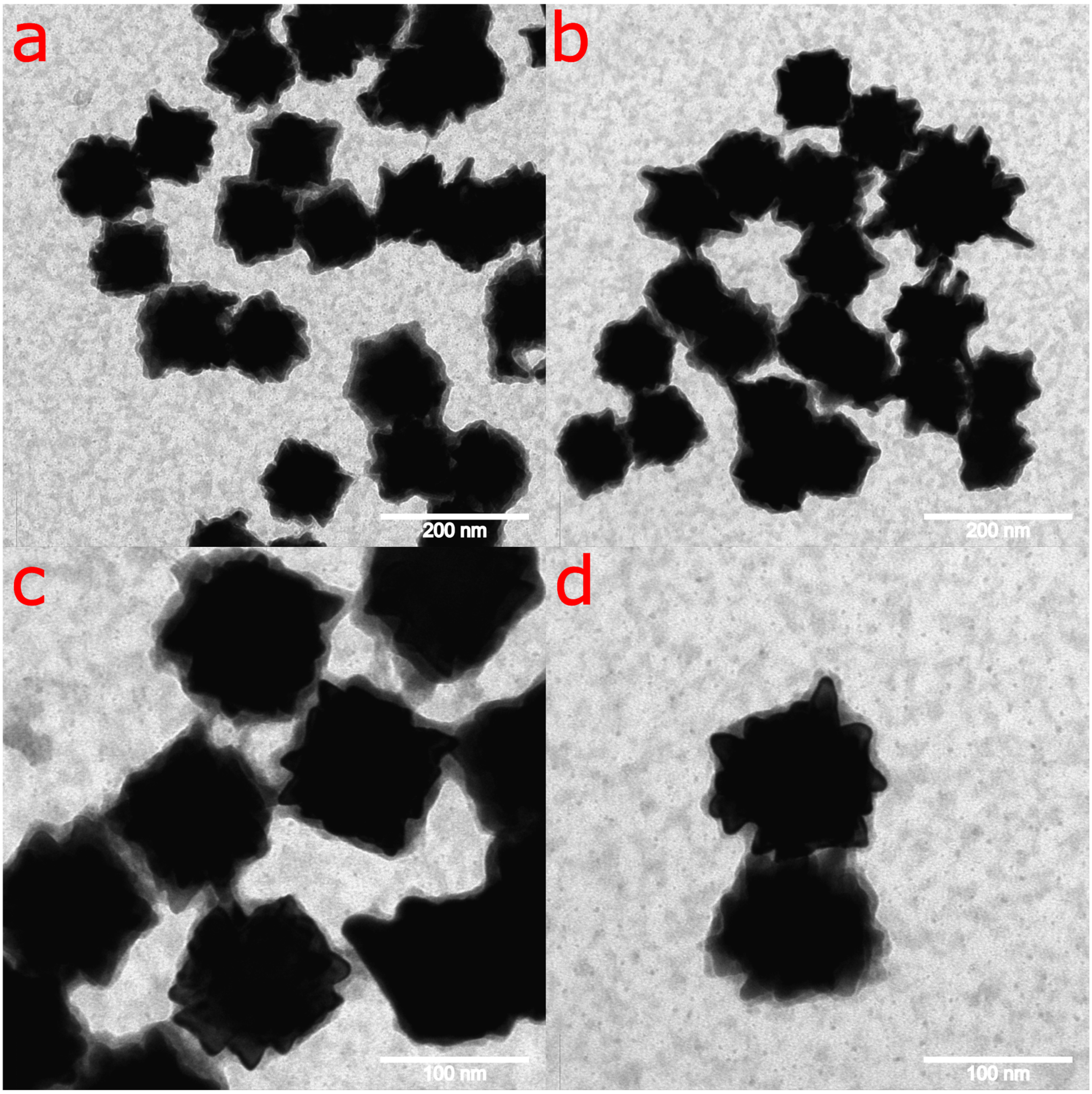

Figure S71. The TEM images of nanostars with rectangular cores (Elite 8 in the single-peak system, also labelled as L3-4 in the manuscript). The scale bars are shown in the images.

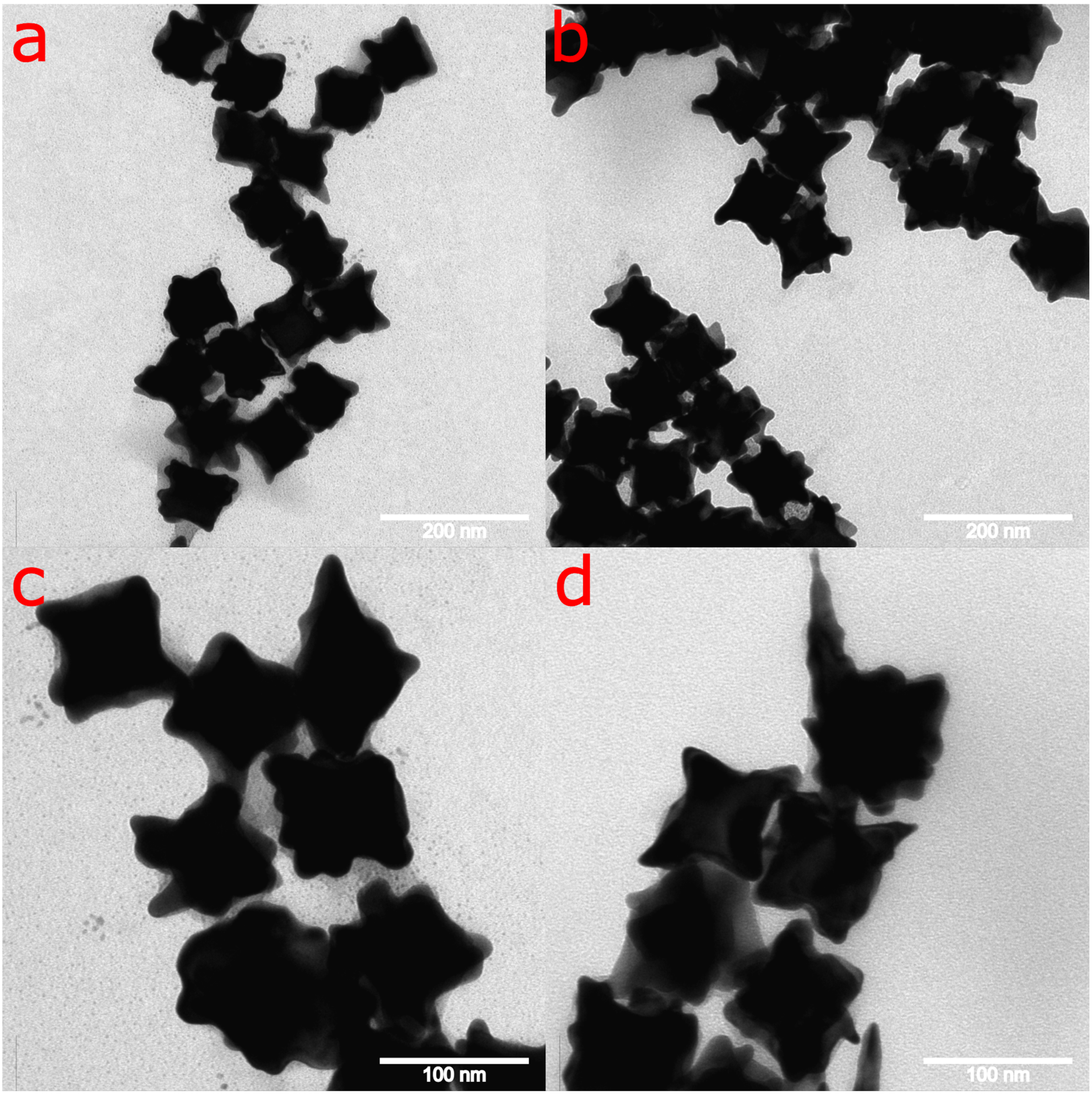

Figure S72. The TEM images of nanostars with large size and tips (Elite 9 in the single-peak system). The scale bars are shown in the images.

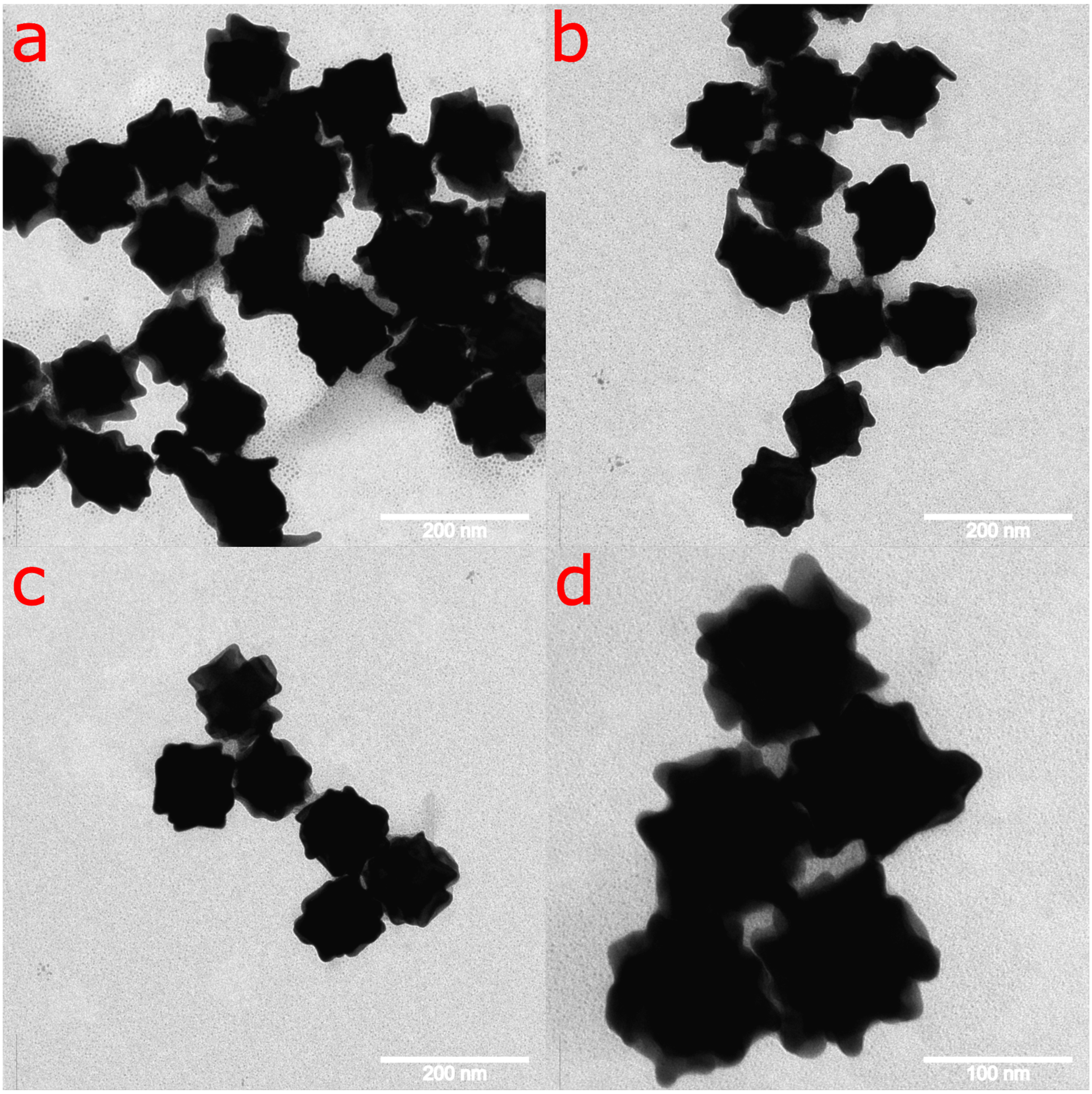

Figure S73. The TEM images of nanostars with large size and tips (Elite 10 in the single-peak system, also labelled as L3-5 in the manuscript). The scale bars are shown in the images.

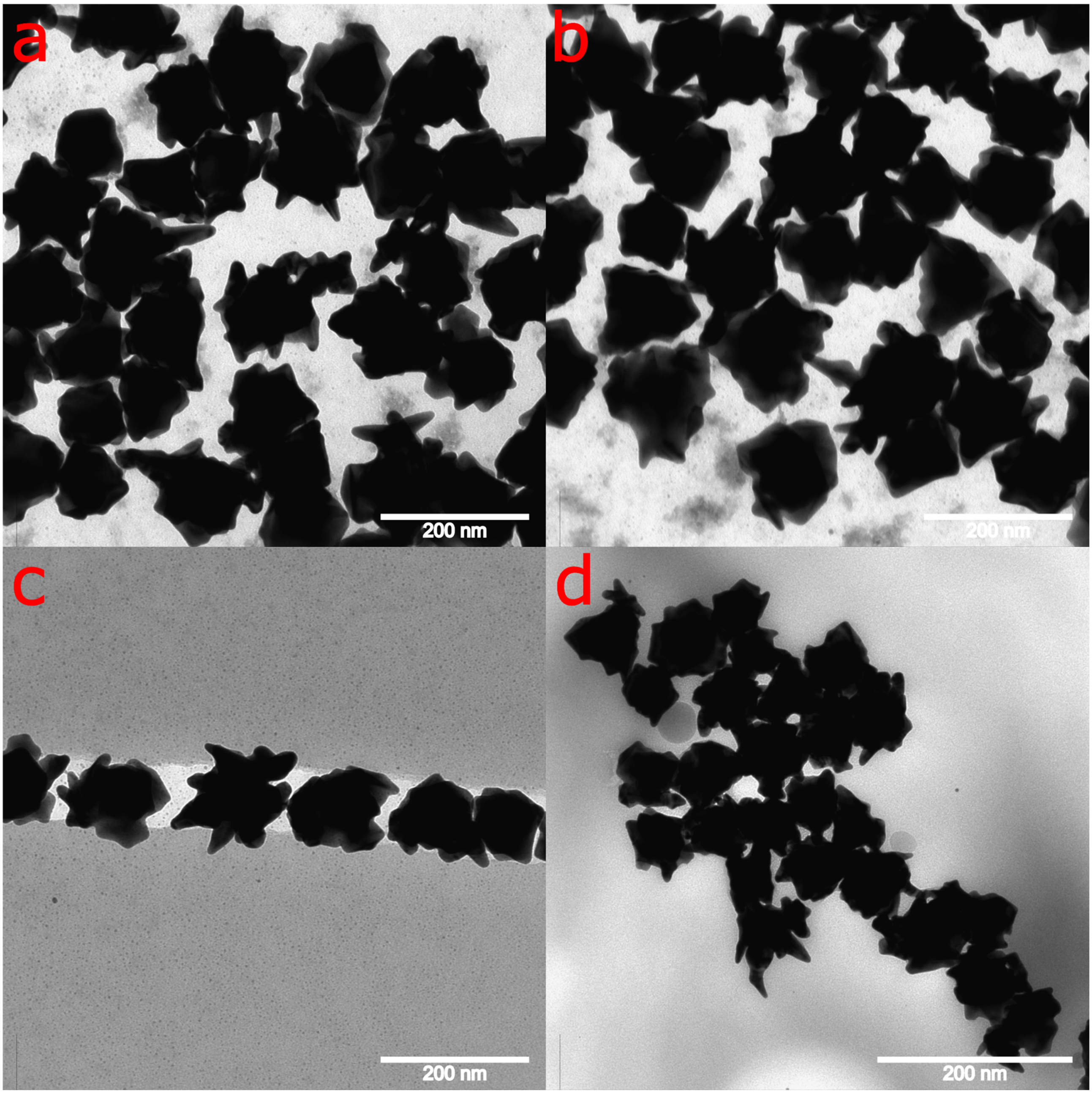

Figure S74. The TEM images of nanostars with large size and tips (Elite 11 in the single-peak system). The scale bars are shown in the images.

3.5. Estimating the possible experiments in exploration

The total number of possible experiments in exploring the three hierarchically-linked chemical space was estimated. We assume for a thorough grid search, one hundred different values for the volume of one chemical reagent are necessary. Regarding the pH variable, it is limited by the control decimal with a precision tolerance of ±0.2. Considering the pH range from 4 to 8 we used, the number of possible different values of the pH variable can be estimated as $\frac{8-4}{0.2}+1=21$. In calculating the possible combination number, the boundary conditions we put to constrain the overall volume should be considered as well.

1. In chemical space 1, the freedom of the variables is four. Four reagent volumes (CTAB, $HAuCl_4$, $AgNO_3$ and ascorbic acid) can change freely with a linear constraint so that their total volume should be no larger than 11.5 mL. Thus, the number of the total combinations is $(10^2)^4 \times \frac{1}{2} \times \frac{1}{3} \times \frac{1}{4}$, where $\frac{1}{2} \times \frac{1}{3} \times \frac{1}{4}$ estimates the influence from the constraint. In this calculation, the minimal volume interval is $\frac{11.5}{100-1} \approx 0.116$ mL.
2. In chemical space 2, the freedom of the variables is five. Four reagent volumes (CTAB, $HAuCl_4$, $AgNO_3$ and hydroquinone) and the pH variable can change. The linear constraint is that the total volume of CTAB and hydroquinone should be no larger than 7.0 mL. Additionally, the total volume of $HAuCl_4$ and $AgNO_3$ should be no larger than 5.0 mL. Thus, the number of the total combinations is $(10^2)^4 \times 21 \times \frac{1}{2} \times \frac{1}{2}$, where $\frac{1}{2} \times \frac{1}{2}$ estimates the influence from the two constraints. In this calculation, the minimal volume interval is $\frac{5}{100-1} \approx 0.05$ mL.
3. In chemical space 3, the freedom of the variables is five. Five reagent volumes (CTAB, $HAuCl_4$, $AgNO_3$, ascorbic acid and HCl) can change. The linear constraint is that the total volume of these five reagents should be no larger than 11.5 mL. Thus, the number of the total combinations is $(10^2)^5 \times \frac{1}{2} \times \frac{1}{3} \times \frac{1}{4} \times \frac{1}{5}$, where $\frac{1}{2} \times \frac{1}{3} \times \frac{1}{4} \times \frac{1}{5}$ estimates the influence from the constraint. In this calculation, the minimal volume interval is $\frac{11.5}{100-1} \approx 0.116$ mL.

Since the chemical spaces were hierarchically-linked and any solution (regardless their morphology and polydispersity) in principle can be used as the seed, the number of the total possible combinations in the exploration is $(10^2)^4 \times \frac{1}{2} \times \frac{1}{3} \times \frac{1}{4} \times (10^2)^4 \times 21 \times \frac{1}{2} \times \frac{1}{2} \times (10^2)^5 \times \frac{1}{2} \times \frac{1}{3} \times \frac{1}{4} \times \frac{1}{5} \approx 1.8 \times 10^{23}$.

3.6. Mutation, crossover, and random sampling in the exploration algorithm

The MAP-Elites algorithm serves two purposes in exploration:

1. Facilitated the diversity of the samples and find new elites (classes) that did not exist before.
2. Improve the performance of the existing elites.

During our experimental implementation, they are realised by three operations including crossover, mutation and random sampling. Crossover and mutation are evolutionary operations mimicking the natural evolution process. Compared to random sampling, these operations constrained the sampling points either near the current elites or as a combination of the input variables from the elites, while random sampling can search the space uniformly/without preference.

When the desired classes are distributed uniformly in the space, all three operations help to find the new elites. When crossover/mutation trapped the exploration due to their sampling constraint, random sampling help to add new input features. This is demonstrated by the observations of obtaining new classes through random sampling during the various exploration processes.

However, if the desired classes are within small subregions in the input space, relying on random sampling is inefficient. In this case, crossover and mutation, which maintain certain input features from the existing elites, help to target these subregions efficiently. It is demonstrated by the exploration of the single-peak system in chemical space 2, where the new classes (Elite 6-8) emerged at a very low concentration of CTAB. This feature is passed from the parent set via evolutionary operations and is not easily available by random sampling.

To improve the performance of existing elites, the evolutionary operations played a dominant role over random sampling. The ratios of the times where a better elite from evolutionary operations or random sampling was found in the open-ended exploration in the three chemical spaces are listed in **Table S13**.

**Space**	**Chemical Space 1**	**Chemical Space 2 (Multiple-peak)**	**Chemical Space 2 (Single-peak)**	**Chemical Space 3**
**Ratio**	30:2	35:5	8:0	15:0

Table S13. The ratios of the times where the elite's performance is improved from evolutionary operations or random sampling during the open-ended exploration of three chemical spaces. Note the exploration processes for multiple-peak and single-peak systems in chemical space 2 are shown separately, and the number of times to create a new elite is not counted here.

Considering the frequency of evolutionary operations and the random sampling in designing experiments (which is 20:3), the evolutionary operations are more likely to improve the performance of the elites. More importantly, it was at the early stage of the exploration that random sampling worked. When the performance of the elites was moderately good, improving the performance further

with random sampling was unlikely to happen, which was consistent with the observation in benchmarking the algorithm in **Section 2.5**.

3.7. Monodispersity analysis during exploration

In the previous sections, TEM images for the discovered nanostructures showed their optimal monodispersity. Here we quantitively analysed the monodispersity of the discovered nanospheres and nanorods to give a performance metric of the results. The diameter of the nanospheres, and the width, length, and aspect ratio of the nanorods were analysed using their TEM images with at least 100 nanoparticles (see precise numbers below). The polydispersity index (PDI) of the measured geometric parameter was also calculated via Eq.(33):

$$PDI(x) = \left(\frac{\sigma_x}{E[x]}\right)^2 \tag{33}$$

where x is the geometric parameter, $E[x]$ is its average value from the measurement and σ_x is the standard deviation.

3.7.1. Chemical Space 1

Nanospheres (L1-1):

The average size of the nanospheres (L1-1) was estimated by measuring the diameters of 500 nanospheres from **Figure S38b-d**. The mean and standard deviation of the diameters are 14.77 nm and 1.36 nm, respectively. The PDI from the measured diameters was calculated as 0.008. The histogram of the diameter distribution of the nanospheres is shown in **Figure S75**.

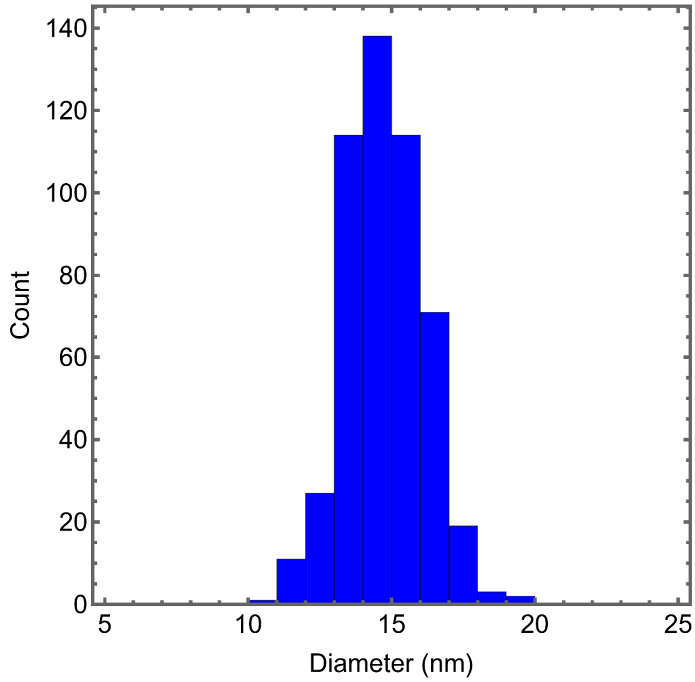

Figure S75. Histograms of the diameter distribution of the nanosphere sample L1-1. 500 nanospheres from **Figure S38b-d** were measured.

Nanorods (L1-2 to L1-6):

Three geometric parameters including the width, length and aspect ratio of the nanorods (L1-2 to L1-6) were measured, with the corresponding PDI summarized in **Table S14**. The histograms of the distributions of these three parameters corresponding to different samples (L1-2 to L1-6) are available in **Figure S76-Figure S80**. L1-2 and L1-3 showed very similar distributions for every geometric parameter, which is consistent with their similar UV-Vis spectra as shown in Figure 4B in the manuscript. The increased aspect ratio from L1-2/L1-3 to L1-6 is also consistent with the red-shifted longitudinal peak as observed in the corresponding UV-Vis spectra.

Sample	Width (nm)	PDI (Width)	Length (nm)	PDI (Length)	Aspect ratio	PDI (Aspect ratio)
**L1-2**	12.68 ± 1.20	0.009	28.37 ± 2.72	0.009	2.26 ± 0.32	0.020
**L1-3**	13.16 ± 1.60	0.015	27.99 ± 3.34	0.014	2.15 ± 0.33	0.024
**L1-4**	11.67 ± 1.67	0.020	35.45 ± 3.25	0.008	3.08 ± 0.37	0.014
**L1-5**	11.18 ± 1.69	0.023	39.13 ± 4.43	0.013	3.57 ± 0.61	0.029
**L1-6**	7.59 ± 0.73	0.009	30.45 ± 3.34	0.012	4.03 ± 0.48	0.014

Table S14. The geometric parameters and their corresponding PDI of nanorod samples L1-2, L1-3, L1-4, L1-5 and L1-6. The width, length and aspect ratio are shown in the format of mean ± standard deviation. The corresponding PDI for every parameter was calculated via Eq.(33).

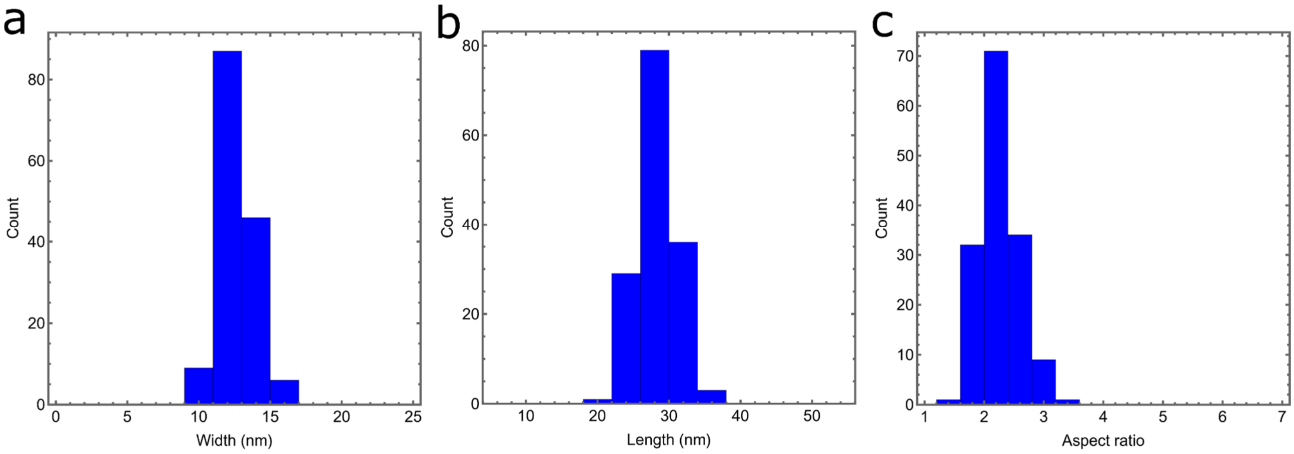

Figure S76. Histograms of the geometric parameters of L1-2. The distributions of width, length and aspect ratio are shown in (a), (b) and (c) respectively. 148 nanorods from **Figure S39c-d** were measured.

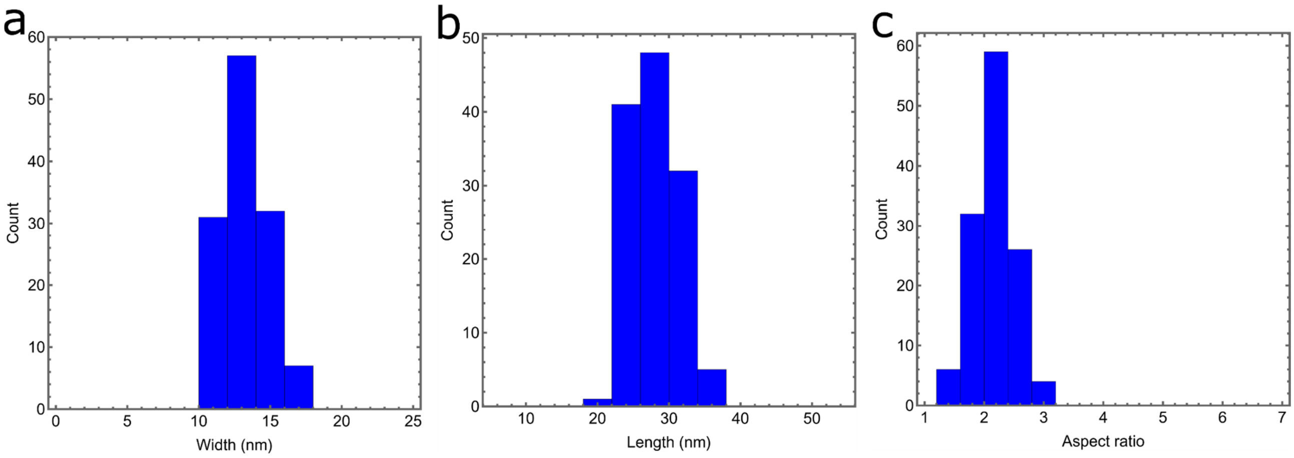

Figure S77. Histograms of the geometric parameters of L1-3. The distributions of width, length and aspect ratio are shown in (a), (b) and (c) respectively. 127 nanorods from **Figure S40c-d** were measured.

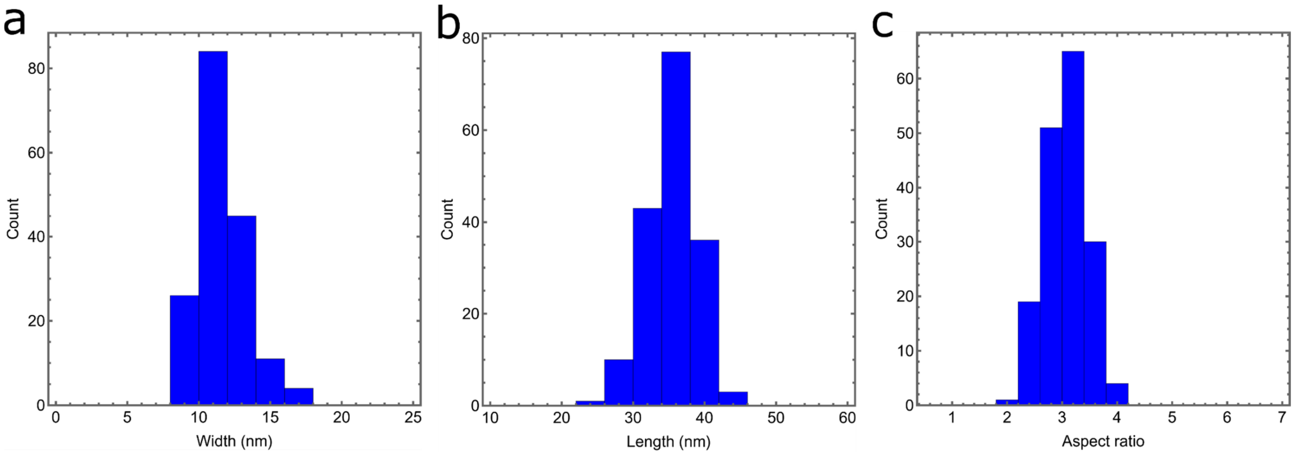

Figure S78. Histograms of the geometric parameters of L1-4. The distributions of width, length and aspect ratio are shown in (a), (b) and (c) respectively. 170 nanorods from **Figure S41b-c** were measured.

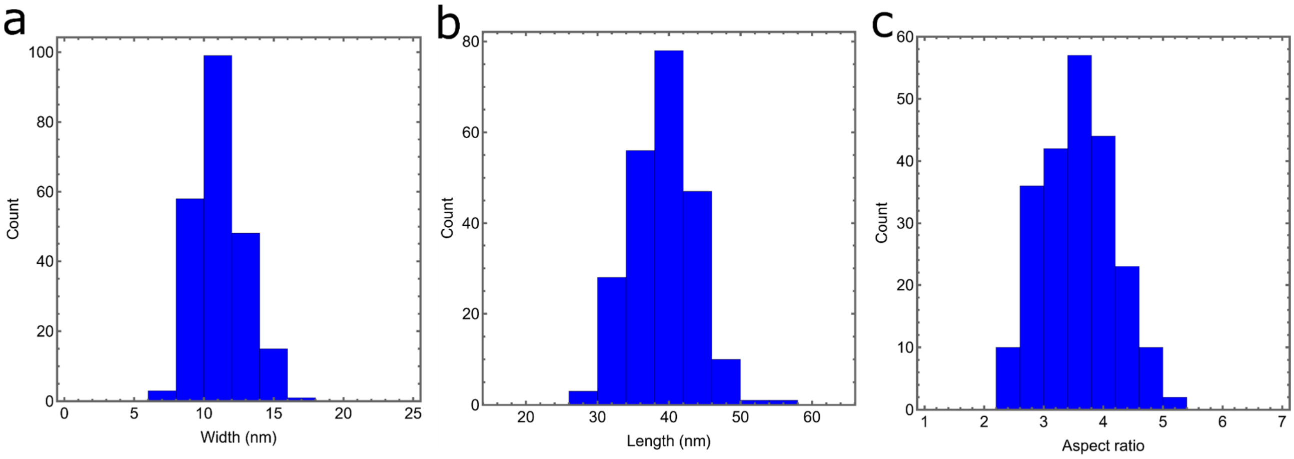

Figure S79. Histograms of the geometric parameters of L1-5. The distributions of width, length and aspect ratio are shown in (a), (b) and (c) respectively. 224 nanorods from **Figure S42b-d** were measured.

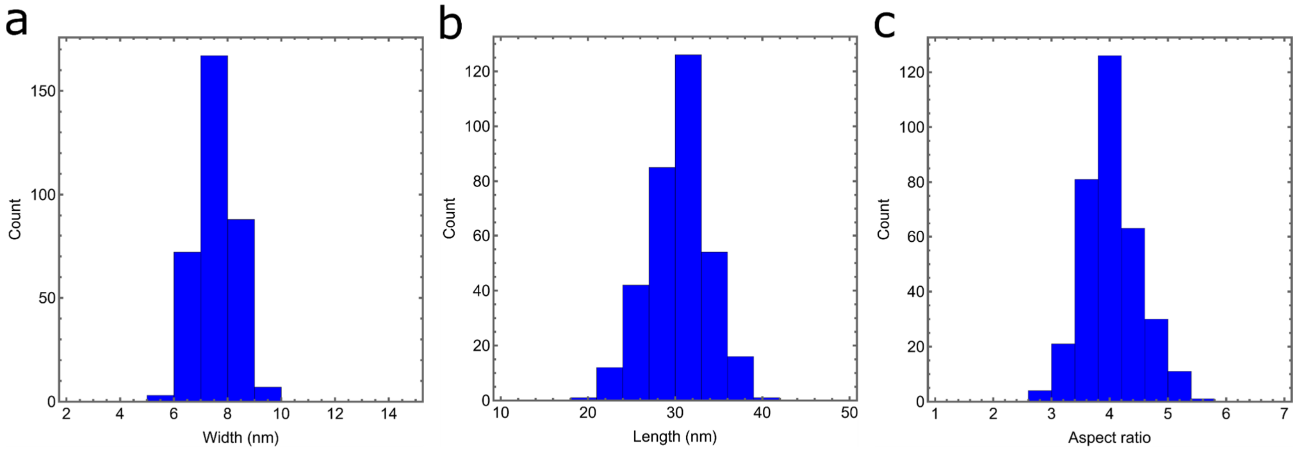

Figure S80. Histograms of the geometric parameters of L1-6. The distributions of width, length and aspect ratio are shown in (a), (b) and (c) respectively. 337 nanorods from **Figure S43b-d** were measured.

3.7.2. Chemical Space 2

Nanospheres (L2-11-2 and L2-12-2):

The average sizes for both nanosphere samples (L1-11-2 and L1-12-2) were estimated. The means and standard deviations of the diameters and the corresponding PDI are summarized in **Table S15**. The histograms of the distributions of the diameters of L1-11-2 and L1-12-2 are shown in **Figure S81a** and **Figure S81b**, respectively.

Sample	Diameter (nm)	PDI (Diameter)
**L2-11-2**	42.48 ± 2.31	0.003
**L2-12-2**	45.02 ± 2.18	0.002

Table S15. The measured diameters and the corresponding PDI of L2-11-2 to L2-12-2. The diameters are shown in the format of mean ± standard deviation.

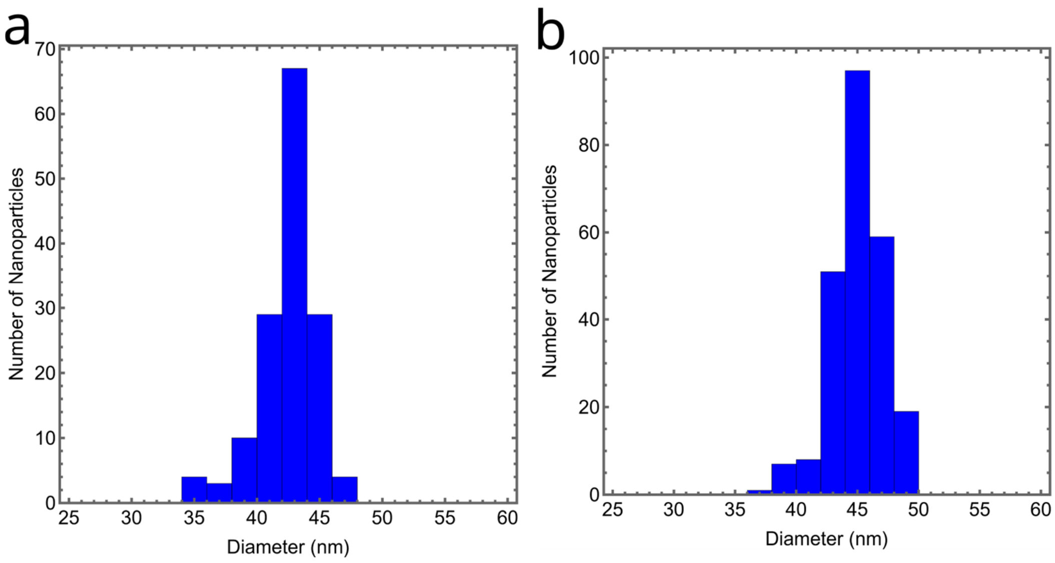

Figure S81. Histograms of the diameters of nanospheres. (a) and (b) correspond to L2-11-2 and L2-12-2. 146 and 242 nanospheres from Figure S61 and Figure S62 were measured, respectively.

Nanorods (L2-1 to L2-10, and L2-13):

Three geometric parameters including the width, length, and aspect ratio of the nanorods (L2-1 to L2-10, as well as L2-13) were measured and summarized in **Table S16**. The histograms of the distributions of these three parameters corresponding to different samples (L2-1 to L2-10, together with L2-13) are available in **Figure S82**-**Figure S92**. Au nanorods with spherical caps showed a red-shifted longitudinal peak with the increased aspect ratio (L2-13, L2-10, L2-1, L2-2). A similar phenomenon was observed for Au nanorods with rectangular caps (L2-3, L2-5, L2-7 and L2-9). Although L2-1 and L2-3 have similar aspect ratios, the longitudinal peak of Au nanorods with rectangular caps (L2-3) is more red-shifted compared to the ones with spherical caps (L2-1). This is consistent with the previous studies about the effects of the end-cap shape of Au nanorods on the extinction spectrum (*57*).

**Sample**	**Width (nm)**	**PDI (Width)**	**Length (nm)**	**PDI (Length)**	**Aspect ratio**	**PDI (Aspect ratio)**
**L2-1**	19.04 ± 1.72	0.008	45.86 ± 5.18	0.013	2.43 ± 0.36	0.022
**L2-2**	18.08 ± 1.27	0.005	51.00 ± 4.45	0.008	2.84 ± 0.36	0.016
**L2-3**	29.73 ± 1.72	0.003	70.26 ± 6.37	0.008	2.37 ± 0.25	0.011
**L2-4**	28.21 ± 3.03	0.012	54.69 ± 4.92	0.008	1.95 ± 0.22	0.013
**L2-5**	19.98 ± 1.58	0.006	61.57 ± 5.67	0.008	3.11 ± 0.41	0.017
**L2-6**	15.66 ± 1.81	0.013	46.63 ± 4.48	0.009	3.01 ± 0.42	0.019
**L2-7**	22.33 ± 2.09	0.009	75.13 ± 6.92	0.008	3.40 ± 0.48	0.020
**L2-8**	16.52 ± 1.48	0.008	48.52 ± 5.20	0.011	2.95 ± 0.37	0.016
**L2-9**	16.65 ± 1.18	0.005	75.64 ± 7.40	0.010	4.57 ± 0.61	0.018
**L2-10**	26.36 ± 1.53	0.003	51.95 ± 4.60	0.008	1.98 ± 0.21	0.011
**L2-13**	28.27 ± 1.71	0.004	48.67 ± 4.94	0.010	1.73 ± 0.20	0.013

Table S16. The geometric parameters and their corresponding PDI of nanorod samples L2-1 to L2-10, together with L2-13. The width, length and aspect ratio are shown in the format of mean ± standard deviation. The corresponding PDI for every parameter was calculated via Eq.(33).

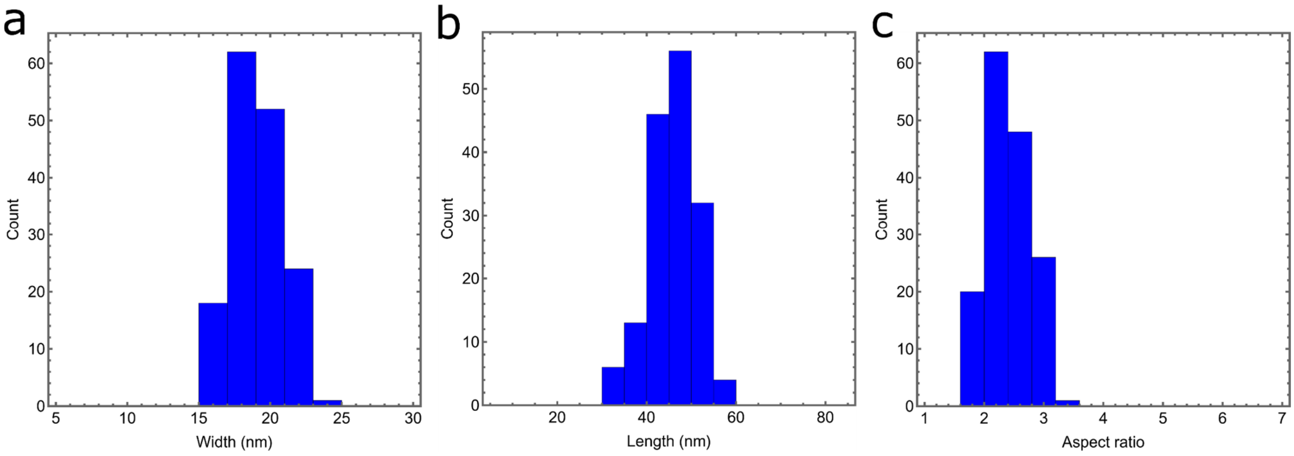

Figure S82. Histograms of the geometric parameters of L2-1. The distributions of width, length and aspect ratio are shown in (a), (b) and (c) respectively. 157 nanorods from **Figure S48b-c** were measured.

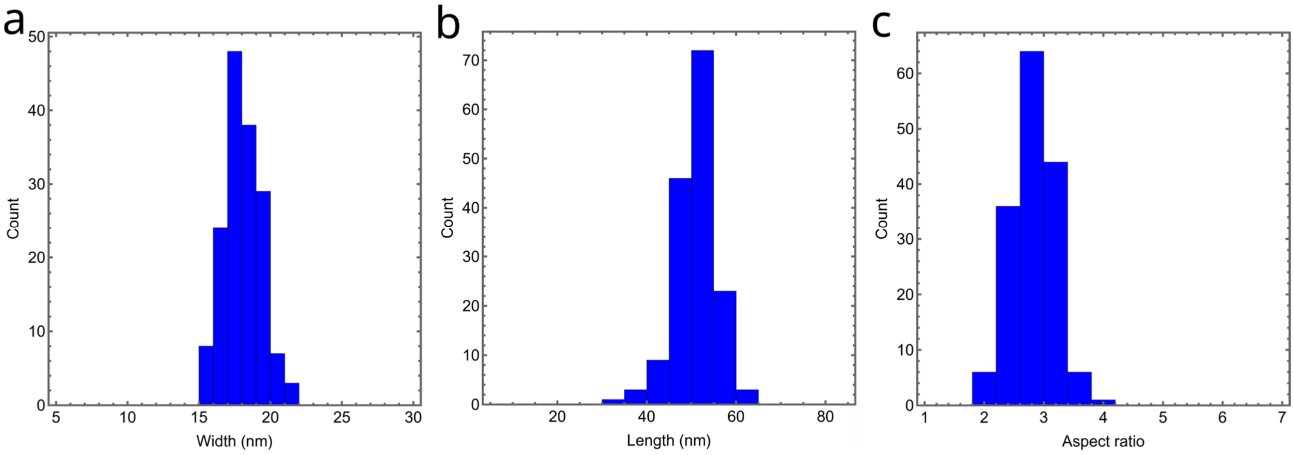

Figure S83. Histograms of the geometric parameters of L2-2. The distributions of width, length and aspect ratio are shown in (a), (b) and (c) respectively. 157 nanorods from **Figure S49b-c** were measured.

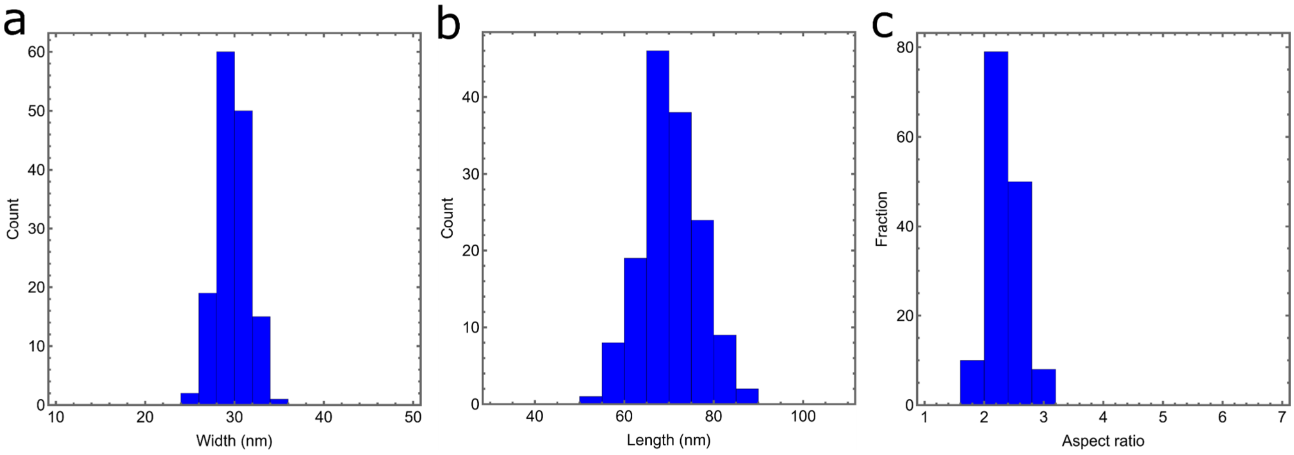

Figure S84. Histograms of the geometric parameters of L2-3. The distributions of width, length and aspect ratio are shown in (a), (b) and (c) respectively. 147 nanorods from **Figure S50a-c** were measured.

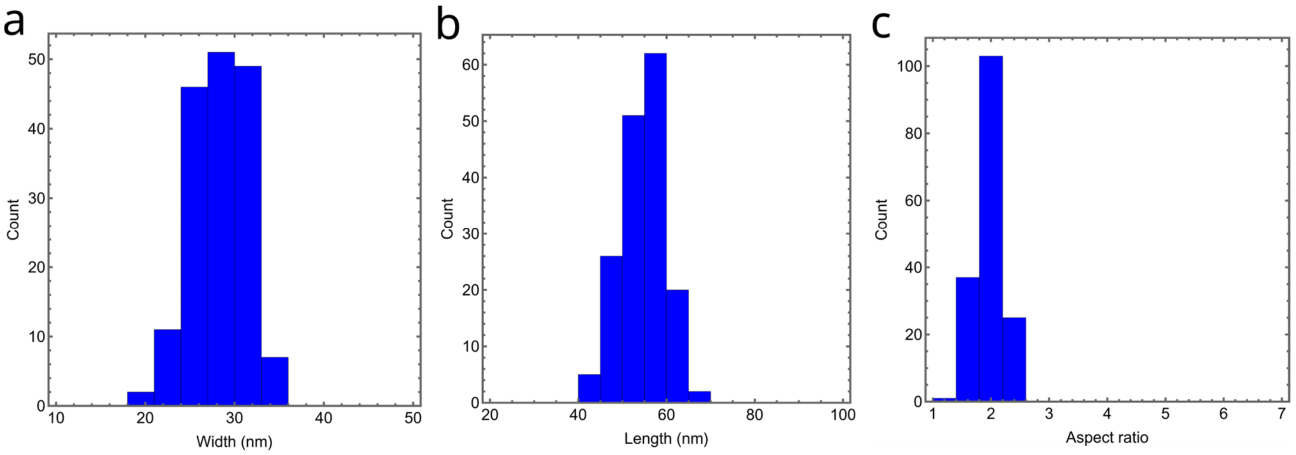

Figure S85. Histograms of the geometric parameters of L2-4. The distributions of width, length and aspect ratio are shown in (a), (b) and (c) respectively. 166 nanorods from **Figure S51a** were measured.

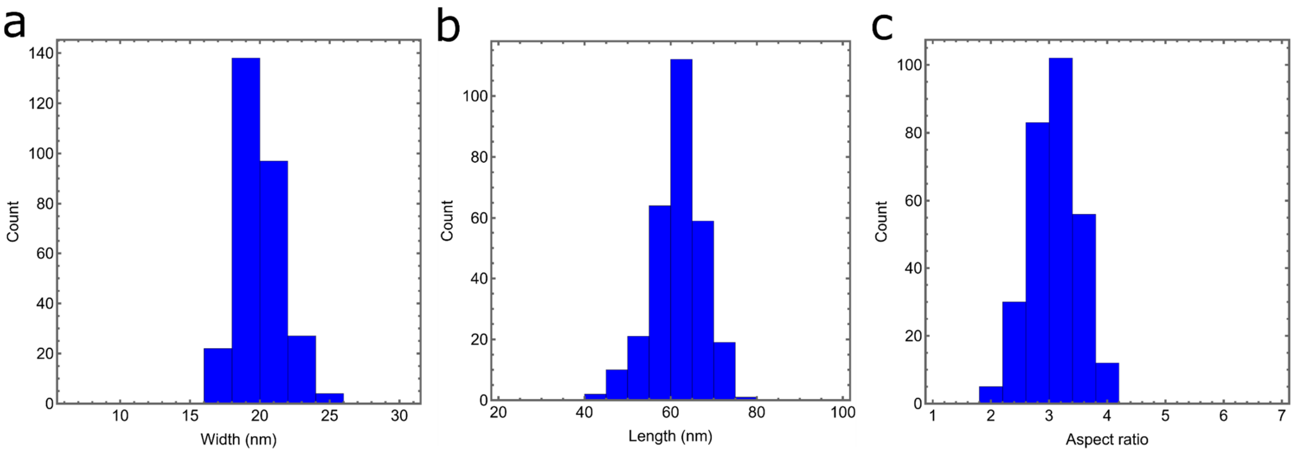

Figure S86. Histograms of the geometric parameters of L2-5. The distributions of width, length and aspect ratio are shown in (a), (b) and (c) respectively. 288 nanorods from **Figure S52a-b** were measured.

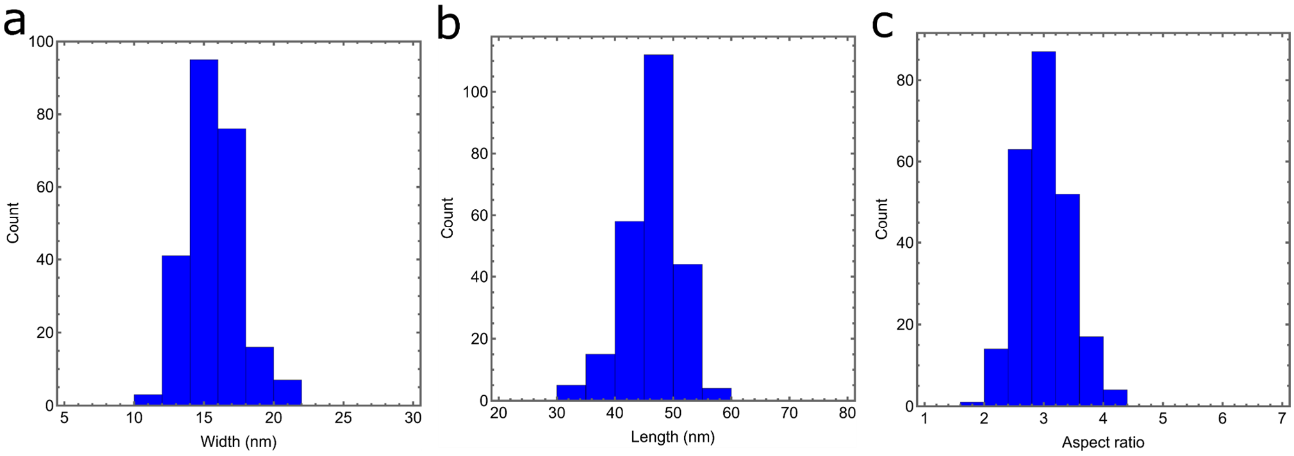

Figure S87. Histograms of the geometric parameters of L2-6. The distributions of width, length and aspect ratio are shown in (a), (b) and (c) respectively. 238 nanorods from **Figure S53a-c** were measured.

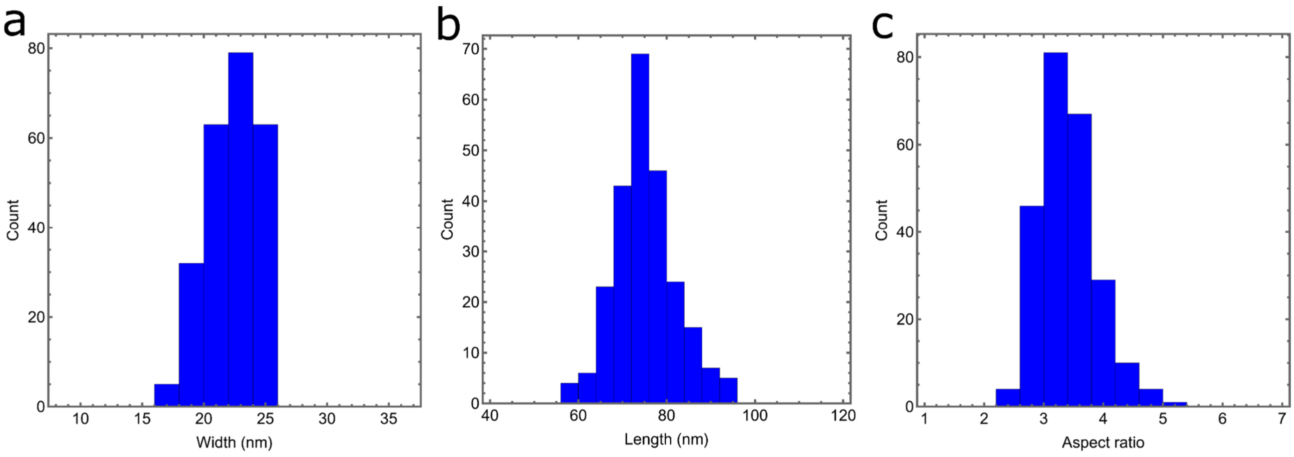

Figure S88. Histograms of the geometric parameters of L2-7. The distributions of width, length and aspect ratio are shown in (a), (b) and (c) respectively. 242 nanorods from **Figure S54a-c** were measured.

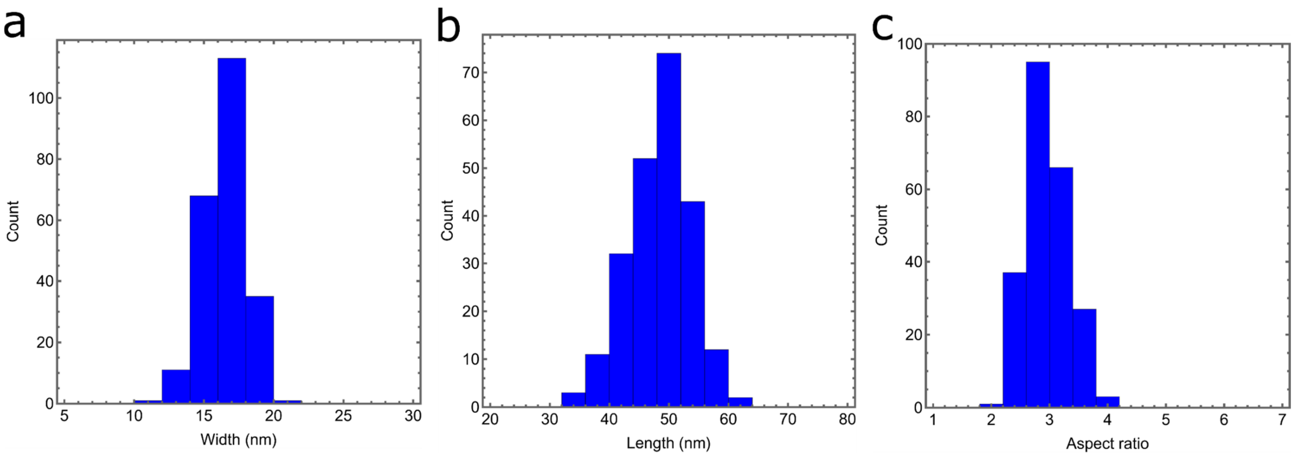

Figure S89. Histograms of the geometric parameters of L2-8. The distributions of width, length and aspect ratio are shown in (a), (b) and (c) respectively. 229 nanorods from **Figure S55a-b** were measured.

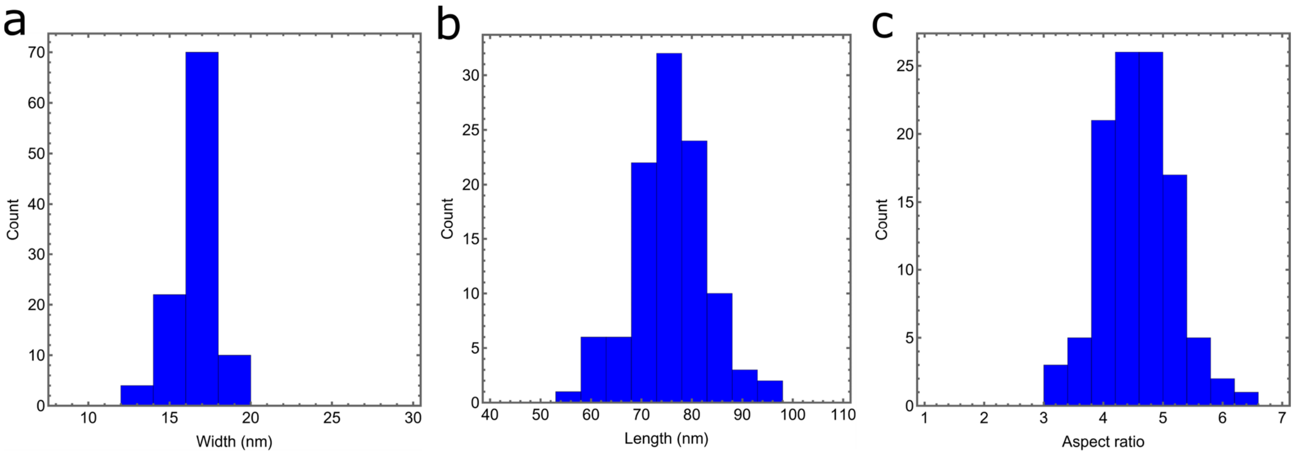

Figure S90. Histograms of the geometric parameters of L2-9. The distributions of width, length and aspect ratio are shown in (a), (b) and (c) respectively. 106 nanorods from **Figure S56a-c** were measured.

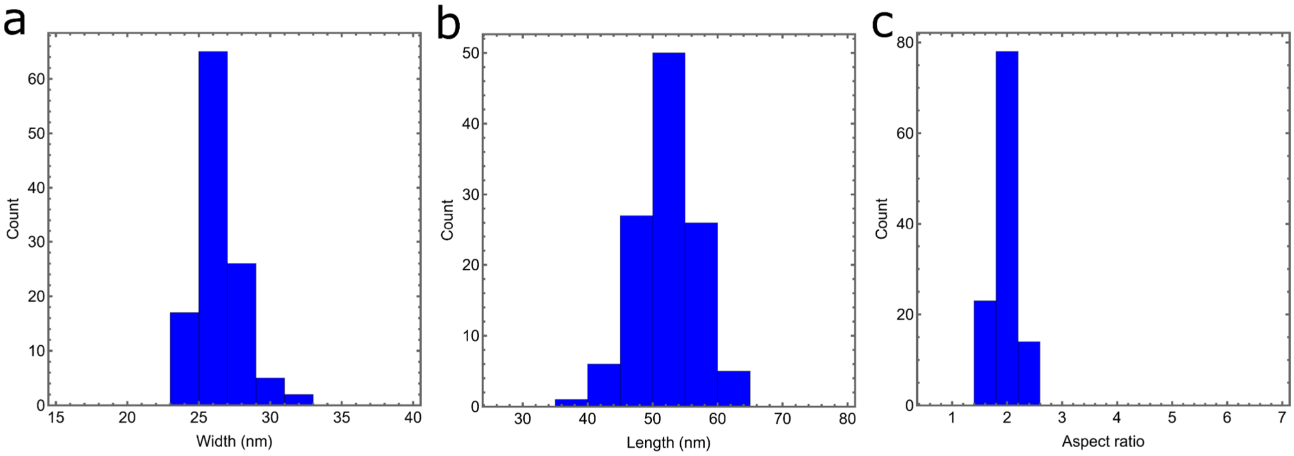

Figure S91. Histograms of the geometric parameters of L2-10. The distributions of width, length and aspect ratio are shown in (a), (b) and (c) respectively. 115 nanorods from **Figure S57b-c** were measured.

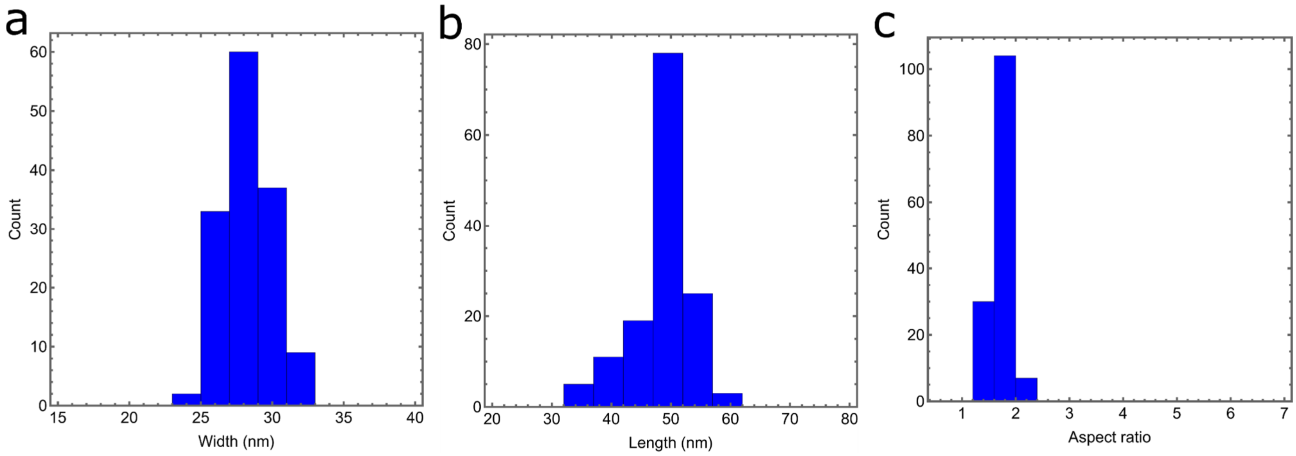

Figure S92. Histograms of the geometric parameters of L2-13. The distributions of width, length and aspect ratio are shown in (a), (b) and (c) respectively. 141 nanorods from **Figure S60c-d** were measured.

3.8. Time cost analysis during exploration

To give a detailed insight into the time cost during the autonomous exploration, we recorded the time cost of individual operations (including solution preparation, waiting for complete growth and UV-Vis characterisation and cleaning) during the exploration of chemical space 2 and 3 (30 steps, 720 experiments in total). In both cases, the waiting time for the growth of a batch of samples was set as 60 minutes. The UV-Vis analysis and cleaning took 98 minutes through the whole 30 steps, regardless of the required synthetic conditions.

The time cost is higher in exploring the chemical space 2 due to the waiting time for a stable pH read-out and the process to control the pH. The time cost for liquid dispensing/pH control varied in every step, depending on the synthetic conditions generated for that step (139 ± 7 minutes, see **Figure S93**). Thus, the total time cost for a single step including 24 experiments took 297 ± 7 minutes (ca. five hours). During the exploration of chemical space 3, only liquid dispensing was required, and the preparation time was decreased to 63 ± 7 minutes (see **Figure S94**), thus giving a total of 221 ± 7 minutes (ca. three and a half hours) for one complete step.

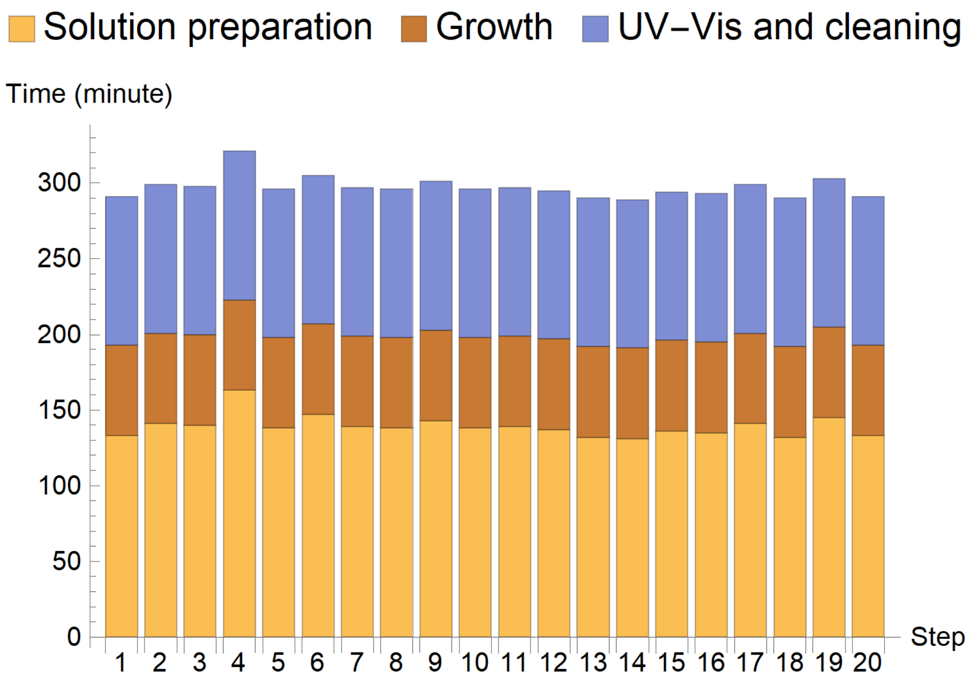

Figure S93. The time cost distribution during exploring chemical space 2 with pH control. The time cost for solution preparation (liquid dispensing and pH control), waiting for growth and UV-Vis characterisation and cleaning are shown in different colours.

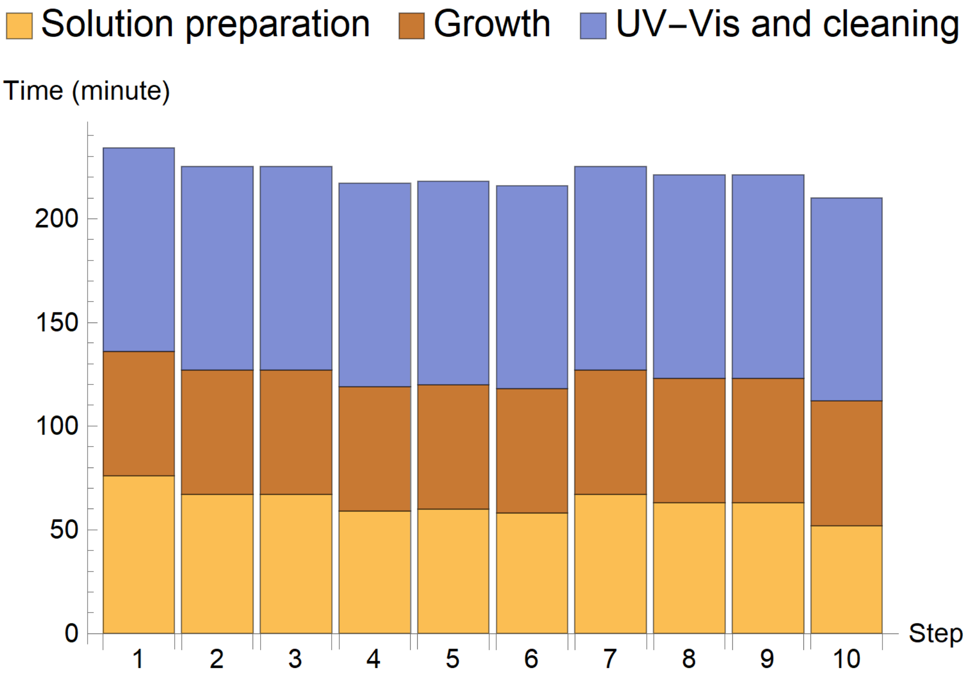

Figure S94. The time cost distribution during exploring chemical space 3 with only liquid dispensing. The time cost for solution preparation (liquid dispensing and pH control), waiting for growth and UV-Vis characterisation and cleaning are shown in different colours.

4. Optimisation towards specific optical properties

The experimental implementation of the optimisation strategy based on GS-LS will be introduced in this section. The flow diagram of the strategy is shown in **Figure S95**. The procedure for the optimisation was demonstrated as follows:

1. After exploring the chemical space, a simulated target spectrum can be reasonably generated from the available nanostructures via discrete-dipole approximation simulation. The aim is to find multiple solutions with similar UV-Vis features as this target, but with distinct synthetic conditions.
2. All data available from the previous exploration of this given chemical space is collected.
3. Data filtration:
    a. The UV-Vis is tested to make sure it is not too noisy (same as described in the exploration).
    b. Confirm that there is at least one peak present.
    c. The prominence of at least one peak is larger than a threshold (0.2).

   In this work, we are concerned with nanostructures large enough to exhibit UV-Vis peaks therefore both the target and relevant experimental spectra should contain peaks. This is the reason for the criteria (b and c) above. Sampling points with spectra failing to meet either/both of these criteria are discarded.
4. For every sample in the dataset that passes the filtration in step 3:
    a. The sample's UV-Vis will be compared with the target spectrum to measure their similarity.
    b. The local sparseness near the sample is calculated using the filtrated data.
    c. The fitness function is defined by the linear summation of the similarity and local sparseness.
5. The samples with the top N highest fitness are selected as the parents and new experiments will be designed by the crossover and mutation within the parents, together with a small portion of random sampling.
6. The autonomous platform conducts the new experiments and updates the dataset.

Step 3-6 above were iterated until the optimisation is over. The local sparseness of the sample was updated during the iteration. The final solutions, with the highest similarity metric among its K-nearest neighbours, will be selected from the dataset. They are further reproduced and characterised with TEMs to check the resulting morphologies.

The optimisation strategy was demonstrated with two cases, where the source codes for the simulations are available at https://github.com/croningp/NanoDiscovery. In this first case, the UV-

Vis from Au nanorod of a specific size was set as the target spectrum. After optimisation two samples with almost identical UV-Vis spectra but separated in the input chemical space were found to match the target. Both of them correspond to Au nanorods. In the second case, the target spectrum is simulated from Au octahedra. Multiple solutions with different morphologies including normal octahedra, concave octahedra, smooth polyhedra and mixtures were found to match the target spectrum.

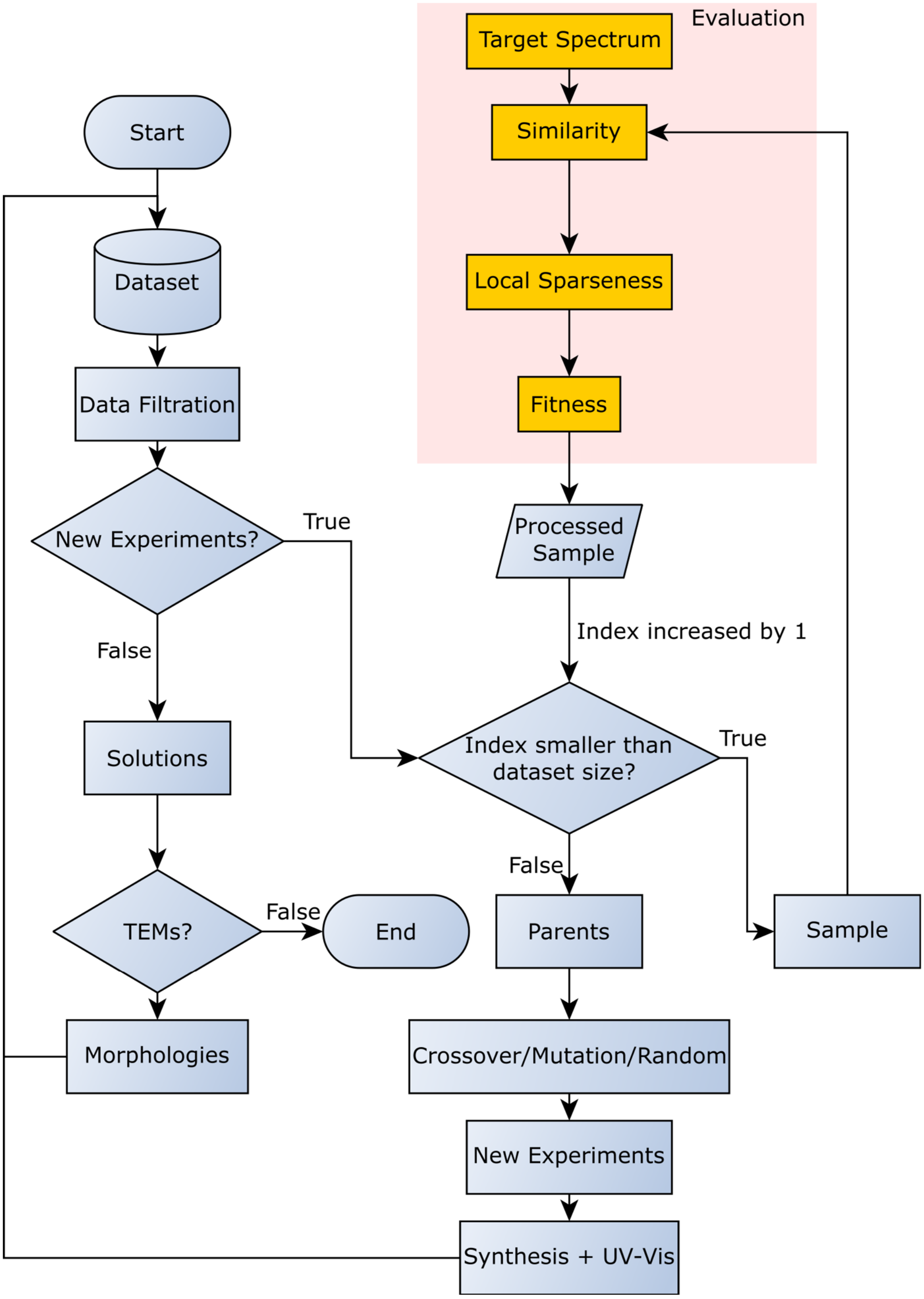

Figure S95. The flow diagram of the optimisation strategy with the autonomous platform using the optimisation algorithm based on GS-LS.

4.1. Similarity, local sparseness, and fitness

The optimisation algorithm was implemented based on GS-LS using the evolutionary algorithm (EA) as the optimiser. After setting the target spectrum, the similarity (M_S) and local sparseness (S) metrics are defined as discussed before (Eq. (1)-Eq. (3)).

$$S(x) = \frac{1}{K}\sum_{i=1}^{K} dist(\boldsymbol{x}, \boldsymbol{x'}_i) \quad (1)$$

where $dist(\boldsymbol{x}, \boldsymbol{x'}_i)$ measures the Euclidean distance between $\boldsymbol{x}$ and $\boldsymbol{x'}_i$, and $\boldsymbol{x'}_i$ is the ith closest sample to $\boldsymbol{x}$. In both optimisation cases, we evaluated the ten-nearest neighbours for local sparseness. Note that multiple points may have the same input condition, so the duplicated input points were deleted before calculating the local spareness.

$$M_S = -|p - p_{target}| - k_1 \sum_i |I_{x,i} - I_{target,i}| + k_2 \quad (2)$$

where p and p_{target} are the peak positions of the highest peak in the UV-Vis spectra of the sample and the target. $I_{x,i}$ and $I_{target,i}$ are the ith intensity of the UV-Vis data of the sample and the target respectively. We set $k_1 = 0.2$ and $k_2 = 0$ in the optimisation. It should be noted the unit of $|p - p_{target}|$ here is set as nanometer. This setting of fitness function tends to find samples with the same peak position while maintaining spectral similarity.

$$F = M_S + k_3 S \quad (3)$$

where S is the local sparseness term and k_3 was set as 100.

The top five samples with the highest fitness (F) defined by Eq. (3) were selected as the parents for crossover and mutation to generate the new experiments. Its ten nearest unique neighbours (including itself) are used to calculate the local sparseness. The local sparseness term was updated from step to step. There are 23 samples generated per step and among these 23 samples, 10 samples were mutated from the parent set, 10 samples were from crossover among parents with a further 40% chance of mutation and 3 samples were randomly generated in the input chemical space. The multi-Gaussian distribution in the mutation was set with a mean of 0 and a standard deviation of 0.08 for all dimensions. The boundary condition was maintained in a similar way as discussed above (**Figure S33**). The procedure to process the UV-Vis signal including smoothing, normalizing, and discarding spectra is the same as described in exploring chemical space 1 (**Section 3.2**). Note all the UV-Vis spectra were normalized before data processing.

After the optimisation, the solutions were selected by comparing the similarity metric with its six nearest neighbours (including itself). Only if the sample's similarity metric is no less than these

neighbours', the sample is regarded as a local maximum in the observation set and returned as a solution.

4.2. Target UV-Vis from rods

After the exploration of chemical space 1, a target spectrum was set considering the existence of nanorods. The target spectrum was from the DDA simulation of cylindrical Au nanorods with a diameter of 11 nm and a length of 33 nm. The experimental details and boundary conditions in optimisation towards Au nanorods are the same as those in chemical space 1 (**Section 3.2**). The linear transformation between input variables and reagent volumes is the same as that in **Section 3.2**. The initial data set is from the exploration of chemical space 1 (16 steps in total including the first step of random sampling). The optimisation was run for 5 steps with the initial dataset.

Stability of the system:

During the optimisation, standard samples with the unchanged condition were synthesised (**Table S17** and **Figure S96**) to track the stability of the platform.

Standard	CTAB (mL)	$HAuCl_4$ (mL)	$AgNO_3$ (mL)	Ascorbic acid (mL)	Water (mL)	Seed (mL)
**4**	4.40	2.50	1.80	1.10	1.80	0.50

Table S17. The input parameters of the standard samples to test the stability of the autonomous platform in the optimisation.

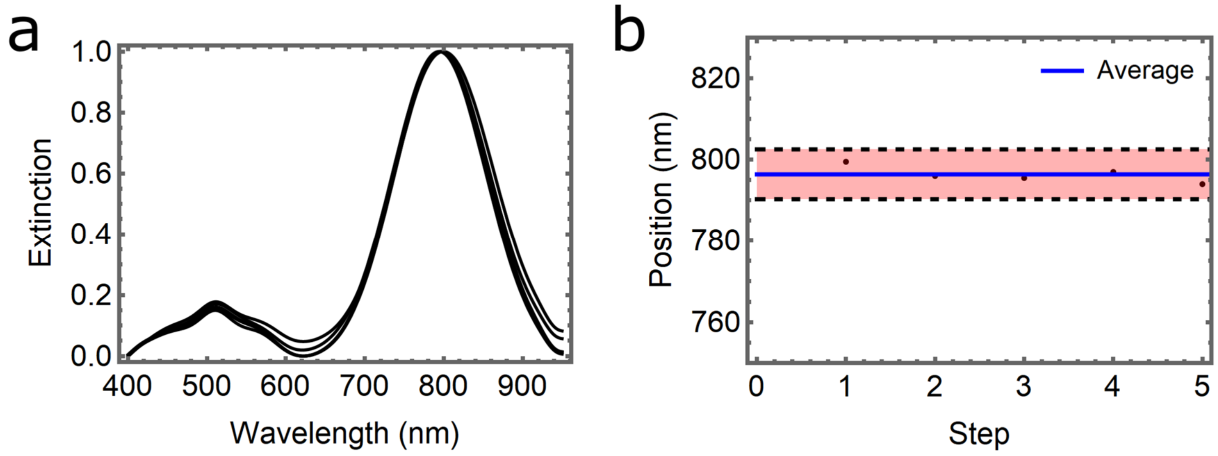

Figure S96. The demonstration of the stability of the platform. (a) The normalized UV-Vis spectra of the standard samples in different batches to measure the stability of the autonomous platform during the optimisation. (b) The different peak positions corresponding to the longitudinal mode of nanorods. The mean value is 796 nm with a standard deviation of 2 nm. All peak positions are within 3 standard deviations of their mean value as indicated by the red background.

Solution	CTAB (mL)	$HAuCl_4$ (mL)	$AgNO_3$ (mL)	Ascorbic acid (mL)	Water (mL)	Seed (mL)
**B**	4.63	2.59	2.88	1.40	0.00	0.50
**1**	5.41	2.79	1.12	1.51	0.67	0.50
**2**	4.63	2.08	1.39	1.40	2.00	0.50

Table S18. The synthetic conditions of solutions for the rod target. The reagents used here have the same concentrations as those in chemical space 1. B indicates the best solution before the optimisation.

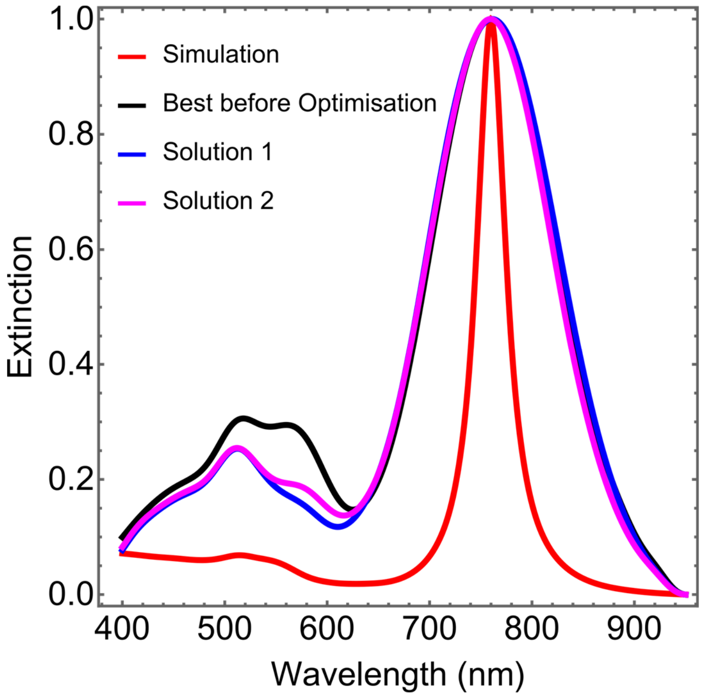

Figure S97. The UV-Vis of the target from simulation and solutions in optimisation. The similarity metric of solution 1 to 2 is -54.85 and -53.09 respectively while before optimisation, the highest similarity is -57.22.

After the optimisation, we selected the top two solutions (**Table S18**) with the highest similarity for TEM characterisation. Before the optimisation, polyhedral by-products were clearly present in this sample of the best solution as indicated in **Figure S97** and **Figure S98** (shape yield of rods is ca. 57% by counting ca. 900 nanoparticles from **Figure S98b-d**). The peak around 570 nm was due to these by-products in the sample. After the optimisation, the multiple solutions showed a decreased by-product peak with similar desired UV-Vis features. TEM characterisation indicates the UV-Vis features were due to Au nanorods (**Figure S99-Figure S100**). Solution 1 shows the higher yield of target nanorods (shape yield of rods is ca. 95% by counting ca. 1100 nanoparticles from **Figure S99a** and **b**), which is consistent with the small by-product peak as observed in the UV-Vis. Solution 2, despite exhibiting close similarity to the target and a marginally higher fitness than solution 1, was revealed to have the lesser nanorods of the two solutions. This reveals the strength of this method, in

that it will provide multiple solutions to a problem, increasing the robustness of producing a truly optimised sample.

These two solutions were selected by the criterion of K-nearest neighbours so that they have the highest similarity metric compared to its six nearest neighbours (including itself). In calculating the distance between sampling points, the normalized variables in the range from 0 to 1 instead of the reagent volumes were used. The synthetic conditions of both solutions together with their nearest neighbours were labelled as cross and red points respectively in **Figure S101**. The similarity metric distributions of the rest samples with various synthetic conditions are shown as well.

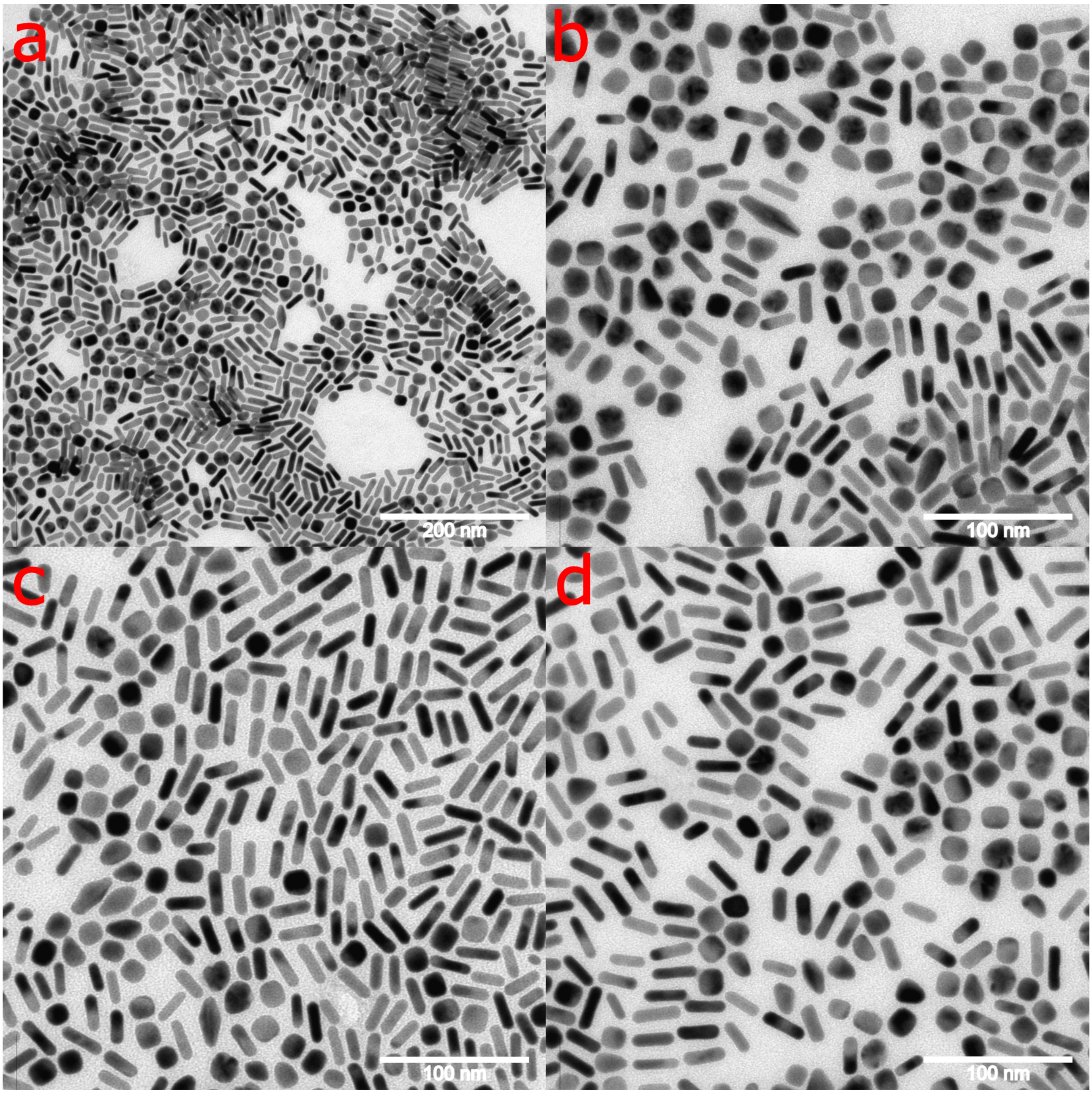

Figure S98. The TEM images of the best solution before optimisation. The scale bars are shown in the images.

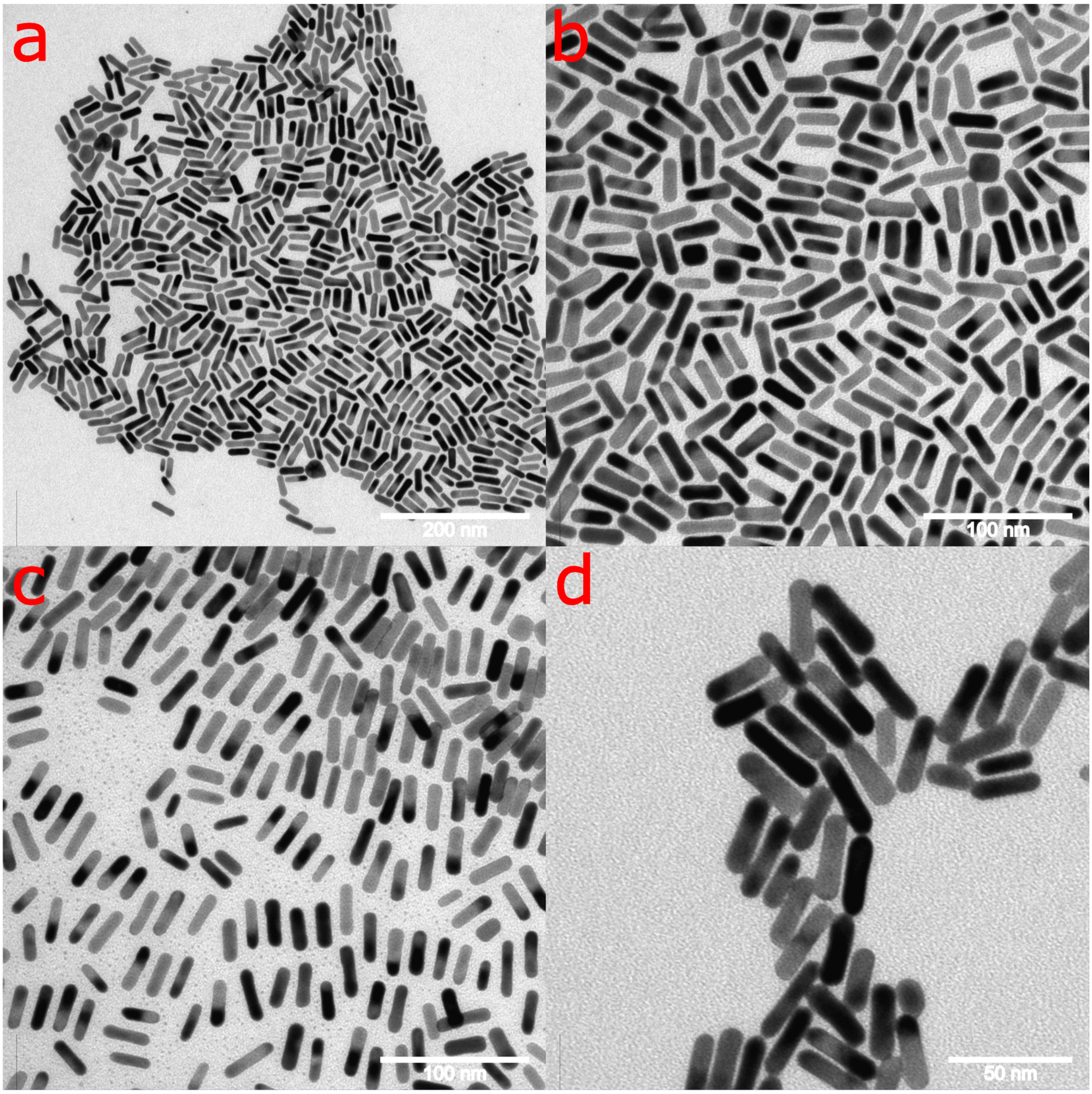

Figure S99. The TEM images of solution 1 in optimisation. The scale bars are shown in the images.

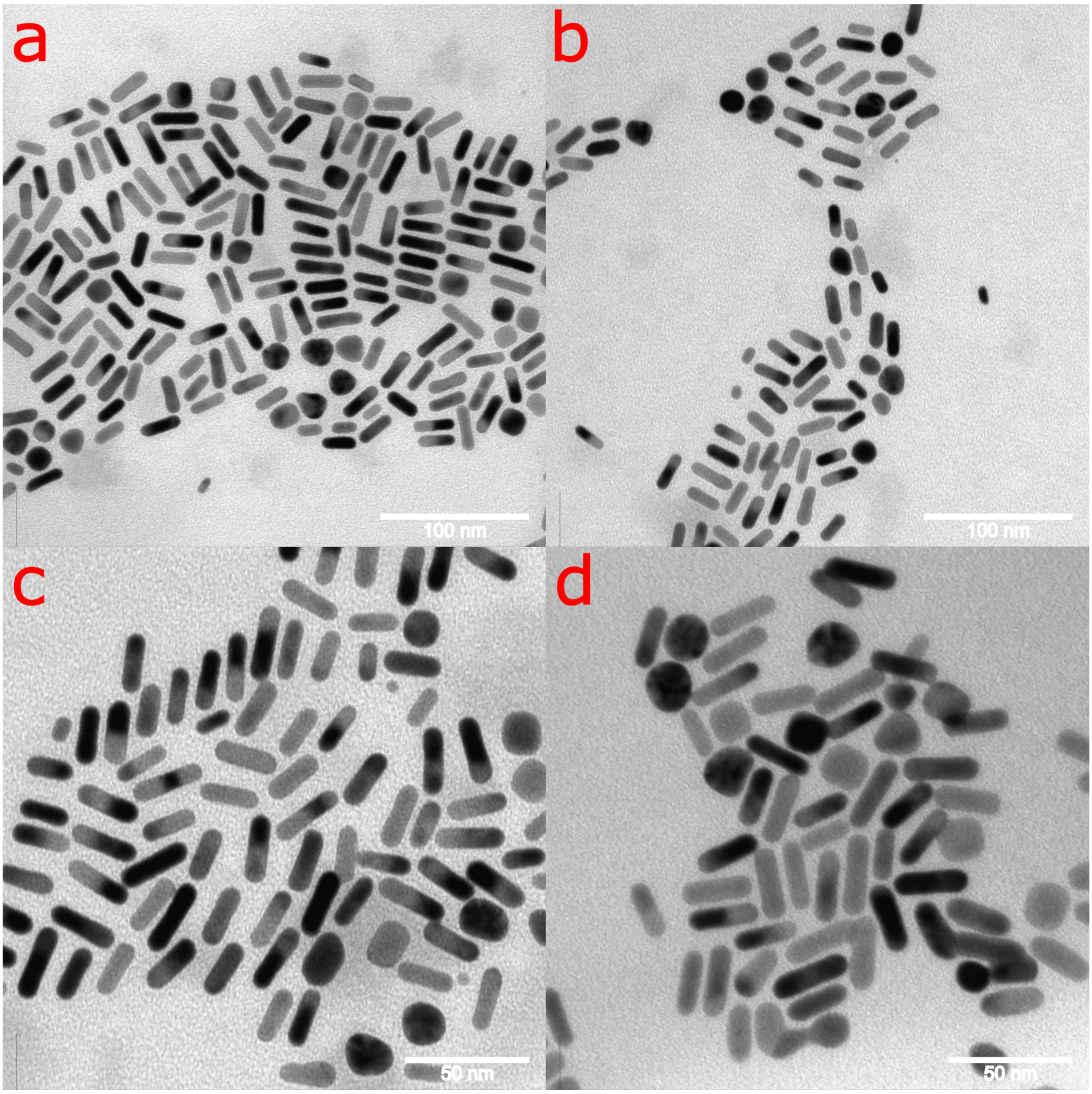

Figure S100. The TEM images of solution 2 in optimisation. The scale bars are shown in the images.

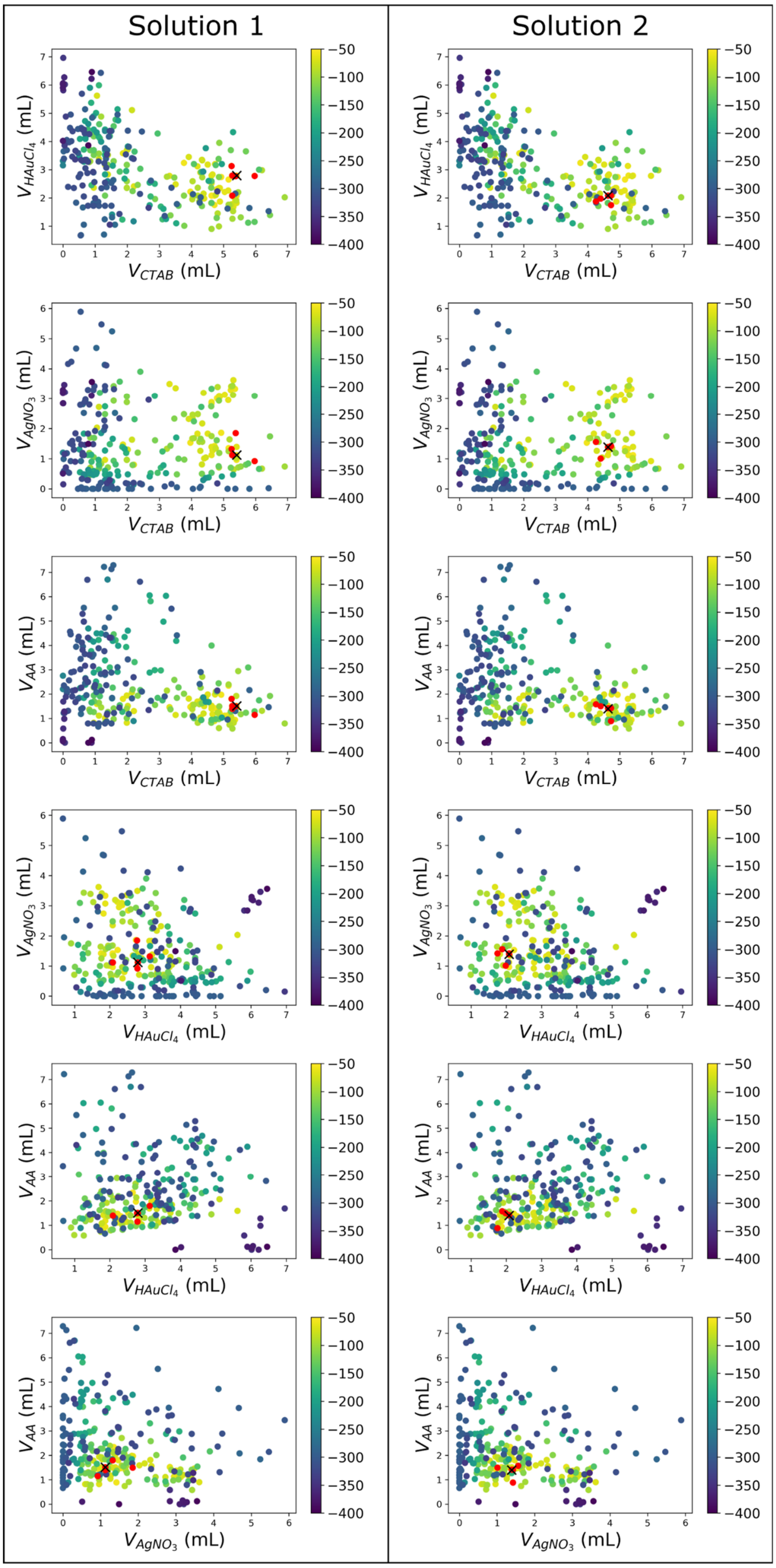

Figure S101. The synthetic condition distribution of the top two solutions from the optimisation. The solutions are labelled by the cross and their neighbours are labelled by the red points. The similarity metric distributions of the rest of the samples are shown in the same figure. The colour bars show the similarity metrics of samples and are in the range from -400 to -50. AA: ascorbic acid.

4.3. Target UV-Vis from octahedra

After exploration of chemical space 3, the emergence of octahedral features was observed in Au nanostars (L3-1, see **Figure S65**), and octahedral nanoparticles were observed in another sample (Elite 3, see **Figure S66**). Thus, a target spectrum from the DDA simulation of Au octahedra with a longer axis length of ca. 80 nm (edge length of $\frac{80}{\sqrt{2}} \approx 57$ nm) was set. The optimisation was expected to amplify the octahedral feature in this chemical space. The experimental details and boundary conditions in the optimisation towards octahedra are the same as those in chemical space 3 (**Section 3.4**) except for the concentration of $HAuCl_4$ which is halved to 0.43 mM. This is because after exploring chemical space 3, all the available data were compared with the target spectrum. The volumes of $HAuCl_4$ used in the best five samples are all below 1.00 mL, as a result, the chemical space was shrunk in the optimisation.

The linear transformation between input variables in the algorithm and volumes of the reagents is the same as described in **Section 3.4**. The only difference was the concentration of $HAuCl_4$, which means the maximum concentration of $HAuCl_4$ in the growth solution was halved compared to that during exploring chemical space 3. Thus, the chemical space where the optimisation will happen was shrunk. As a result, only data from the original chemical space 3 dataset, within the bounds of the shrunk chemical space were used. The optimisation was run for 5 steps starting with the initial dataset.

Again, standard samples with the unchanged synthetic condition in the same shrunk chemical space were used to track the stability of the autonomous platform (**Table S19** and **Figure S102**).

**Standard**	**CTAC (mL)**	**$HAuCl_4$ (mL)**	**$AgNO_3$ (mL)**	**HCl (mL)**	**Ascorbic acid (mL)**	**Water (mL)**	**Seed (mL)**
**5**	1.57	1.43	1.37	0.60	5.00	1.53	0.50

Table S19. The input parameters of the standard samples to test the stability of the autonomous platform in the optimisation.

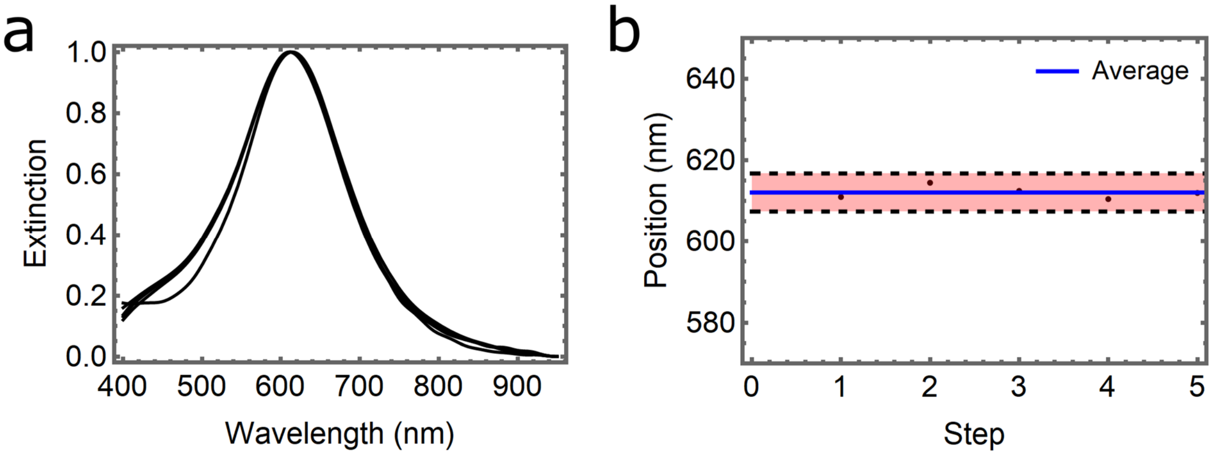

Figure S102. The demonstration of the stability of the platform. (a) The normalized UV-Vis spectra of the standard samples in different batches to measure the stability of the autonomous platform during the optimisation. (b) The different peak positions from the standard samples. The mean value is 612 nm with a standard deviation of 2 nm. All peak positions are within 3 standard deviations of their mean value as indicated by the red background.

**Solution**	**CTAC (mL)**	**$HAuCl_4$ (mL)**	**$AgNO_3$ (mL)**	**HCl (mL)**	**Ascorbic acid (mL)**	**Water (mL)**	**Seed (mL)**
**1**	0.75	0.50	4.15	2.25	1.24	2.61	0.50
**2**	2.43	1.26	0.19	0.31	5.51	1.80	0.50
**3**	0.12	0.63	2.08	5.08	0.59	3.00	0.50
**4**	1.22	1.26	0.19	0.31	5.51	3.01	0.50
**5**	4.24	0.91	0.76	2.57	2.89	0.13	0.50

Table S20. The synthetic conditions of the solutions for the octahedral target. The reagents used here have the same concentrations as those in chemical space 3 except that the concentration of $HAuCl_4$ was decreased to half (0.43 mM).

After the optimisation, all the data (from both exploration and optimisation) in the shrunk chemical space were used further to give the final solutions. Again, the K-nearest neighbour criterion was implemented to find local maxima regarding the observation set. Each solution was selected so that its similarity metric was no less than its six nearest neighbours (including itself). The synthetic conditions of the top five solutions with the highest similarity and their corresponding UV-Vis spectra are shown in **Table S20** and **Figure S103**. The TEM images of these five solutions are shown in **Figure S104**-**Figure S108**. The distribution of the five solutions in the chemical space and their corresponding neighbours are shown in **Figure S109**-**Figure S113**.

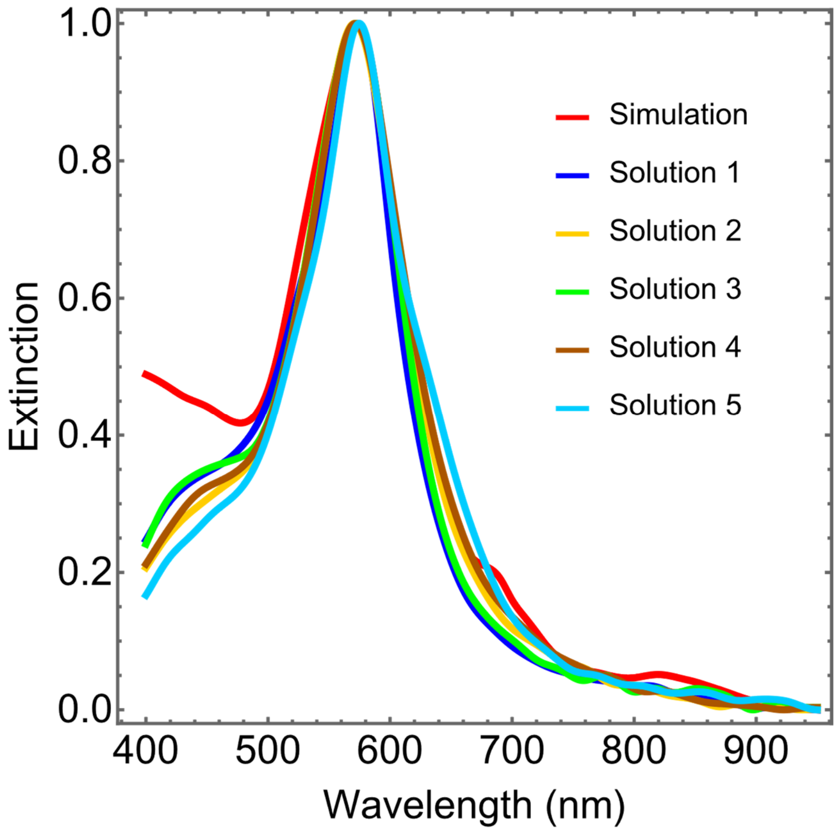

Figure S103. The UV-Vis of the target from simulation and solutions in optimisation. The similarity metric of solution 1 to 5 is -12.17, -10.97, -10.14, -10.07 and -13.92 respectively.

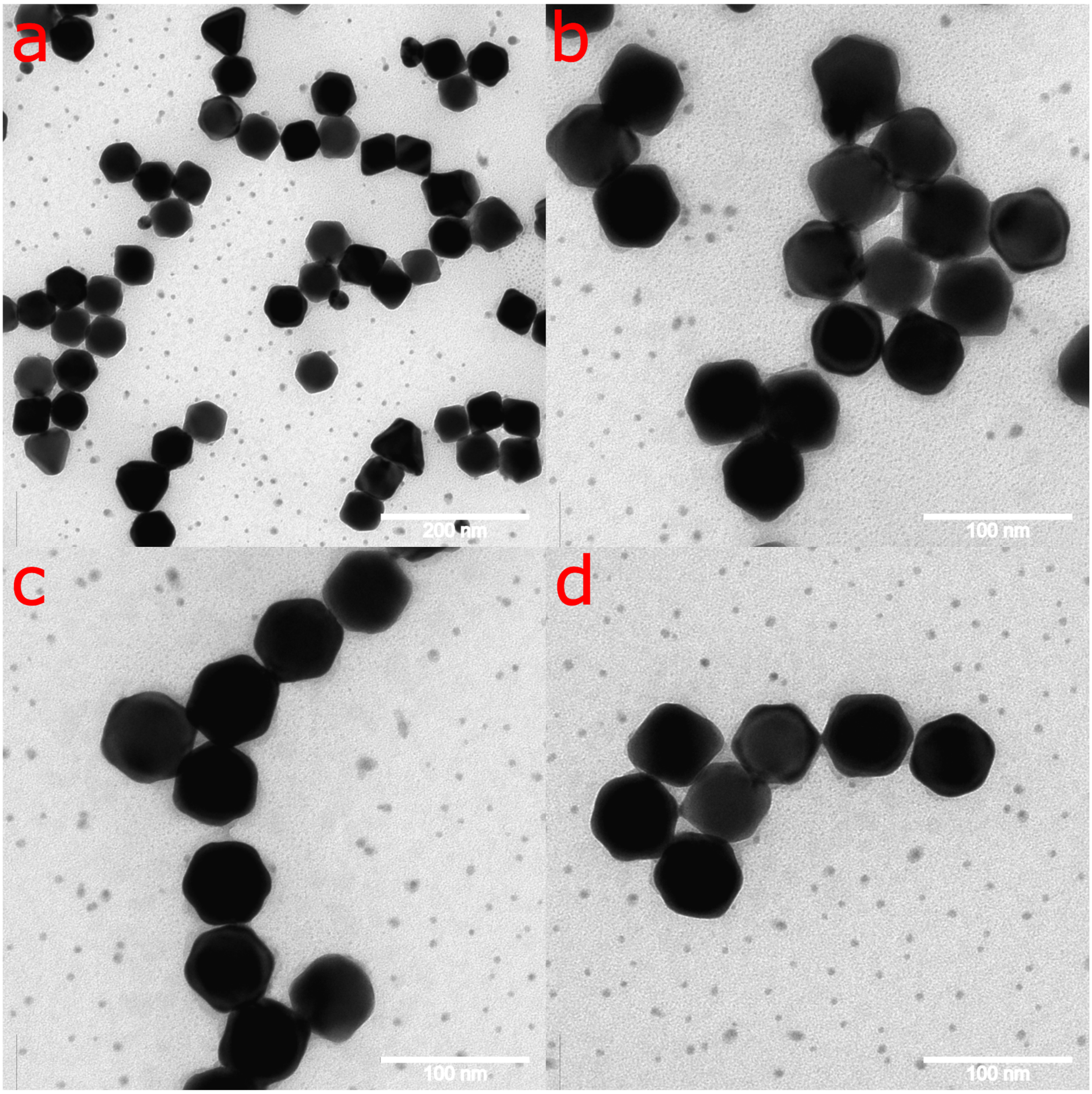

Figure S104. The TEM images of solution 1 (octahedral nanoparticles) in optimisation. The scale bars are shown in the images.

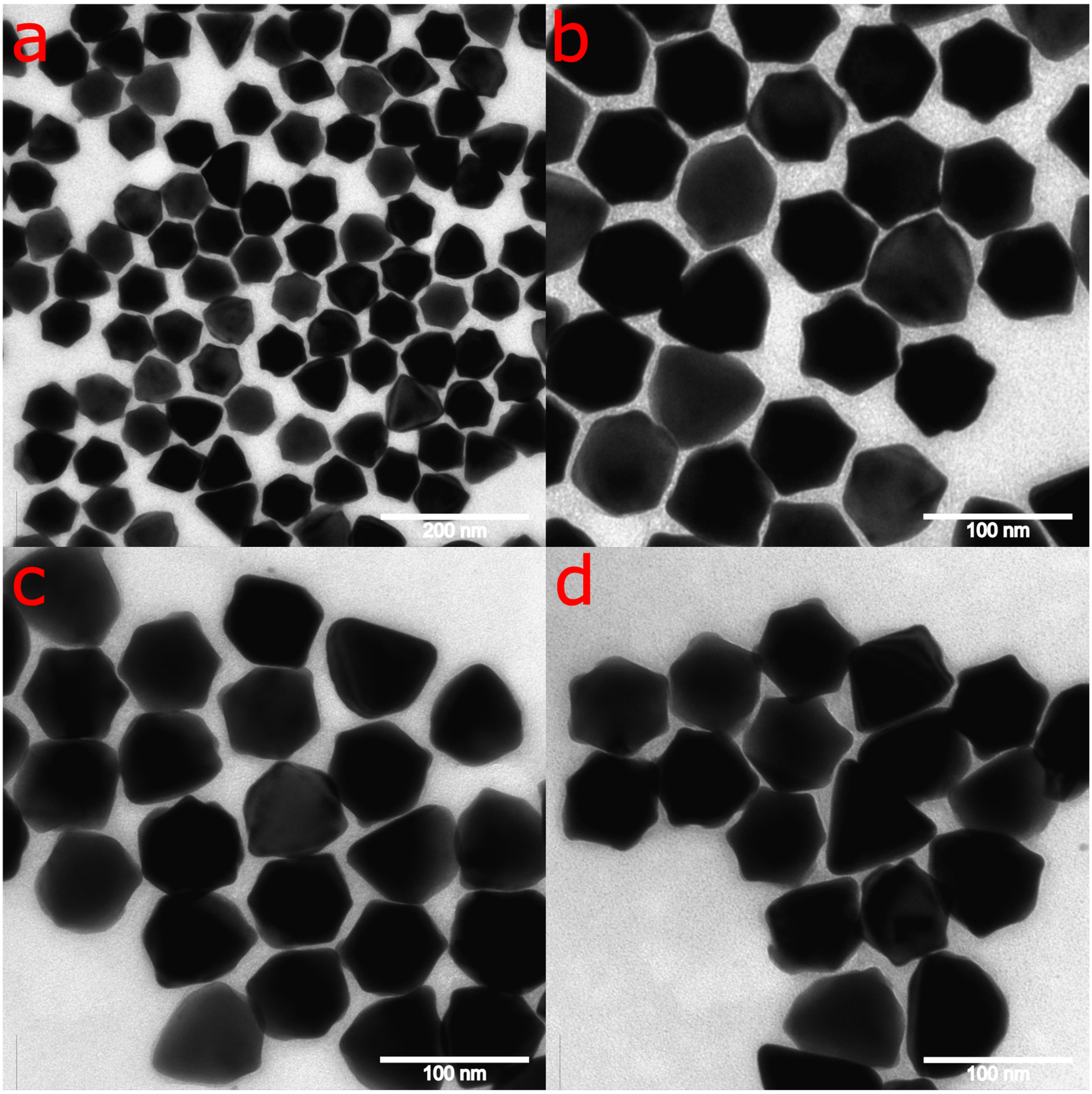

Figure S105. The TEM images of solution 2 (octahedral nanoparticles) in optimisation. The scale bars are shown in the images.

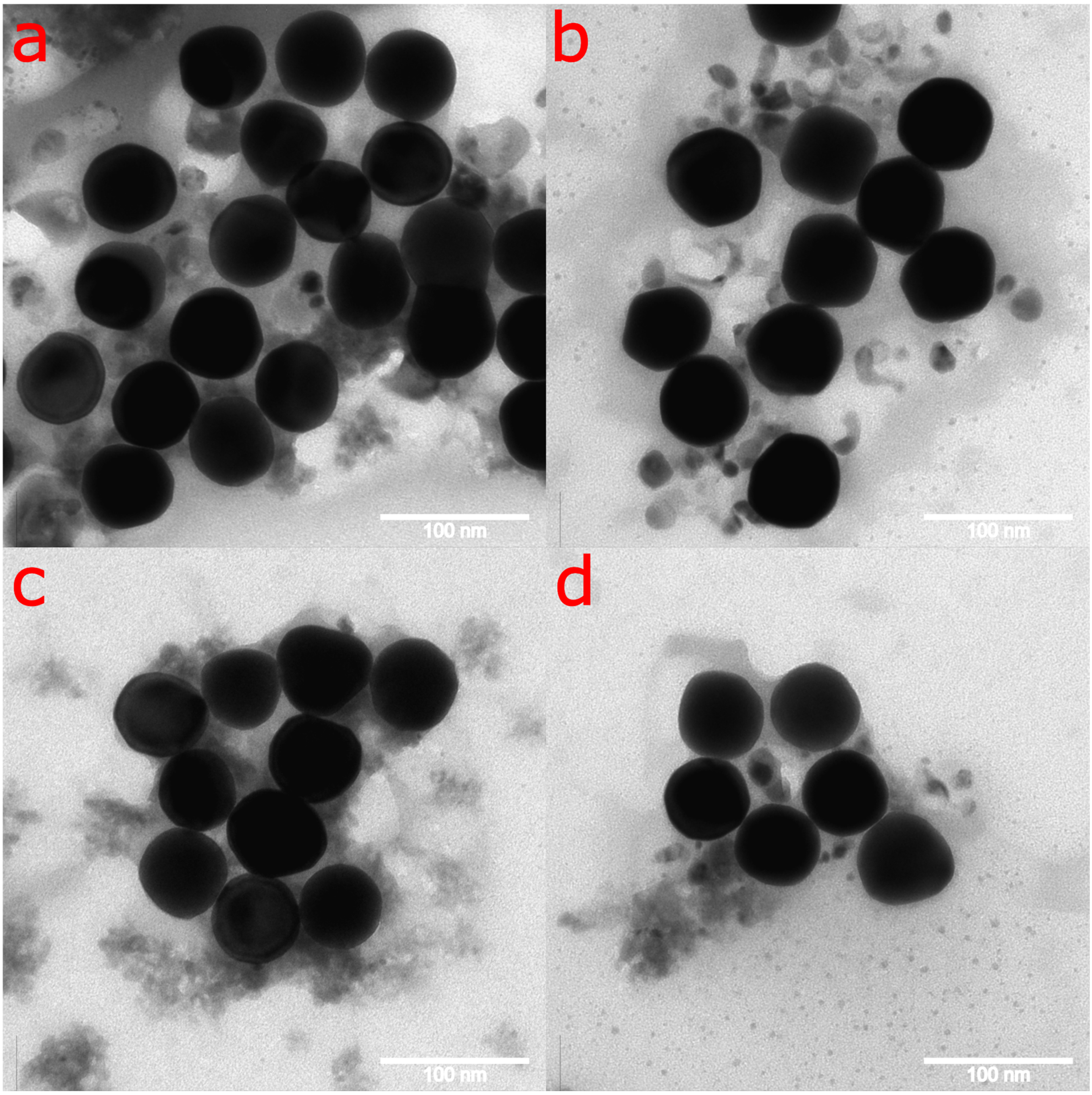

Figure S106. The TEM images of solution 3 (smooth polyhedral nanoparticles) in optimisation. The scale bars are shown in the images.

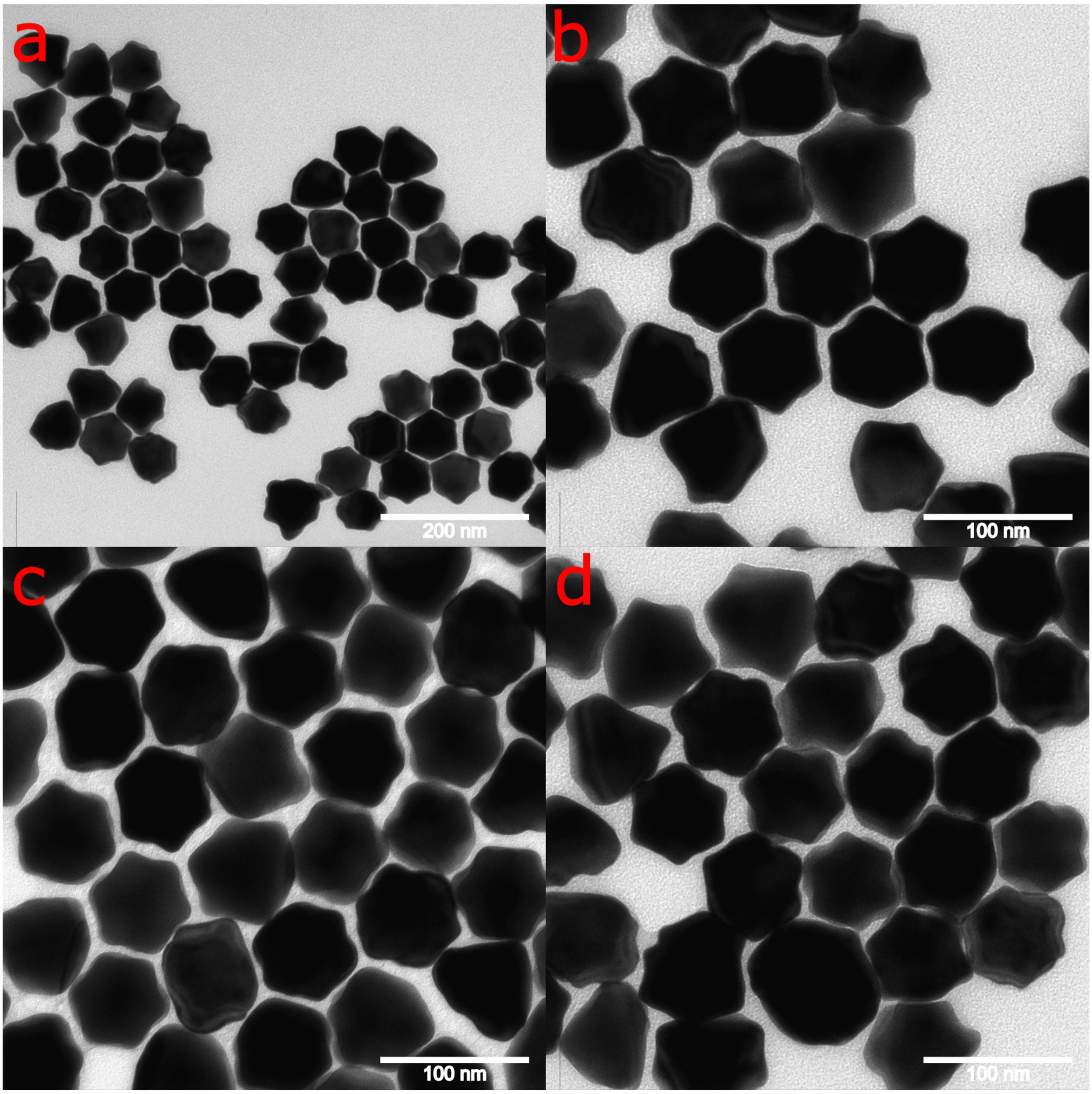

Figure S107. The TEM images of solution 4 (octahedral nanoparticles) in optimisation. The scale bars are shown in the images.

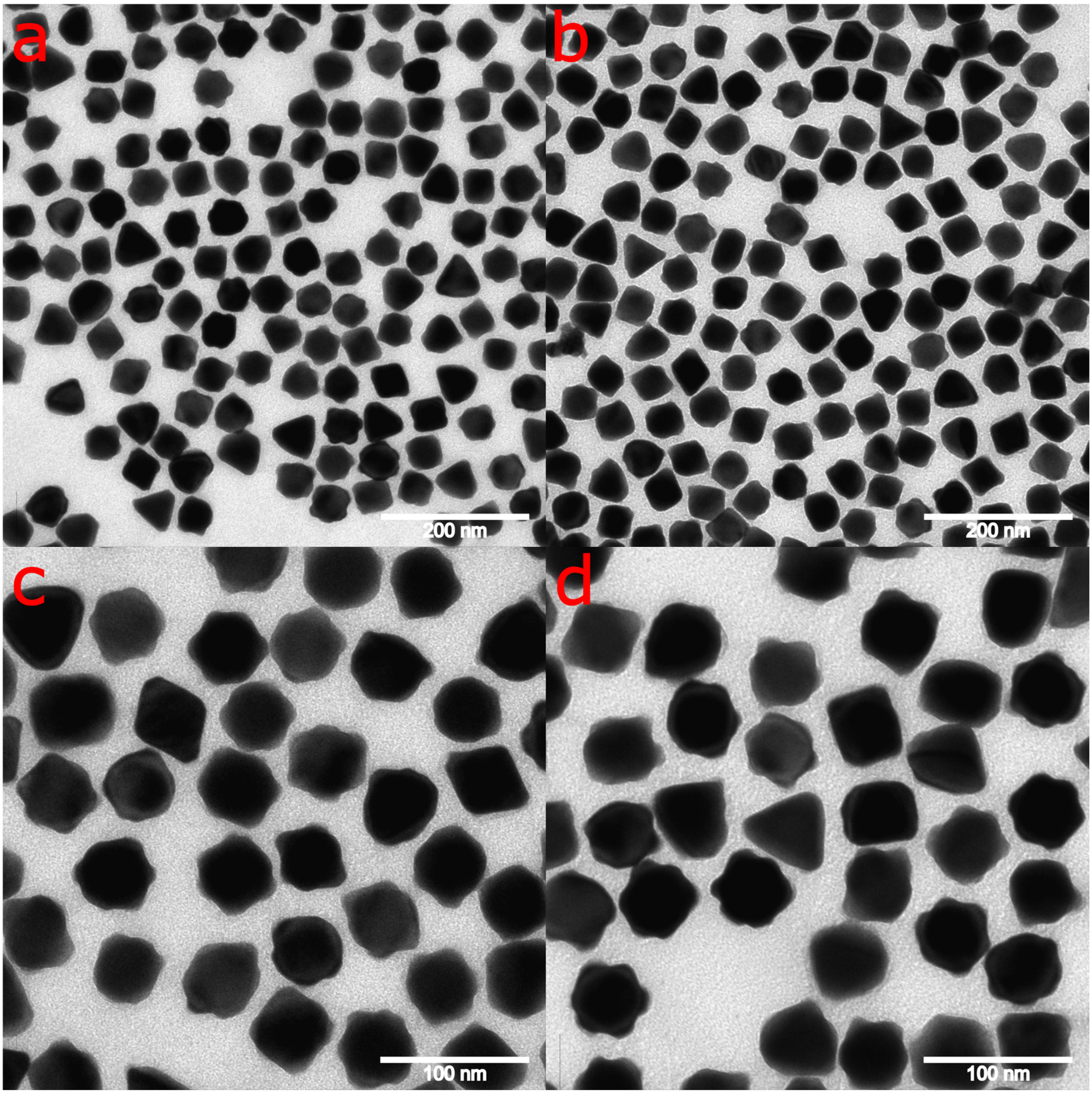

Figure S108. The TEM images of solution 5 (a mixture of multiple polyhedral nanoparticles) in optimisation. The scale bars are shown in the images.

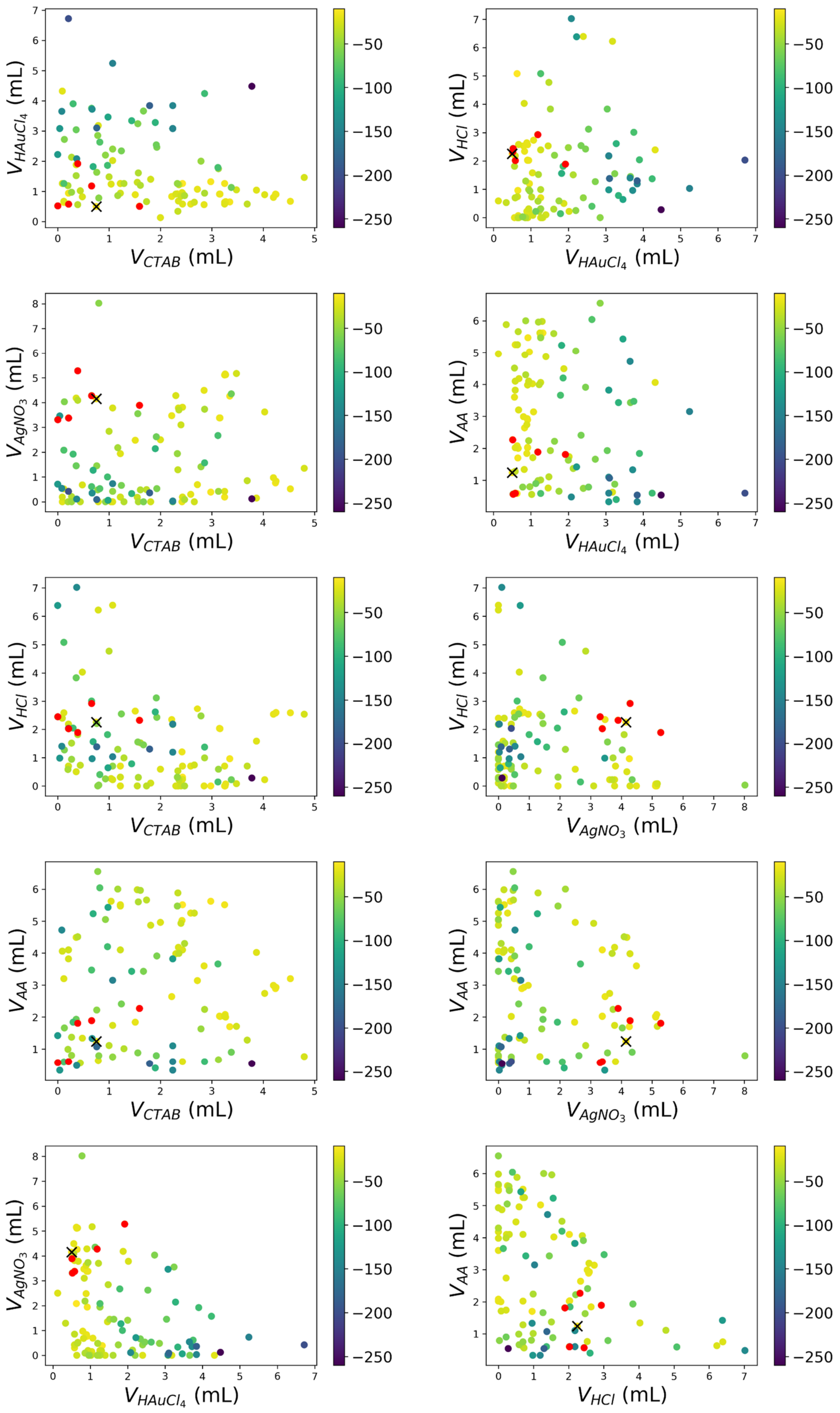

Figure S109. The synthetic condition distribution of solution 1 from the optimisation. The solution is labelled by the cross and its neighbours are labelled by the red points. The similarity metric distribution of the rest of the samples is shown in the same figure. The colour bars show the similarity metrics of samples and are in the range from -260 to -10. AA: ascorbic acid.

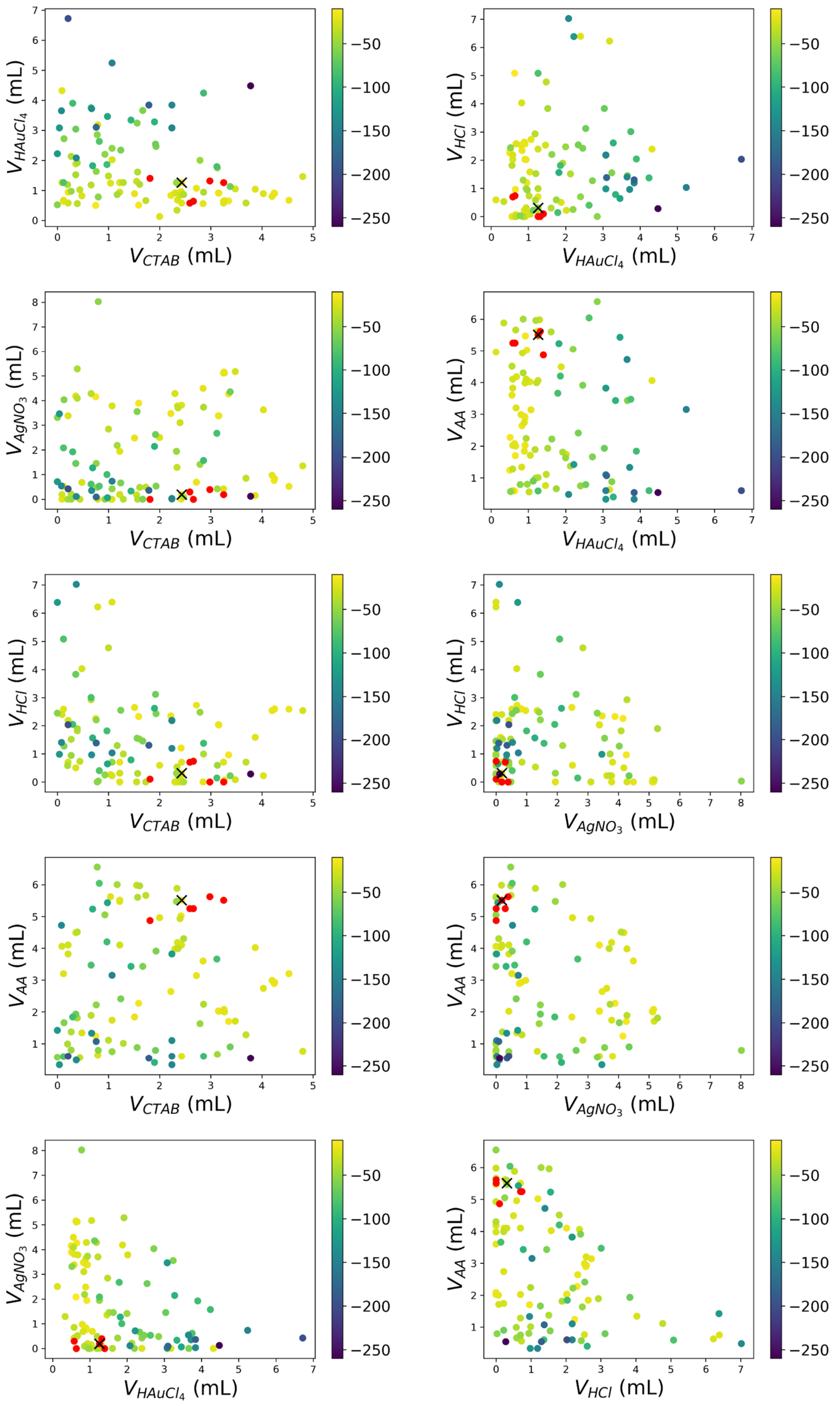

Figure S110. The synthetic condition distribution of solution 2 from the optimisation. The solution is labelled by the cross and its neighbours are labelled by the red points. The similarity metric distribution of the rest of the samples is shown in the same figure. The colour bars show the similarity metrics of samples and are in the range from -260 to -10. AA: ascorbic acid.

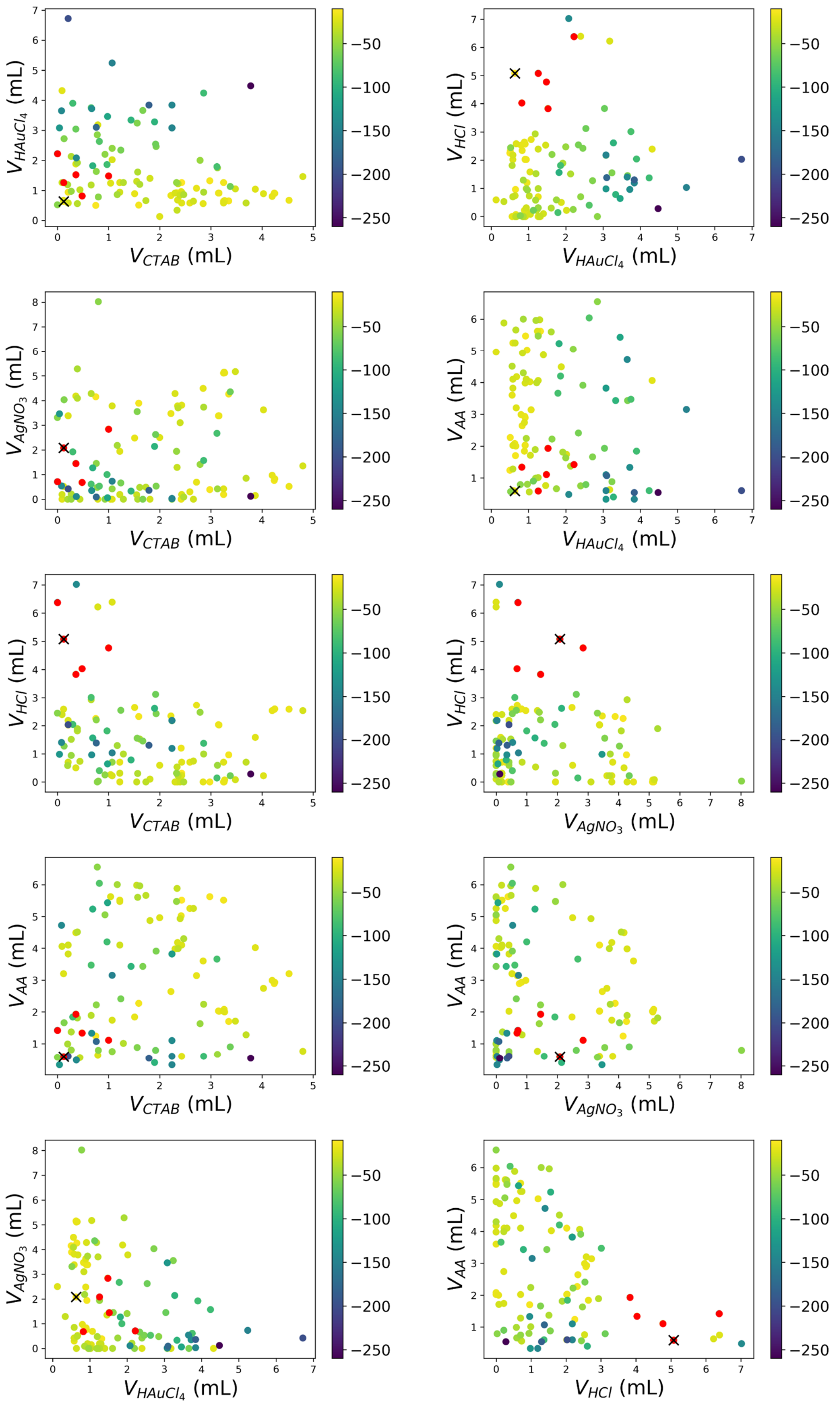

Figure S111. The synthetic condition distribution of solution 3 from the optimisation. The solution is labelled by the cross and its neighbours are labelled by the red points. The similarity metric distribution of the rest of the samples is shown in the same figure. The colour bars show the similarity metrics of samples and are in the range from -260 to -10. AA: ascorbic acid.

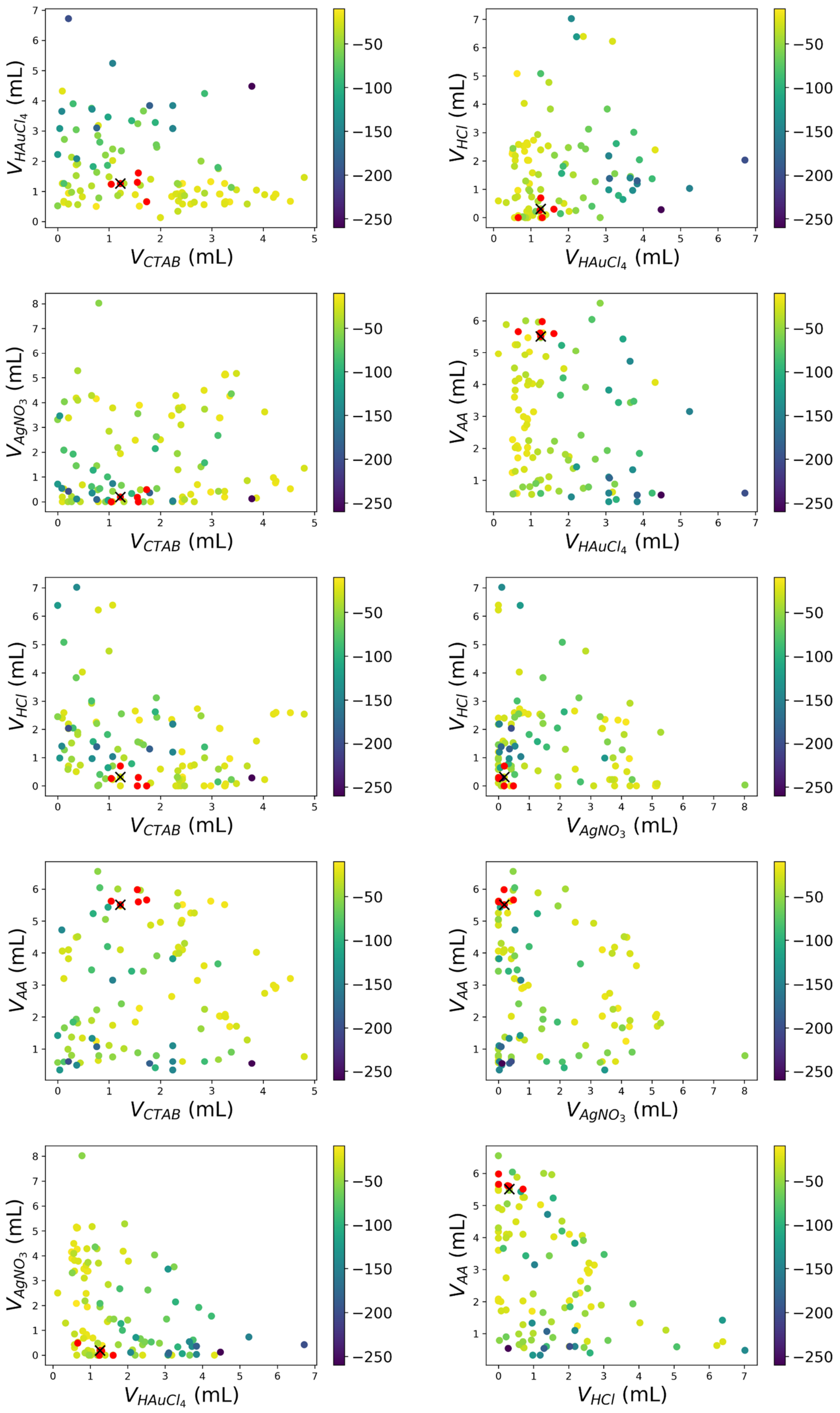

Figure S112. The synthetic condition distribution of solution 4 from the optimisation. The solution is labelled by the cross and its neighbours are labelled by the red points. The similarity metric distribution of the rest of the samples is shown in the same figure. The colour bars show the similarity metrics of samples and are in the range from -260 to -10. AA: ascorbic acid.

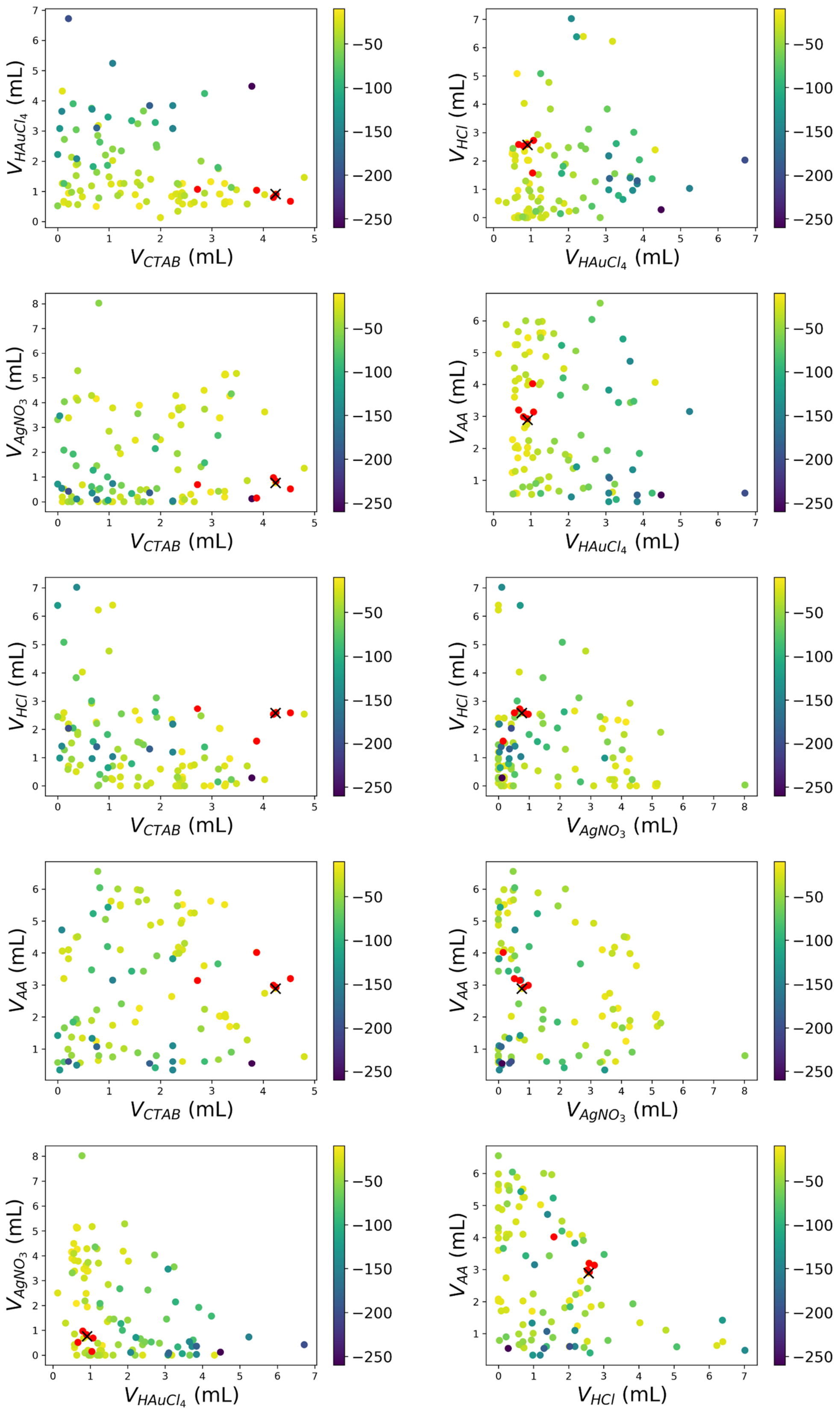

Figure S113. The synthetic condition distribution of solution 5 from the optimisation. The solution is labelled by the cross and its neighbours are labelled by the red points. The similarity metric distribution of the rest of the samples is shown in the same figure. The colour bars show the similarity metrics of samples and are in the range from -260 to -10. AA: ascorbic acid.

5. Autonomous synthesis of nanoparticles via multistep growth

In this section, we will introduce the fully autonomous synthesis of multiple batches of AuNPs many of which are both products and required as seeds for nanoparticles of higher complexity. Their unique digital signatures would be generated after the synthesis. Considering the synthesis of nanoparticles, three steps are necessary:

1. Define the desired nanoparticles and their hierarchical relationship as a synthetic network.
2. Design many identical reactions that are needed to satisfy the multiple requirements of a single nanoparticle. Several samples would be needed for analysis, as seeding materials for the next reaction level and for potential applications. Repeated parallel reactions to generate enough volume of one nanoparticle must therefore exist.
3. Define the necessary operations that are required to conduct the chemical reactions on the autonomous platform.

Directed graphs can handle complicated networks and are easy to visualize. Thus, they are used to satisfy the three steps above.

5.1. The directed synthesis graph, reaction graph and hardware graph

The three directed graphs were defined as follows:

1. **The directed synthesis graph**. A graph is created according to the synthetic routines for multiple nanoparticles. The nanoparticles are represented by nodes. The hierarchical relationship between nanoparticles, which is defined by using one nanoparticle as the seed for another, is indicated by the directed edges among the nodes.
2. **The directed reaction graph**. A graph is automatically generated using **the synthesis graph** to determine the experimental design. In designing the experiments, the multiple repeated syntheses of the same nanoparticle for its UV-Vis characterisation or seeding of new reactions are considered. The node in the reaction graph represents one sample of a nanoparticle, while the directed edges indicate which samples will be used as the seeds for new reactions.
3. **The directed hardware graph**. A graph is generated from **the reaction graph** and used to define the operations that will be conducted on the platform. The available experimental resources from the hardware are distributed among the parallel synthesis of multiple samples. In our system, this graph represents the distribution of samples in the available vials on the wheel, defines the seed transfer operation among the samples, and assigns samples for UV-Vis characterisation. All the operations to be executed on the platform are defined by the hardware graph.

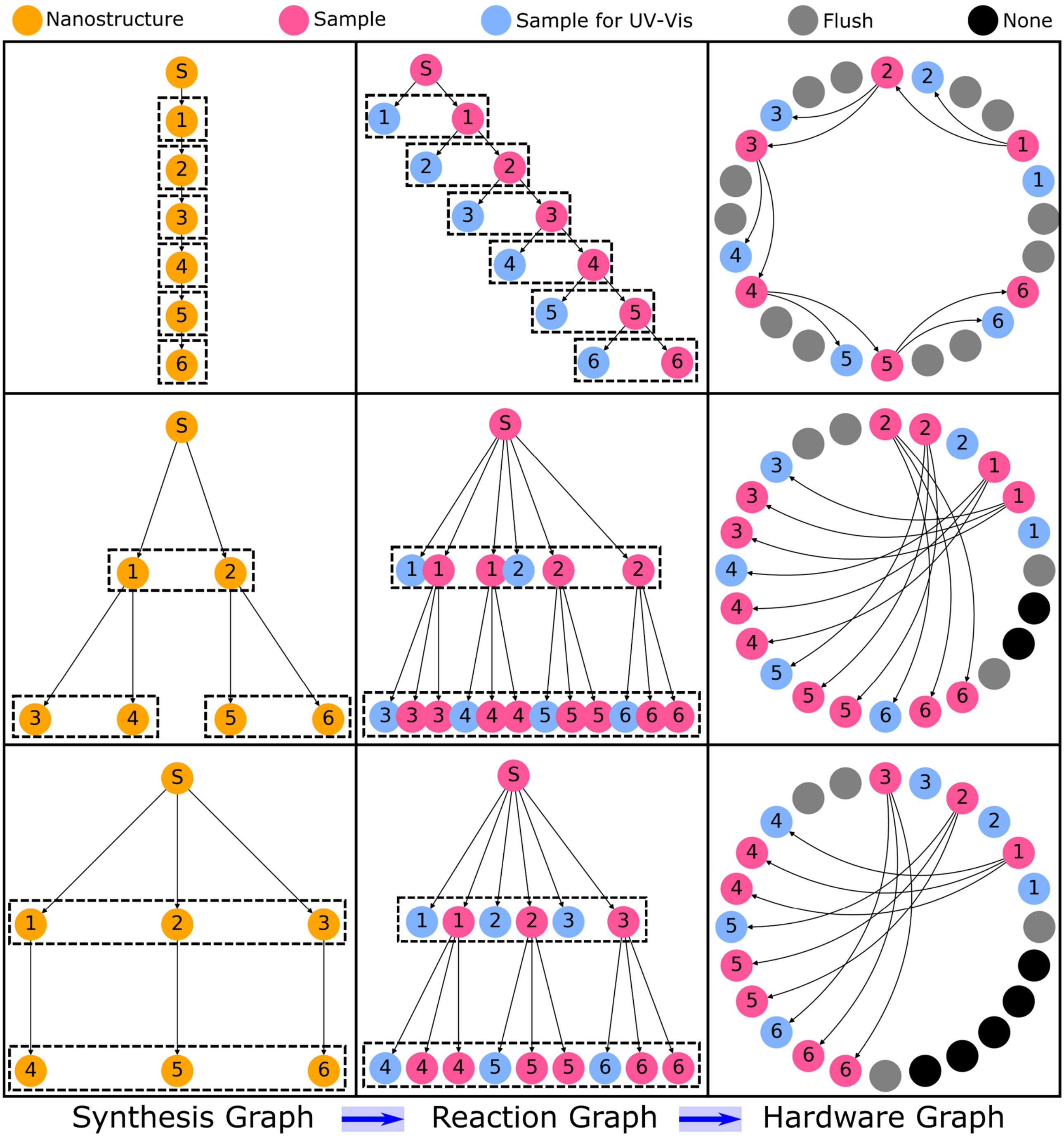

Figure S114. Examples of the synthesis graph, reaction graph and the corresponding hardware graph. The synthesis graph is composed of multiple desired nanoparticles and their hierarchical relationships. The reaction graph designs the chemical reactions according to the synthesis graph. The hardware graph distributes the resources of the hardware for the parallel synthesis of multiple samples and defines the seed solution transfer among samples. The slots with grey or dark colour in the hardware graph indicate that they are used to flush the system or not used in the experiments, respectively. The initial seed was labelled as S.

With the directed graph structure, the platform can handle various synthetic networks containing multiple nanoparticles performed in parallel. The synthesis graphs of different synthetic networks are shown in **Figure S114**, **left column**. The chemical reactions to achieve multiple nanoparticles in different networks were designed according to the synthesis graph and recorded in the reaction graph

(**Figure S114**, **middle column**). Finally, the hardware graph was generated according to the reaction graph. The samples to be synthesised were mapped to the available slots on the wheel. The seed transfer operations from one sample to others were then defined (**Figure S114**, **right column**).

The number and duration of the growth steps to reach different nanoparticles can vary. Depending on the number of steps, the nanoparticles are divided into different batches, which are indicated by the different layers in the synthesis graph or reaction graph (**Figure S114**, **left and middle columns**). The synthesis was conducted batch by batch. Between batches, UV-Vis was used to validate the sample reproducibility before using it as the seeds. Since multistep synthesis may require a long growth time from batch to batch, pre- and post-reaction wash/flush routines were performed after each batch of reactions to ensure no contamination in transfer. Note that depending on the synthesis graph, not all of the vial slots are used in the complete process. See the hardware graphs in **Figure S114 middle and bottom rows** for examples.

The graph representation of the synthesis of many nanoparticles with clearly defined chemical reactions and the set-up of the hardware offers a generic way to both represent and set up the multistep synthesis in our system. The source code to generate the graphs is available at https://github.com/croningp/NanoDiscovery.

5.2. The autonomous synthesis of desired nanoparticles

To validate the directed graph strategy described above and the reproducibility of the autonomous platform, we synthesised six nanoparticles of varying shapes including rods, spheres and stars, which were all discovered in the exploration. They are labelled from N1 to N6 (which correspond to L1-5, L1-1, L2-12-12-2, L2-7, L3-3 and L3-1 as discussed before). N1 and N2 which correspond to small Au nanorods and nanospheres are from chemical space 1; N3 and N4 which correspond to large Au nanospheres and nanorods are from chemical space 2; N5 and N6 which correspond to Au nanostars are from chemical space 3. Up to three steps of growth were required to complete this series which can be seen in **Figure S115**.

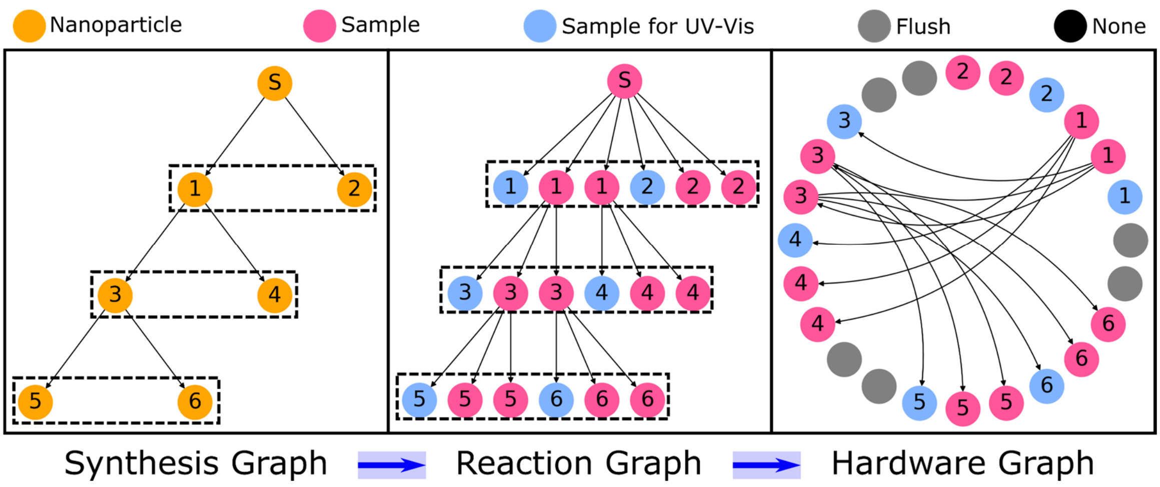

Figure S115. The synthesis graph, reaction graph and hardware graph for the multistep synthesis of six nanoparticles. Every nanoparticle was repeated three times so there were 18 experiments in total. The sample that will be characterised with UV-Vis was labelled as blue. The initial seed was labelled as S.

5.2.1. Experimental details

Chemicals and stock solutions:

- Type I ultrapure water. (18.4 MΩ•cm)
- Hexadecyltrimethylammonium bromide, >99 %, Acros Organics, CTAB. (0.2 M)
- Hexadecyltrimethylammonium chloride, >99 %, Acros Organics, CTAC. (0.2 M)
- Gold (III) chloride trihydrate, 99.9 %, Sigma-Aldrich, $HAuCl_4$.
  (0.86 mM for overgrowth solution and 0.43 mM for the initial seed preparation)
- Ascorbic acid, 99.9%, Sigma-Aldrich (13.1 mM, four times as concentrated as that from chemical space 1)
- Silver nitrate, 99.9999 %, Sigma-Aldrich, $AgNO_3$ (0.25 mM)
- Hydroquinone, 99.5%, Acros Organics. (13.1 mM)
- Hydrochloric acid, ACS Reagent, 37%, Honeywell Fluka™, diluted with Type I water. (0.1 M)
- Sodium hydroxide, 98%-100.5%, Honeywell Fluka™, NaOH. (0.1 M)

The initial 2 nm Au seed was synthesised as described in **Section 3.2**. Note ascorbic acid (13.1 mM) was used in the multistep synthesis and the volumes of the reagents were changed accordingly to maintain their concentrations to the original synthetic conditions. The waiting times for three batches of N1-N2, N3-N4 and N5-N6 were 2, 16 and 1 hours, respectively. They enabled that Batch 1/3 can grow for at least 90 minutes, and Batch 2 can grow for at least 16 hours, which are adequate to complete the growth. The synthesis of every nanoparticle was repeated 3 times, and one of the repeats was used for the UV-Vis characterisation. Here, the water reference of the UV-Vis was taken before pumping in the samples, where the flow cells were cleaned and filled with Type I water. The synthetic conditions of these six nanoparticles are listed in **Table S21**.

**Batch 1 (wait for 2 hours)**						
**Label**	**CTAB (mL)**	**$HAuCl_4$ (mL)**	**$AgNO_3$ (mL)**	**Ascorbic acid (mL)**	**Water (mL)**	**Seed (mL)**
**N1 (L1-5)**	4.66	2.83	1.27	0.29	2.45	0.50
**N2 (L1-1)**	1.51	2.53	0.09	1.78	5.59	0.50
**Batch 2 (wait for 16 hours)**						

Label	CTAB (mL)	Hydroquinone (mL)	Water (mL)	pH	$AgNO_3$ (mL)	$HAuCl_4$ (mL)	Seed (mL)
**N3 (L2-12-2)**	1.85	1.07	4.08	6.68	0.07	3.09	0.50
**N4 (L2-7)**	4.17	2.54	0.29	4.22	2.30	2.70	0.50
**Batch 3 (wait for 1 hour)**							
**Label**	**CTAC (mL)**	**$HAuCl_4$ (mL)**	**$AgNO_3$ (mL)**	**HCl (mL)**	**Ascorbic acid (mL)**	**Water (mL)**	**Seed (mL)**
**N5 (L3-3)**	6.81	1.92	0.03	1.19	0.60	0.95	0.50
**N6 (L3-1)**	2.66	0.32	0.00	0.74	5.25	2.53	0.50

Table S21. The synthetic conditions of N1 to N6. Note N1 is the seed for N3 and N4, and N3 is the seed for N5 and N6. When reproducing N3 and N4, the operations of adding acid or base sequentially were conducted as recorded in preparing the original solutions without measuring the pH. Note ascorbic acid (13.1 mM) was used. The waiting time between the batch synthesis and the UV-Vis analysis is also shown in the table.

5.2.2. The graph representation of N1 to N6

The corresponding synthesis graph, reaction graph and hardware graph are shown in **Figure S115**. The autonomous multistep synthesis was repeated three times independently to validate the stability of the system. The average highest peak position (including three repeats and the original spectrum from exploration) with their standard deviation are shown in **Table S22**. These reproduced spectra were only normalized to the range from 0 to 1 without being processed by the low-pass filter to reflect the possible detection noise. The comparison of UV-Vis spectra between the target and the three repeats is shown in **Figure 6** in the manuscript, which demonstrates the high synthetic reproducibility offered by the autonomous platform.

Label	Average Peak Position (nm)	Standard Deviation (nm)
**N1 (L1-5)**	799.6	6.5
**N2 (L1-1)**	525.9	0.9
**N3 (L2-12-2)**	530.0	1.2
**N4 (L2-7)**	777.0	6.9
**N5 (L3-3)**	673.4	2.6
**N6 (L3-1)**	561.0	3.4

Table S22. The average peak position and the standard deviation of the highest peak for N1 to N6.

5.3. The unique digital signature of nanoparticles

It is important to create the unique digital signatures of nanoparticles from various synthetic conditions regarding their wide applications. Here we introduce how to create unique digital signatures using the information of synthetic procedures as well as the validation of synthesised

nanoparticles. Since there are multiple ways to describe the same synthetic procedure, we implemented the universal chemical description language, χDL (*47*), to describe the procedure. Depending on the type of nanoparticles, various techniques such as electron microscopy, dynamic light scattering, and small-angle X-ray scattering can be used to validate the synthesis of nanoparticles. Considering the plasmonic effect of AuNPs, in-line UV-Vis was used to validate the synthesis.

The digital signature of one nanoparticle sample can be generated using the hash function as follows:

1. Both the chemicals and the operations required to reproduce the sample are described using χDL.
2. The chemical synthesis of the sample is conducted, and the product is validated with the original sample.
3. A "True" or "False" statement is appended to the string format of χDL depending on if the sample is reproduced as desired.
4. The string including χDL as well as the True/False statement is encoded with UTF-8, and further converted to the unique digital signature using the hash function of SHA-256.

All the chemicals were represented using their CAS numbers, with corresponding concentrations in the unit of M (molar per litre). In the synthesis where a nanoparticle solution was used as the seed, its digital signature was used in the χDL file, and would participate in hashing the new nanoparticles. For each of the six reproduced nanoparticles, the actual growth time was calculated according to the time difference between the last synthesis operation (adding seeds) and the UV-Vis analysis, and was used in the χDL file.

The code for χDL is available at https://croningroup.gitlab.io/chemputer/xdl/standard/index.html and https://gitlab.com/croningroup/chemputer/xdl.git. χDL 1 was used in this work. The code of the specific version of the χDL package (1.6.0) that was used to generate the digital signatures of the six AuNPs is available at https://gitlab.com/croningroup/chemputer/xdl/-/tree/v1.6.0.

REFERENCES AND NOTES

1. L. Cheng, C. Wang, L. Feng, K. Yang, Z. Liu, Functional nanomaterials for phototherapies of cancer. *Chem. Rev.* **114**, 10869–10939 (2014).

2. B. Radisavljevic, A. Radenovic, J. Brivio, V. Giacometti, A. Kis, Single-layer MoS_2 transistors. *Nat. Nanotechnol.* **6**, 147–150 (2011).

3. S. Cao, F. F. Tao, Y. Tang, Y. Li, J. Yu, Size- and shape-dependent catalytic performances of oxidation and reduction reactions on nanocatalysts. *Chem. Soc. Rev.* **45**, 4747–4765 (2016).

4. T. B. Hoang, G. M. Akselrod, M. H. Mikkelsen, Ultrafast room-temperature single photon emission from quantum dots coupled to plasmonic nanocavities. *Nano Lett.* **16**, 270–275 (2016).

5. I. O. Sosa, C. Noguez, R. G. Barrera, Optical properties of metal nanoparticles with arbitrary shapes. *J. Phys. Chem. B* **107**, 6269–6275 (2003).

6. Y. H. Yu, C. C. M. Ma, C. C. Teng, Y. L. Huang, S. H. Lee, I. Wang, M. H. Wei, Electrical, morphological, and electromagnetic interference shielding properties of silver nanowires and nanoparticles conductive composites. *Mater. Chem. Phys.* **136**, 334–340 (2012).

7. L. Yang, Z. Wang, L. Ma, A. Li, J. Xin, R. Wei, H. Lin, R. Wang, Z. Chen, J. Gao, The roles of morphology on the relaxation rates of magnetic nanoparticles. *ACS Nano* **12**, 4605–4614 (2018).

8. S. E. Lohse, C. J. Murphy, The quest for shape control: A history of gold nanorod synthesis. *Chem. Mater.* **25**, 1250–1261 (2013).

9. W. Shepherd, M. Wilms, J. Van Embden, E. D. Gaspera, Accurate control of stoichiometry and doping in barium stannate perovskite oxide nanoparticles. *Chem. Commun.* **55**, 11880–11883 (2019).

10. L. Scarabelli, A. Sánchez-Iglesias, J. Pérez-Juste, L. M. Liz-Marzán, A “tips and tricks” practical guide to the synthesis of gold nanorods. *J. Phys. Chem. Lett.* **6**, 4270–4279 (2015).

11. A. Ali, H. Zafar, M. Zia, I. U. Haq, A. R. Phull, J. S. Ali, A. Hussain, Synthesis, characterization, applications, and challenges of iron oxide nanoparticles. *Nanotechnol. Sci. Appl.* **9**, 49–67 (2016).

12. E. M. Marlett, Electrochemical synthesis of organometallics. *Ann. N. Y. Acad. Sci.* **125**, 12–24 (1965).

13. K. Esumi, K. Matsuhisa, K. Torigoe, Preparation of rodlike gold particles by UV irradiation using cationic micelles as a template. *Langmuir* **11**, 3285–3287 (1995).

14. H. E. Lee, R. M. Kim, H. Y. Ahn, Y. Y. Lee, G. H. Byun, S. W. Im, J. Mun, J. Rho, K. T. Nam, Cysteine-encoded chirality evolution in plasmonic rhombic dodecahedral gold nanoparticles. *Nat. Commun.* **11**, 1–10 (2020).

15. B. Nikoobakht, M. A. El-Sayed, Preparation and growth mechanism of gold nanorods (NRs) using seed-mediated growth method. *Chem. Mater.* **15**, 1957–1962 (2003).

16. H. L. Wu, H. R. Tsai, Y. T. Hung, K. U. Lao, C. W. Liao, P. J. Chung, J. S. Huang, I. C. Chen, M. H. Huang, A comparative study of gold nanocubes, octahedra, and rhombic dodecahedra as highly sensitive SERS substrates. *Inorg. Chem.* **50**, 8106–8111 (2011).

17. I. Ojea-Jiménez, N. G. Bastús, V. Puntes, Influence of the sequence of the reagents addition in the citrate-mediated synthesis of gold nanoparticles. *J. Phys. Chem. C* **115**, 15752–15757 (2011).

18. G. Mountrichas, S. Pispas, E. I. Kamitsos, Effect of temperature on the direct synthesis of gold nanoparticles mediated by poly(dimethylaminoethyl methacrylate) homopolymer. *J. Phys. Chem. C* **118**, 22754–22759 (2014).

19. R. Baber, L. Mazzei, N. T. K. Thanh, A. Gavriilidis, An engineering approach to synthesis of gold and silver nanoparticles by controlling hydrodynamics and mixing based on a coaxial flow reactor. *Nanoscale* **9**, 14149–14161 (2017).

20. J. H. Lee, K. J. Gibson, G. Chen, Y. Weizmann, Bipyramid-templated synthesis of monodisperse anisotropic gold nanocrystals. *Nat. Commun.* **6**, 7571 (2015).

21. S. Steiner, J. Wolf, S. Glatzel, A. Andreou, J. M. Granda, G. Keenan, T. Hinkley, G. Aragon-Camarasa, P. J. Kitson, D. Angelone, L. Cronin, Organic synthesis in a modular robotic system driven by a chemical programming language. *Science* **363**, eaav2211 (2019).

22. D. S. Salley, G. A. Keenan, D. L. Long, N. L. Bell, L. Cronin, A modular programmable inorganic cluster discovery robot for the discovery and synthesis of polyoxometalates. *ACS Cent. Sci.* **6**, 1587–1593 (2020).

23. J. Li, S. G. Ballmer, E. P. Gillis, S. Fujii, M. J. Schmidt, A. M. E. Palazzolo, J. W. Lehmann, G. F. Morehouse, M. D. Burke, Synthesis of many different types of organic small molecules using one automated process. *Science* **347**, 1221–1226 (2015).

24. B. Burger, P. M. Maffettone, V. V. Gusev, C. M. Aitchison, Y. Bai, X. Wang, X. Li, B. M. Alston, B. Li, R. Clowes, N. Rankin, B. Harris, R. S. Sprick, A. I. Cooper, A mobile robotic chemist. *Nature* **583**, 237–241 (2020).

25. J. Chang, P. Nikolaev, J. Carpena-Núñez, R. Rao, K. Decker, A. E. Islam, J. Kim, M. A. Pitt, J. I. Myung, B. Maruyama, Efficient closed-loop maximization of carbon nanotube growth rate using bayesian optimization. *Sci. Rep.* **10**, 1–9 (2020).

26. R. W. Epps, M. S. Bowen, A. A. Volk, K. Abdel-Latif, S. Han, K. G. Reyes, A. Amassian, M. Abolhasani, Artificial chemist: An autonomous quantum dot synthesis bot. *Adv. Mater.* **32**, 1–9 (2020).

27. S. Langner, F. Häse, J. D. Perea, T. Stubhan, J. Hauch, L. M. Roch, T. Heumueller, A. Aspuru-Guzik, C. J. Brabec, Beyond ternary OPV: High-throughput experimentation and self-driving laboratories optimize multicomponent systems. *Adv. Mater.* **32**, e1907801 (2020).

28. H. Tao, T. Wu, S. Kheiri, M. Aldeghi, A. Aspuru-Guzik, E. Kumacheva, Self-driving platform for metal nanoparticle synthesis: Combining microfluidics and machine learning. *Adv. Funct. Mater.* **2106725**, 1–9 (2021).

29. P. Raccuglia, K. C. Elbert, P. D. F. Adler, C. Falk, M. B. Wenny, A. Mollo, M. Zeller, S. A. Friedler, J. Schrier, A. J. Norquist, Machine-learning-assisted materials discovery using failed experiments. *Nature* **533**, 73–76 (2016).

30. A. O. Oliynyk, E. Antono, T. D. Sparks, L. Ghadbeigi, M. W. Gaultois, B. Meredig, A. Mar, High-throughput machine-learning-driven synthesis of full-heusler compounds. *Chem. Mater.* **28**, 7324–7331 (2016).

31. D. Salley, G. Keenan, J. Grizou, A. Sharma, S. Martín, L. Cronin, A nanomaterials discovery robot for the Darwinian evolution of shape programmable gold nanoparticles. *Nat. Commun.* **11**, 1–7 (2020).

32. H. Tao, T. Wu, M. Aldeghi, T. C. Wu, A. Aspuru-Guzik, E. Kumacheva, Nanoparticle synthesis assisted by machine learning. *Nat. Rev. Mater.* **6**, 701–716 (2021).

33. J. Li, J. Li, R. Liu, Y. Tu, Y. Li, J. Cheng, T. He, X. Zhu, Autonomous discovery of optically active chiral inorganic perovskite nanocrystals through an intelligent cloud lab. *Nat. Commun.* **11**, 1–10 (2020).

34. A. G. Kusne, H. Yu, C. Wu, H. Zhang, J. Hattrick-Simpers, B. DeCost, S. Sarker, C. Oses, C. Toher, S. Curtarolo, A. V. Davydov, R. Agarwal, L. A. Bendersky, M. Li, A. Mehta, I. Takeuchi, On-the-fly closed-loop materials discovery via Bayesian active learning. *Nat. Commun.* **11**, 1–11 (2020).

35. J. K. Pugh, L. B. Soros, K. O. Stanley, Quality diversity: A new frontier for evolutionary computation. *Front. Robot. AI.* **3**, 1–17 (2016).

36. J. Lehman, K. O. Stanley, Evolving a diversity of virtual creatures through novelty search and local comp, in *Proceedings of the 13th Annual Conference on Genetic and Evolutionary Computation* (GECCO, 2011) pp. 211–218.

37. J.-B. Mouret, J. Clune, Illuminating search spaces by mapping elites. arXiv: 1504.04909 (2015).

38. K. Arulkumaran, A. Cully, J. Togelius, Alphastar: An evolutionary computation perspective, in *Proceedings of the Genetic and Evolutionary Computation Conference Companion* (GECCO, 2019), pp. 314–315.

39. R. Kaushik, P. Desreumaux, J. B. Mouret, Adaptive prior selection for repertoire-based online adaptation in robotics. *Front. Robot. AI.* **6**, 151 (2020).

40. J. Verhellen, J. Van Den Abeele, Illuminating elite patches of chemical space. *Chem. Sci.* **11**, 11485–11491 (2020).

41. J. Grizou, L. J. Points, A. Sharma, L. Cronin, A curious formulation robot enables the discovery of a novel protocell behavior. *Sci. Adv.* **6**, eaay4237 (2020).

42. A. Rao, M. Schoenenberger, E. Gnecco, T. Glatzel, E. Meyer, D. Brändlin, L. Scandella, Characterization of nanoparticles using atomic force microscopy. *J. Phys. Conf. Ser.* **61**, 971–976 (2007).

43. A. E. Vladár, V. D. Hodoroaba, *Characterization of nanoparticles by scanning electron microscopy* (Elsevier Inc., 2019); http://dx.doi.org/10.1016/B978-0-12-814182-3.00002-X).

44. Z. L. Wang, Transmission electron microscopy of shape-controlled nanocrystals and their assemblies. *J. Phys. Chem. B* **104**, 1153–1175 (2000).

45. T. Zheng, S. Bott, Q. Huo, Techniques for accurate sizing of gold nanoparticles using dynamic light scattering with particular application to chemical and biological sensing based on aggregate formation. *ACS Appl. Mater. Interfaces* **8**, 21585–21594 (2016).

46. T. Li, A. J. Senesi, B. Lee, Small angle x-ray scattering for nanoparticle research. *Chem. Rev.* **116**, 11128–11180 (2016).

47. S. Hessam, M. Craven, A. I. Leonov, G. Keenan, L. Cronin, A universal system for digitization and automatic execution of the chemical synthesis literature. *Science* **370**, 101–108 (2020).

48. M. J. Walsh, S. J. Barrow, W. Tong, A. M. Funston, J. Etheridge, Symmetry breaking and silver in gold nanorod growth. *ACS Nano* **9**, 715–724 (2015).

49. K. Gui, J. Zheng, K. Wang, D. Li, S. Zhuang, FDTD modelling of silver nanoparticles embedded in phase separation interface of H-PDLC. *J. Nanomater.* **2015**, 1–7 (2015).

50. J. Marcheselli, D. Chateau, F. Lerouge, P. Baldeck, C. Andraud, S. Parola, S. Baroni, S. Corni, M. Garavelli, I. Rivalta, Simulating plasmon resonances of gold nanoparticles with bipyramidal shapes by boundary element methods. *J. Chem. Theory Comput.* **16**, 3807–3815 (2020).

51. B. T. Draine, P. J. Flatau, Discrete-dipole approximation for scattering calculations. *J. Opt. Soc. Am. A* **11**, 1491–1499 (1994).

52. N. B. Piller, O. J. F. Martin, Increasing the performance of the coupled-dipole approximation: A spectral approach. *IEEE Trans. Antennas Propag.* **46**, 1126–1137 (1998).

53. M. A. Yurkin, M. Min, A. G. Hoekstra, Application of the discrete dipole approximation to very large refractive indices: Filtered coupled dipoles revived. *Phys. Rev. E Stat. Nonlinear, Soft Matter Phys.* **82**, 1–12 (2010).

54. P. Van Rysselberghe, Remarks concerning the clausius-mossotti law. **36**, 1152–1155 (1932).

55. Q. Wang, Z. Wang, Z. Li, J. Xiao, H. Shan, Z. Fang, L. Qi, Controlled growth and shape-directed self-assembly of gold nanoarrows. *Sci. Adv.* **3**, e1701183 (2017).

56. V. Thambi, A. Kar, P. Ghosh, D. Paital, A. R. S. Gautam, S. Khatua, Synthesis of complex nanoparticle geometries via pH-controlled overgrowth of gold nanorods. *ACS Omega* **4**, 13733–13739 (2019).

57. K. S. Lee, M. A. El-Sayed, Dependence of the enhanced optical scattering efficiency relative to that of absorption for gold metal nanorods on aspect ratio, size, end-cap shape, and medium refractive index. *J. Phys. Chem. B* **109**, 20331–20338 (2005).
